# Boron-containing capsaicinoids†

**DOI:** 10.1039/d1ra04943g

**Published:** 2021-07-23

**Authors:** Jennifer A. Melanson, Maxim F. Landry, Martin Lanteigne, Katherine McQuillan, Hebelin Correa, Russell G. Kerr, Stephen A. Westcott

**Affiliations:** Department of Chemistry and Biochemistry, Mount Allison University Sackville NB E4L 1G8 Canada swestcott@mta.ca jamelanson@mta.ca mflandry@mta.ca; Nautilus Biosciences CRODA Canada Inc., Duffy Research Centre Charlottetown PE C1A 4P3 Canada martin.lanteigne@croda.com katherine.mcquillan@croda.com hebelin.correa@croda.com; Department of Chemistry, University of Prince Edward Island Charlottetown PE C1A 4P3 Canada rkerr@upei.ca

## Abstract

This study reports on the preparation of eight new boron-containing capsaicinoids bearing long aliphatic chains, as an expansion of our previous studies to include tertiary amide derivatives into our substrate scope. Our boron-moiety, a pinacolboronate ester (Bpin) fragment, has been incorporated in two locations: as an aryl substituent of the capsaicinoid produced by the reductive amination of veratraldehyde, or at the terminal end of an aliphatic substituent using an iridium catalyzed hydroboration reaction. We report that most compounds in our series show moderate antimicrobial and cytotoxic activity, surpassing activities noted in our previous study.

## Abstract 2

This study reports on the preparation of eight new boron-containing capsaicinoids bearing long aliphatic chains, and initial bioactivities are reported.

## Graphical abstract

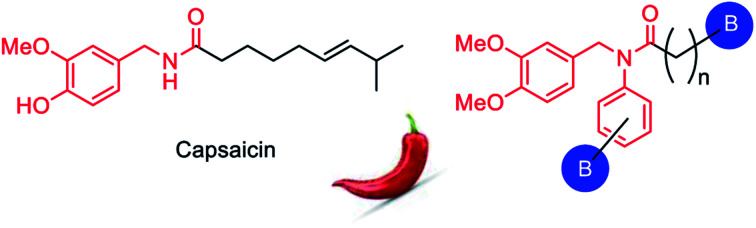

## Introduction

**Fig. 1:**
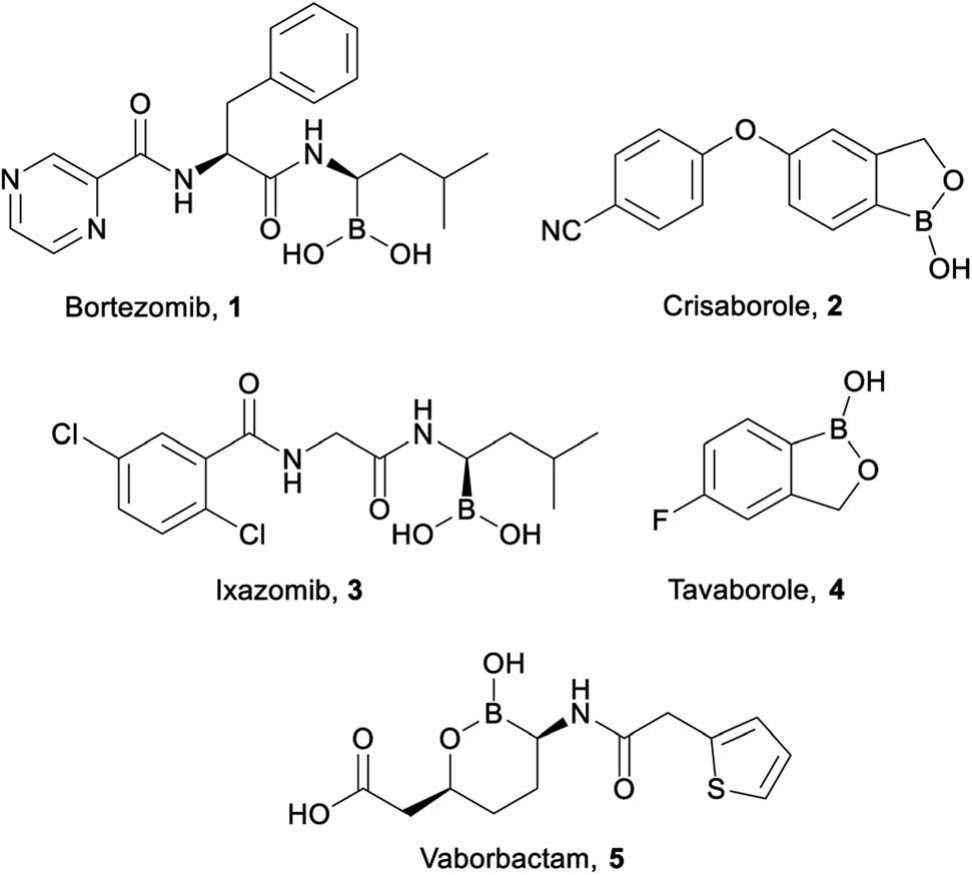
FDA approved organoboron compounds.

Organoboron compounds are beginning to receive well-warranted attention in the pharmaceutical industry, including five drugs recently approved by the FDA (Fig. 1): Bortezomib 1 (Velcade, injected cancer therapy),^1,2^ Crisaborole 2 (Eucrisa, topical eczema treatment),^3^ Ixazomib 3 (Ninlaro, oral cancer therapy),^4–6^ Tavaborole 4 (Kerydin, topical anti-fungal),^7,8^ and Vaborbactam 5 (in combination with Meropenem as Vabomere, injected broad-spectrum antibiotic).^9–11^ Indeed, organoboron compounds display a wide variety of marketable pharmaceutical activities.^12,13^

**Fig. 2:**
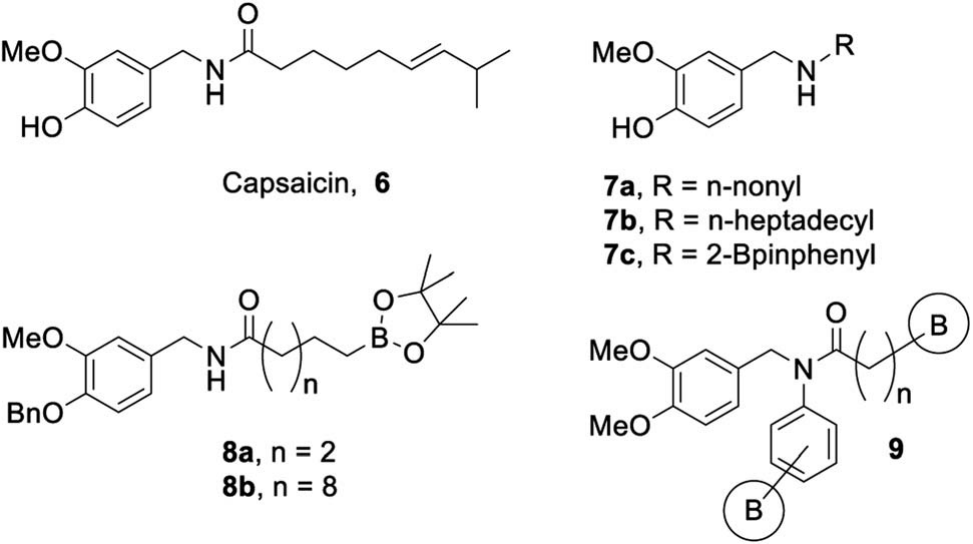
Capsaicin and biologically active capsaicinoids.

Our group has a long-standing interest in developing bioactive organoboron compounds,^14–34^ including boron-containing capsaicinoids.^35,36^ Capsaicin (6, Fig. 2) is the colourless, hydrophobic, crystalline compound^37^ that is responsible for the characteristic spicy “heat” flavouring of the chili fruits of the *Capsicum* genus. Capsaicin has many uses beyond the kitchen, including as an anticancer, antifungal, or antibacterial agent, as an insect or animal repellent, as an analgesic, and even in weight loss aid.^38^ Capsaicinoids are natural products that are structurally related to capsaicin, which also tend to display many of the same marketable properties.

To this extent, our initial study^35^ focused on the synthesis and biological activity of select capsaicinoids that bear either a long aliphatic moiety or incorporate boron as a substituent on a phenyl group. These compounds were screened against *Mycobacterium tuberculosis* H37Ra, and it was found that substrates bearing long aliphatic chains (Fig. 2, 7a/b) demonstrated greater bioactivity.^35^ In addition, our boron-containing capsaicinoid (Fig. 2, 7c) showed comparable bioactivity to our boron-omitted lipophilic substrates.^35^ Our second study showcases two boron-capsaicinoids bearing either a short or a long aliphatic tail, with the boron moiety attached to the terminal end of this tail (Fig. 2, 8a/b).^36^ Both compounds demonstrated antibacterial activity against vancomycin-resistant *Enterococcus*, and the more lipophilic compound (Fig. 2, 8b) displayed broader antimicrobial activity.^36^ Based on the results of both studies,^35,36^ we have chosen to expand the substrate scope to include boron-containing capsaicinoids featuring tertiary amides that bear a long aliphatic tail (Fig. 2, 9).

## Results and discussion

### Chemistry

**Scheme 1:**
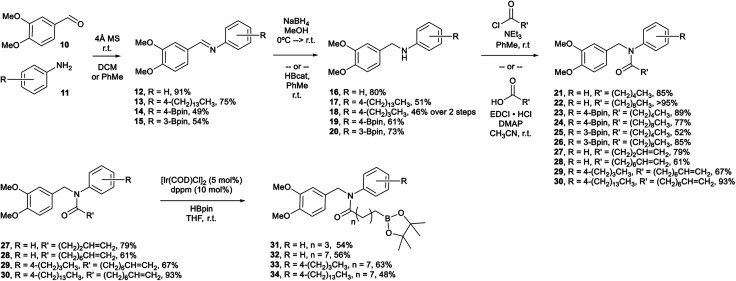
Synthesis of boron-containing capsaicinoids.

Veratraldehyde (Scheme 1, 10) is the methylated derivative of vanillin and is used commercially as a fragrant and flavourant,^39^ as well as a starting material in the synthesis of some pharmaceuticals (*e.g.*, Tiapamil,^40–42^ and Verazide^43,44^). In our previous study,^35^ we chose vanillin as our starting aldehyde, but its use requires additional protection and deprotection steps for the compound to be suitable in the methodology employed. Consequently, we decided to use 10 as a replacement.

In our current study, we have shown that the amidation of the boro-aryl amine substrates (19, 20) employed a saturated aliphatic acyl chloride to provide the final tertiary amide capsaicinoid (23–26) in good yield without further manipulation.

We have also prepared eight boron-containing capsaicinoids (Scheme 1, 23–26/31–34) in 3–4 steps from 10, as well as two substrates that do not contain a boron moiety (Scheme 1, 21/22) using the same methodology. We have prepared these substrates by a divergent synthesis, beginning with the reductive amination of 10 and either a desboro or boro-aryl primary amine (11). Our desboro-aryl amine substrates either were unsubstituted (16) or bore aliphatic chains (17, 18). We have previously found that increasing the length of aliphatic substituents on our boron-containing capsaicinoids provided greater antimicrobial activity,^36^ and so functionalization at this position provided an opportunity to further tune the lipophilicity of the capsaicinoid. Amines 16–18 were isolated in modest to good yield.

The desboro-aryl amine substrates were subjected to EDCI-assisted amidation using terminally unsaturated aliphatic acids to provide intermediates (27–30) towards the final tertiary amide capsaicinoids (31–34). These intermediates were subjected to metal-catalyzed hydroboration, employing [IrCl(cod)(dppm)]^45^ and pinacolborane (HBpin, pin = 1,2-O_2_C_2_Me_4_) at room temperature. The postulated catalytic cycle is anticipated to proceed *via* the generally accepted Ir(i/iii) pathway,^46^ whereby loss of the cod ligand opens up coordination sites for the subsequent B–H bond activation step. Coordination of the alkene followed by insertion into either the Ir–H (shown) or the Ir–B bond would generate an iridium alkylboronate ester intermediate. Reductive elimination would afford the expected linear product. This methodology was successful in generating the amide capsaicinoids 31–34 in modest yields.

### Biology

Our 8 boron-containing capsaicinoids were evaluated for antimicrobial and cytotoxic activities. Antimicrobial assays were performed using methicillin-resistant *Staphylococcus aureus* MRSA 310, vancomycin-resistant *Enterococcus faecium* VRE 379, *Staphylococcus warneri* (ATCC 17917), *Proteus vulgaris* (ATCC 12454), *Pseudomonas aeruginosa* (ATCC 14210), and *Candida albicans* (ATCC 14035). Compound 25 showed moderate activity against MRSA, VRE, and *S. warneri*, whereas compound 31 showed a low activity against MRSA and *S. warneri* and compound 32 was only active against MRSA (Table 1). Interestingly, compound 26 was also selectively active against the MRSA pathogen and was able to be administered at the lowest dosage in the antimicrobial series to achieve 90% growth inhibition at a concentration of 15.3 μM (compared to the control antibiotic Vancomycin that demonstrates MIC90 of MRSA at 1.4 μM). Compounds 23, 24, 33, and 34 were inactive against all tested pathogens. None of the boron-containing capsaicinoids showed activity against *P. aeruginosa*, *P. vulgaris*, and *C. albicans*.

**Table 1:** Antimicrobial activities^a^

Compound (μM)	MRSA		VRE		*S. warneri*	
	MIC90	IC_50_	MIC90	IC_50_	MIC90	IC_50_
25	136.9	70.6 ± 6.6	273.8	91.6 ± 6.8	136.9	78.3 ± 39.8
26	15.3	13.2 ± 0.0	Inactive	Inactive	Inactive	Inactive
31	Inactive	120.6 ± 2.0	Inactive	Inactive	282.3	135.0 ± 15.4
32	Inactive	153.9 ± 37.1	Inactive	Inactive	Inactive	Inactive
Control	1.4^a^	0.5 ± 0.1^a^	2.4^b^	1.2 ± 0.1^b^	0.3^a^	0.3 ± 0.1^a^
8a*	Inactive	107.1	Inactive	Inactive	—	—
8b*	Inactive	244.7	Inactive	276.7	—	—
Phenylboronic acid*	Inactive	169.6	Inactive	Inactive	—	—
Capsaicin*	Inactive	Inactive	Inactive	Inactive	—	—

a Inhibition at concentrations above 300 μM was considered inactive. Controls: (a) Vancomycin, (b) Rifampicin. Reported activity is the average ± SD. *Data collected in previous study, inhibition above 400 μM was considered inactive; activity against *S. warneri* was not previously studied.^36^

In addition to the antimicrobial study, our boron-containing capsaicinoid series was also submitted to cytotoxic assays using human foreskin BJ fibroblast cells (ATCC CRL-2522), green monkey kidney cells Vero (ATCC CCL-81), human breast adenocarcinoma cells (ATCC HTB-26 MDA-MB 231 and MCF7. ATCC HTB-22), and human colorectal carcinoma cells (HCT116 ATCC CCL-247). Compounds 23, 25, and 31 showed a selective but low to moderate activity against the three cancer cell lines tested. Compounds 24, 26, 32, and 33 demonstrated unselective cytotoxic effects across all cell lines, with compound 33 being the most cytotoxic compound of our study. In addition to being broadly cytotoxic, compound 33 showed notable activity against MCF7 (with an IC_50_ of 3.4 μM). The most lipophilic capsaicinoid, compound 34, was found to be inactive against all screened cell lines (Table 2).

**Table 2:** Cytotoxicity activities^a^

Compound (IC_50_ μM)	Normal cell lines		Cancer cell lines		
	Vero	BJ	HCT	HTB	MCF7
23	Inactive	Inactive	117.7 ± 4.9	85.8 ± 7.1	Inactive
24	26.9 ± 4.4	36.1 ± 0.8	33.8 ± 2.1	25.2 ± 1.3	24.1 ± 3.8
25	Inactive	Inactive	136.7 ± 12.0	76.1 ± 3.4	117.7 ± 19.5
26	47.2 ± 6.7	38.6 ± 1.0	44.3 ± 10.5	31.5 ± 2.7	35.7 ± 16.2
31	Inactive	Inactive	125.9 ± 16.5	58.0 ± 3.5	108.3 ± 30.9
32	77.1 ± 8.4	111.7 ± 10.0	80.1 ± 3.9	42.6 ± 1.8	68.5 ± 15.1
33	20.9 ± 1.2	24.2 ± 0.2	20.3 ± 0.7	21.2 ± 3.5	3.4 ± 2.2
Doxorubicin	>14.7	>14.7	<1.2 × 10^−4^	0.4 ± 0.2	0.2 ± 0.2

a Inhibition at concentrations above 300 μM was considered inactive. Reported activity is the average ± SD.

## Conclusions

As a result of this study, we have synthesized fourteen capsaicinoids in 3–4 steps from commercially available veratraldehyde in modest to good yield. Most compounds demonstrated low to moderate activity in cytotoxicity assays, but compound 33 displayed the most notable activity against MCF7. Unfortunately, 33 was also found to be cytoxic against normal cell lines. Compounds 23, 25, and 31 were found to be selectively cytotoxic against cancer cell lines, but the activity displayed was very modest. In our antimicrobial studies, notable antibacterial activity was displayed from compound 26 against MRSA. Although the compound does not best the industry standard, it does outperform our previously published substrates.^36^ We feel that the antimicrobial and cytotoxic assays warrant additional examination of capsaicinoids bearing boron-moieties to further tailor bioactivity. Further studies are in progress and will be reported in due course.

## Experimental

### Materials and methods

Reagents and solvents were obtained from Sigma-Aldrich, Strem Chemicals, TCI Chemicals, or Alfa Aesar. Where specified, manipulations under an inert atmosphere were performed in a MB Unilab glove box produced by MBraun or using standard Schlenk techniques. NMR spectra were recorded on a JEOL JNM-GSX400 FT NMR (^1^H: 400 MHz; ^11^B: 128 MHz; ^13^C: 100 MHz) spectrometer. ^1^H, ^13^C{^1^H}, and ^11^B{^1^H} NMR chemical shifts (*δ*/ppm) are referenced to Me_4_Si, Me_4_Si, and BF_3_·OEt_2_, respectively. Chemical shifts (*δ*) are reported in ppm [relative to residual solvent peaks (^1^H and ^13^C) or external BF_3_·OEt_2_ (^11^B). Multiplicities are reported as singlet (s), doublet (d), triplet (t), quartet (q), quintet (quint), or multiplet (m), with broad (br) or apparent (a) added as a prefix where necessary, and coupling constants (*J*) reported in hertz. Melting points were measured uncorrected with a Stuart SMP30 apparatus. High resolution mass spectrometry was performed at DalChem Mass Spec Laboratory (Dalhousie University, Halifax, NS).

#### General procedure 1

##### Synthesis of diarylimines

A solution of 3,4-dimethoxybenzaldehyde (1 equiv.) in DCM [0.6 M] was added to an Erlenmeyer flask equipped with a layer of 4 Å MS, after which the prescribed aniline substrate (1–1.5 equiv.) was added and the vessel was sealed. The resulting solution was reacted at ambient temperature (20 °C) for 4 days before decanting to remove the 4 Å MS and concentrating the reaction mixture *in vacuo*. The resulting oil/solid was dissolved in hot EtOH and then allowed to cool to RT. The precipitate was filtered and washed with hexanes, and then recrystallized from EtOH to afford the desired product as a colourless, crystalline solid.

#### General procedure 2

##### Synthesis of BPin substituted diarylimines

A solution of 3,4-dimethoxybenzaldehyde (1 equiv.) in PhMe [0.2 M] was added to an Erlenmeyer flask equipped with a layer of 4 Å MS under an inert atmosphere (glove box) after which the prescribed BPin substituted aniline substrate (1 equiv.) was added and the vessel was sealed. The resulting solution reacted at ambient temperature (20 °C) for 2 weeks before decanting to remove the 4 Å MS and concentrating the reaction mixture *in vacuo*. The resulting crude oil/solid was dissolved in hot EtOH and then allowed to cool to RT. The precipitate was filtered and washed with hexanes, and then recrystallized from EtOH to afford the desired product as a colourless, crystalline solid.

##### Synthesis of 1-(3,4-dimethoxyphenyl)-*N*-phenylmethanimine (12)

General procedure 1 was followed using aniline (1.10 mL, 12.0 mmol) and 3,4-dimethoxybenzaldehyde (2.00 g, 12.0 mmol) to give the title compound as a colourless solid. Yield: 2.63 g (91%). MP (EtOH) 75.7–78.0 °C; ^1^H NMR (400 MHz, in CDCl_3_-d_1_): *δ* 8.35 (s, 1H), 7.62 (br d, 1H), 7.40–7.36 (m, 2H), 7.29 (at, 1H), 7.23–7.19 (m, 3H), 6.91 (br d, 1H), 3.97 (br d, 3H), 3.92 (br d, 3H) ppm; ^13^C NMR (100 MHz, in CDCl_3_-d_1_): *δ* 159.9, 152.3, 152.0, 149.4, 129.6, 129.2, 125.6, 124.5, 120.9, 110.4, 108.8, 56.0 ppm. Comparable to literature.^47^

##### Synthesis of 1-(3,4-dimethoxyphenyl)-*N*-(4-tetradecylphenyl)methanimine (13)

General procedure 1 was followed using 4-tetradecylaniline (1.74 g, 6.02 mmol) and 3,4-dimethoxybenzaldehyde (1.00 g, 6.02 mmol) to give the title compound as a colourless solid. Yield: 1.97 g (75%). MP (EtOH) 68.0–68.8 °C; FT-IR (neat): *ν*_max_ 2914 (s), 2848 (m), 1624 (w), 1595 (m), 1580 (m), 1512 (s), 1270 (s), 1144 (m) cm^−1^; ^1^H NMR (400 MHz, in CDCl_3_-d_1_): *δ* 8.38 (s, 1H), 7.61 (s, 1H), 7.28 (d, 1H, *J* = 8.2 Hz), 7.19 (d, 2H, *J* = 8.3 Hz), 7.14 (d, 2H, *J* = 8.3 Hz), 6.93 (d, 1H, *J* = 8.2 Hz), 3.99 (s, 3H), 3.95 (s, 3H), 2.61 (t, 2H, *J* = 7.7 Hz), 1.66–1.59 (m, 2H), 1.31–1.26 (m, 22H), 0.88 (t, 3H, *J* = 6.8 Hz) ppm; ^13^C NMR (100 MHz, in CDCl_3_-d_1_): *δ* 159.2, 152.0, 149.9, 149.5, 140.7, 129.9, 129.2, 124.4, 120.9, 110.5, 108.9, 56.1, 35.6, 32.1, 31.7, 29.82, 29.75, 29.7, 29.5, 29.4, 22.8, 14.3 ppm; HRMS (ESI) calc'd for C_29_H_44_NO_2_^+^ [M + H]^+^ 438.3367, found 438.3349.

##### Synthesis of 1-(3,4-dimethoxyphenyl)-*N*-(4-(4,4,5,5-tetramethyl-1,3,2-dioxaborolan-2-yl)phenyl)methanimine (14)

General procedure 2 was followed using 4-(4,4,5,5-tetramethyl-1,3,2-dioxaborolan-2-yl)aniline (1.12 g, 5.11 mmol) and 3,4-dimethoxybenzaldehyde (0.850 g, 5.11 mmol) to give the title compound as a colourless solid. Yield: 0.918 g (49%). MP (EtOH) 114.0–114.2 °C; FT-IR (neat): *ν*_max_ 2976 (w), 2934 (w), 1626 (w), 1593 (m), 1580 (m), 1267 (s), 1135 (s) cm^−1^; ^1^H NMR (400 MHz, in CDCl_3_-d_1_): *δ* 8.35 (s, 1H), 7.84 (d, 2H, *J* = 8.3 Hz), 7.61 (d, 1H, *J* = 1.8 Hz), 7.31 (dd, 1H, *J* = 8.2, 1.8 Hz), 7.17 (d, 2H, *J* = 8.3 Hz), 6.93 (d, 1H, *J* = 8.2 Hz), 3.99 (s, 3H), 3.95 (s, 3H), 1.35 (s, 12H) ppm; ^13^C NMR (100 MHz, in CDCl_3_-d_1_): *δ* 160.4, 155.0, 152.2, 149.6, 136.0, 129.6, 124.8, 120.4, 110.5, 109.0, 83.9, 56.1, 25.0 ppm; ^11^B NMR (128 MHz, in CDCl_3_-d_1_):*δ* 30.4 ppm; HRMS (ESI) calc'd for C_21_H_27_BNO_4_^+^ [M + H]^+^ 368.2028, found 368.2014.

##### Synthesis of 1-(3,4-dimethoxyphenyl)-*N*-(3-(4,4,5,5-tetramethyl-1,3,2-dioxaborolan-2-yl)phenyl)methanimine (15)

General procedure 2 was followed using 3-(4,4,5,5-tetramethyl-1,3,2-dioxaborolan-2-yl)aniline (1.12 g, 5.11 mmol) and 3,4-dimethoxybenzaldehyde (0.850 g, 5.11 mmol) to give the title compound as a colourless solid. Yield: 1.01 g (54%). MP (EtOH) 124.0–124.5 °C; FT-IR (neat): *ν*_max_ 2982 (w), 2930 (w), 1619 (m), 1567 (m), 1266 (m), 1135 (s) cm^−1^; ^1^H NMR (400 MHz, in CDCl_3_-d_1_): *δ* 8.41 (s, 1H), 7.67–7.65 (m, 1H), 7.62 (br s, 2H), 7.40 (at, 1H), 7.35–7.29 (m, 2H), 6.93 (d, 1H, *J* = 8.2 Hz), 3.98 (s, 3H), 3.95 (s, 3H), 1.36 (s, 12H) ppm; ^13^C NMR (100 MHz, in CDCl_3_-d_1_): *δ* 160.0, 152.0, 151.7, 149.5, 132.1, 129.8, 128.7, 126.2, 124.9, 124.5, 110.5, 108.9, 84.0, 56.1, 25.0 ppm; ^11^B NMR (128 MHz, in CDCl_3_-d_1_): *δ* 30.1 ppm; HRMS (ESI) calc'd for C_21_H_27_BNO_4_^+^ [M + H]^+^ 368.2028, found 368.2029.

#### General procedure 3

##### Synthesis of diarylamines

A stirring solution of diarylimine (1 equiv.) in MeOH [0.25 M] cooled to 0 °C, and NaBH_4_ (2.5 equiv.) was slowly added over 5–10 minutes (portion wise). The reaction gradually increased from 0 °C to ambient temperature and was stirred overnight before the solvent was removed *in vacuo*. The crude oil was partitioned between EtOAC and water, extracted with EtOAc, and the organic fractions were dried over MgSO_4_, filtered, and concentrated *in vacuo*. The resulting oil/solid was dissolved in hot EtOH and then allowed to cool to RT. The precipitate was filtered and washed with hexanes, and then recrystallized from EtOH to afford the desired product as a colourless, crystalline solid.

#### General procedure 4

##### Synthesis of diarylamines

A solution of HBCat (2 equiv.) in PhMe [1.5 M] was added to a stirring solution of diarylimine (1 equiv.) in PhMe [0.3 M] and was stirred for 48 hours before the solvent was removed *in vacuo*. The crude oil was partitioned between DCM and water, extracted with DCM, and the organic fractions were dried over MgSO_4_, filtered, and concentrated *in vacuo*. The resulting oil/solid was dissolved in hot EtOH and then allowed to cool to RT. The precipitate was filtered and washed with hexanes, and then recrystallized from EtOH to afford the desired product as a colourless, crystalline solid.

##### Synthesis of *N*-(3,4-dimethoxybenzyl)phenylamine (16)

General procedure 3 was followed using 12 (1.00 g, 4.14 mmol) to give the title compound as a colourless solid. Yield: 0.804 g (80%). MP (EtOH) 78.6–79.7 °C; ^1^H NMR (400 MHz, in CDCl_3_-d_1_): *δ* 7.21–7.15 (m, 2H), 6.93–6.91 (m, 2H), 6.85–6.82 (m, 1H), 6.75–6.70 (m, 1H), 6.65 (br d, 2H), 4.25 (s, 2H), 3.96 (br s, 1H), 3.874 (s, 3H), 3.867 (s, 3H) ppm; ^13^C NMR (100 MHz, in CDCl_3_-d_1_): *δ* 149.2, 148.3, 148.3, 132.0, 129.4, 119.8, 117.7, 113.0, 111.2, 110.8, 56.0, 48.3 ppm. Comparable to literature.^48^

##### Synthesis of 4-tetradecyl-*N*-(3,4-dimethoxybenzyl)aniline (17)

General procedure 3 was followed using 13 (1.70 g, 3.88 mmol) to give the title compound as a colourless solid. Yield: 0.869 g (51%). MP (EtOH) 73.5–74.9 °C; FT-IR (neat): *ν*_max_ 3374 (m), 2919 (s), 2849 (s), 1614 (m), 1513 (s), 1464 (s), 1259 (s), 1231 (s), 1139 (s) cm^−1^; ^1^H NMR (400 MHz, in CDCl_3_-d_1_): *δ* 7.00 (d, 2H, *J* = 8.3 Hz), 6.92 (ad, 2H), 6.84 (d, 1H, *J* = 8.7 Hz), 6.60 (d, 2H, *J* = 8.3 Hz), 4.23 (s, 2H), 3.88–3.87 (m, 6H), 2.49 (t, 2H, *J* = 7.7 Hz), 1.60–1.51 (m, 2H), 1.37–1.21 (m, 22H), 0.88 (t, 3H) ppm; ^13^C NMR (100 MHz, in CDCl_3_-d_1_): *δ* 149.2, 148.3, 132.4, 132.2, 129.3, 119.9, 113.1, 111.2, 110.9, 56.1, 56.0, 48.8, 35.2, 32.1, 32.0, 29.83, 29.80, 29.78, 29.7, 29.51, 29.46, 22.8, 14.3 ppm; HRMS (ESI) calc'd for C_29_H_46_NO_2_^+^ [M + H]^+^ 440.3523, found 440.3539.

##### Synthesis of 4-butyl-*N*-(3,4-dimethoxybenzyl)aniline (18; 2 step procedure)

A solution of 3,4-dimethoxybenzaldehyde (1 g, 6.018 mmol) in DCM (10 mL) was added to an Erlenmeyer flask equipped with a layer of 4 Å MS, after which 4-butylaniline (2.00 mL, 12.0 mmol) was added and the vessel was sealed. The resulting solution reacted at room temperature (20 °C) for 1 week before decanting to remove the 4 Å MS and concentrating the solvent *in vacuo*. The resulting oil/solid was then dissolved in MeOH (24 mL) and cooled to 0 °C, and NaBH_4_ (0.524 g, 15.0 mmol) was added portion wise. The reaction gradually increased from 0 °C to ambient temperature and was stirred overnight before the solvent was removed *in vacuo*. The crude concentration was partitioned between EtOAC and water, extracted with EtOAc, and the organic fractions were dried over MgSO_4_, filtered, and concentrated *in vacuo*. Precipitation and recrystallization from EtOH provided the title compound as a colourless crystalline solid. Yield: 0.824 g (46% isolated yield over 2 steps). MP (EtOH) 70.3–72.6 °C; FT-IR (neat): *ν*_max_ 3375 (m), 2958–2835 (w), 16.16 (m), 1514 (s), 1258 (m), 1232 (s), 1138 (s) cm^−1^; ^1^H NMR (400 MHz, in CDCl_3_-d_1_): *δ* 7.02–7.00 (m, 2H), 6.93–6.92 (m, 2H), 6.85–6.83 (m, 1H), 6.60 (ad, 2H), 4.24 (s, 2H), 3.88 (ad, 6H) 2.51 (at, 2H), 1.59–1.51 (m, 2H), 1.39–1.30 (m, 2H), 0.94–0.90 (m, 3H) ppm; ^13^C NMR (100 MHz, in CDCl_3_-d_1_): *δ* 149.2, 148.2, 146.3, 132.25, 132.22, 129.2, 119.8, 113.0, 111.2, 110.9, 56.0, 55.9, 48.7, 34.8, 34.1, 22.4, 14.1 ppm; HRMS (ESI) calc'd for C_19_H_26_NO_2_^+^ [M + H]^+^ 300.1958, found 300.1969.

##### Synthesis of 3,4-dimethoxy-*N*-[4-(4,4,5,5-tetramethyl-1,3,2-dioxaborolan-2-yl)phenyl]-benzenemethanamine (19)

General procedure 4 was followed using 14 (0.340 g, 0.930 mmol) to give the title compound as a colourless solid. Yield: 0.210 g (61%). MP (EtOH) 179.1–180.4 °C; FT-IR (neat): *ν*_max_ 3377 (m), 2980 (w), 2938 (w), 2838 (w), 1602 (s), 1512 (m), 1264 (s), 1138 (s) cm^−1^; ^1^H NMR (400 MHz, in CDCl_3_-d_1_): *δ* 7.64 (d, 2H, *J* = 8.1 Hz), 6.91–6.89 (m, 2H), 6.83 (d, 1H, *J* = 7.8 Hz), 6.62 (d, 2H, *J* = 8.1 Hz), 4.29 (s, 2H), 4.17 (s, 1H), 3.87 (s, 3H), 3.86 (s, 3H), 1.32 (s, 12H) ppm; ^13^C NMR (100 MHz, in CDCl_3_-d_1_): *δ* 150.8, 149.3, 148.4, 136.5, 131.6, 119.8, 112.0, 111.3, 110.8, 83.4, 56.1, 56.0, 47.8, 25.0 ppm; ^11^B NMR (128 MHz, in CDCl_3_-d_1_): *δ* 30.0 ppm; HRMS (ESI) calc'd for C_21_H_28_BNNaO_4_^+^ [M + Na]^+^ 392.2004, found 392.1995.

##### Synthesis of 3,4-dimethoxy-*N*-[3-(4,4,5,5-tetramethyl-1,3,2-dioxaborolan-2-yl)phenyl]-benzenemethanamine (20)

General procedure 4 was followed using 15 (0.550 g, 1.50 mmol) to give the title compound as a colourless solid. Yield: 0.405 g (73%). MP (EtOH) 119.1–121.5 °C; FT-IR (neat): *ν*_max_ 3415 (br), 29.75 (w), 2931 (w), 1602 (m), 1512 (m), 1247 (s), 1142 (s) cm^−1^; ^1^H NMR (400 MHz, in CDCl_3_-d_1_): *δ* 7.22–7.17 (m, 2H), 7.14–7.14 (1H, m), 6.91–6.89 (m, 2H), 6.84–6.82 (m, 1H), 6.74–6.71 (m, 1H), 4.28 (s, 2H), 3.92 (s, 1H), 3.88 (s, 3H), 3.87 (s, 3H), 1.33 (s, 12H) ppm; ^13^C NMR (100 MHz, in CDCl_3_-d_1_): *δ* 149.2, 148.3, 147.7, 132.1, 128.9, 124.1, 119.8, 119.3, 115.7, 110.9, 83.8, 56.0, 55.9, 48.4, 25.0 ppm; ^11^B NMR (128 MHz, in CDCl_3_-d_1_): *δ* 30.5 ppm; HRMS (ESI) calc'd for C_21_H_28_BNNaO_4_^+^ [M + Na]^+^ 392.2004, found 392.2011.

#### General procedure 5

##### Synthesis of diarylamides from acid chlorides

A solution of the prescribed acyl chloride (1 equiv.) in PhMe [0.1–0.4 M] was added to a stirring solution of amine (1 equiv.) in PhMe [0.2–0.7 M], followed by the addition of a solution of NEt_3_ (1.1 equiv.) in PhMe [0.2–0.8 M]. The resulting mixture was stirred under inert atmosphere for 20–24 h before a white precipitate was removed by filtration. The mother liquor was concentrated to produce a colourless solid that was recrystallized from EtOH (unless otherwise specified).

##### Synthesis of *N*-(3,4-dimethoxyphenylmethyl)-*N*-(phenyl)-hexanamide (21)

General procedure 5 was following using a solution of 16 (0.530 g, 2.20 mmol) in toluene (5 mL), of hexanoyl chloride (0.290 g, 2.20 mmol) in toluene (3 mL), and of Et_3_N (0.240 g, 2.40 mmol) in toluene (3 mL). The reaction was allowed to proceed under inert atmosphere for 20 h. A white precipitate was collected by suction filtration and solvent was removed from the filtrate under vacuum yielding a clear oil that solidified while refrigerated at 5 °C for a week. Yield: 0.640 g (85%). MP (EtOH) 31.5–33.8 °C; FT-IR (neat): *ν*_max_ 2999 (w), 2952 (m), 2868 (w), 1650 (s), 1588 (m), 1511 (s), 1258 (s), 1137 (s) cm^−1^; ^1^H NMR (400 MHz, in CDCl_3_-d_1_): *δ* 7.33–7.27 (m, 3H), 6.94–6.92 (m, 2H), 6.77 (d, 1H, *J* = 1.8 Hz), 6.71 (d, 1H, *J* = 8.2 Hz), 6.64 (dd, 1H, *J* = 8.2, 1.8 Hz), 4.80 (s, 2H), 3.84 (s, 3H), 3.79 (s, 3H), 2.03 (t, 2H, *J* = 7.5 Hz), 1.58 (quint, 2H, *J* = 7.5 Hz), 1.23–1.13 (m, 4H), 0.81 (t, 3H, *J* = 6.9 Hz) ppm; ^13^C NMR (100 MHz, in CDCl_3_-d_1_): *δ* 173.1, 148.8, 148.3, 142.5, 130.5, 129.5, 128.7, 128.0, 121.5, 112.1, 110.7, 55.9, 52.7, 34.4, 31.5, 25.4, 22.5, 14.0 ppm; HRMS (ESI) calc'd for C_21_H_27_NNaO_5_^+^ [M + Na]^+^ 364.1883, found 364.1873.

##### Synthesis of *N*-(3,4-dimethoxyphenylmethyl)-*N*-(phenyl)-decanamide (22)

General procedure 5 was following using a solution of 16 (0.090 g, 0.37 mmol) in toluene (5 mL), of decanoyl chloride (0.080 g, 0.39 mmol) in toluene (2 mL), and of Et_3_N (0.040 g, 0.42 mmol) in toluene (2 mL). The reaction was allowed to proceed under inert atmosphere for 20 h. A white precipitate was collected by suction filtration and solvent was removed from the filtrate under vacuum yielding a clear oil. Yield: 0.14 g (>95%). FT-IR (neat): *ν*_max_ 2923 (m), 2852 (m), 1652 (m), 1593 (m), 1514 (m), 1259 (s), 1139 (m) cm^−1^; ^1^H NMR (400 MHz, in CDCl_3_-d_1_): *δ* 7.34–7.29 (m, 3H), 6.93 (d, 2H, *J* = 7.9 Hz), 6.76 (s, 1H), 6.71 (d, 1H, *J* = 8.2 Hz), 6.65 (d, 1H, *J* = 8.2 Hz), 4.80 (s, 2H), 3.84 (s, 3H), 3.79 (s, 3H), 2.03 (t, 2H, *J* = 7.5 Hz), 1.57 (t, 2H, *J* = 6.7 Hz), 1.28–1.17 (m, 12H), 0.85 (t, 3H, *J* = 6.9 Hz) ppm; ^13^C NMR (100 MHz, in CDCl_3_-d_1_): *δ* 173.1, 148.8, 148.3, 130.5, 129.6, 128.7, 128.0, 121.5, 112.2, 110.7, 55.9, 52.7, 34.5, 32.0, 29.51, 29.46, 29.3, 25.7, 22.8, 14.2 ppm; HRMS (ESI) calc'd for C_25_H_35_NNaO_5_^+^ [M + Na]^+^ 420.2509, 420.2509.

##### Synthesis of *N*-(3,4-dimethoxyphenylmethyl)-*N*-[4-(4,4,5,5-tetramethyl-1,3,2-dioxaborolan-2-yl)phenyl]-hexanamide (23)

General procedure 5 was following using a solution of 19 (0.260 g, 0.700 mmol) in toluene (10 mL), of hexanoyl chloride (0.090 g, 0.70 mmol) in toluene (4 mL), and of Et_3_N (0.080 g, 0.79 mmol) in toluene (4 mL). The reaction was allowed to proceed under inert atmosphere for 24 h. A white precipitate was collected by suction filtration and solvent was removed from the filtrate under vacuum yielding a colourless solid. Yield: 0.29 g (89%). MP (EtOH) 51.5–53.5 °C; FT-IR (neat): *ν*_max_ 2949 (m), 2859 (w), 1646 (s), 1603 (s), 1511 (m), 1253 (m), 1136 (s) cm^−1^; ^1^H NMR (400 MHz, in CDCl_3_-d_1_): *δ* 7.75 (d, 2H, *J* = 8.0 Hz), 6.94 (d, 2H, *J* = 8.0 Hz), 6.79 (s, 1H), 6.69 (d, 1H, *J* = 8.1 Hz), 6.61 (d, 1H, *J* = 8.1 Hz), 4.80 (s, 2H), 3.83 (s, 3H), 3.79 (s, 3H), 2.03 (t, 2H, *J* = 7.4 Hz), 1.57 (quintet, 2H, *J* = 7.4 Hz), 1.34 (s, 12H), 1.25–1.11 (m, 4H), 0.81 (t, 3H, *J* = 7.0 Hz) ppm; ^13^C NMR (100 MHz, in CDCl_3_-d_1_): *δ* 173.0, 148.9, 148.3, 145.2, 136.0, 130.4, 127.9, 121.5, 112.1, 110.7, 84.2, 55.93, 55.90, 52.7, 34.5, 31.5, 25.4, 25.0, 22.5, 14.0 ppm; ^11^B NMR (128 MHz, in CDCl_3_-d_1_): *δ* 30.4 ppm; HRMS (ESI) calc'd for C_27_H_38_BNNaO_5_^+^ [M + Na]^+^ 490.2735, found 490.2747.

##### Synthesis of *N*-(3,4-dimethoxyphenylmethyl)-*N*-[4-(4,4,5,5-tetramethyl-1,3,2-dioxaborolan-2-yl)phenyl]-decanamide (24)

General procedure 5 was following using a solution of 19 (0.210 g, 0.570 mmol) in toluene (5 mL), of decanoyl chloride (0.110 g, 0.580 mmol) in toluene (2 mL), and of Et_3_N (0.060 g, 0.59 mmol) in toluene (2 mL). The reaction was allowed to proceed under inert atmosphere for 24 h. A white precipitate was collected by suction filtration and solvent was removed from the filtrate under vacuum yielding a colourless solid. Yield: 0.23 g (77%). MP (EtOH) 58.1–59.8 °C; FT-IR (neat): *ν*_max_ 2924 (m), 2853 (w), 1656 (m), 1604 (m), 1514 (m), 1260 (s), 1140 (s) cm^−1^; ^1^H NMR (400 MHz, in CDCl_3_-d_1_): *δ* 7.75 (d, 2H, *J* = 8.1), 6.95 (d, 2H, *J* = 8.1 Hz), 6.79 (d, 1H, *J* = 1.8 Hz), 6.69 (d, 1H, *J* = 8.2 Hz), 6.61 (dd, 1H, *J* = 8.2, 1.8 Hz), 4.80 (s, 2H), 3.84 (s, 3H), 3.80 (s, 3H), 2.03 (t, 2H, *J* = 7.5 Hz), 1.56 (quint, 2H, *J* = 7.0 Hz), 1.34 (s, 12H), 1.26–1.17 (m, 12H), 0.85 (t, 3H, *J* = 7.0 Hz) ppm; ^13^C NMR (100 MHz, in CDCl_3_-d_1_): *δ* 173.0, 148.9, 148.4, 145.2, 136.0, 130.4, 128.0, 121.5, 112.1, 110.7, 84.2, 55.95, 55.92, 52.7, 34.6, 32.0, 29.53, 29.48, 29.4, 25.7, 25.0, 22.8, 14.3 ppm; ^11^B NMR (128 MHz, in CDCl_3_-d_1_): *δ* 32.4 ppm; HRMS (ESI) calc'd for C_31_H_46_BNNaO_5_^+^ [M + Na]^+^ 546.3361, found 546.3368.

##### Synthesis of *N*-(3,4-dimethoxyphenylmethyl)-*N*-[3-(4,4,5,5-tetramethyl-1,3,2-dioxaborolan-2-yl)phenyl]-hexanamide (25)

General procedure 5 was following using a solution of 20 (0.230 g, 0.620 mmol) in toluene (3 mL), of hexanoyl chloride (0.090 g, 0.70 mmol) in toluene (2 mL), and of Et_3_N (0.090 g, 0.89 mmol) in toluene (2 mL). The reaction was allowed to proceed under inert atmosphere for 24 h. A white precipitate was collected by suction filtration and solvent was removed from the filtrate under vacuum yielding a pale yellow solid. Yield: 0.15 g (52%). MP (EtOH) 58.0–60.2 °C; FT-IR (neat): *ν*_max_ 2931 (m), 1652 (m), 1592 (w), 1514 (m), 1260 (s), 1140 (s) cm^−1^; ^1^H NMR (400 MHz, in CDCl_3_-d_1_): *δ* 7.73 (d, 1H, *J* = 7.4 Hz), 7.49 (s, 1H), 7.29 (at, 1H), 6.92 (d, 1H, *J* = 7.9 Hz), 6.76 (s, 1H), 6.71 (d, 1H, *J* = 8.1 Hz), 6.69 (d, 1H, *J* = 8.1 Hz), 4.80 (s, 2H), 3.84 (s, 3H), 3.79 (s, 3H), 2.02 (t, 2H, *J* = 7.5 Hz), 1.61–1.54 (m, 4H), 1.34 (s, 12H), 1.23–1.13 (m, 4H), 0.81 (t, 3H, *J* = 6.9 Hz) ppm; ^13^C NMR (100 MHz, in CDCl_3_-d_1_): *δ* 173.1, 148.8, 148.3, 142.2, 134.5, 134.2, 131.8, 130.5, 128.9, 121.6, 112.2, 110.8, 84.3, 55.9, 52.8, 34.5, 31.6, 25.4, 25.0, 22.6, 14.1 ppm; ^11^B NMR (128 MHz, in CDCl_3_-d_1_): *δ* 30.5 ppm; HRMS (ESI) calc'd for C_27_H_38_BNNaO_5_^+^ [M + Na]^+^ 490.2735, found 490.2750.

##### Synthesis of *N*-(3,4-dimethoxyphenylmethyl)-*N*-[3-(4,4,5,5-tetramethyl-1,3,2-dioxaborolan-2-yl)phenyl]-decanamide (26)

General procedure 5 was following using a solution of 20 (0.300 g, 0.810 mmol) in toluene (10 mL), of decanoyl chloride (0.150 g, 0.790 mmol) in toluene (2 mL), and of Et_3_N (0.090 g, 0.89 mmol) in toluene (2 mL). The reaction was allowed to proceed under inert atmosphere for 24 h. A white precipitate was collected by suction filtration and solvent was removed from the filtrate under vacuum yielding the title compound as a yellow oil. Yield: 0.36 g (85%). FT-IR (neat): *ν*_max_ 2924 (m), 2853 (w), 1655 (m), 1592 (w), 1514 (m), 1261 (s), 1140 (s) cm^−1^; ^1^H NMR (400 MHz, in CDCl_3_-d_1_): *δ* 7.73 (d, 1H, *J* = 7.4 Hz), 7.49 (s, 1H), 7.31–7.27 (m, 1H), 6.92 (ddd, 1H, *J* = 7.9, 2.0, 1.1 Hz), 6.76 (d, 1H, *J* = 2.0 Hz), 6.72–6.65 (m, 2H), 4.80 (s, 2H), 3.84 (s, 3H), 3.79 (s, 3H), 2.01 (t, 2H, *J* = 7.5 Hz), 1.59–1.53 (m, 2H), 1.34 (s, 12H), 1.28–1.17 (m, 12H), 0.85 (t, 3H, *J* = 7.0 Hz) ppm; ^13^C NMR (100 MHz, in CDCl_3_-d_1_): *δ* 173.1, 148.8, 148.3, 142.2, 134.5, 134.2, 131.8, 130.5, 128.9, 121.6, 112.3, 110.7, 84.3, 55.9, 52.8, 34.6, 32.0, 29.5, 29.4, 25.7, 25.0, 22.8, 14.2 ppm; ^11^B NMR (128 MHz, in CDCl_3_-d_1_): *δ* 30.3 ppm; HRMS (ESI) calc'd for C_31_H_46_BNNaO_5_^+^ [M + Na]^+^ 546.3361, found 546.3366.

#### General procedure 6

##### Synthesis of diarylamides from carboxylic acids

A solution of diarylamine (1 equiv.), alkenoic acid (1.2–2 equiv.), 1-ethyl-3-(3-dimethylaminopropyl)carbodiimide HCl (EDCI·HCl, 1.2–2 equiv.), and 4-dimethylaminopyridine (DMAP, 1.5–2 equiv.) in CH_3_CN [0.1–0.2 M] was stirred under an inert atmosphere for 24–48 h before a white precipitate was removed by filtration and the mother liquor was diluted with EtOAc. The resulting solution was then extracted with 2 M HCl, then H_2_O, then brine. The organic layer was dried over MgSO_4_, filtered and concentrated *in vacuo*, before dissolving in CHCl_3_ and filtering through a Dowex plug. The mother liquor was concentrated once more to provide the product as a yellow oil.

##### Synthesis of *N*-(3,4-dimethoxybenzyl)-*N*-phenylpent-4-enamide (27)

General procedure 6 was followed using 16 (0.600 g, 2.47 mmol), 4-pentenoic acid (0.296 g, 2.96 mmol), EDCI·HCl (0.567 g, 2.96 mmol), and DMAP (0.452 g, 3.70 mmol) in CH_3_CN (15 mL) to give the title compound as a yellow oil. Yield: 0.517 g (79%). FT-IR (neat): *ν*_max_ 3070 (w), 2934 (w), 2834 (w), 1651 (s), 1593 (m), 1513 (s), 1259 (s), 1235 (s), 1155 (m), 1138 (s) cm^−1^; ^1^H NMR (400 MHz, in CDCl_3_-d_1_): *δ* 7.34–7.29 (m, 3H), 6.95–6.93 (m, 2H), 6.76 (s, 1H), 6.71 (d, 1H, *J* = 8.1 Hz), 6.64 (d, 1H, *J* = 8.1 Hz), 5.76–5.67 (m, 1H), 4.92 (at, 2H), 4.80 (s, 2H), 3.84 (s, 3H), 3.78 (s, 3H), 2.35 (q, 2H, *J* = 7.0 Hz), 2.13 (t, 2H, *J* = 7.4 Hz) ppm; ^13^C NMR (100 MHz, in CDCl_3_-d_1_): *δ* 172.1, 148.8, 148.3, 142.3, 137.6, 130.3, 129.6, 128.7, 128.1, 121.5, 115.2, 112.1, 110.7, 55.9, 52.8, 33.8, 29.6 ppm; HRMS (ESI) calc'd for C_20_H_23_NNaO_3_^+^ [M + Na]^+^ 348.1570, found 348.1575.

##### Synthesis of *N*-(3,4-dimethoxybenzyl)-*N*-phenylnon-8-enamide (28)

General procedure 6 was followed using 16 (0.600 g, 2.47 mmol), 8-nonenoic acid (0.462 g, 2.96 mmol), EDCI·HCl (0.567 g, 2.96 mmol), and DMAP (0.452 g, 3.70 mmol) in CH_3_CN (15 mL) to give the title compound as a yellow oil. Yield: 0.484 g (61%). FT-IR (neat): *ν*_max_ 3075 (w), 2926 (m), 2853 (w), 1652 (s), 1593 (m), 1513 (m), 1259 (s), 1236 (s), 1156 (m), 1139 (m) cm^−1^; ^1^H NMR (400 MHz, in CDCl_3_-d_1_): *δ* 7.34–7.27 (m, 3H), 6.93 (dd, 2H, *J* = 7.6, 1.7 Hz), 6.76 (d, 1H, 1.4 Hz), 6.71 (d, 1H, *J* = 8.1 Hz), 6.64 (dd, 1H, *J* = 8.1, 1.4 Hz), 5.82–5.70 (m, 1H), 4.97–4.88 (m, 2H), 4.80 (s, 2H), 3.84 (s, 3H), 3.78 (s, 3H), 2.03 (t, 2H, *J* = 7.5 Hz), 1.97 (q, 2H, *J* = 7.1 Hz), 1.57 (quintet, 2H, *J* = 7.1 Hz), 1.31–1.17 (m, 6H) ppm; ^13^C NMR (100 MHz, in CDCl_3_-d_1_): *δ* 173.1, 148.8, 148.3, 142.5, 139.2, 130.4, 129.5, 128.7, 128.0, 121.4, 114.3, 112.1, 110.7, 55.9, 52.7, 34.4, 33.8, 29.2, 28.9, 28.8, 25.6 ppm; HRMS (ESI) calc'd for C_24_H_31_NNaO_3_^+^ [M + Na]^+^ 404.2196, found 404.2191.

##### Synthesis of *N*-(4-butylphenyl)-*N*-(3,4-dimethoxybenzyl)non-8-enamide (29)

General procedure 6 was followed using 18 (0.200 g, 0.668 mmol), 8-nonenoic acid (0.209 g, 1.34 mmol), EDCI·HCl (0.256 g, 1.34 mmol), and DMAP (0.150 g, 1.47 mmol) in CH_3_CN (5 mL) to give the title compound as a yellow oil. Yield: 0.196 g (67%). FT-IR (neat): *ν*_max_ 3075 (w), 2927 (m), 2855 (w), 1652 (s), 1606 (w), 1510 (s), 1259 (s), 1236 (s), 1156 (m), 1139 (m) cm^−1^; ^1^H NMR (400 MHz, in CDCl_3_-d_1_): *δ* 7.11 (d, 2H, *J* = 8.1 Hz), 6.82 (d, 2H, *J* = 8.1 Hz), 6.75–6.72 (m, 2H), 6.68 (d, 2H, *J* = 8.2 Hz), 5.82–5.71 (m, 1H), 4.97–4.89 (m, 2H), 4.78 (s, 2H), 3.85 (s, 3H), 3.79 (s, 3H), 2.60 (t, 2H, *J* = 7.7 Hz), 2.04 (t, 2H *J* = 7.4 Hz), 1.98 (q, 2H, *J* = 7.1 Hz), 1.62–1.55 (m, 5H), 1.39–1.25 (m, 5H), 1.19 (s, 4H), 0.93 (td, 3H, *J* = 7.3, 2.0 Hz) ppm; ^13^C NMR (100 MHz, in CDCl_3_-d_1_): *δ* 173.2, 148.7, 148.2, 142.8, 140.0, 139.2, 130.6, 129.4, 128.3, 121.4, 114.3, 112.2, 110.7, 55.90, 55.86, 52.7, 35.3, 34.4, 33.8, 33.6, 29.2, 28.9, 28.8, 25.6, 22.4, 14.1 ppm; HRMS (ESI) calc'd for C_28_H_39_NNaO_3_^+^ [M + Na]^+^ 460.2822, found 460.2824.

##### Synthesis of *N*-(3,4-dimethoxybenzyl)-*N*-(4-tetradecylphenyl)non-8-enamide (30)

General procedure 6 was followed using 17 (0.200 g, 0.455 mmol), 8-nonenoic acid (0.157 g, 0.910 mmol), EDCI·HCl (0.174 g, 0.910 mmol), and DMAP (0.122 g, 1.00 mmol) in CH_3_CN (5 mL) to give the title compound as a yellow oil. Yield: 0.238 g (93%). FT-IR (neat): *ν*_max_ 3375 (m), 2997–2838 (w), 1635 (w), 1601 (m), 1509 (m), 1238 (m), 1171 (m) cm^−1^; ^1^H NMR (400 MHz, in CDCl_3_-d_1_): *δ* 7.10 (d, 2H *J* = 8.1 Hz), 6.82 (d, 2H, *J* = 8.1 Hz), 6.75 (s, 1H), 6.72 (d, 1H, *J* = 8.2 Hz), 6.67 (d, 1H, *J* = 8.2 Hz), 5.81–5.70 (m, 1H), 4.97–4.92 (m, 1H), 4.97–4.88 (m, 1H), 4.78 (s, 2H), 3.84 (s, 3H), 3.78 (s, 3H), 2.58 (t, 2H, *J* = 7.5 Hz), 2.03 (t, 2H, *J* = 7.5 Hz), 1.97 (q, 2H, *J* = 7.0 Hz), 1.58 (q, 4H, *J* = 7.0 Hz), 1.40–1.18 (m, 30H), 0.89–0.85 (m, 3H) ppm; ^13^C NMR (100 MHz, in CDCl_3_-d_1_): *δ* 173.2, 148.7, 148.2, 142.8, 140.0, 139.1, 130.6, 129.4, 128.3, 121.4, 114.3, 112.2, 110.7, 55.9, 55.8, 52.7, 35.6, 34.3, 33.8, 32.0, 31.4, 29.8, 29.74, 29.69, 29.6, 29.5, 29.4, 29.2, 28.9, 28.8, 25.6, 22.8, 14.2 ppm; HRMS (ESI) calc'd for C_38_H_59_NNaO_3_^+^ [M + Na]^+^ 600.4387, found 600.4377.

#### General procedure 7

##### Hydroboration of diarylalkenamides

A solution of [Ir(COD)Cl]_2_ (5 mol%) and bis(diphenylphosphino)methane (dppm, 10 mol%) in THF under an inert atmosphere for 10 min before adding a solution of diarylalkenamide (1 equiv.) in THF, followed by a solution of HBPin (1.5–2.4 equiv.) in THF. The resulting reaction mixture was stirred for 24 h before removing the solvent *in vacuo*. The crude mixture was dissolved as a concentrated solution in 1 : 1 EtOH/hexanes, and was refrigerated at 5 °C until a white precipitate forms. The precipitate was removed *via* filtration using a celite plug, and the mother liquor was concentrated to provide the crude material as an oil. The crude material was purified *via* short silica plug (eluent – 100% EtOAc), which provided the product as a yellow oil upon concentration of the fractions containing the product.

##### Synthesis of *N*-(3,4-dimethoxybenzyl)-*N*-phenyl-5-(4,4,5,5-tetramethyl-1,3,2-dioxaborolan-2-yl)pentanamide (31)

General procedure 7 was followed using [Ir(COD)Cl]_2_ (0.014 g, 0.021 mmol) and dppm (0.016 g, 0.041 mmol) in THF (2 mL), 27 (0.100 g, 0.411 mmol) in THF (1 mL), and HBPin (0.140 mL, 0.986 mmol) in THF (1 mL) to provide the product as a yellow oil. Yield: 0.073 g (54%). FT-IR (neat): *ν*_max_ 2976–2932 (w), 2250 (w), 1651 (m), 1594 (m), 1371 (m), 1236 (m), 1141 (s) cm^−1^; ^1^H NMR (400 MHz, in CDCl_3_-d_1_): *δ* 7.32–7.27 (m, 3H), 6.93–6.91 (m, 2H), 6.75 (d, 1H, *J* = 1.6 Hz), 6.70 (d, 1H, *J* = 8.2 Hz), 6.63 (dd, 1H, *J* = 8.2, 1.6 Hz), 4.78 (s, 2H), 3.83 (s, 3H), 3.78 (s, 3H), 2.03 (t, 2H, *J* = 7.6 Hz), 1.58 (dt, 2H, *J* = 15.3, 7.8 Hz), 1.28 (t, 2H, *J* = 7.8 Hz), 1.22–1.19 (m, 12H), 0.66 (t, 2H, *J* = 8.0 Hz) ppm; ^13^C NMR (100 MHz, in CDCl_3_-d_1_): *δ* 173.0, 148.8, 148.3, 142.5, 130.4, 129.5, 128.6, 127.9, 121.4, 112.1, 110.7, 83.0, 55.88, 55.86, 52.7, 34.4, 28.3, 24.9, 24.8, 23.9 ppm; ^11^B NMR (100 MHz, in CDCl_3_-d_1_): *δ* 33.5 ppm; HRMS (ESI) calc'd for C_26_H_36_BNNaO_5_^+^ [M + Na]^+^ 476.2579, found 476.2574.

##### Synthesis of *N*-(3,4-dimethoxybenzyl)-*N*-phenyl-9-(4,4,5,5-tetramethyl-1,3,2-dioxaborolan-2-yl)nonamide (32)

General procedure 7 was followed using [Ir(COD)Cl]_2_ (0.011 g, 0.016 mmol) and dppm (0.012 g, 0.031 mmol) in THF (2 mL), 28 (0.100 g, 0.311 mmol) in THF (1 mL), and HBPin (0.068 g, 0.47 mmol) in THF (1 mL) to provide the product as a yellow oil. Yield: 0.089 g (56%). FT-IR (neat): *ν*_max_ 2976–2583 (m), 1652 (m), 1594 (m), 1514 (m), 1371 (m), 1260 (s), 1237 (s), 1141 (s) cm^−1^; ^1^H NMR (400 MHz, in CDCl_3_-d_1_): *δ* 7.34–7.29 (m, 3H), 6.93 (ad, 2H), 6.76 (br s, 1H), 6.71 (d, 1H, *J* = 8.1 Hz), 6.65 (d, 1H, *J* = 8.1), 4.80 (s, 2H), 3.84 (s, 3H), 3.79 (s, 3H), 2.02 (t, 2H, *J* = 7.4 Hz), 1.56 (br s, 2H), 1.38–1.30 (m, 2H), 1.21–1.16 (m, 20H), 0.73 (t, 2H, *J* = 7.8 Hz) ppm; ^13^C NMR (100 MHz, in CDCl_3_-d_1_): *δ* 173.1, 148.8, 148.3, 142.5, 130.4, 129.5, 128.6, 127.9, 121.4, 112.1, 110.7, 82.9, 55.9, 52.7, 34.5, 32.5, 29.4, 29.34, 29.31, 25.7, 24.9, 24.0, 11.6 ppm; ^11^B NMR (100 MHz, in CDCl_3_-d_1_): *δ* 33.5 ppm; HRMS (ESI) calc'd for C_30_H_44_BNNaO_5_^+^ [M + Na]^+^ 532.3205, found 532.3194.

##### Synthesis of *N*-(4-butylphenyl)-*N*-(3,4-dimethoxybenzyl)-9-(4,4,5,5-tetramethyl-1,3,2-dioxaborolan-2-yl)nonamide (33)

General procedure 7 was followed using [Ir(COD)Cl]_2_ (0.008 g, 0.01 mmol) and dppm (0.008 g, 0.02 mmol) in THF (2 mL), 29 (0.100 g, 0.229 mmol) in THF (1 mL), and HBPin (0.080 mL, 0.550 mmol) in THF (1 mL) to provide the product as a yellow oil. Yield: 0.08 g (63%). FT-IR (neat): *ν*_max_ 2925 (m), 2854 (w), 1652 (m), 1607 (w), 1592 (w), 1511 (s), 1371 (m), 1260 (s), 1237 (s), 1141 (s) cm^−1^; ^1^H NMR (400 MHz, in CDCl_3_-d_1_): *δ* 7.10 (d, 2H, *J* = 8.1 Hz), 6.81 (d, 2H, *J* = 8.1 Hz), 6.75 (br s, 1H), 6.72 (d, 1H, *J* = 8.2), 6.67 (d, 1H, *J* = 8.2 Hz), 4.77 (s, 2H), 3.84 (s, 3H), 3.78 (s, 3H), 2.59 (t, 2H, *J* = 7.7 Hz), 2.02 (t, 2H, *J* = 7.5 Hz), 1.62–1.52 (m, 4H), 1.38–1.29 (m, 4H), 1.21–1.16 (m, 20H), 0.92 (td, 3H, *J* = 7.3), 0.73 (t, 2H, *J* = 7.8 Hz) ppm; ^13^C NMR (100 MHz, in CDCl_3_-d_1_): *δ* 173.3, 148.8, 148.2, 142.7, 140.1, 130.7, 129.4, 128.3, 121.4, 112.2, 110.7, 82.9, 55.92, 55.88, 52.7, 35.3, 34.4, 33.6, 32.5, 29.41, 29.36, 25.8, 24.9, 24.1, 22.5, 14.1, 11.3 ppm; ^11^B NMR (100 MHz, in CDCl_3_-d_1_): *δ* 33.3 ppm; HRMS (ESI) calc'd for C_34_H_52_BNNaO_5_^+^ [M + Na]^+^ 588.3831, found 588.3821.

##### Synthesis of *N*-(3,4-dimethoxybenzyl)-*N*-(4-tetradecylphenyl)-9-(4,4,5,5-tetramethyl-1,3,2-dioxaborolan-2-yl)nonamide (34)

General procedure 7 was followed using [Ir(COD)Cl]_2_ (0.006 g, 0.009 mmol) and dppm (0.007 g, 0.02 mmol) in THF (2 mL), 30 (0.100 g, 0.177 mmol) in THF (1 mL), and HBPin (0.034 g, 0.27 mmol) in THF (1 mL) to provide the product as a yellow oil. Yield: 0.06 g (48%). FT-IR (neat): *ν*_max_ 2922 (s), 2852 (s), 1656 (s), 1607 (w), 1592 (w), 1511 (s), 1378 (s), 1260 (s), 1237 (s), 1142 (s) cm^−1^; ^1^H NMR (400 MHz, in CDCl_3_-d_1_): *δ* 7.10 (d, 2H, *J* = 8.1 Hz), 6.82 (d, 2H, *J* = 8.1 Hz), 6.75 (d, 1H, *J* = 1.7 Hz), 6.72 (d, 1H, *J* = 8.2 Hz), 6.67 (dd, 1H, *J* = 8.2, 1.7 Hz), 4.77 (2, s), 3.85 (s, 3H), 3.78 (s, 3H), 2.58 (t, 2H, *J* = 7.8 Hz), 2.02 (t, 2H, *J* = 7.5 Hz), 1.64–1.52 (m, 4H), 1.30–1.16 (m, 44H), 0.87 (t, 3H, *J* = 6.8 Hz), 0.73 (t, 2H, *J* = 7.8 Hz) ppm; ^13^C NMR (100 MHz, in CDCl_3_-d_1_): *δ* 173.3, 148.7, 148.2, 142.8, 140.1, 130.6, 129.4, 128.3, 121.4, 112.2, 110.7, 82.9, 55.9, 55.8, 52.7, 35.6, 34.4, 32.5, 32.0, 31.4, 29.79, 29.76, 29.7, 29.6, 29.5, 29.43, 29.39, 29.3, 25.7, 24.9, 24.1, 22.8, 14.2, 11.3 ppm; ^11^B NMR (100 MHz, in CDCl_3_-d_1_): *δ* 33.9 ppm; HRMS (ESI) calc'd for C_44_H_72_BNNaO_5_^+^ [M + Na]^+^ 728.5396, found 728.5369.

### Antimicrobial assay

The microorganisms (methicillin-resistant *S. aureus* MRSA 310, vancomycin-resistant *E. faecium* VRE 379, *S. warneri* (ATCC 17917), *P. vulgaris* (ATCC 12454), *P. aeruginosa* (ATCC 14210), and *C. albicans* (ATCC 14035)) were grown overnight on agar media. The following day the cultures were transferred into 0.9% saline water into a sterile glass tube. Using the McFarland reader, the turbidity was determined and further diluted into their respective media. The pure compounds were tested at seven different concentrations ranging from 2 to 128 μg mL^−1^ in triplicate. Samples were plated into 96 well plates (10 μL in 20% DMSO) and then diluted pathogen (90 μL) was pipetted into the pre-prepared sample plates yielding 5 × 10^5^ cfu mL^−1^ with a final volume of 100 μL. Antibiotics that are active against specific pathogens were used as positive controls. The plates were incubated into a 37 °C incubator for 22 h. Growth of *S. warneri*. *P. vulgaris*, *P. aeruginosa* and *C. albicans* were measured by reading optical density (OD_600_), or in the case of slow growing microorganisms (MRSA and VRE), PrestoBlue was used to assess metabolic activity by measuring fluorescence (535–560)/(590–615) (excitation/emission) using a Thermo Scientific Varioskan Flash plate reader at time zero and then again after incubation of the plates for 22 h at 37 °C. After subtracting the time zero OD_600_ from the final reading, the percentages of microorganism survival relative to vehicle control wells were calculated. Comparable to literature.^49^

### Cytotoxicity assay

The cells (human foreskin BJ fibroblast cells (ATCC CRL-2522), green monkey kidney cells Vero (ATCC CCL-81), human breast adenocarcinoma cells (ATCC HTB-26 MDA-MB 231), human breast adenocarcinoma cells (MCF7 ATCC HTB-22), and human colorectal carcinoma cells (HCT116 ATCC CCL-247)) were grown to 80% confluency, the cells were counted, diluted, and plated into treated 96-well cell culture plates. The BJ fibroblast and Vero cells were plated at a cell density of 10 000 cells per well and the HTB26, HCT116 and MCF7 cells were plated at cell density of 5000 cells per well in 90 μL of respective growth medium (without the addition of antibiotics) and incubated for 24 h to allow cells to adhere to the plates before treatment. Pure compounds were tested in triplicate in serial dilutions of seven concentrations ranging from 2 to 128 μg mL^−1^ per well (final well volume of 100 μL, 1% DMSO per well). Each of the cell lines were incubated at 37 °C in a humidified atmosphere of 5% CO_2_; the BJ fibroblast and Vero cells for 24 h and the HTB-26, HCT116 and MCF7 cells for 72 h. Each plate contained four uninoculated positive controls, four untreated negative controls, and one column containing a concentration range for doxorubicin. Alamar blue was added, 24 h after the treatment, and fluorescence was monitored using a Cytation Gen 5 plate reader using 560 nm Ex/590 Em both at time zero and 4 h after Alamar blue addition. The inferred percentage of cell viability relative to vehicle control wells were calculated after subtracting the time zero emission 590 nm measurement from the final reading and the IC_50_ was determined. Comparable to literature.^49^

## Conflicts of interest

There are no conflicts of interest to declare.

## Supplementary Material

RA-011-D1RA04943G-s001

## References

[cit1] Kumar S. K., LaPlant B., Roy V., Reeder C. B., Lacy M. Q., Gertz M. A., Laumann K., Thompson M. A., Witzig T. E., Buadi F. K., Rivera C. E., Mikhael J. R., Bergsagel P. L., Kapoor P., Hwa L., Fonseca R., Stewart A. K., Chanan-Khan A., Rajkumar S. V., Dispenzieri A. (2015). Blood Canc. J..

[cit2] Touchet S., Carreaux F., Carboni B., Bouillon A., Boucher J.-L. (2011). Chem. Soc. Rev..

[cit3] Paller A. S., Tom W. L., Lebwohl M. G., Blumenthal R. L., Boguniewicz M., Call R. S., Eichenfield L. F., Forsha D. W., Rees W. C., Simpson E. L., Spellman M. C., Stein Gold L. F., Zaenglein A. L., Hughes M. H., Zane L. T., Hebert A. A. (2016). J. Am. Acad. Dermatol..

[cit4] Muz B., Ghazarian R. N., Ou M., Luderer M. J., Kusdono H. D., Azab A. K. (2016). Drug Des., Dev. Ther..

[cit5] Kupperman E., Lee E. C., Cao Y., Bannerman B., Fitzgerald M., Berger A., Yu J., Yang Y., Hales P., Bruzzese F., Liu J., Blank J., Garcia K., Tsu C., Dick L., Fleming P., Yu L., Manfredi M., Rolfe M., Bolen J. (2010). Cancer Res..

[cit6] Chauhan D., Tian Z., Zhou B., Kuhn D., Orlowski R., Raje N., Richardson P., Anderson K. C. (2011). Clin. Canc. Res..

[cit7] Baker S. J., Zhang Y.-K., Akama T., Lau A., Zhou H., Hernandez V., Mao W., Alley M. R. K., Sanders V., Plattner J. J. (2006). J. Med. Chem..

[cit8] Sharma N., Sharma D. (2015). J. Pharmacol. Pharmacother..

[cit9] Castanheira M., Rhomberg P. R., Flamm R. K., Jones R. N. (2016). Antimicrob. Agents Chemother..

[cit10] Lomovskaya O., Sun D., Rubio-Aparicio D., Nelson K., Tsivkovski R., Griffith D. C., Dudley M. N. (2017). Antimicrob. Agents Chemother..

[cit11] Griffith D. C., Loutit J. S., Morgan E. E., Durso S., Dudley M. N. (2016). Antimicrob. Agents Chemother..

[cit12] Ali F., Hosmane N. S., Zhu Y. (2020). Molecules.

[cit13] Tevyashova A. N., Chudihov M. V. (2021). Russ. Chem. Rev..

[cit14] He X.-F., Zhang H., Vogels C. M., Decken A., Westcott S. A. (2004). Heteroat. Chem..

[cit15] Westcott S. A., He X.-F., Vogels C. M., Decken A. (2004). Acta Crystallogr., Sect. E: Struct. Rep. Online.

[cit16] Decken A., Singh A., Vogels C. M., Westcott S. A. (2002). Acta Crystallogr., Sect. E: Struct. Rep. Online.

[cit17] Zhang H., Vogels C. M., Wheaton S. L., Baerlocher F. J., Decken A., Westcott S. A. (2005). Synthesis.

[cit18] Hicks J. W., Kyle C. B., Vogels C. M., Wheaton S. L., Baerlocher F. J., Decken A., Westcott S. A. (2008). Chem. Biodiversity.

[cit19] Duguay D. R., Zamora M. T., Blacquiere J. M., Appoh F. E., Vogels C. M., Wheaton S. L., Baerlocher F. J., Decken A., Westcott S. A. (2008). Cent. Eur. J. Chem..

[cit20] Appoh F. E., Wheaton S. L., Vogels C. M., Baerlocher F. J., Decken A., Westcott S. A. (2009). Heteroat. Chem..

[cit21] Gwynne E. A., Holt J. C., Dwan J. R., Appoh F. E., Vogels C. M., Wheaton S. L., Baerlocher F. J., Decken A., Westcott S. A. (2010). Helv. Chim. Acta.

[cit22] Vogels C. M., Nikolcheva L. G., Norman D. W., Spinney H. A., Decken A., Baerlocher M. O., Baerlocher F. J., Westcott S. A. (2011). Can. J. Chem..

[cit23] Mosseler J. A., Melanson J. A., Bowes E. G., Lee G. M., Vogels C. M., Baerlocher F. J., Decken A., Westcott S. A. (2011). J. Mol. Struct..

[cit24] Geier M. J., Bowes E. G., Lee G. M., Li H., O'Neill T., Flewelling A., Vogels C., Decken A., Gray C. A., Westcott S. A. (2013). Heteroat. Chem..

[cit25] Webb M. I., Halcovitch N. R., Bowes E. G., Lee G. M., Geier M. J., Vogels C. M., O'Neill T., Li H., Flewelling A. J., Decken A., Gray C. A., Westcott S. A. (2014). J. Heterocycl. Chem..

[cit26] Campbell-Verduyn L., Bowes E. G., Li H., Vallée A. M., Vogels C. M., Decken A., Gray C. A., Westcott S. A. (2014). Heteroat. Chem..

[cit27] Yang J., Johnson B. J., Letourneau A. A., Vogels C. M., Decken A., Baerlocher F. J., Westcott S. A. (2015). Aust. J. Chem..

[cit28] Hébert M. J. G., Flewelling A. J., Clark T. N., Levesque N. A., Jean-François J., Surette M. E., Gray C. A., Vogels C. M., Touaibia M., Westcott S. A. (2015). Int. J. Med. Chem..

[cit29] Cormier K., Curry R. D., Betsch M. P., Goguen J. A., Vogels C. M., Decken A., Turcotte S., Westcott S. A. (2016). J. Heterocycl. Chem..

[cit30] St-Coeur P.-D., Kinley S., Vogels C. M., Decken A., Morin Jr. P., Westcott S. A. (2016). Can. J. Chem..

[cit31] Zhu D., Hunter C. D., Baird S. R., Davis B. R., Bos A., Geier S. J., Vogels C. M., Decken A., Gray C. A., Westcott S. A. (2017). Heteroat. Chem..

[cit32] Scott R. S., Veinot A. J., Stack D. L., Gormley P. T., Ninh Khuong B., Vogels C. M., Masuda J. D., Baerlocher F. J., MacCormack T. J., Westcott S. A. (2018). Can. J. Chem..

[cit33] Gormley P. T., Geier S. J., Vogels C. M., Ninh Khuong B., MacCormack T. J., Masuda J., Westcott S. A. (2020). J. Braz. Chem. Soc..

[cit34] Irving A. M., Vogels C. M., Nikolcheva L. G., Edwards J. P., He X.-F., Hamilton M. G., Baerlocher M. O., Baerlocher F. J., Decken A., Westcott S. A. (2003). New J. Chem..

[cit35] Patterson A. E., Flewelling A. J., Clark T. N., Geier S. J., Vogels C. M., Masuda J. D., Gray C. A., Westcott S. A. (2015). Can. J. Chem..

[cit36] Ramsaywack S., Bos A., Vogels C. M., Gray C. A., Westcott S. A. (2018). Can. J. Chem..

[cit37] Bennett D. J., Kirby G. W. (1968). J. Chem. Soc. C.

[cit38] Adaszek L., Gadomska D., Mazurek L., Lyp P., Madany J., Winiarczyk S. (2019). Res. Vet. Sci..

[cit39] Aquilina G., Bories G., Chesson A., Cocconcelli P. S., de Knecht J., Dierick N. A., Gralak M. A., Gropp J., Halle I., Hogstrand C., Kroker R., Leng L., López Puente S., Lundebye Haldorsen A.-K., Mantovani A., Martelli G., Mézes M., Renshaw D., Saarela M., Sejrsen K., Westendorf J. (2012). EFSA J..

[cit40] Cocco G., Strozzi C., Chu D. (1979). Clin. Cardiol..

[cit41] Ramuz H. (1978). Arzneim.-Forsch..

[cit42] Khurmi N. S., Robinson C. W., O'Hara M. J., Raftery E. B. (1986). Eur. J. Clin. Pharmacol..

[cit43] Rubbo S. D., Cymerman-Craig J. (1955). Nature.

[cit44] Sah P. P. T., Peoples S. A. (1954). J. Am. Pharm. Assoc..

[cit45] Tejel C., Bravi R., Ciriano M. A., Oro L. A., Bordonaba M., Graiff C., Tiripicchio A., Burini A. (2000). Organometallics.

[cit46] Fernández E. and Segarra A. M., in Iridium Complexes in Organic Synthesis*,* ed. L. A. Oro and C. Claver, John Wiley & Sons, Inc., 2008. ch. 7, pp. 173–194

[cit47] Wang L., Cao C., Cao C. (2019). J. Phys. Org. Chem..

[cit48] Ghiano D. G., Carvalho P. B., Tekwani B. L., Avery M. A., Labadie G. (2011). ARKIVOC.

[cit49] Overy D. P., Berrue F., Correa H., Hanif N., Hay K., Lanteigne M., McQuillan K., Duffy S., Boland P., Jagannathan R., Carr G. S., Vansteeland M., Kerr R. G. (2015). Mycology.

