# Supplementary material for: Boron-containing capsaicinoids
Source: RSC Adv. 2021 Jul 23;11(39):24282–91. doi: 10.1039/d1ra04943g (PMC9036659; doi:10.1039/d1ra04943g)
Supplement: RA-011-D1RA04943G-s001 [file RA-011-D1RA04943G-s001.pdf]

Electronic Supplementary Material (ESI) for RSC Advances.
This journal is © The Royal Society of Chemistry 2021

Electronic Supplementary Material (ESI) for RSC Advances. This journal is © The Royal Society of Chemistry 2020

Supporting Information

Boron-Containing Capsaicinoids

:

Jennifer A. Melanson,[a] Maxim F. Landry,[a] Martin Lanteigne[b], Katherine McQuillan,[b] Hebelin Correa,[b] Russell Kerr,*[c] and Stephen A. Westcott*[a]

[a] *Department of Chemistry and Biochemistry, Mount Allison University, Sackville, NB E4L 1G8, Canada. *E-mail: swestcott@mta.ca*
[b] *Nautilus Biosciences CRODA Canada Inc., Duffy Research Centre, Charlottetown, PE C1A4P3, Canada.*
[c] *Department of Chemistry, University of Prince Edward Island, Charlottetown, PE C1A4P3, Canada. *Email: rkerr@upei.ca*

Table of Contents

General information………S2

NMR, IR, and HRMS Spectra of the Products……………………………………………………………………………S3-S98

Antimicrobial and Cytotoxicity Data………………………………………………………………………………………S99-S106

General Information

Chemistry Data NMR spectra were recorded on a JEOL JNM-GSX400 FT NMR (^{1}H: 400 MHz; ^{11}B: 128 MHz; ^{13}C: 100 MHz) spectrometer. ^{1}H, $^{13}C\{^{1}H\}$, and $^{11}B\{^{1}H\}$ NMR chemical shifts (δ/ppm) are referenced to Me_4Si, Me_4Si, and $BF_3{\cdot}OEt_2$, respectively. Chemical shifts (δ) are reported in ppm [relative to residual solvent peaks (^{1}H and ^{13}C) or external $BF_3{\cdot}OEt_2$ (^{11}B). Multiplicities are reported as singlet (s), doublet (d), triplet (t), quartet (q), quintet (quint), multiplet (m), and broad (br) with coupling constants (*J*) reported in hertz. High resolution mass spectrometry was performed at DalChem Mass Spec Laboratory (Dalhousie University, Halifax, NS).

Antimicrobial Assay Data Growth of *S. warneri*. *P. vulgaris*, *P. aeruginosa* and *C. albicans* were measured by reading optical density (OD600), or in the case of slow growing microorganisms (MRSA and VRE), PrestoBlue was used to assess metabolic activity by measuring fluorescence (535-560)⁄(590-615) (excitation/emission) using a Thermo Scientific Varioskan Flash plate reader at time zero and then again after incubation of the plates for 22 h at 37ºC. After subtracting the time zero OD600 from the final reading, the percentages of microorganism survival relative to vehicle control wells were calculated.

Cytotoxicity Assay Data Alamar blue was added, 24 h after the treatment, and fluorescence was monitored using a Cytation Gen 5 plate reader using 560 nm Ex/590 Em both at time zero and 4 h after Alamar blue addition. The inferred percentage of cell viability relative to vehicle control wells were calculated after subtracting the time zero emission 590 nm measurement from the final reading and the IC50 was determined.

NMR, IR, and HRMS Spectra of the Products

1-(3,4-dimethoxyphenyl)-*N*-(4-tetradecylphenyl)methanimine(**13**)–^{1}H NMR

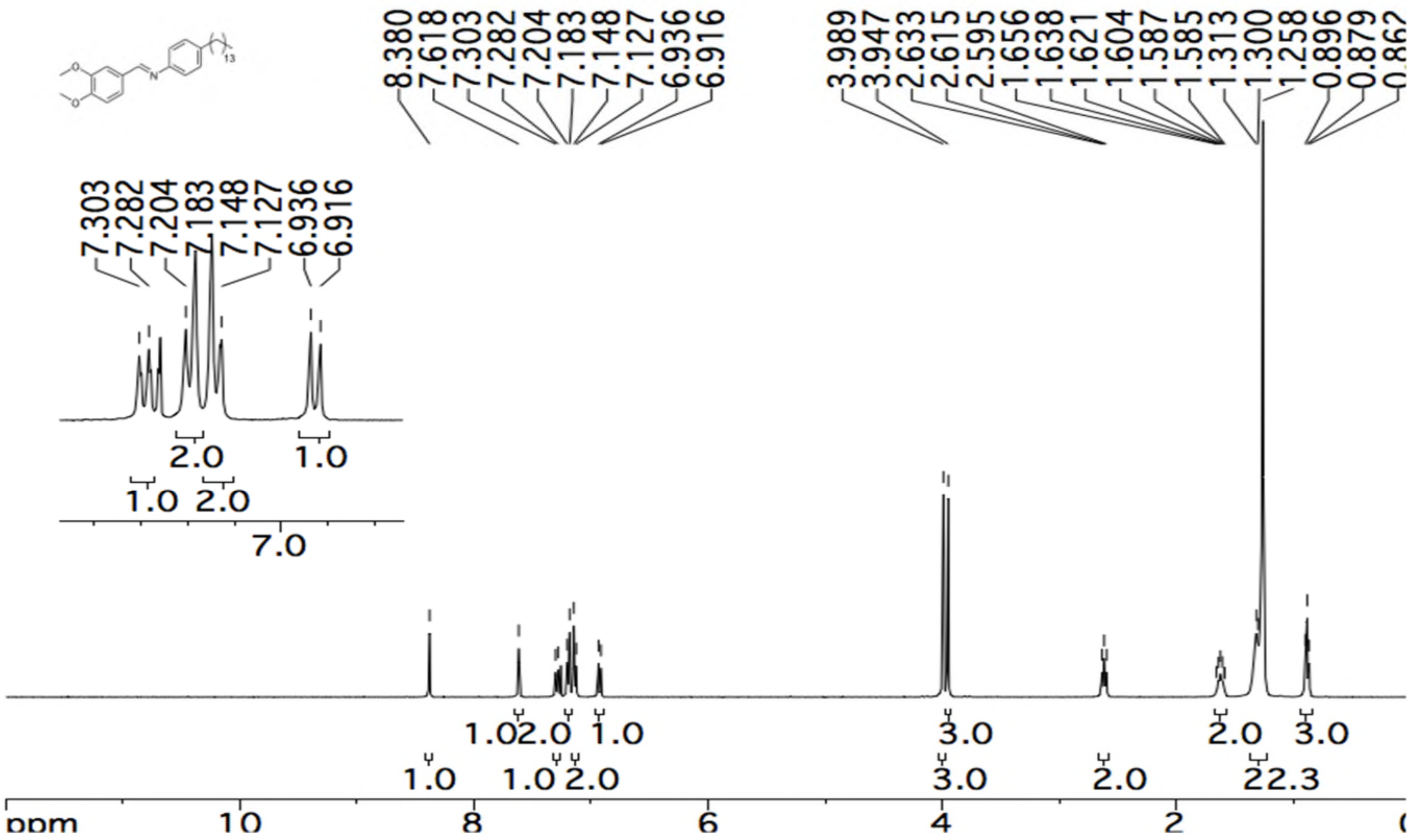

1-(3,4-dimethoxyphenyl)-*N*-(4-tetradecylphenyl)methanimine(**13**)–^{13}C NMR

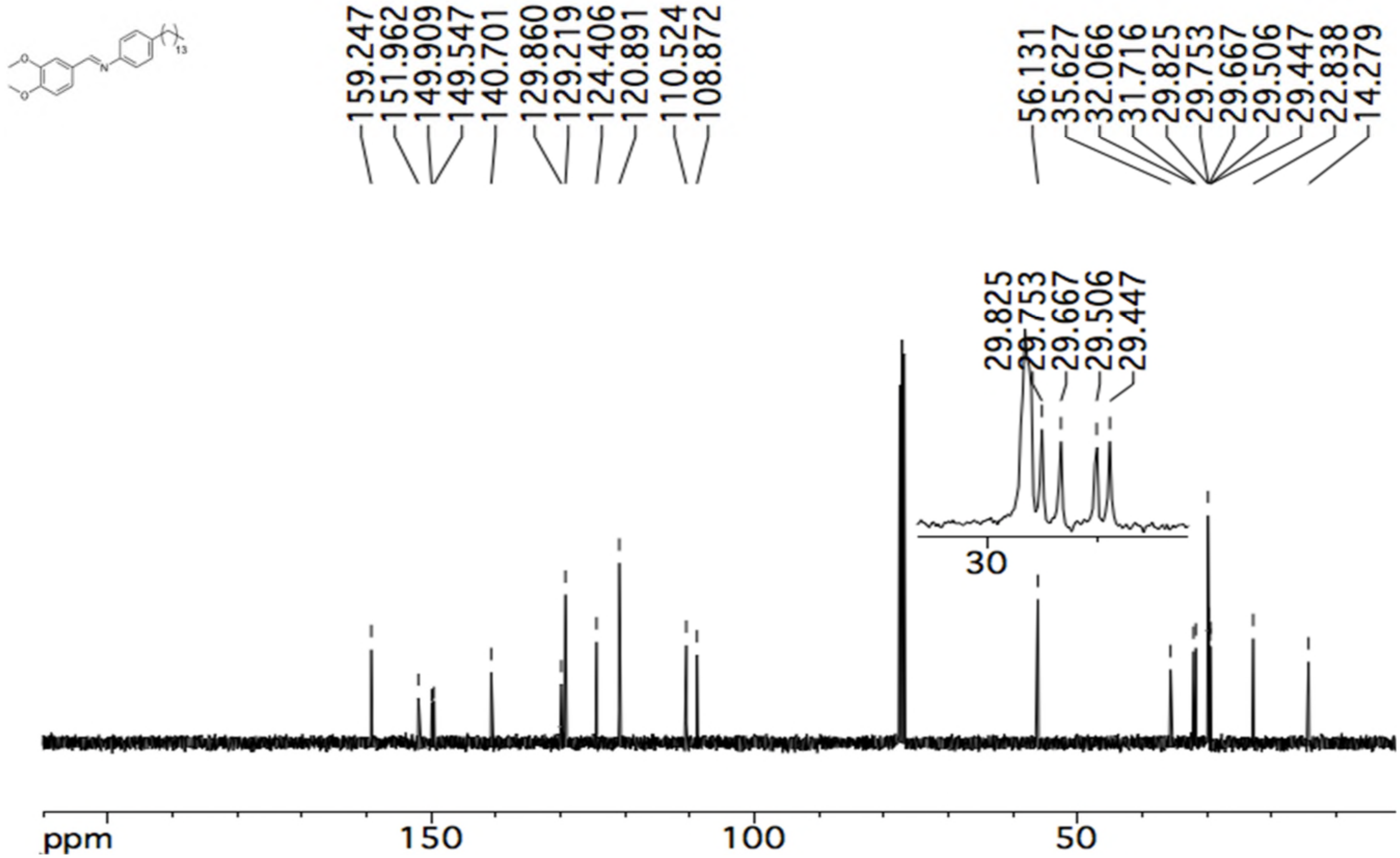

1-(3,4-dimethoxyphenyl)-*N*-(4-tetradecylphenyl)methanimine (**13**) – IR

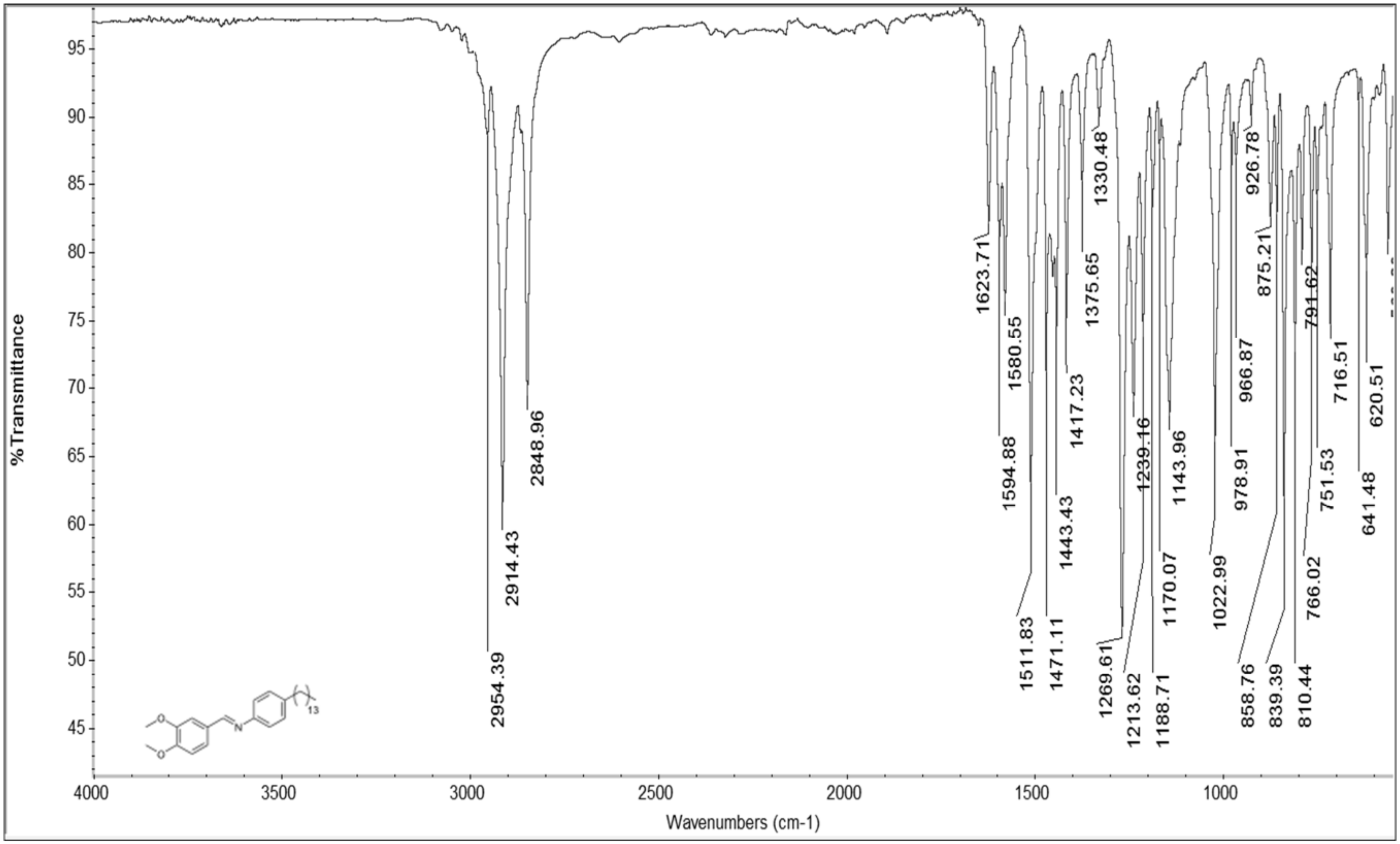

1-(3,4-dimethoxyphenyl)-*N*-(4-tetradecylphenyl)methanimine(**13**)- HRMS

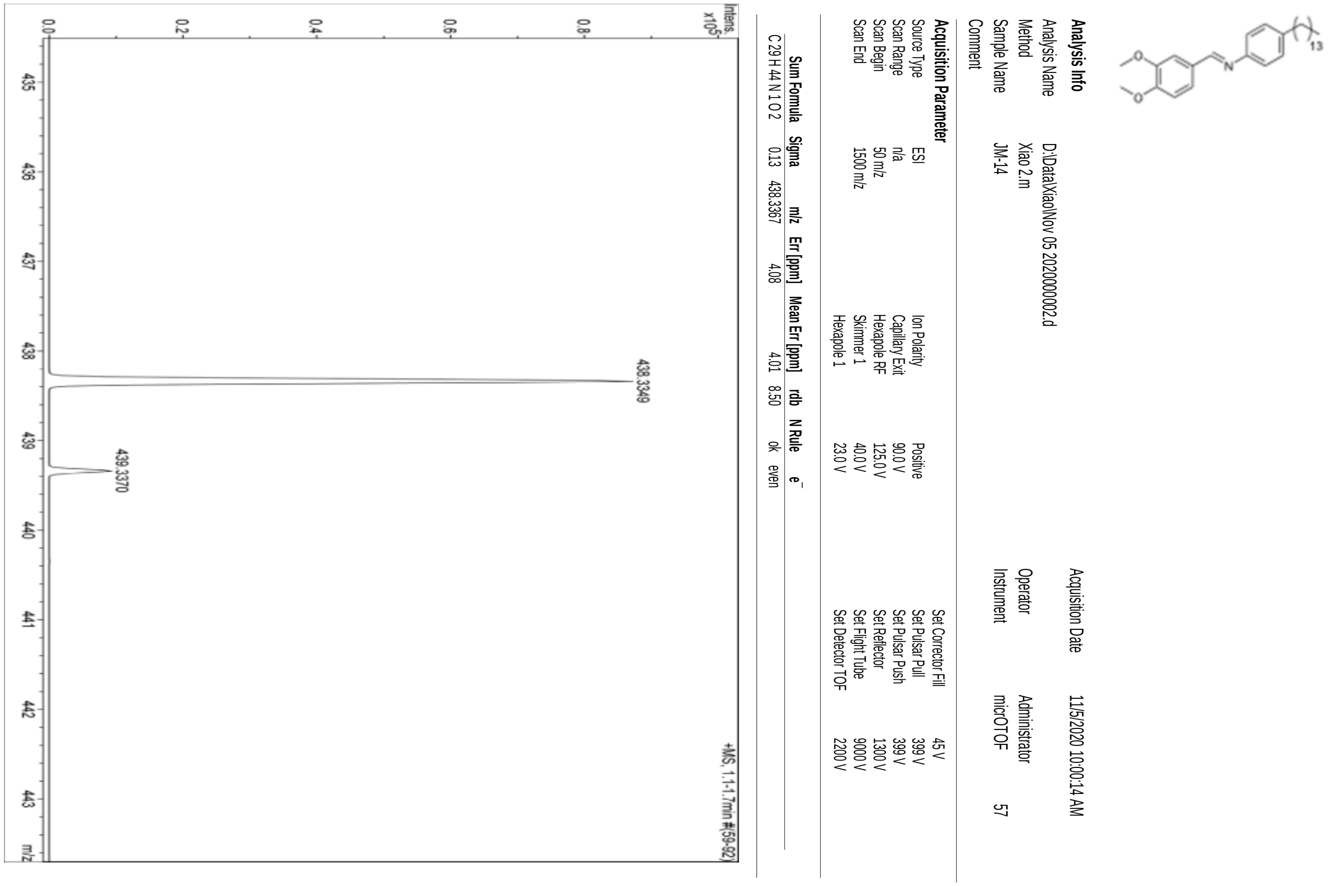

1-(3,4-dimethoxyphenyl)-*N*-(4-(4,4,5,5-tetramethyl-1,3,2-dioxaborolan-2-yl)phenyl)methanimine (**14**) – ^{1}H NMR

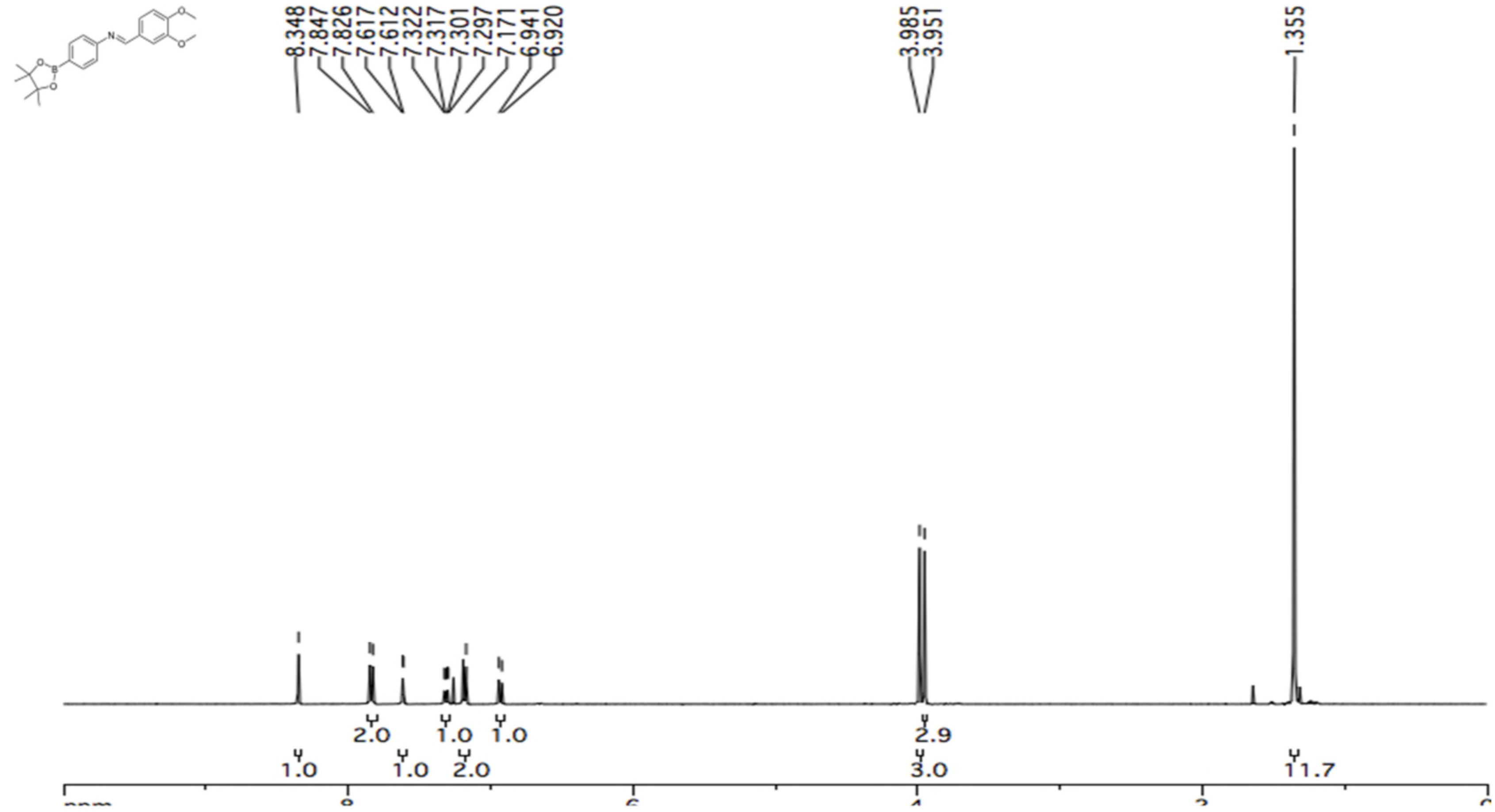

1-(3,4-dimethoxyphenyl)-*N*-(4-(4,4,5,5-tetramethyl-1,3,2-dioxaborolan-2-yl)phenyl)methanimine (**14**) – ^{13}C NMR

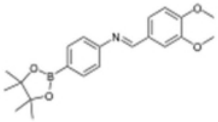

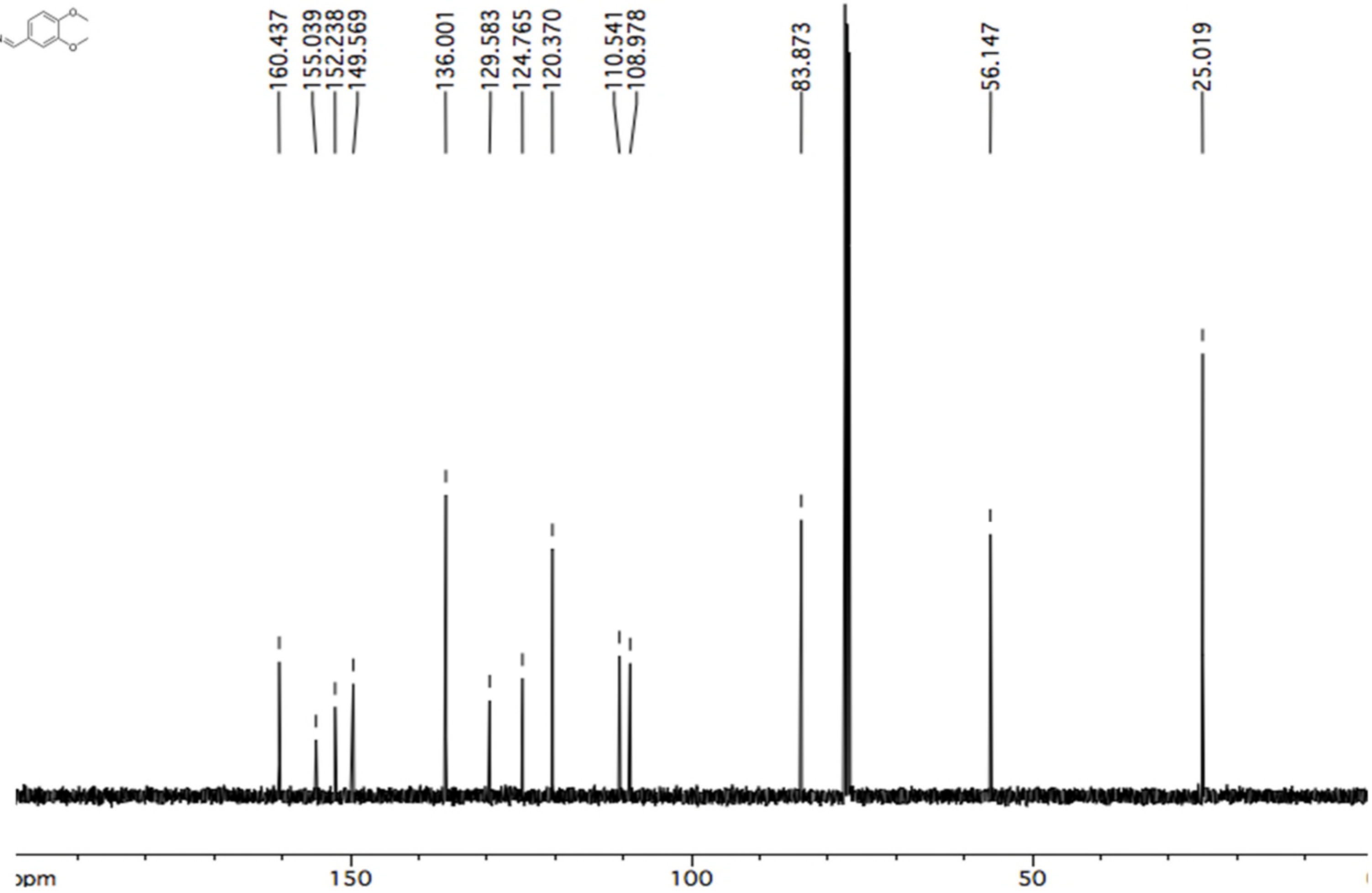

1-(3,4-dimethoxyphenyl)-*N*-(4-(4,4,5,5-tetramethyl-1,3,2-dioxaborolan-2-yl)phenyl)methanimine (**14**) – ^{11}B NMR

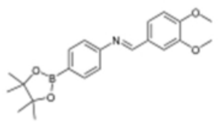

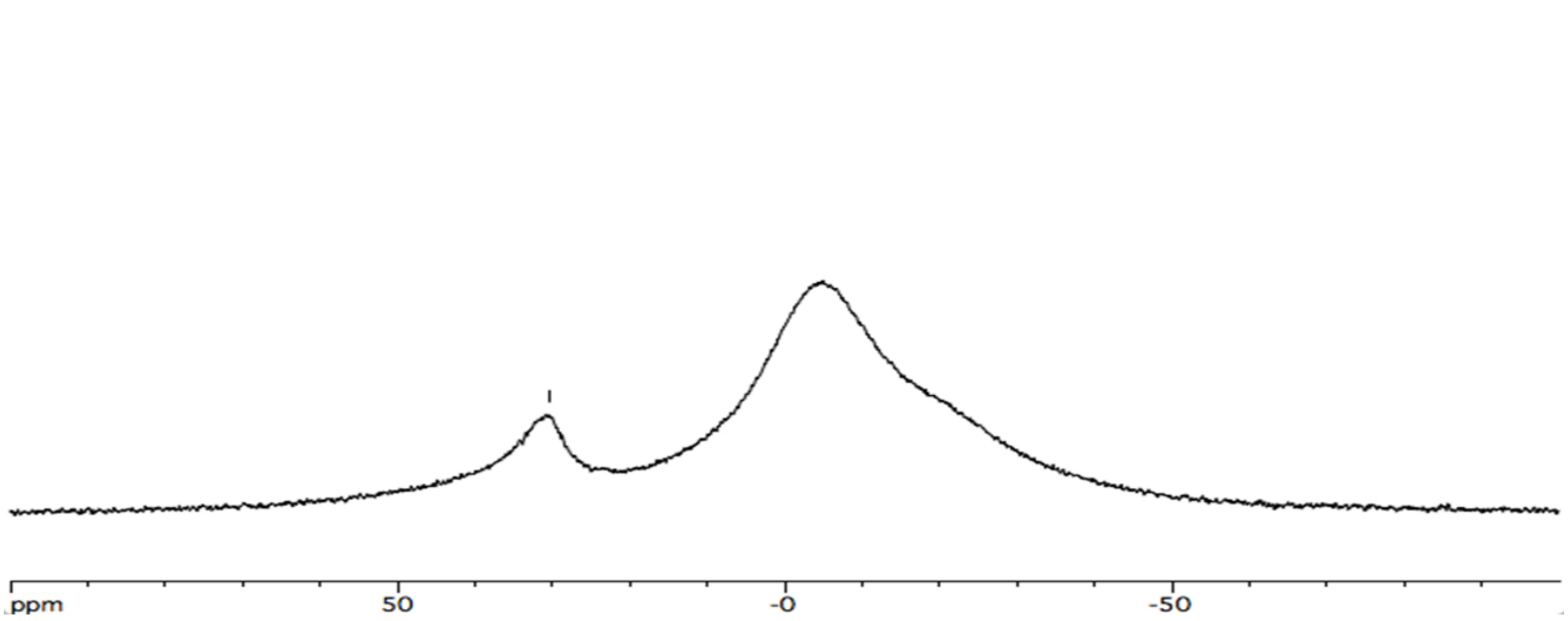

1-(3,4-dimethoxyphenyl)-*N*-(4-(4,4,5,5-tetramethyl-1,3,2-dioxaborolan-2-yl)phenyl)methanimine (**14**) – IR

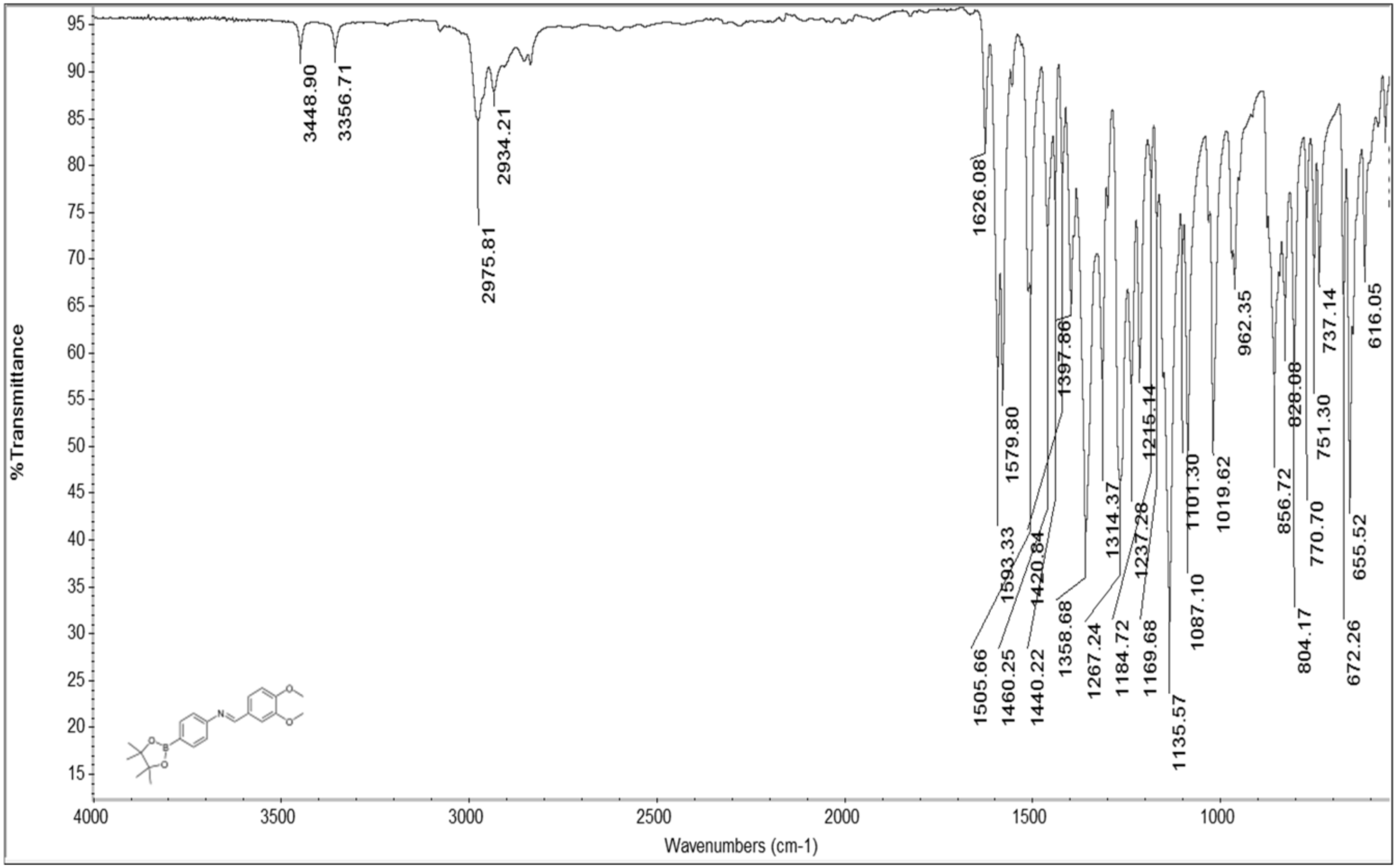

1-(3,4-dimethoxyphenyl)-*N*-(4-(4,4,5,5-tetramethyl-1,3,2-dioxaborolan-2-yl)phenyl)methanimine (**14**) – HRMS

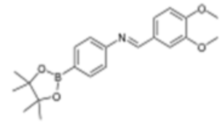

Analysis Info

Analysis Name D:\Data\Xiao\Nov 05 2020000003.d
Method Xiao 2.m
Sample Name JM-49
Comment

Acquisition Date 11/5/2020 10:11:31 AM
Operator Administrator
Instrument micrOTOF 57

Acquisition Parameter

Source Type	ESI	Ion Polarity	Positive	Set Corrector Fill	45 V
Scan Range	n/a	Capillary Exit	90.0 V	Set Pulsar Pull	399 V
Scan Begin	50 m/z	Hexapole RF	125.0 V	Set Pulsar Push	399 V
Scan End	1500 m/z	Skimmer 1	40.0 V	Set Reflector	1300 V
		Hexapole 1	23.0 V	Set Flight Tube	9000 V
				Set Detector TOF	2200 V

Sum Formula	Sigma	m/z	Err [ppm]	Mean Err [ppm]	rdb	N Rule	e⁻
C 21 H 27 B 1 N 1 O 4	0.12	368.2028	3.84	3.98	9.50	ok	even

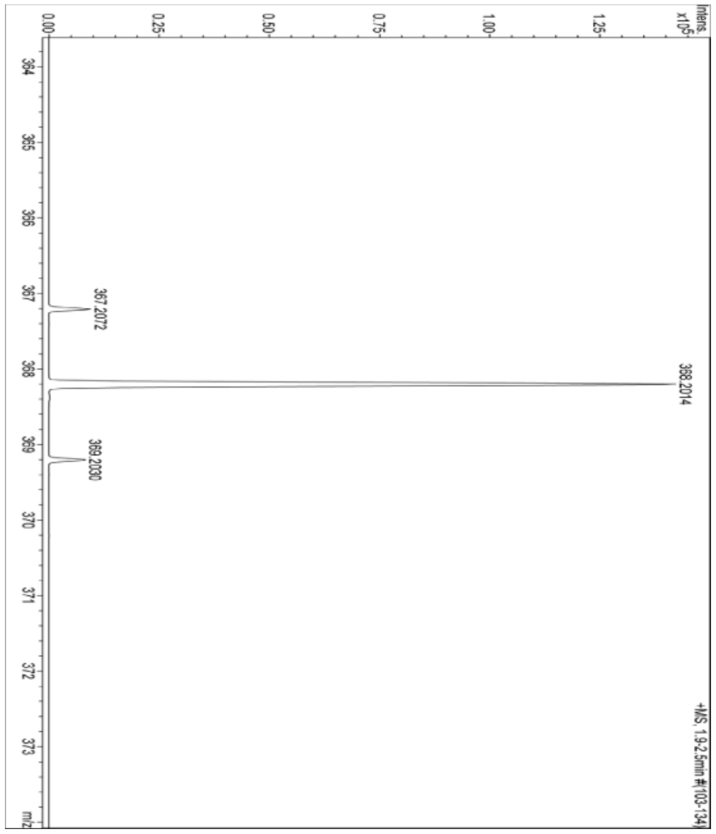

1-(3,4-dimethoxyphenyl)-*N*-(3-(4,4,5,5-tetramethyl-1,3,2-dioxaborolan-2-yl)phenyl)methanimine (**15**) – ^{1}H NMR

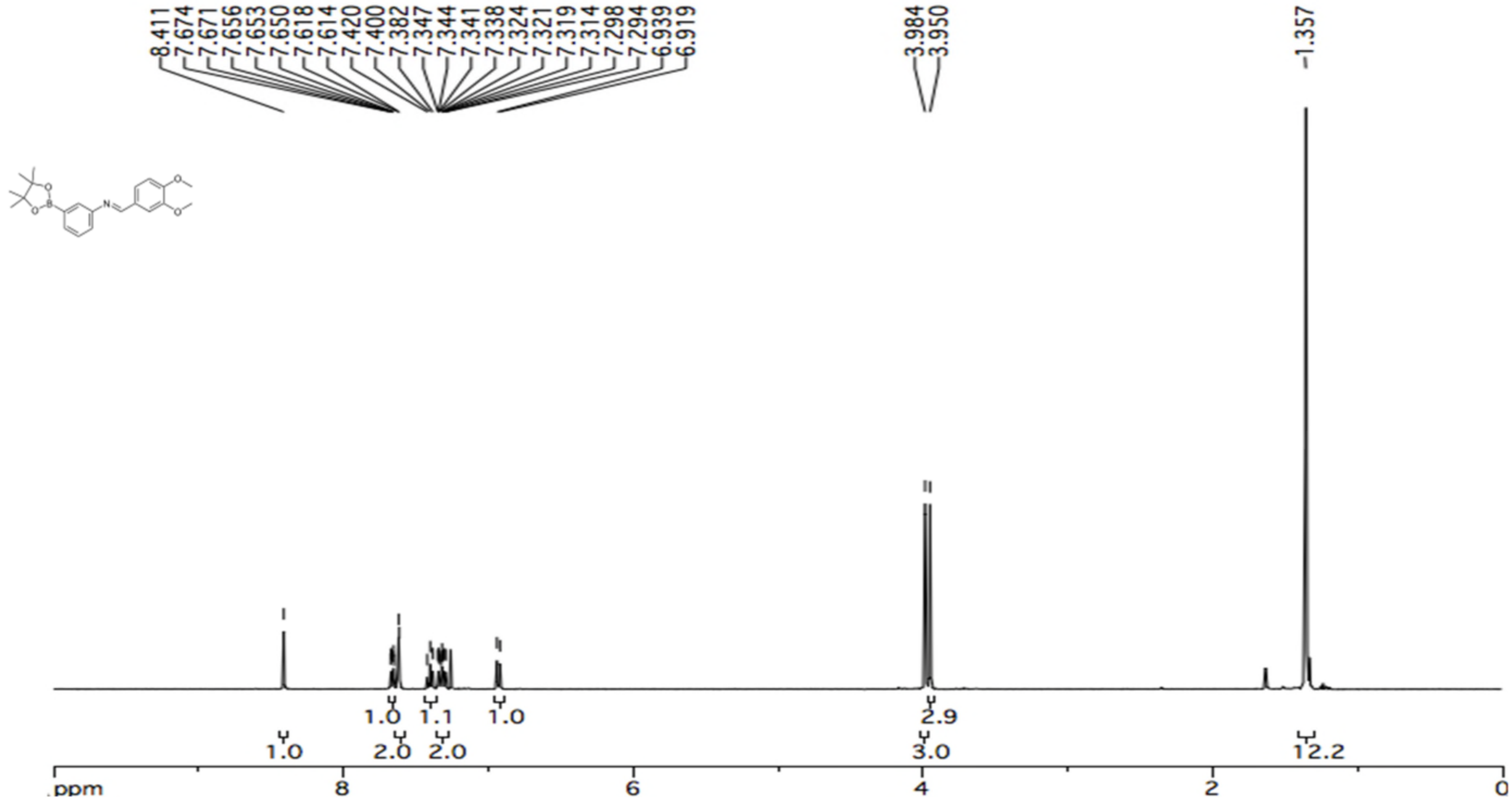

1-(3,4-dimethoxyphenyl)-*N*-(3-(4,4,5,5-tetramethyl-1,3,2-dioxaborolan-2-yl)phenyl)methanimine (**15**) – ^{13}C NMR

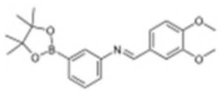

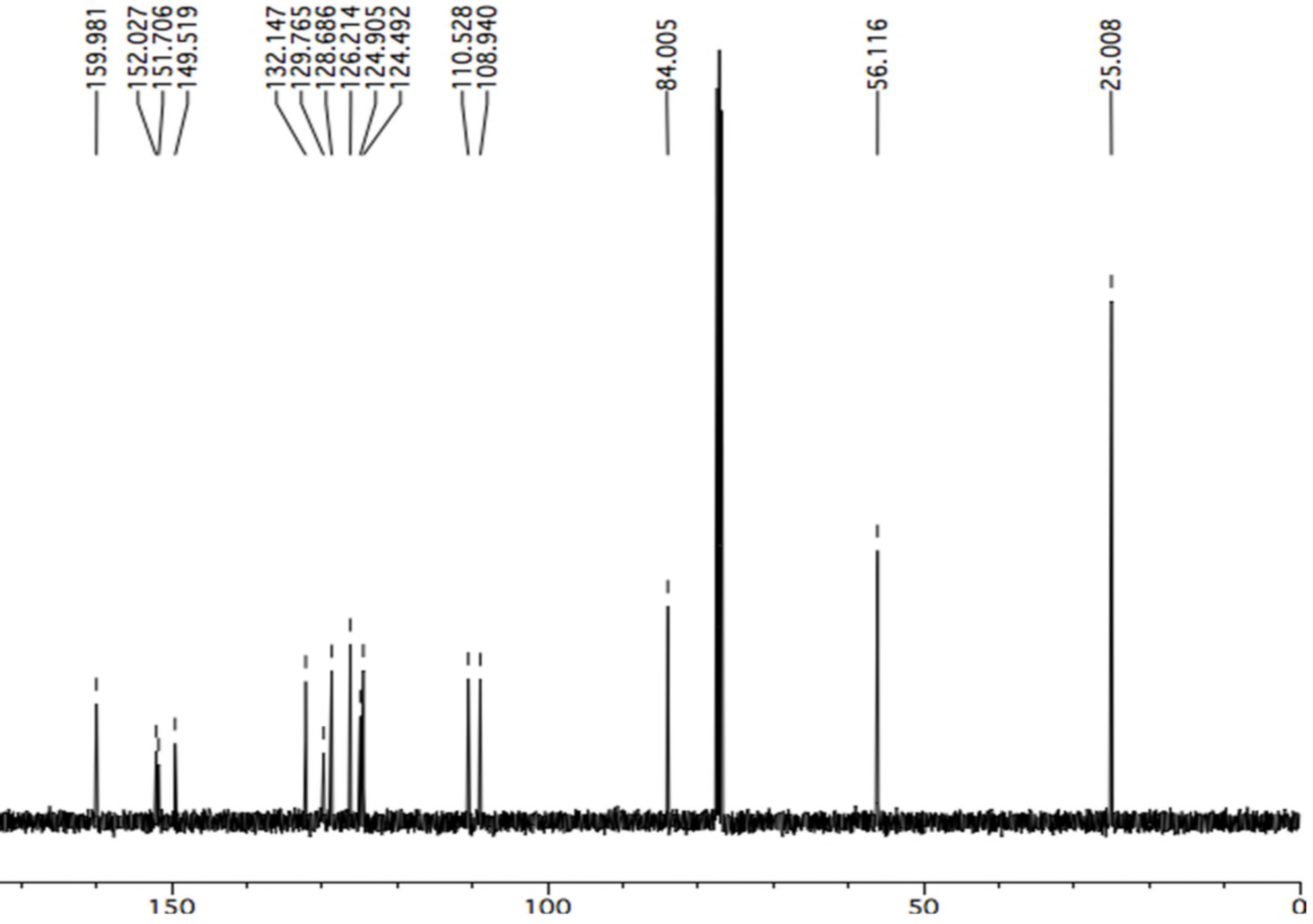

1-(3,4-dimethoxyphenyl)-*N*-(3-(4,4,5,5-tetramethyl-1,3,2-dioxaborolan-2-yl)phenyl)methanimine (**15**) – ^{11}B NMR

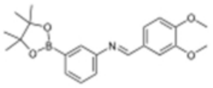

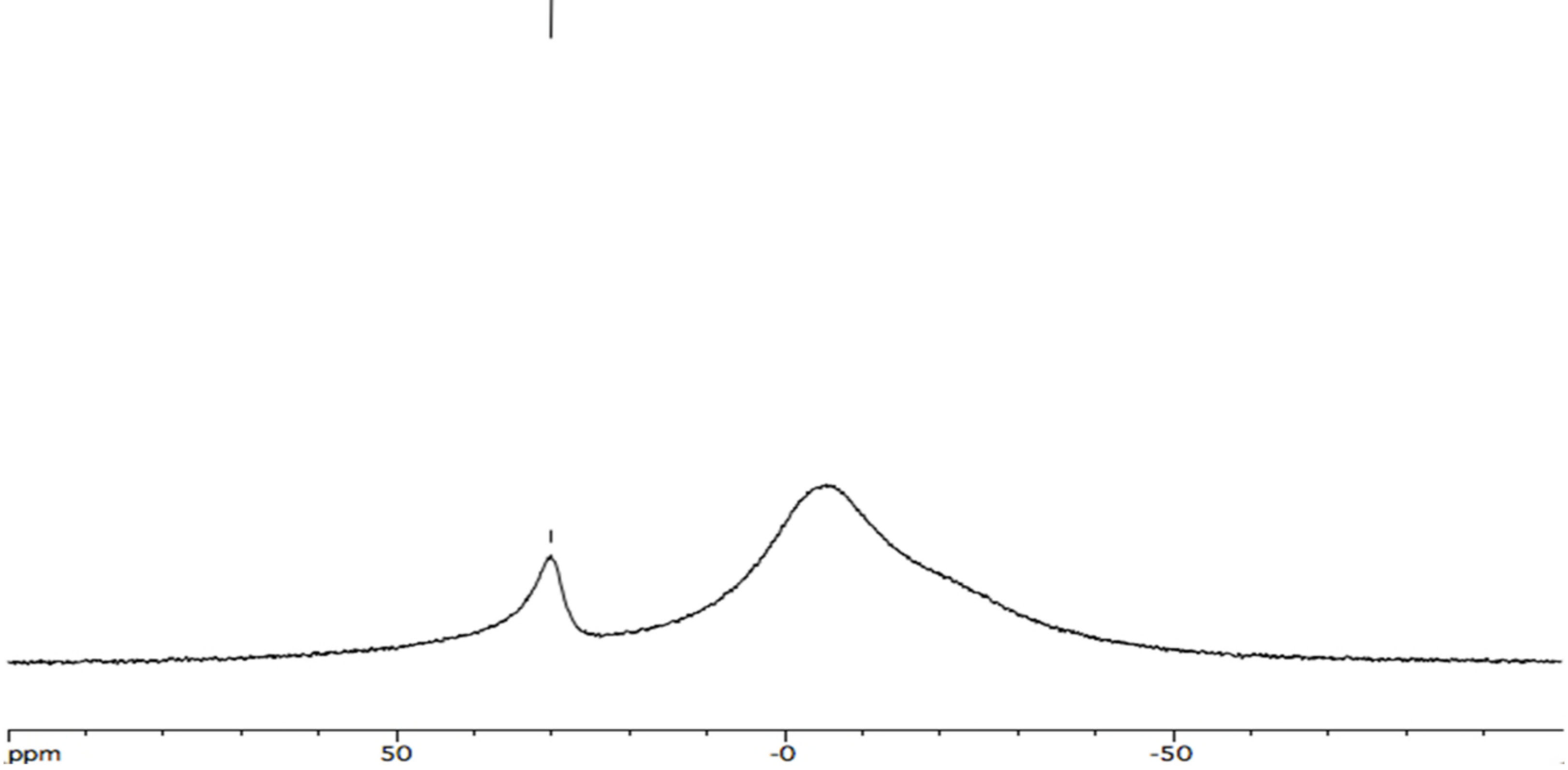

1-(3,4-dimethoxyphenyl)-*N*-(3-(4,4,5,5-tetramethyl-1,3,2-dioxaborolan-2-yl)phenyl)methanimine (**15**) – IR

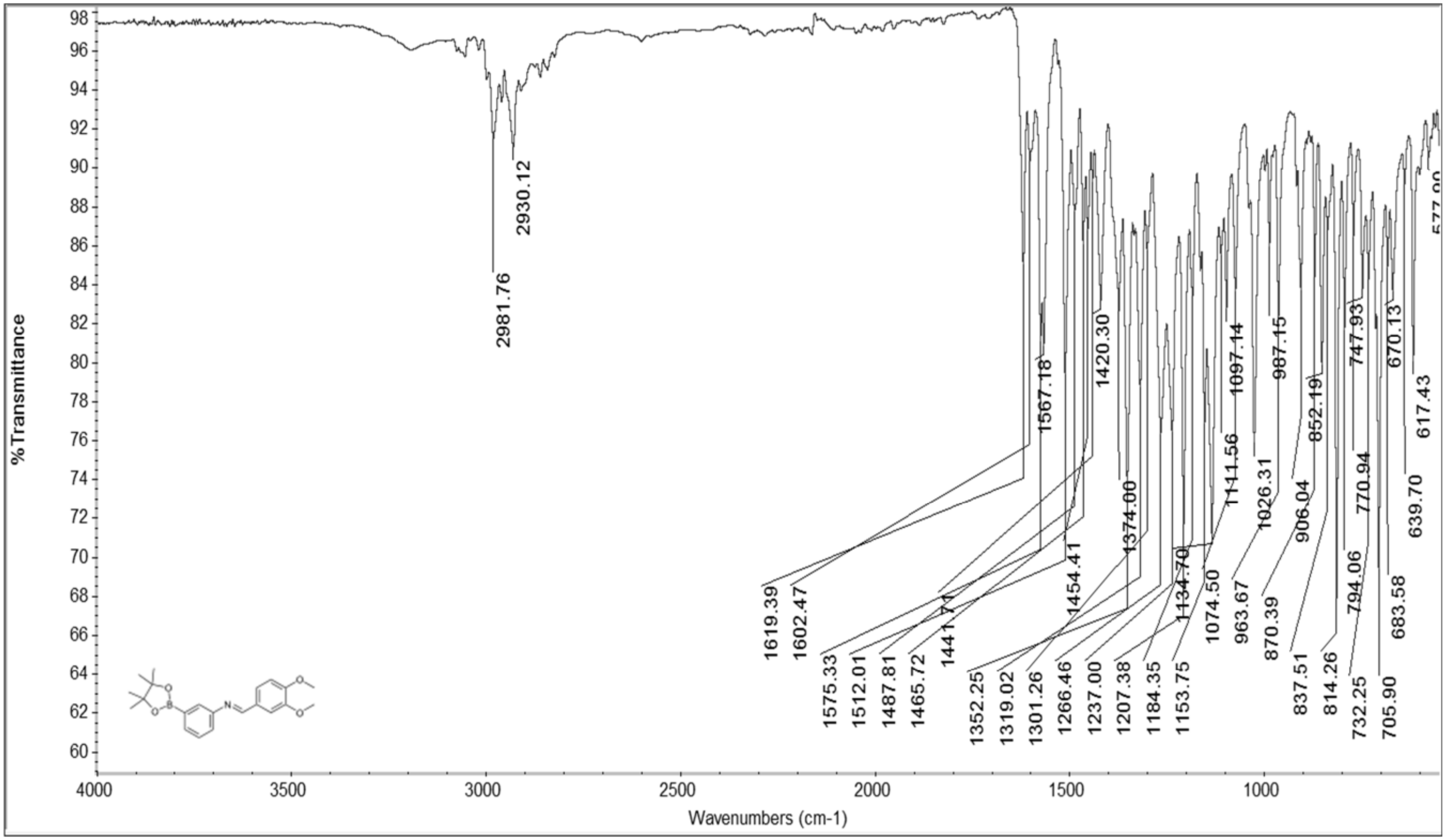

1-(3,4-dimethoxyphenyl)-*N*-(3-(4,4,5,5-tetramethyl-1,3,2-dioxaborolan-2-yl)phenyl)methanimine (**15**) – HRMS

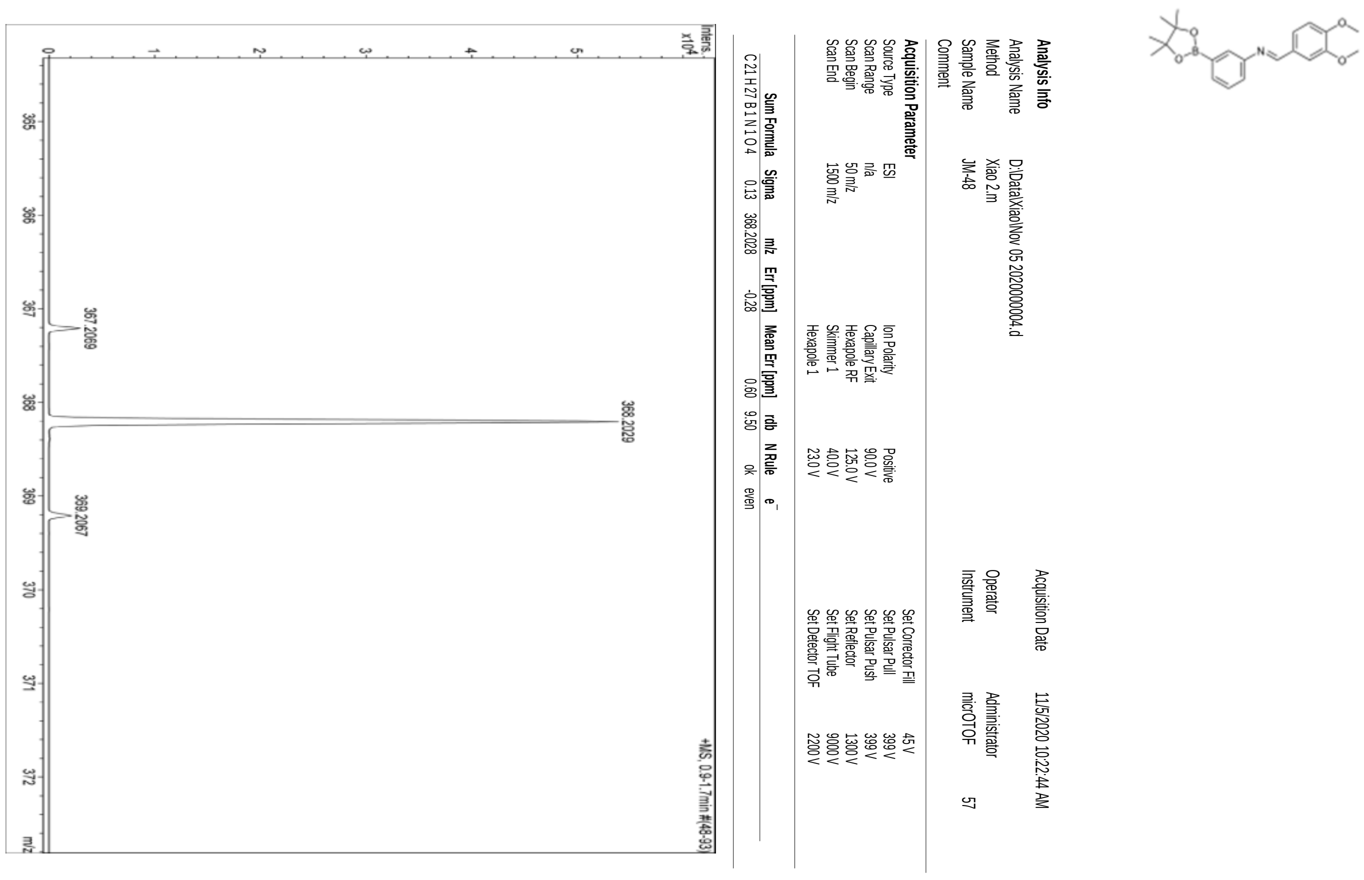

4-tetradecyl-*N*-(3,4-dimethoxybenzyl)aniline (**17**) – ^{1}H NMR

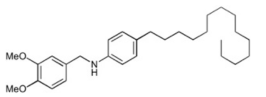

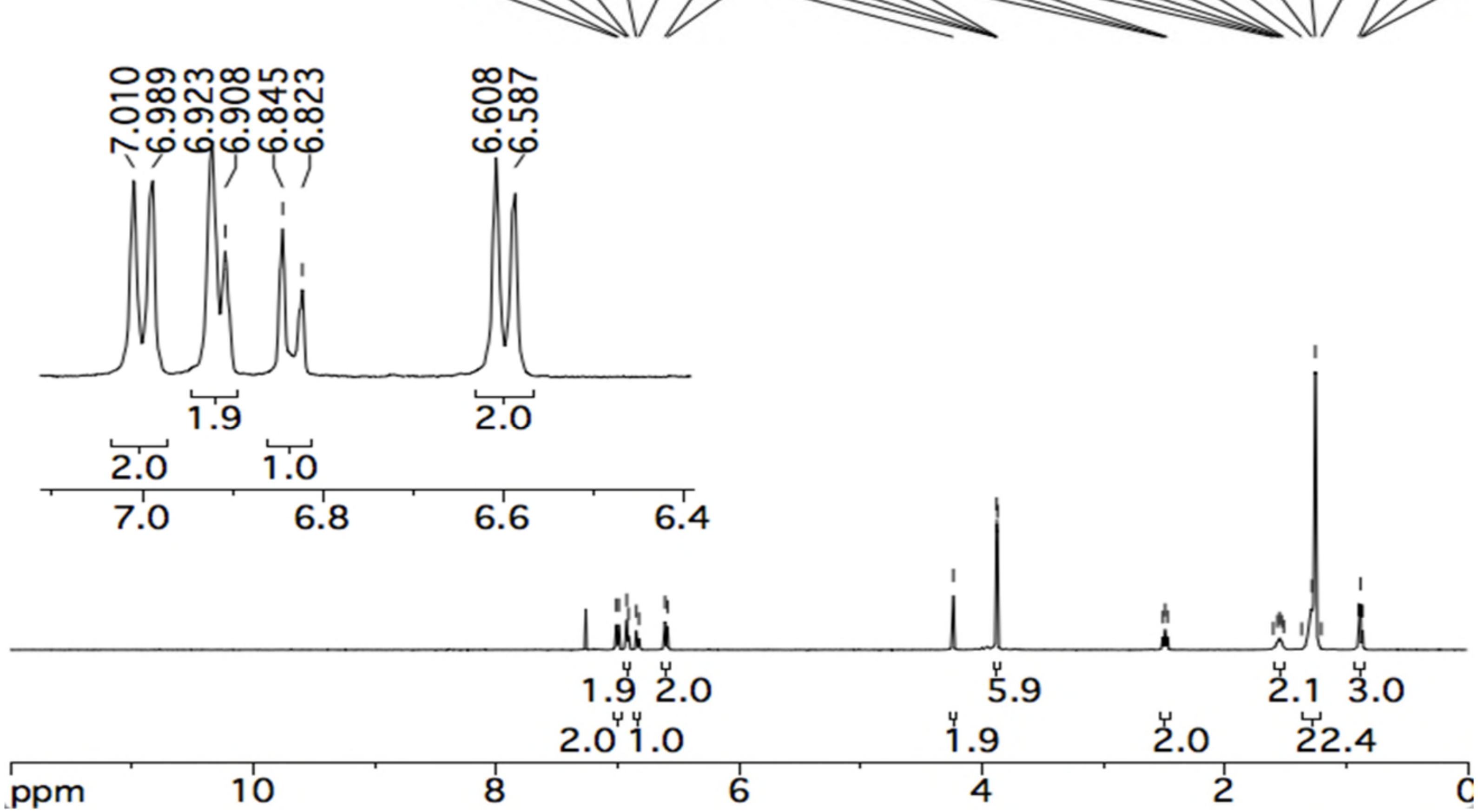

4-tetradecyl-*N*-(3,4-dimethoxybenzyl)aniline (**17**) – ^{13}C NMR

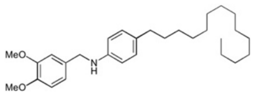

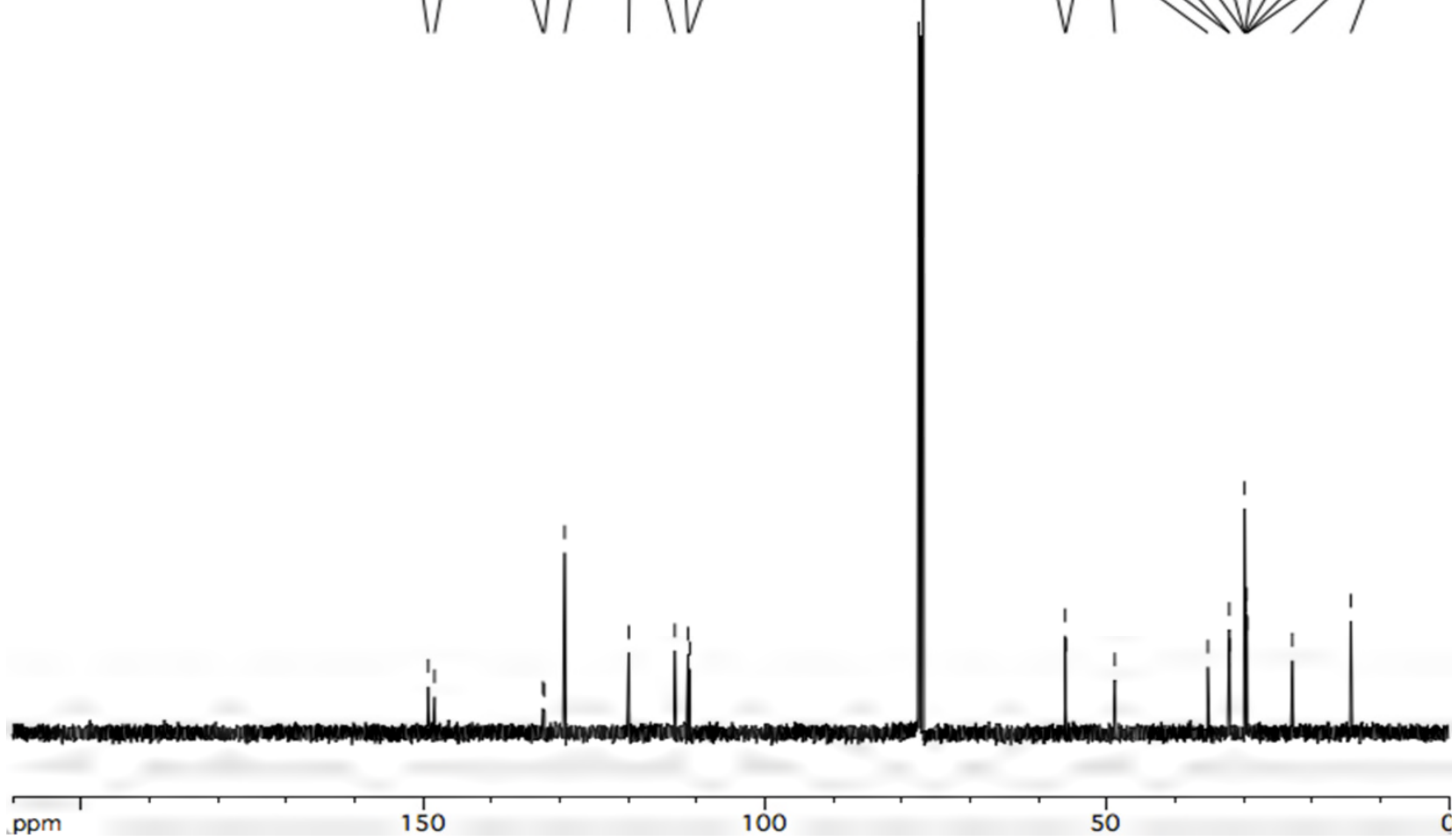

4-tetradecyl-*N*-(3,4-dimethoxybenzyl)aniline (**17**) – IR

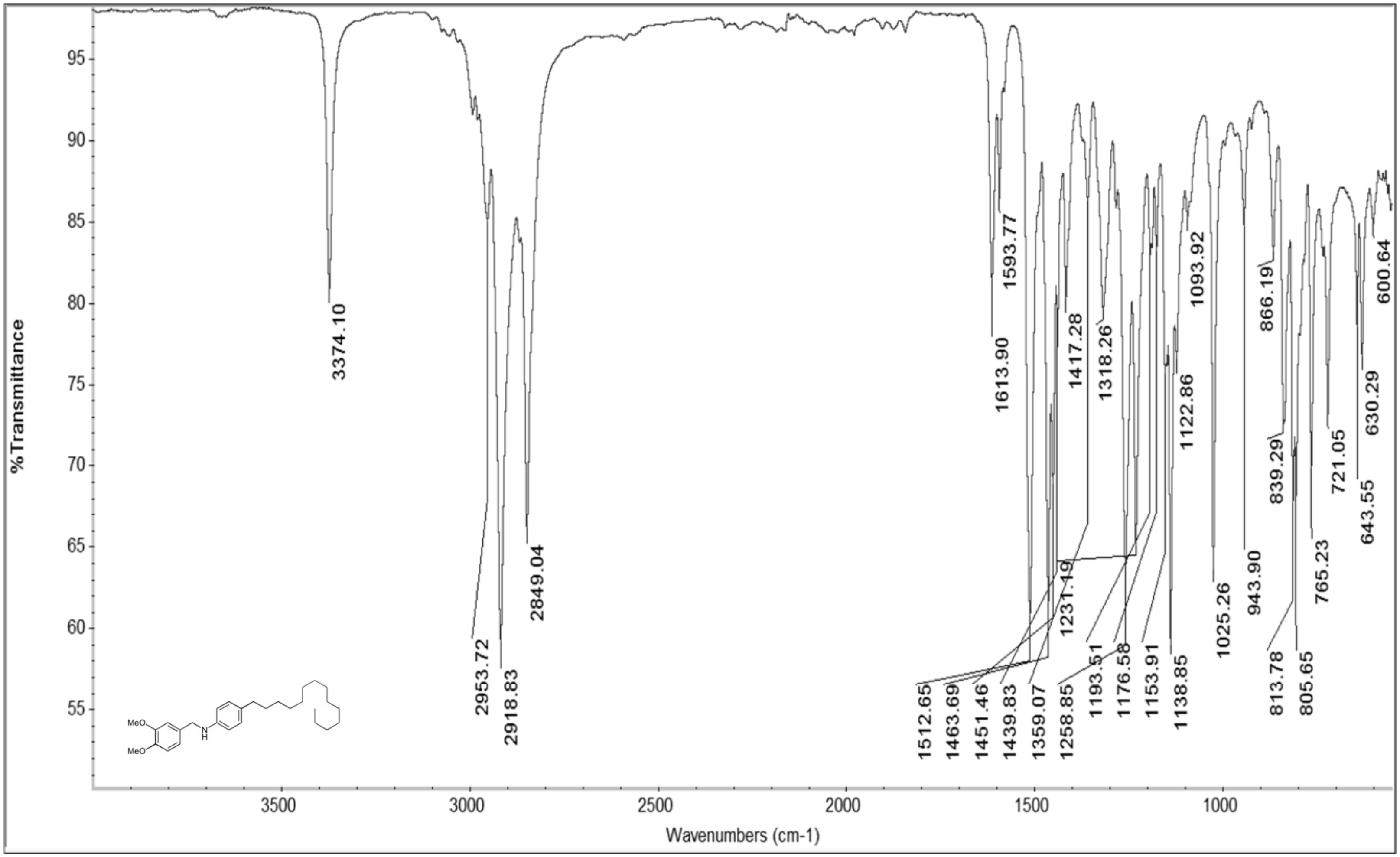

4-tetradecyl-*N*-(3,4-dimethoxybenzyl)aniline (**17**) – HRMS

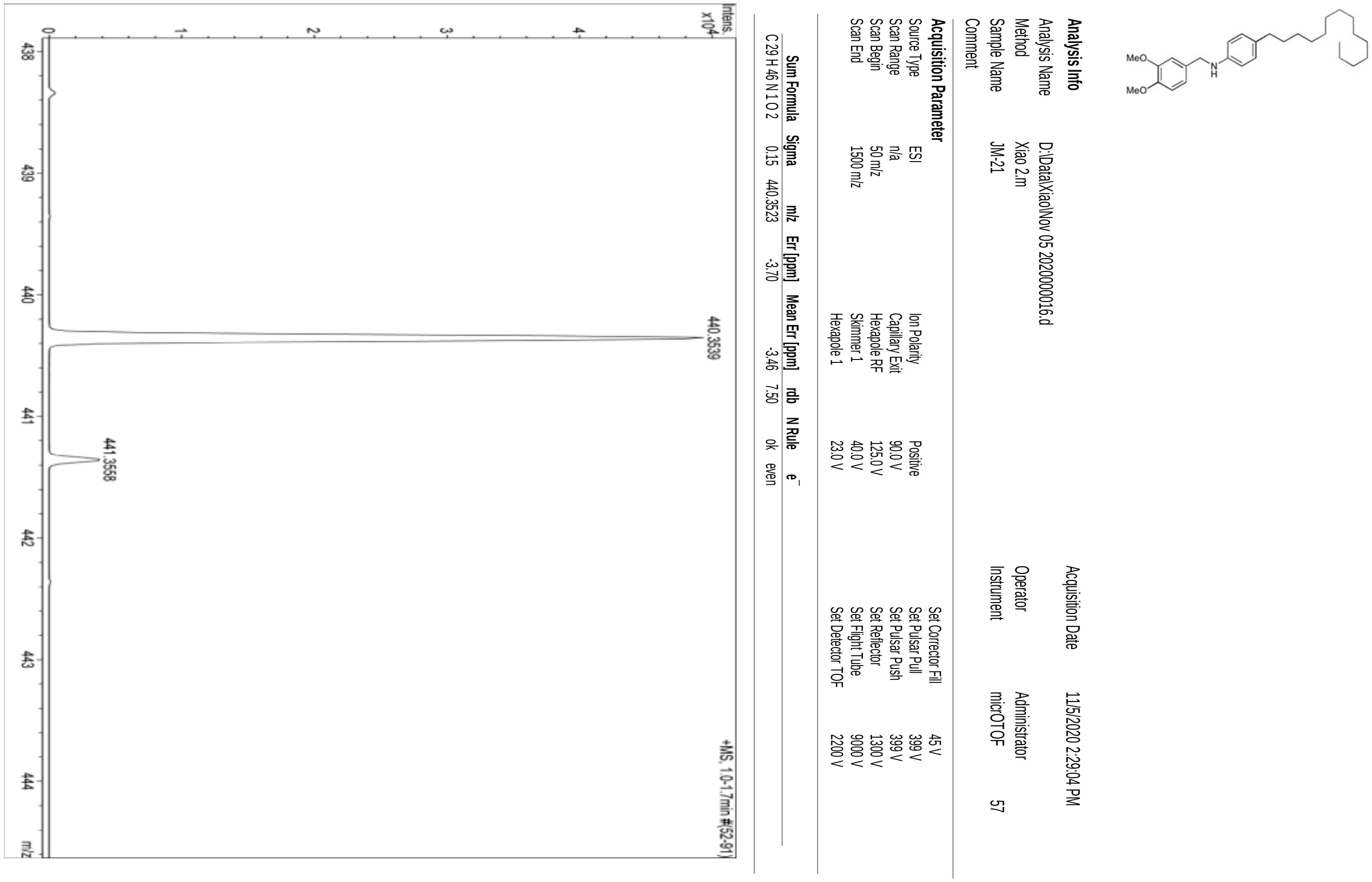

4-butyl-*N*-(3,4-dimethoxybenzyl)aniline (**18**) -^{1}H NMR

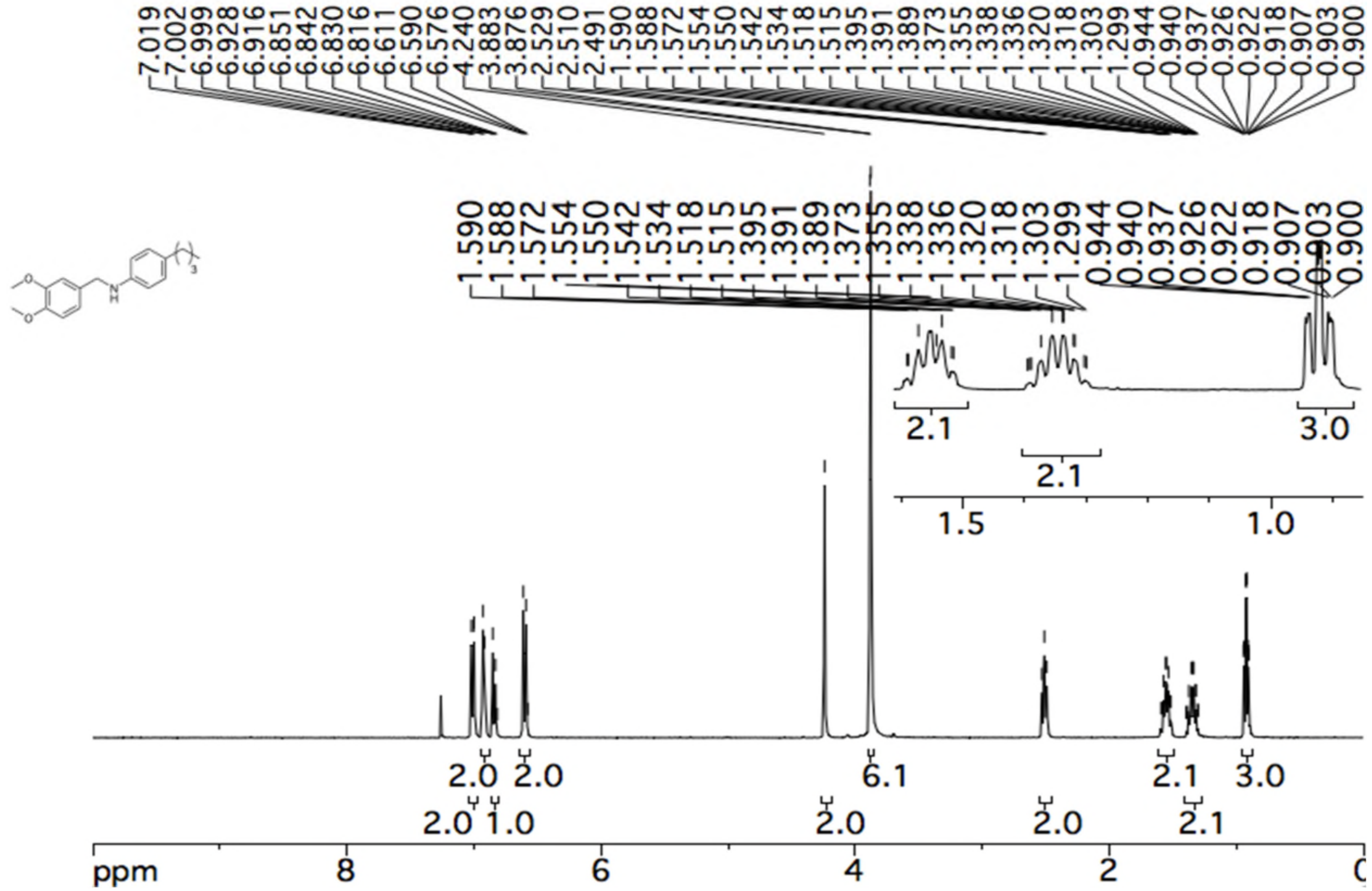

4-butyl-*N*-(3,4-dimethoxybenzyl)aniline (**18**) -^{13}C NMR

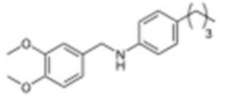

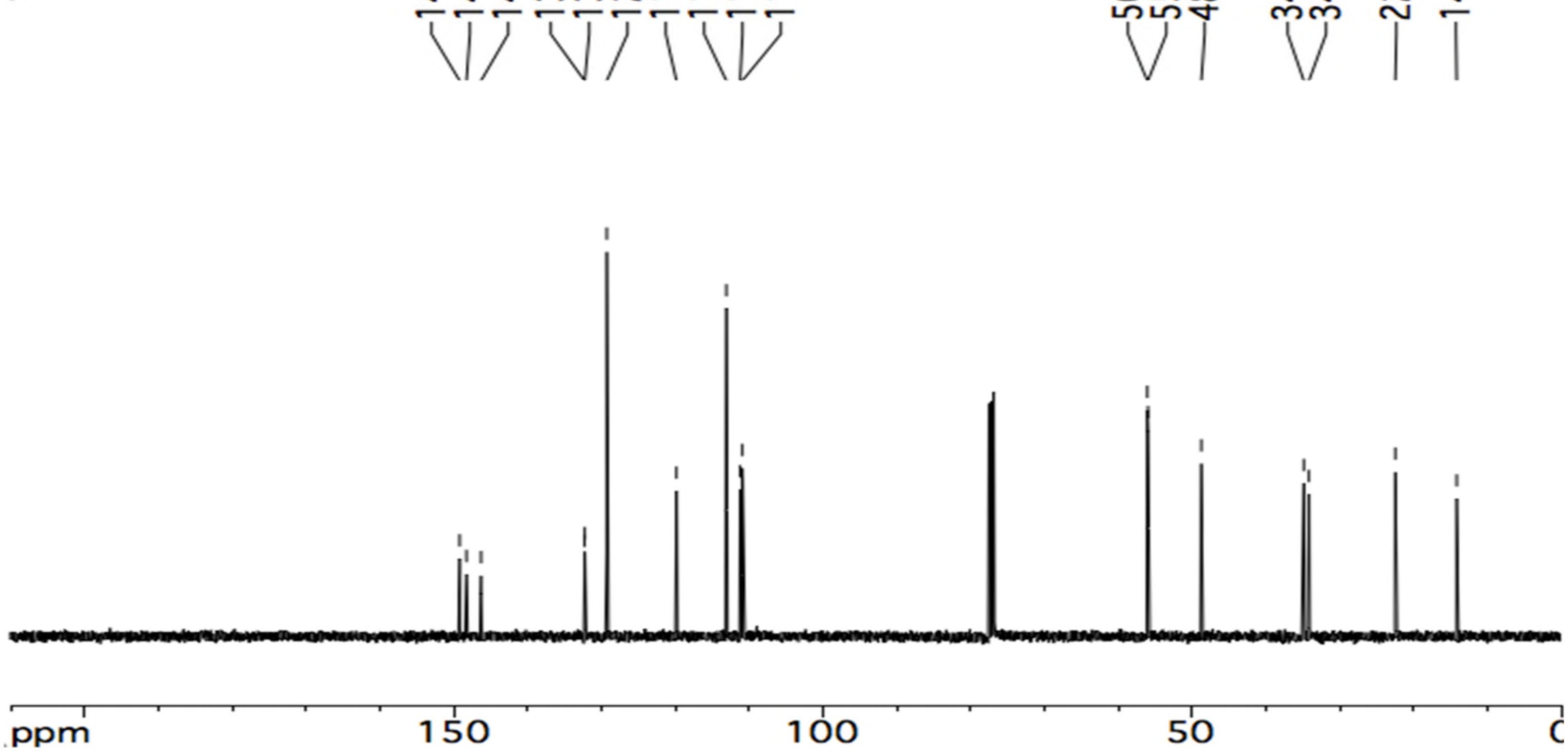

4-butyl-*N*-(3,4-dimethoxybenzyl)aniline (**18**) - IR

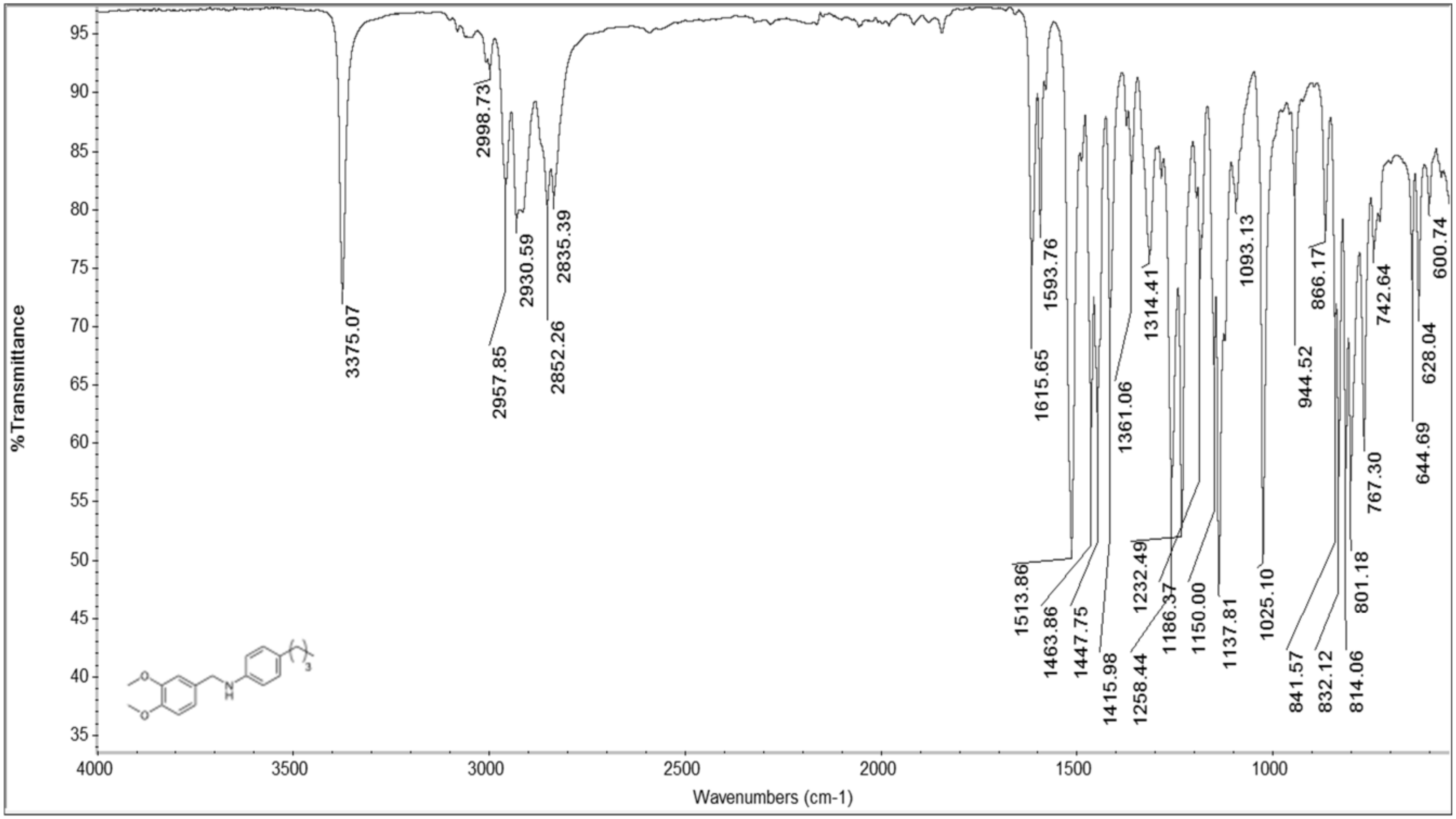

4-butyl-*N*-(3,4-dimethoxybenzyl)aniline (**18**) - HRMS

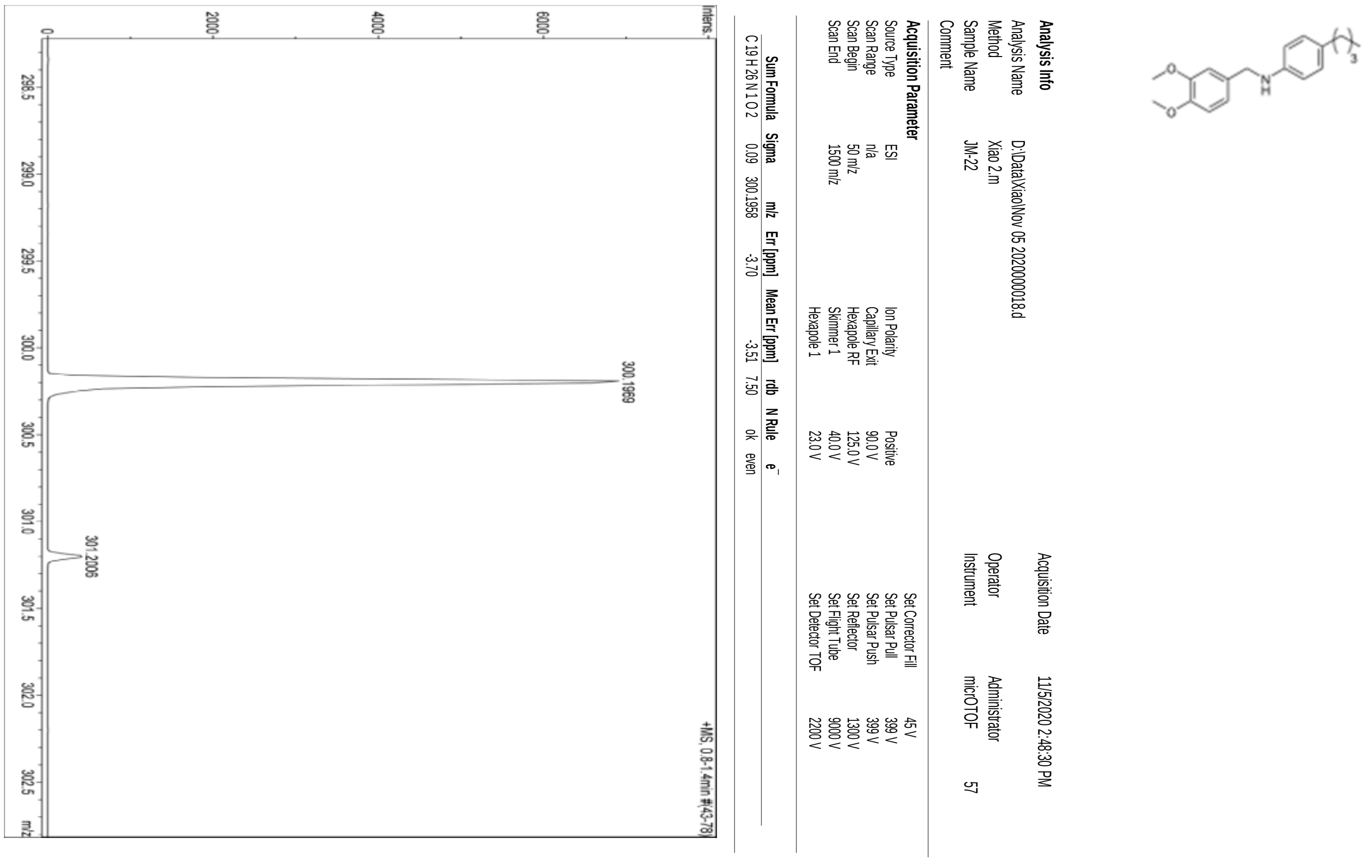

3,4-dimethoxy-*N*-[4-(4,4,5,5-tetramethyl-1,3,2-dioxaborolan-2-yl)phenyl]- benzenemethanamine (**19**) – ^{1}H NMR

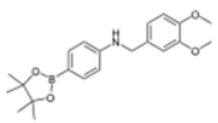

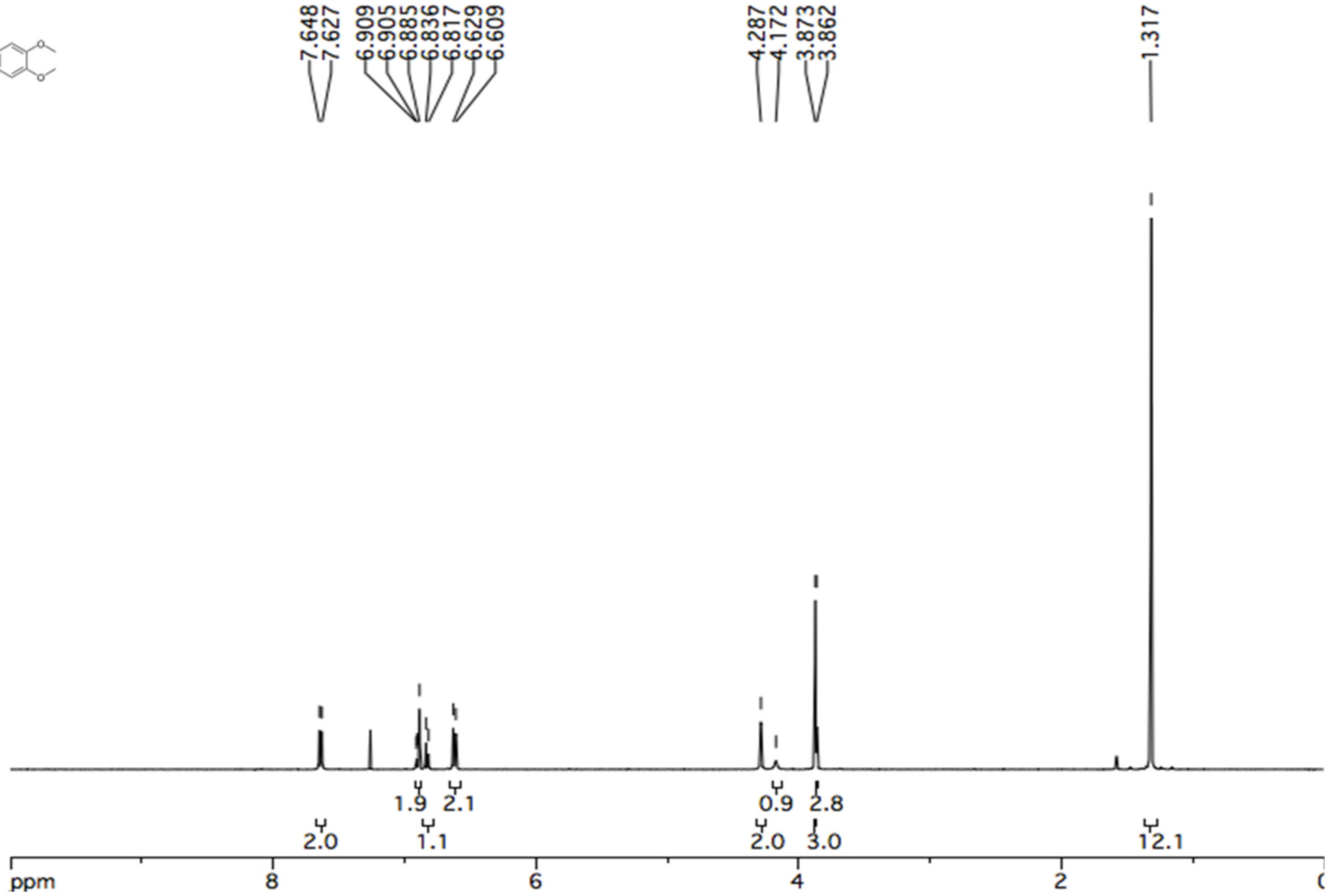

3,4-dimethoxy-*N*-[4-(4,4,5,5-tetramethyl-1,3,2-dioxaborolan-2-yl)phenyl]- benzenemethanamine (**19**) – ^{13}C NMR

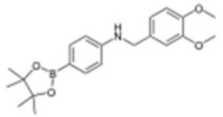

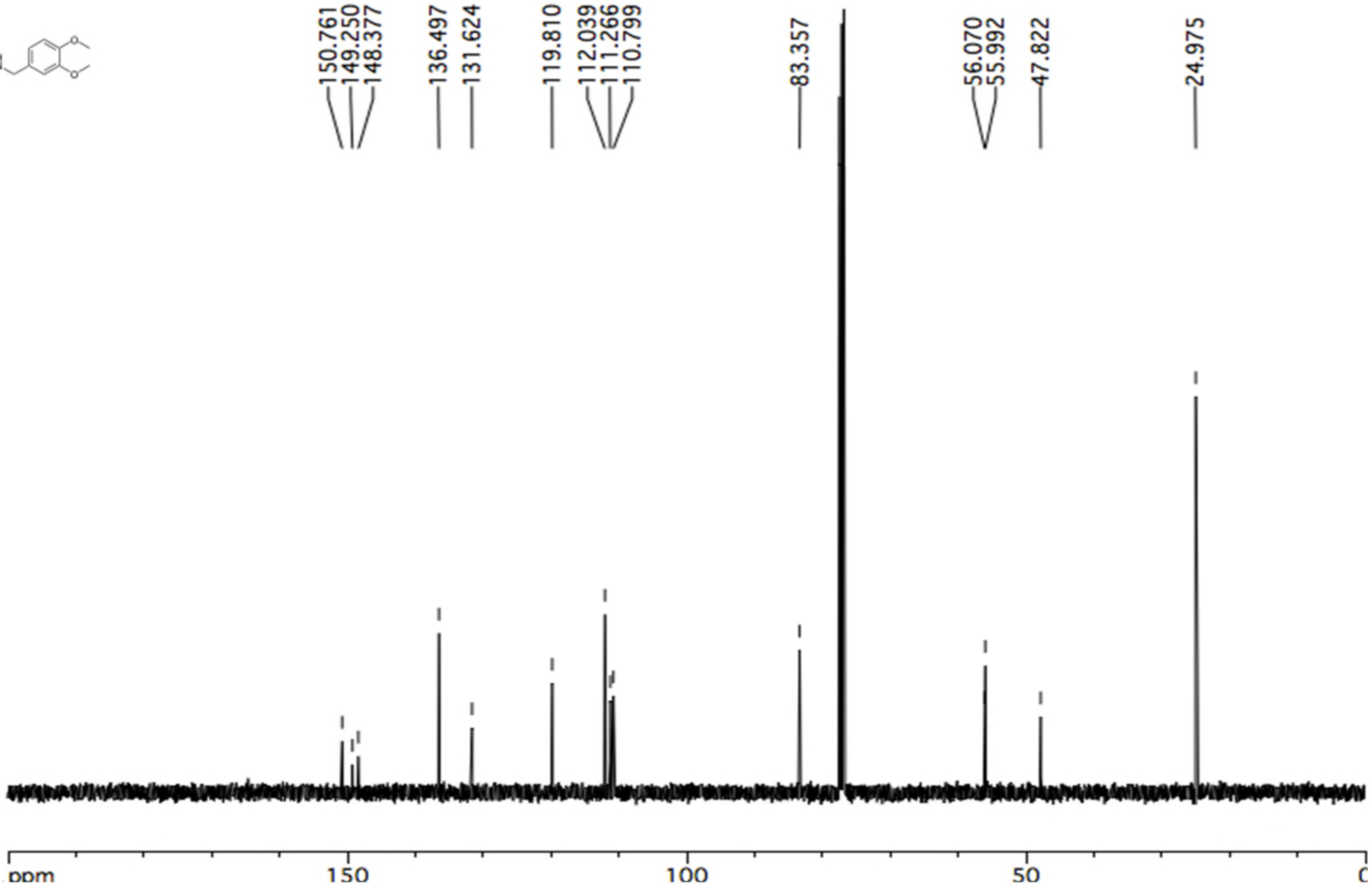

3,4-dimethoxy-*N*-[4-(4,4,5,5-tetramethyl-1,3,2-dioxaborolan-2-yl)phenyl]- benzenemethanamine (**19**) – ^{11}B NMR

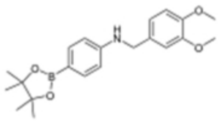

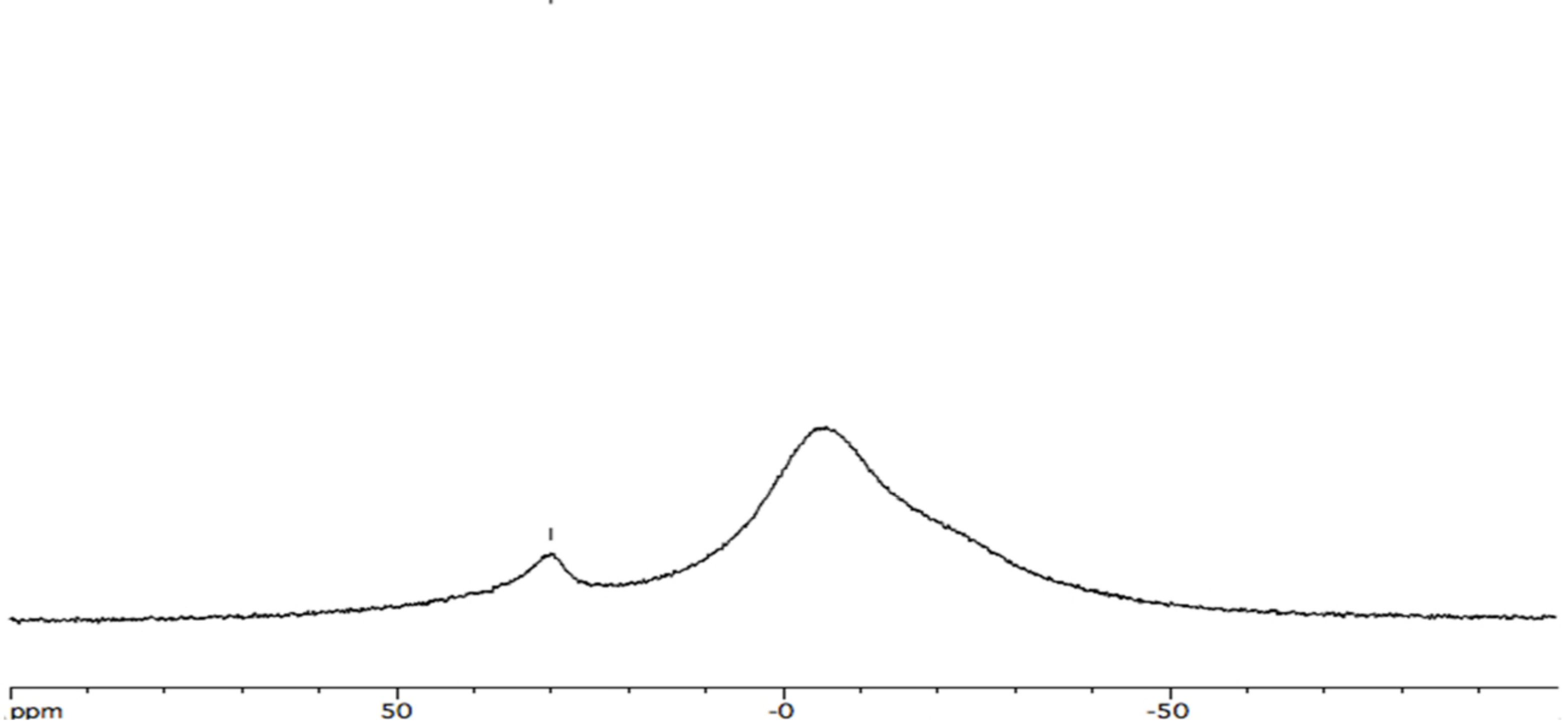

3,4-dimethoxy-*N*-[4-(4,4,5,5-tetramethyl-1,3,2-dioxaborolan-2-yl)phenyl]- benzenemethanamine (**19**) – IR

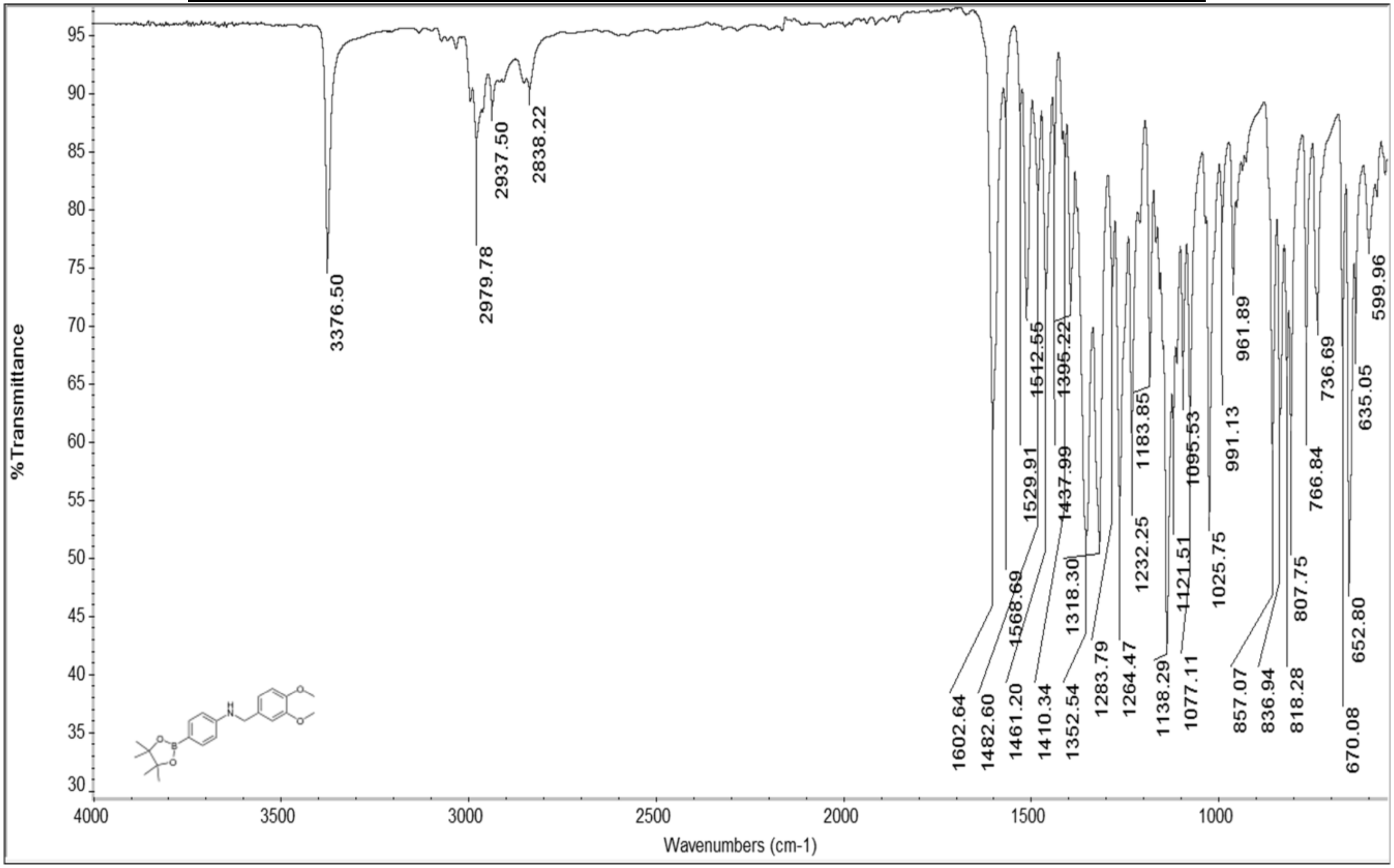

3,4-dimethoxy-*N*-[4-(4,4,5,5-tetramethyl-1,3,2-dioxaborolan-2-yl)phenyl]- benzenemethanamine (**19**) – HRMS

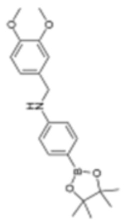

Analysis Info

Analysis Name	D:\Data\Xiao\Nov 05 2020000017.d	Acquisition Date	11/5/2020 2:40:02 PM
Method	Xiao 2.m	Operator	Administrator
Sample Name	JM-55	Instrument	micrOTOF 57
Comment			

Acquisition Parameter

Source Type	ESI	Ion Polarity	Positive	Set Corrector Fill	45 V
Scan Range	n/a	Capillary Exit	90.0 V	Set Pulsar Pull	399 V
Scan Begin	50 m/z	Hexapole RF	125.0 V	Set Pulsar Push	399 V
Scan End	1500 m/z	Skimmer 1	40.0 V	Set Reflector	1300 V
		Hexapole 1	23.0 V	Set Flight Tube	9000 V
				Set Detector TOF	2200 V

Sum Formula	Sigma	m/z	Err [ppm]	Mean Err [ppm]	rdb	N Rule	e⁻
C 21 H 28 B 1 N 1 Na 1 O 4	0.14	392.2004	2.18	2.98	8.50	ok	even

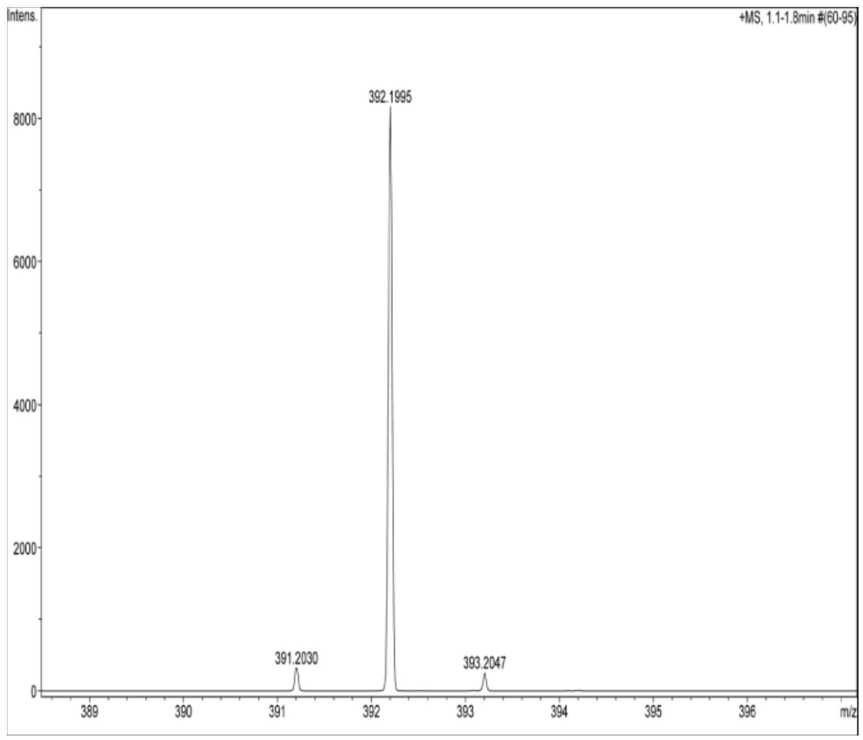

3,4-dimethoxy-*N*-[3-(4,4,5,5-tetramethyl-1,3,2-dioxaborolan-2-yl)phenyl]-benzenemethanamine (**20**) – ^{1}H NMR

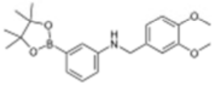

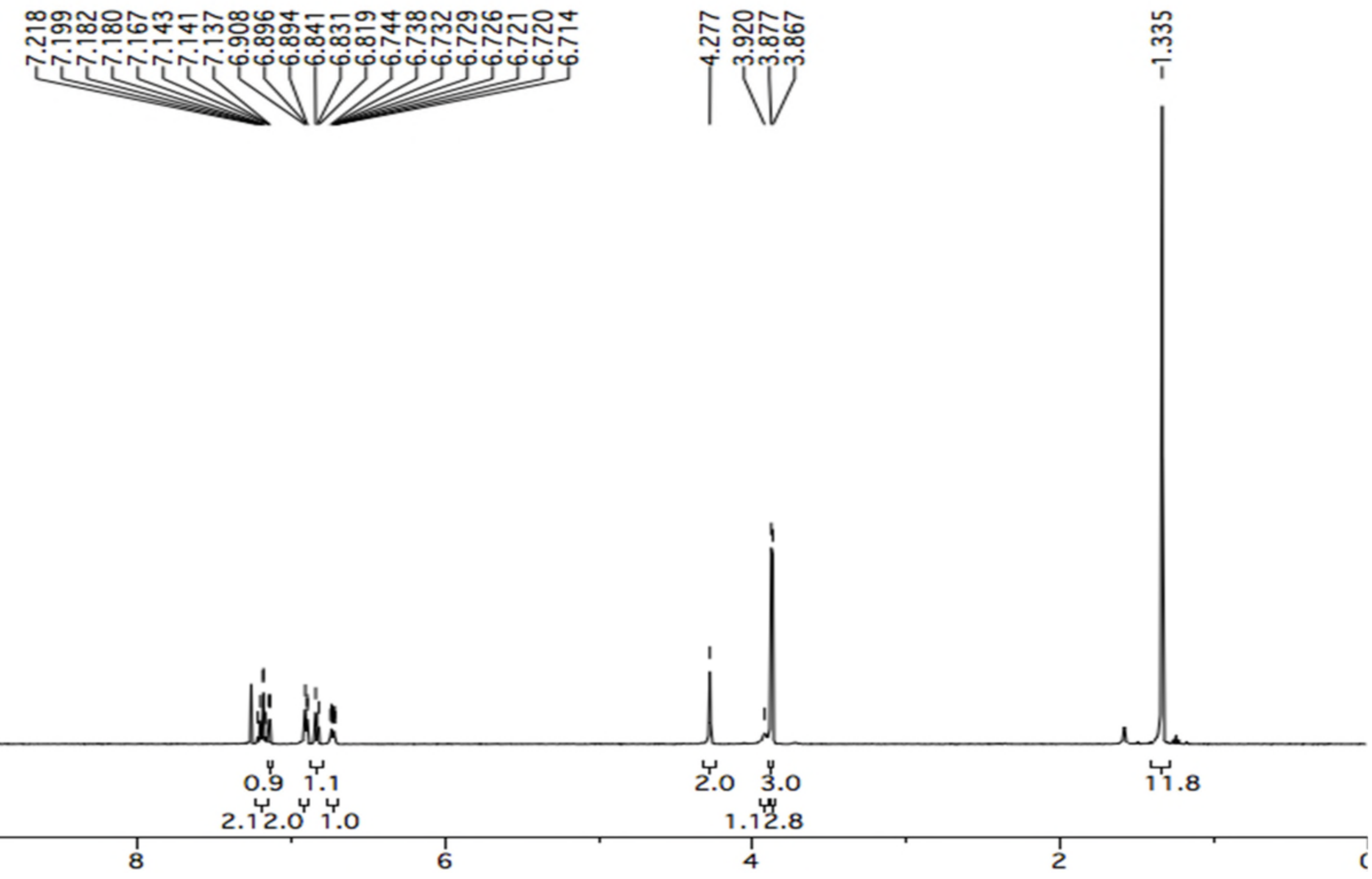

3,4-dimethoxy-*N*-[3-(4,4,5,5-tetramethyl-1,3,2-dioxaborolan-2-yl)phenyl]-benzenemethanamine (**20**) – ^{13}C NMR

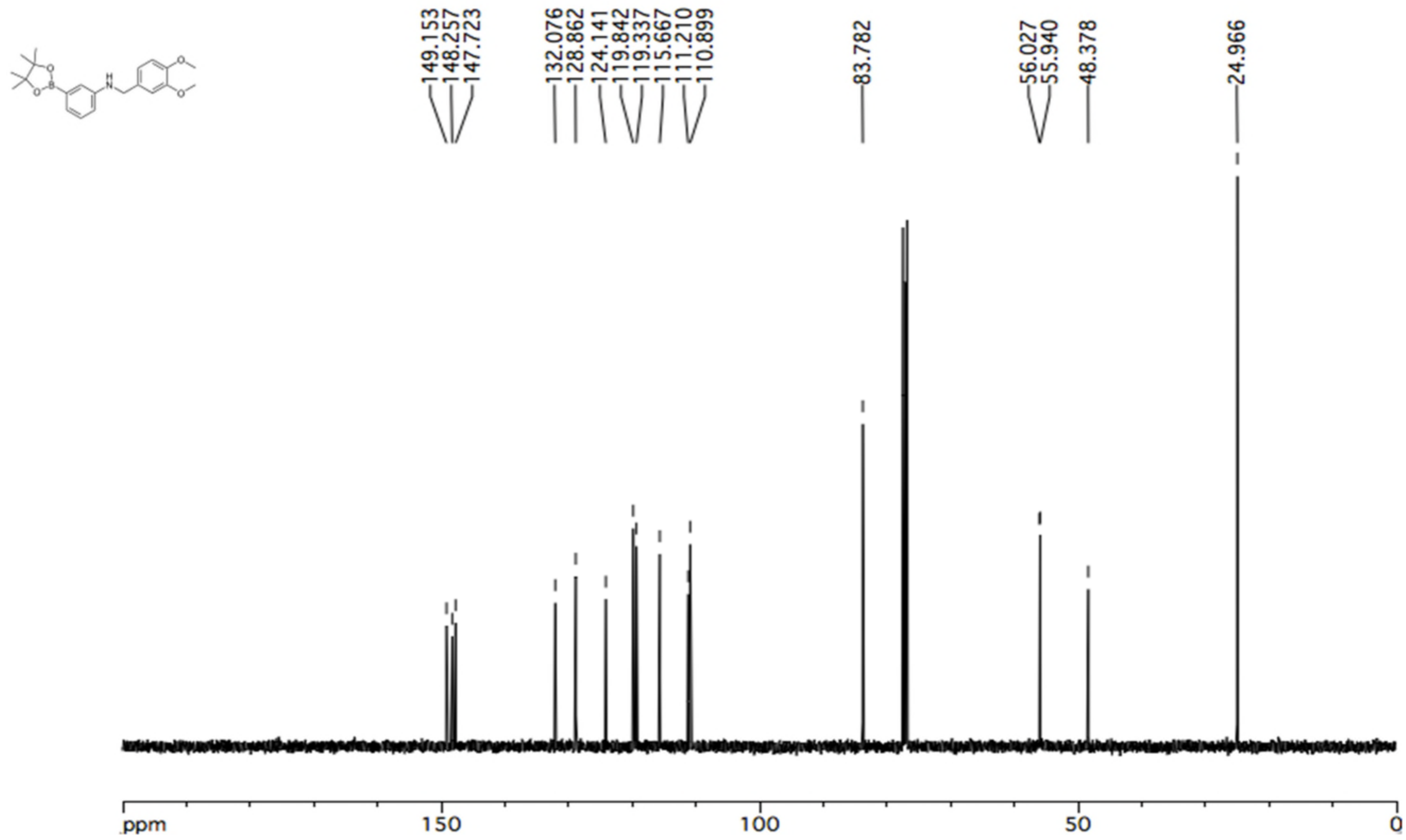

3,4-dimethoxy-*N*-[3-(4,4,5,5-tetramethyl-1,3,2-dioxaborolan-2-yl)phenyl]-benzenemethanamine (**20**) – ^{11}B NMR

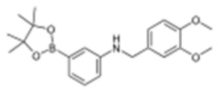

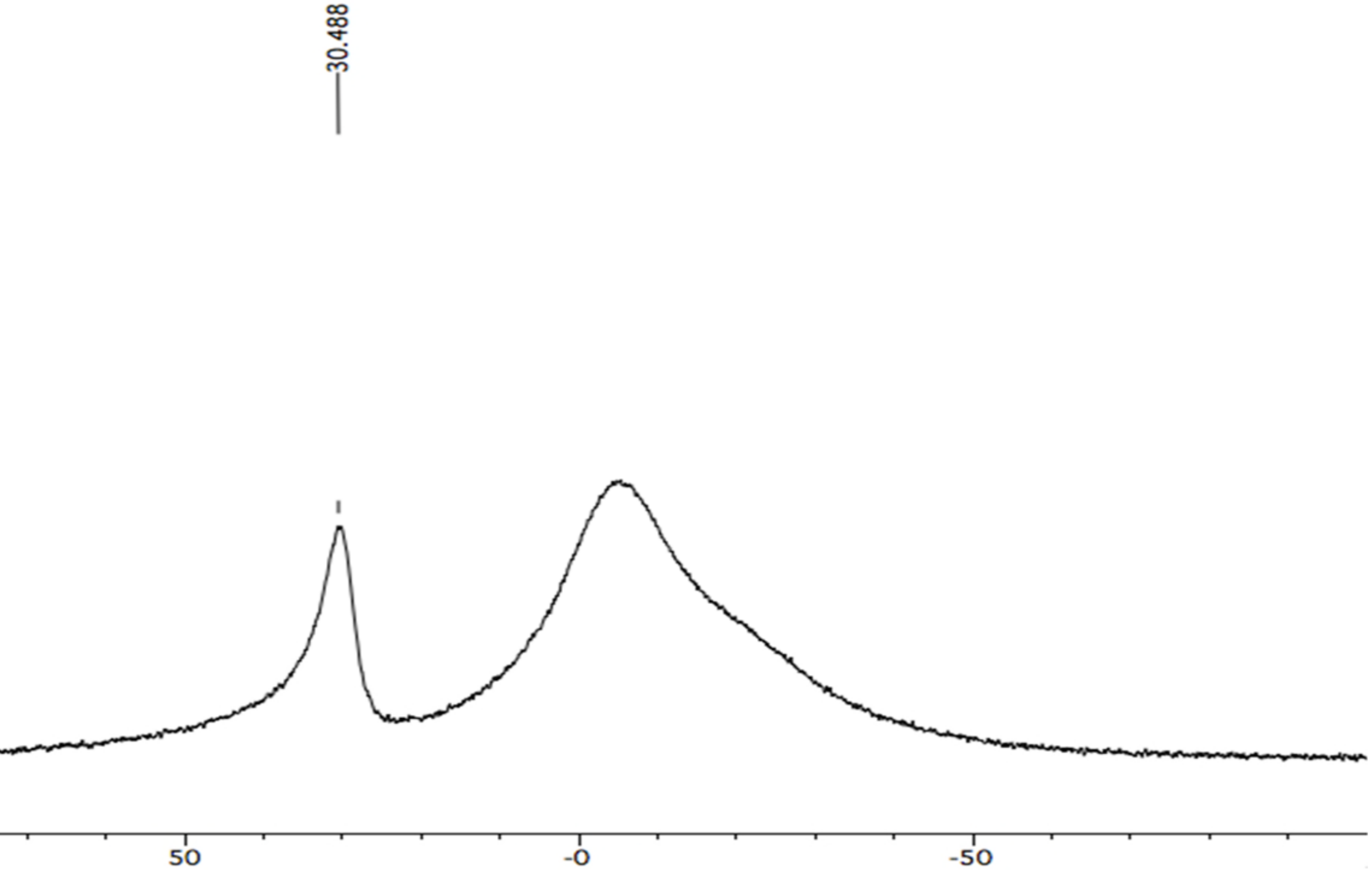

3,4-dimethoxy-*N*-[3-(4,4,5,5-tetramethyl-1,3,2-dioxaborolan-2-yl)phenyl]-benzenemethanamine (**20**) – IR

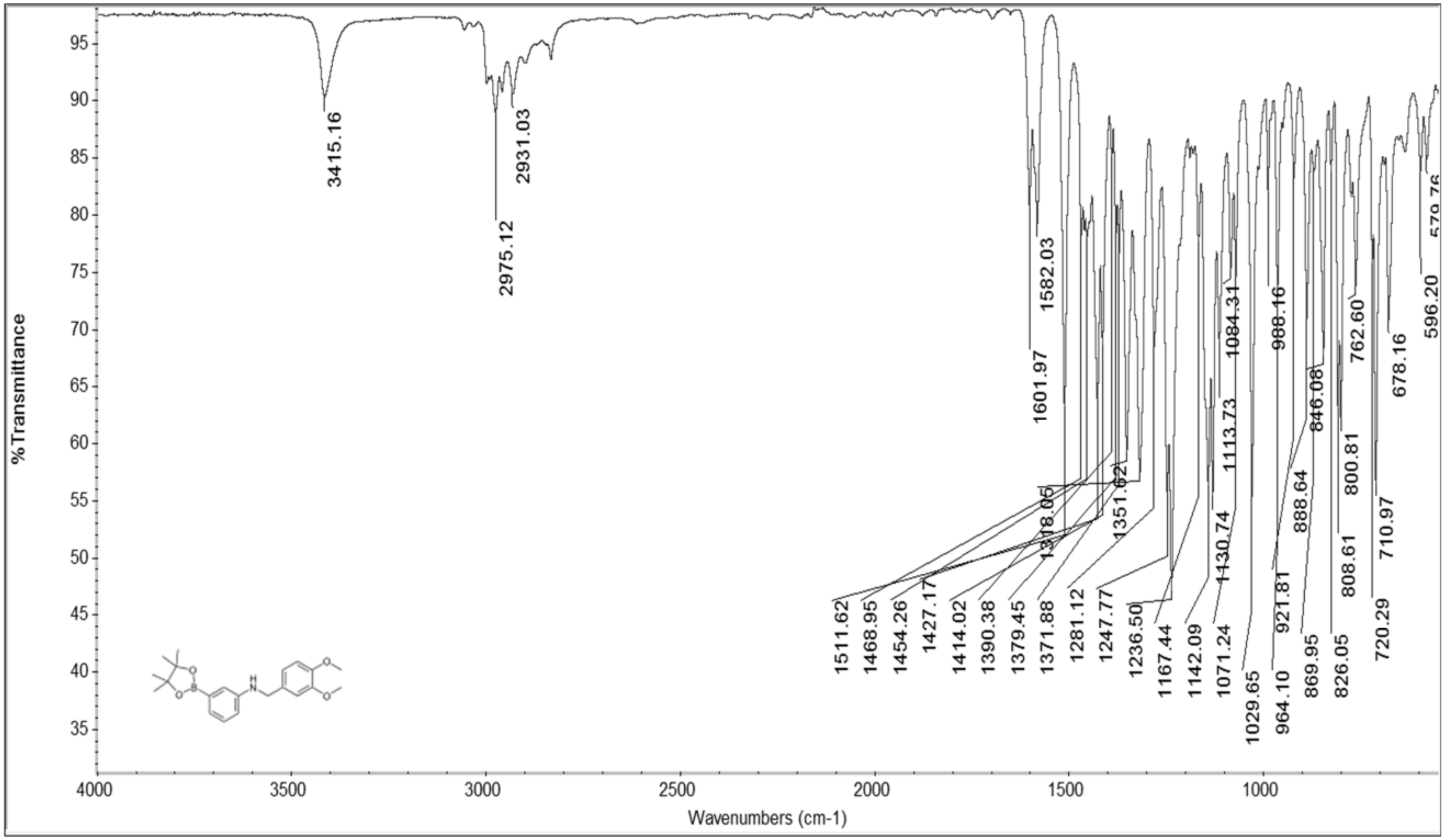

3,4-dimethoxy-*N*-[3-(4,4,5,5-tetramethyl-1,3,2-dioxaborolan-2-yl)phenyl]-benzenemethanamine (**20**) – HRMS

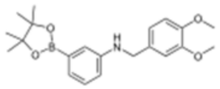

Analysis Info

Analysis Name	D:\Data\Xiao\Nov 05 2020000019.d	Acquisition Date	11/5/2020 2:57:06 PM
Method	Xiao 2.m	Operator	Administrator
Sample Name	JM-54	Instrument	micrOTOF 57
Comment			

Acquisition Parameter

Source Type	ESI	Ion Polarity	Positive	Set Corrector Fill	45 V
Scan Range	n/a	Capillary Exit	90.0 V	Set Pulsar Pull	399 V
Scan Begin	50 m/z	Hexapole RF	125.0 V	Set Pulsar Push	399 V
Scan End	1500 m/z	Skimmer 1	40.0 V	Set Reflector	1300 V
		Hexapole 1	23.0 V	Set Flight Tube	9000 V
				Set Detector TOF	2200 V

Sum Formula	Sigma	m/z	Err [ppm]	Mean Err [ppm]	rdb	N Rule	e⁻
C 21 H 28 B 1 N 1 Na 1 O 4	0.15	392.2004	-2.00	-0.38	8.50	ok	even

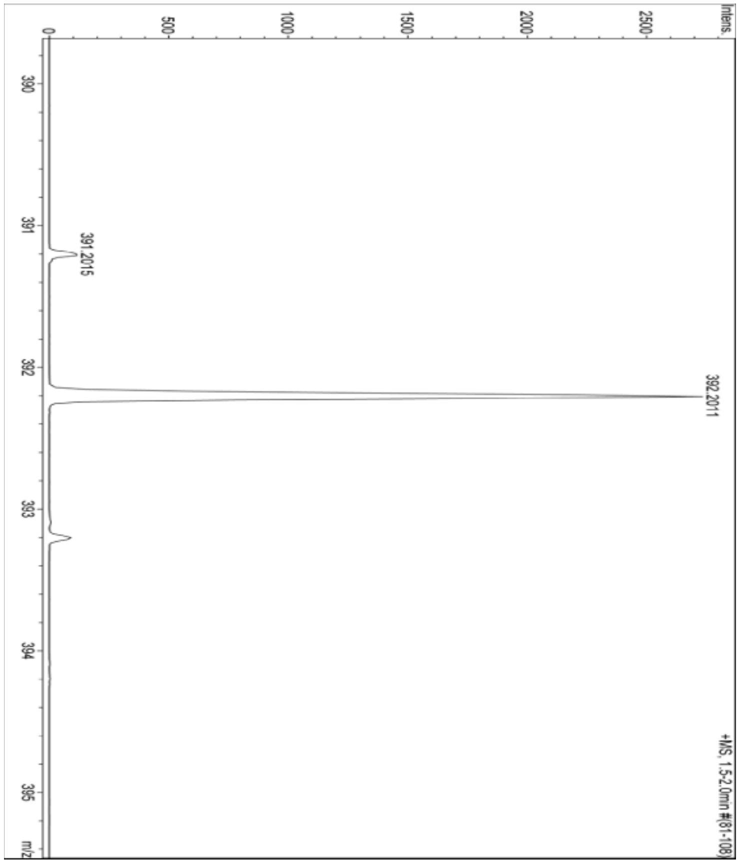

N-[3,4-dimethoxyphenyl)methyl]-*N*-(phenyl)-hexanamide (**21**) – ^{1}H NMR

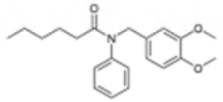

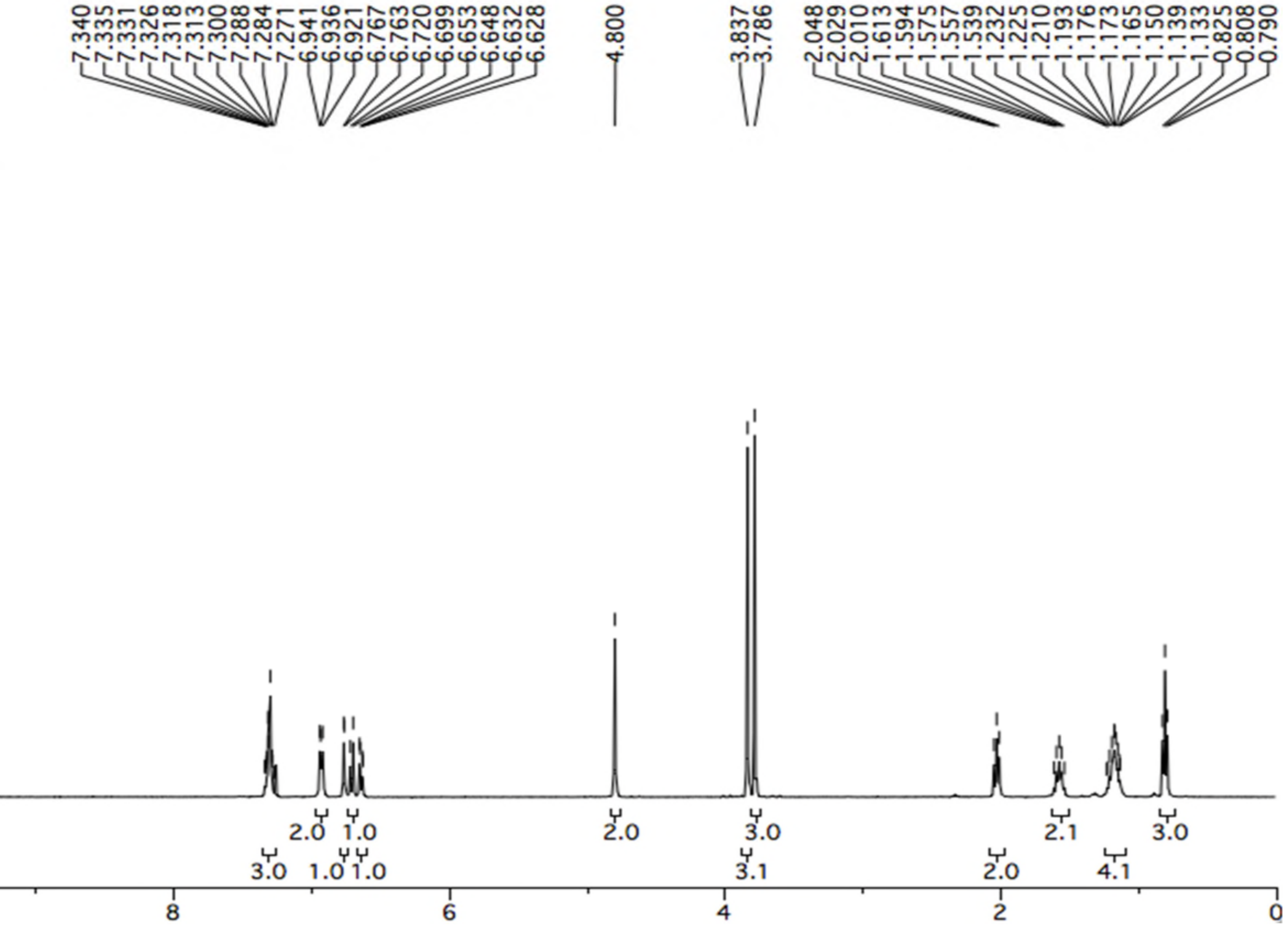

N-[3,4-dimethoxyphenyl)methyl]-*N*-(phenyl)-hexanamide (**21**) – ^{13}C NMR

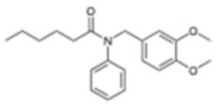

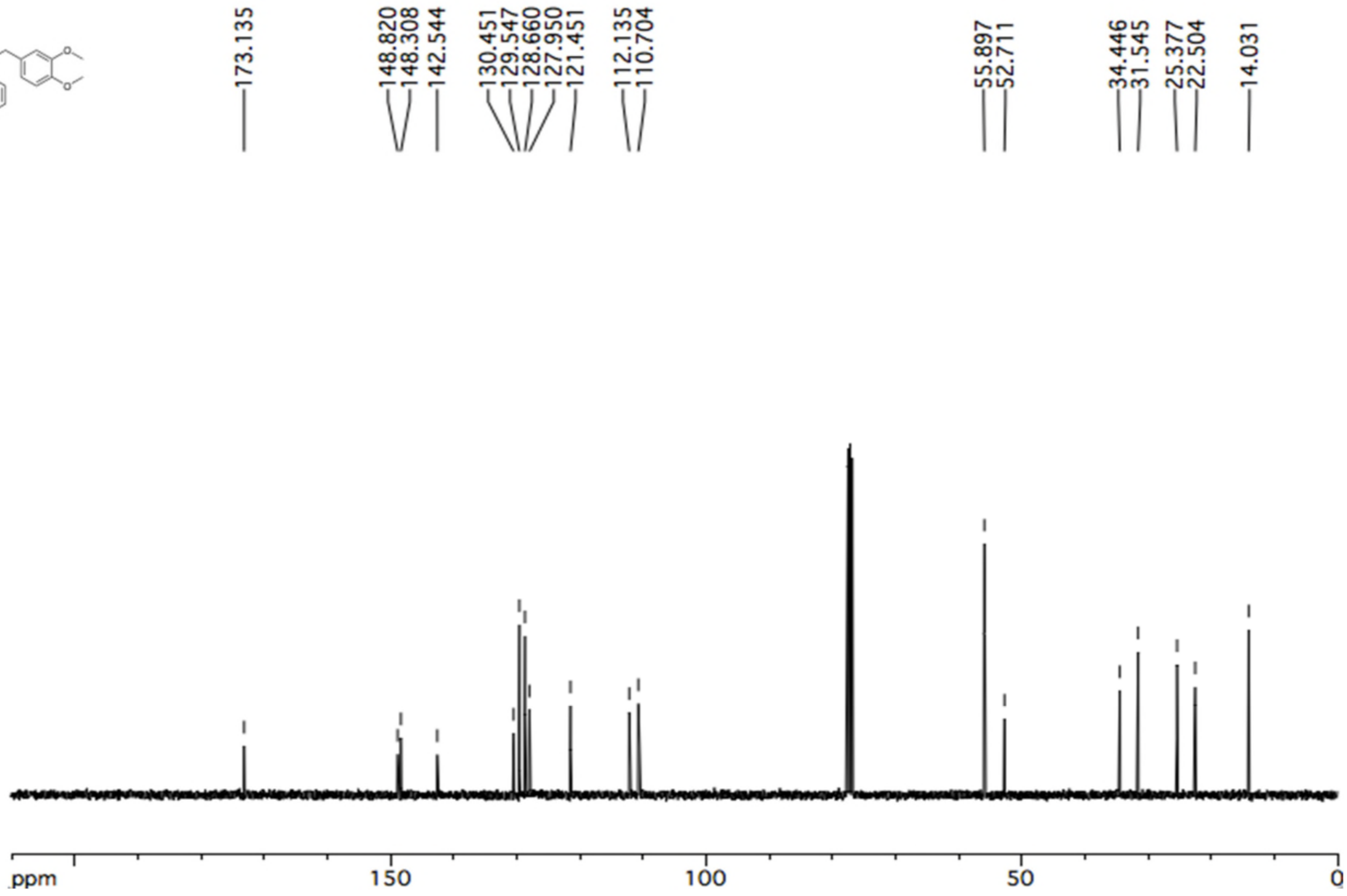

N-[3,4-dimethoxyphenyl)methyl]-*N*-(phenyl)-hexanamide (**21**) – IR

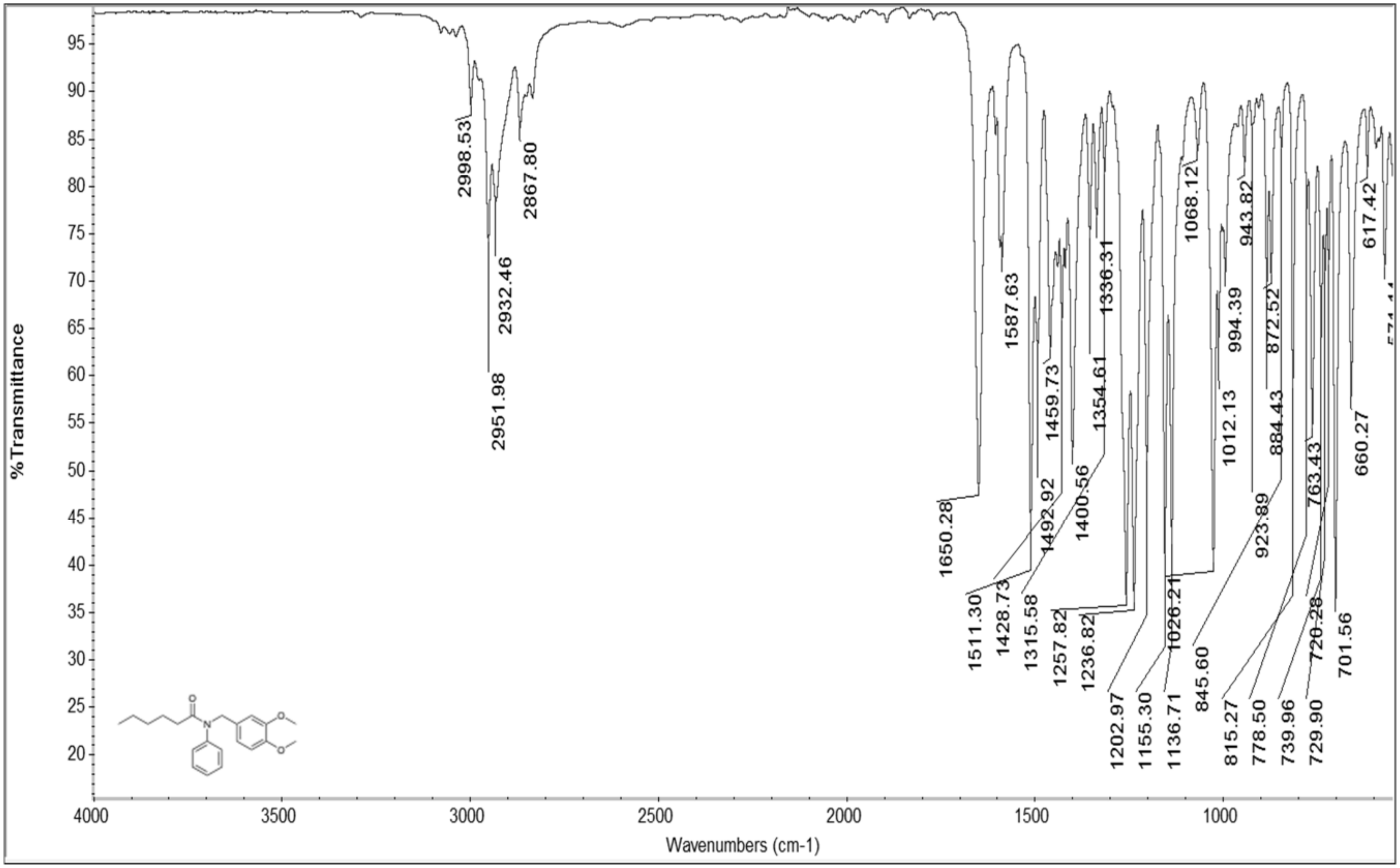

N-[3,4-dimethoxyphenyl)methyl]-*N*-(phenyl)-hexanamide (**21**) – HRMS

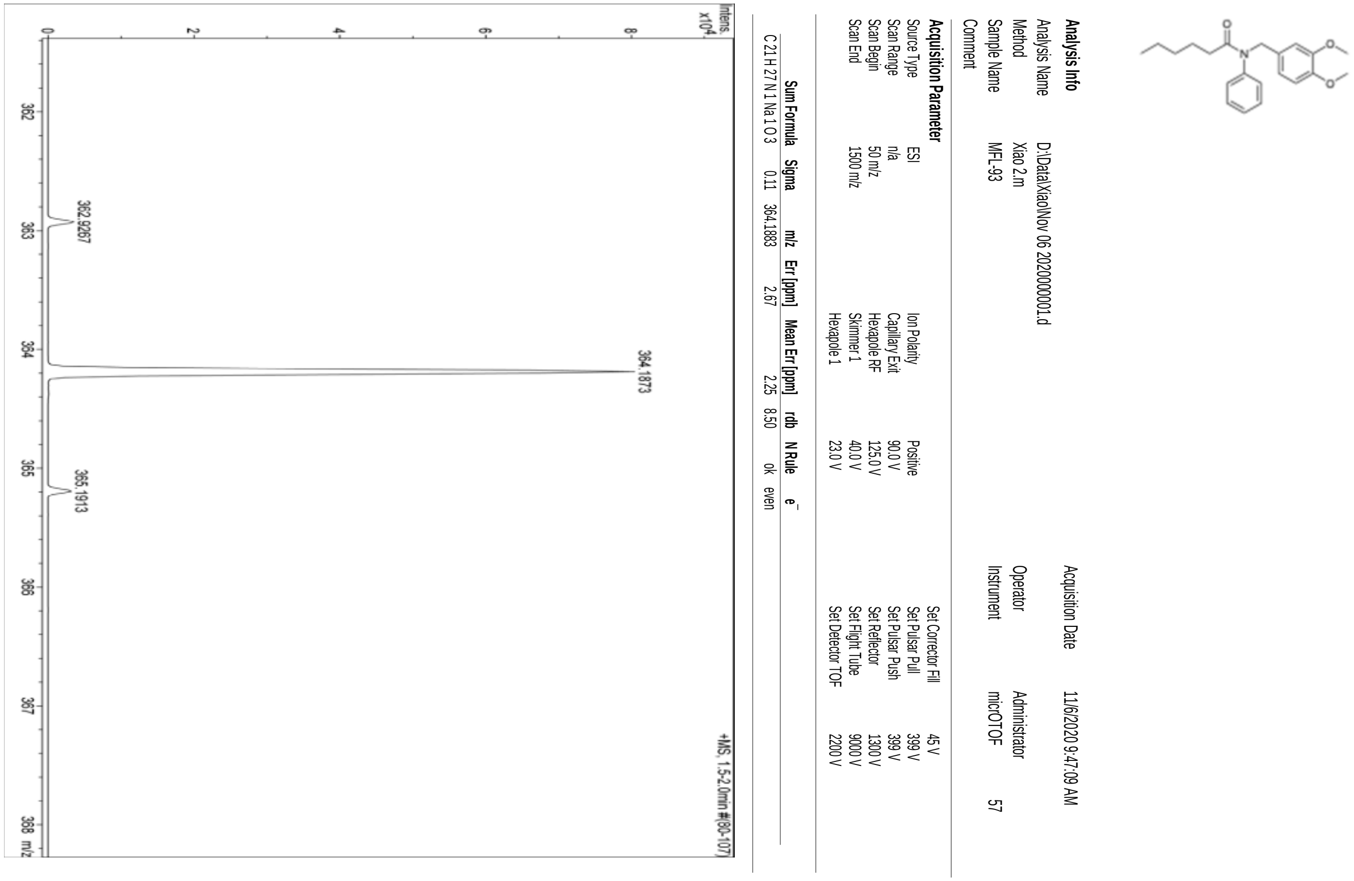

N-[3,4-dimethoxyphenyl)methyl]-*N*-(phenyl)-decanamide (**22**) – ^{1}H NMR

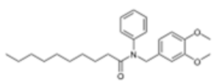

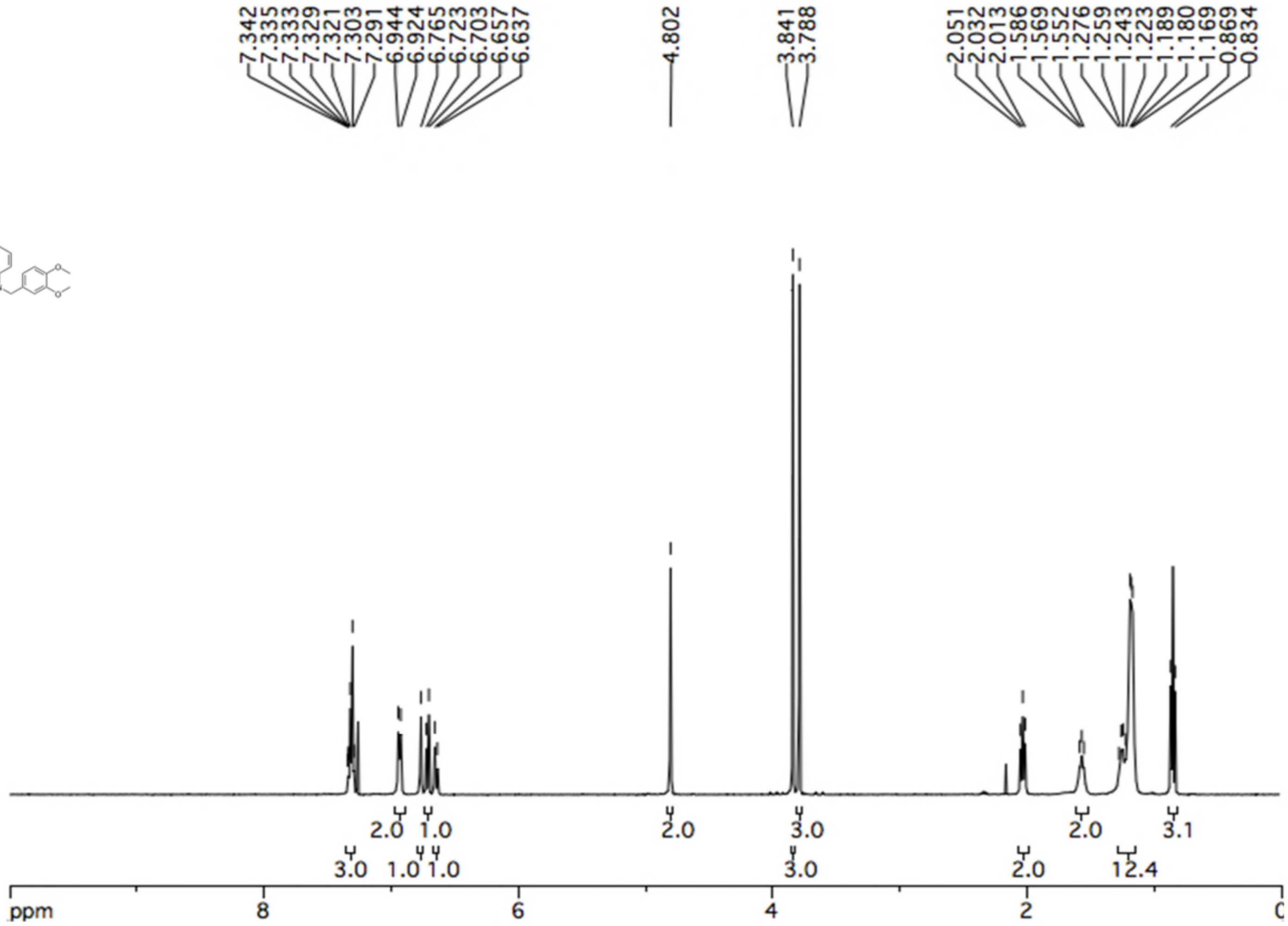

N-[3,4-dimethoxyphenyl)methyl]-*N*-(phenyl)-decanamide (**22**) – ^{13}C NMR

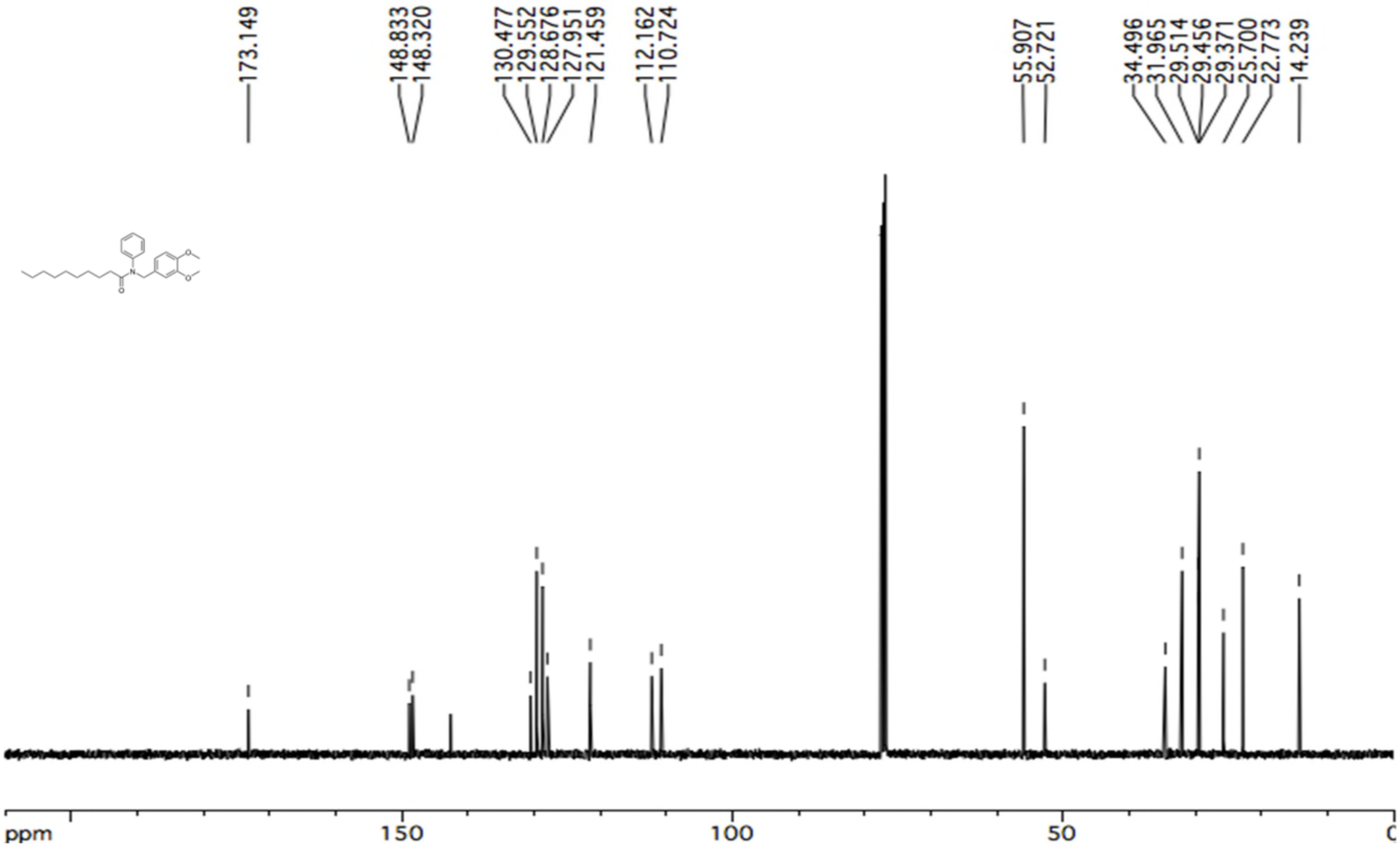

<u>*N*-[3,4-dimethoxyphenyl)methyl]-*N*-(phenyl)-decanamide (**22**) – IR</u>

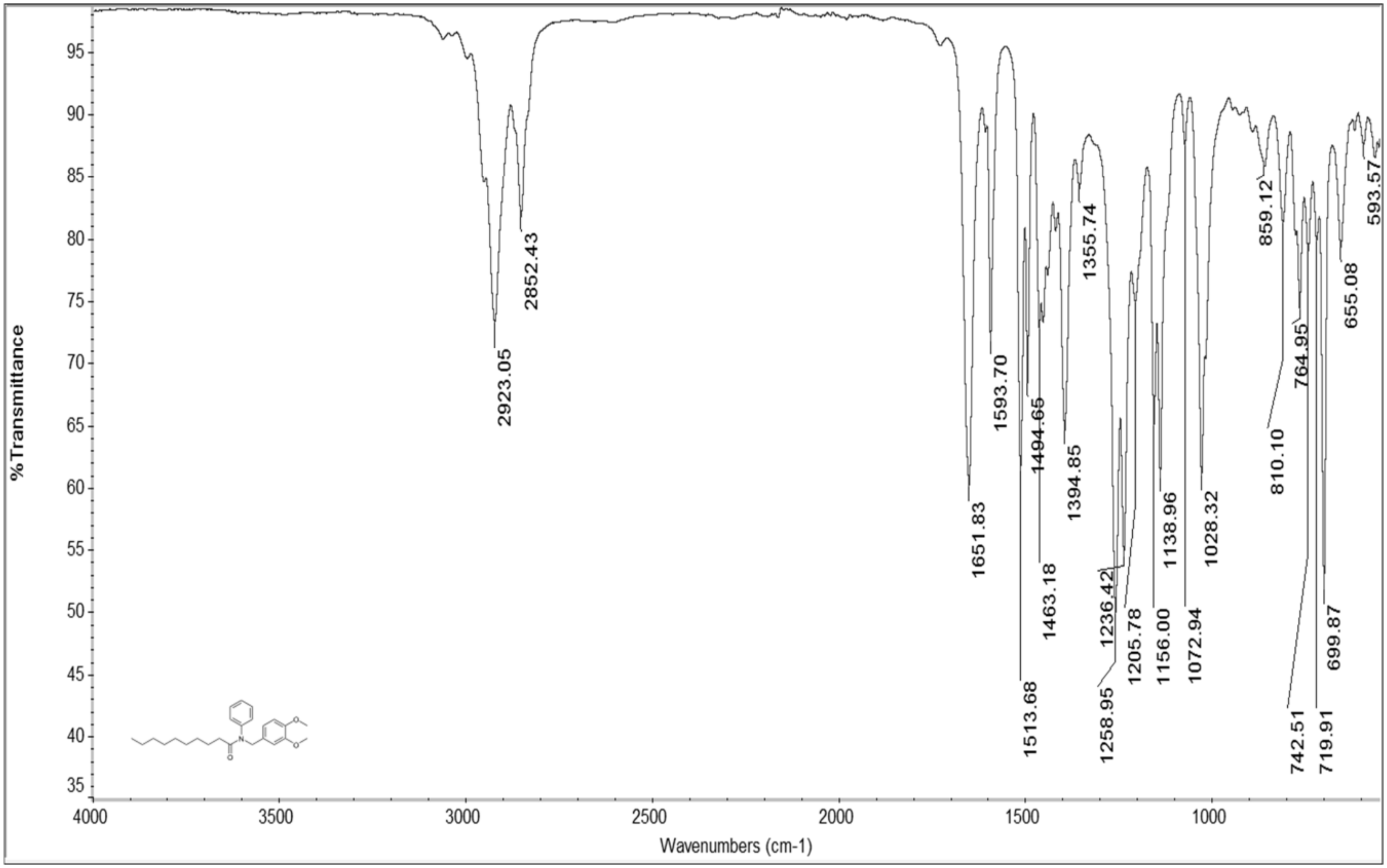

N-[3,4-dimethoxyphenyl)methyl]-*N*-(phenyl)-decanamide (**22**) – HRMS

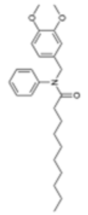

Analysis Info

Analysis Name	D:\Data\Xiao\Nov 06 2020000002.d	Acquisition Date	11/6/2020 9:57:20 AM
Method	Xiao 2.m	Operator	Administrator
Sample Name	MFL-105	Instrument	micrOTOF 57
Comment			

Acquisition Parameter

Source Type	ESI	Ion Polarity	Positive	Set Corrector Fill	45 V
Scan Range	n/a	Capillary Exit	90.0 V	Set Pulsar Pull	399 V
Scan Begin	50 m/z	Hexapole RF	125.0 V	Set Pulsar Push	399 V
Scan End	1500 m/z	Skimmer 1	40.0 V	Set Reflector	1300 V
		Hexapole 1	23.0 V	Set Flight Tube	9000 V
				Set Detector TOF	2200 V

Sum Formula	Sigma	m/z	Err [ppm]	Mean Err [ppm]	rdb	N Rule	e⁻
C 25 H 35 N 1 Na 1 O 3	0.14	420.2509	-0.05	-0.11	8.50	ok	even

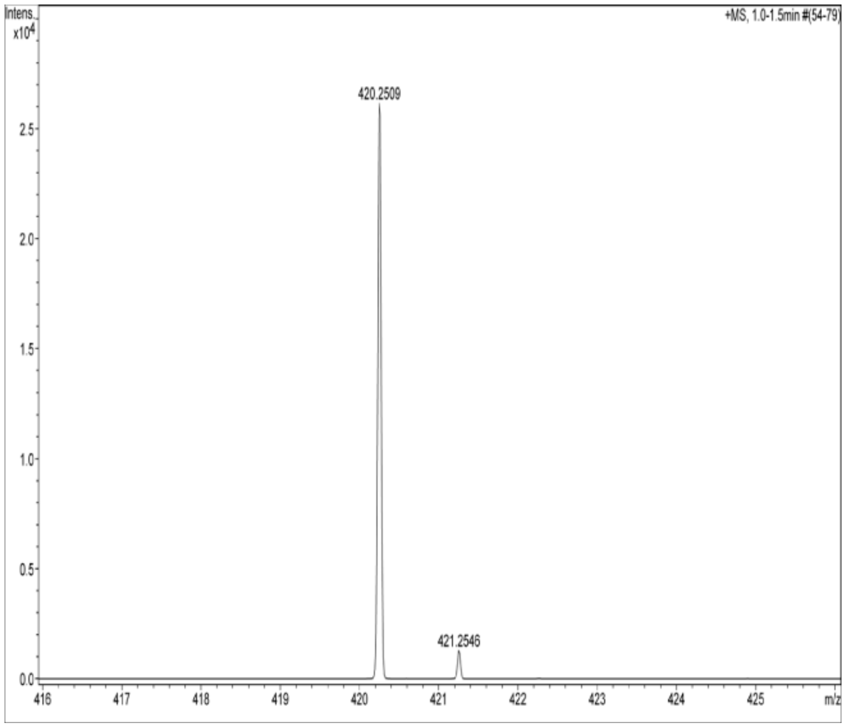

N-(3,4-dimethoxyphenylmethyl)-*N*-[4-(4,4,5,5-tetramethyl-1,3,2-dioxaborolan-2-yl)phenyl]-hexanamide (**23**) – ^{1}H NMR

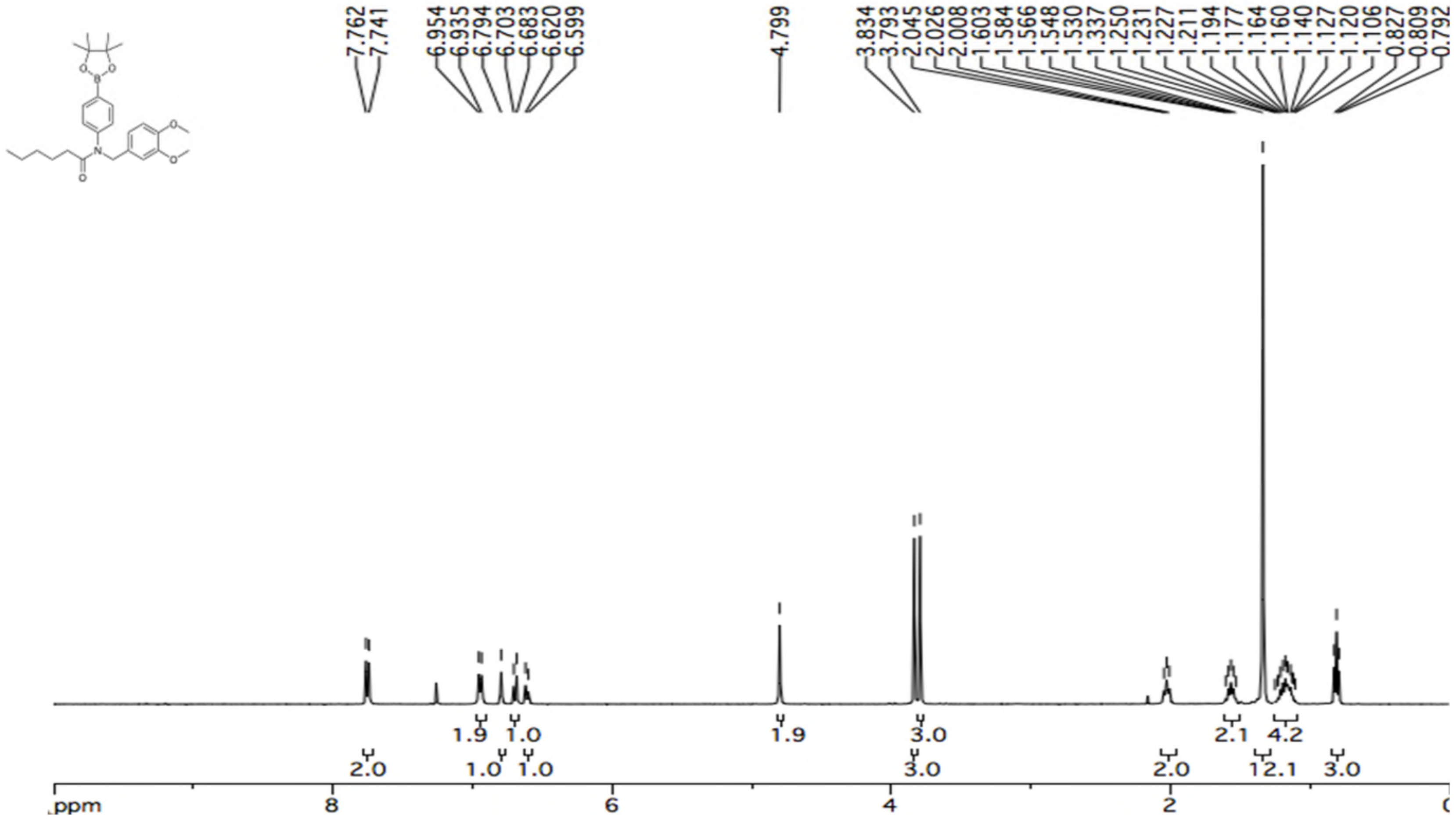

N-(3,4-dimethoxyphenylmethyl)-N-[4-(4,4,5,5-tetramethyl-1,3,2-dioxaborolan-2-yl)phenyl]-hexanamide (**23**) – ^{13}C NMR

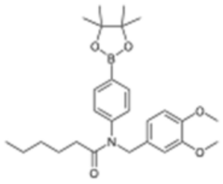

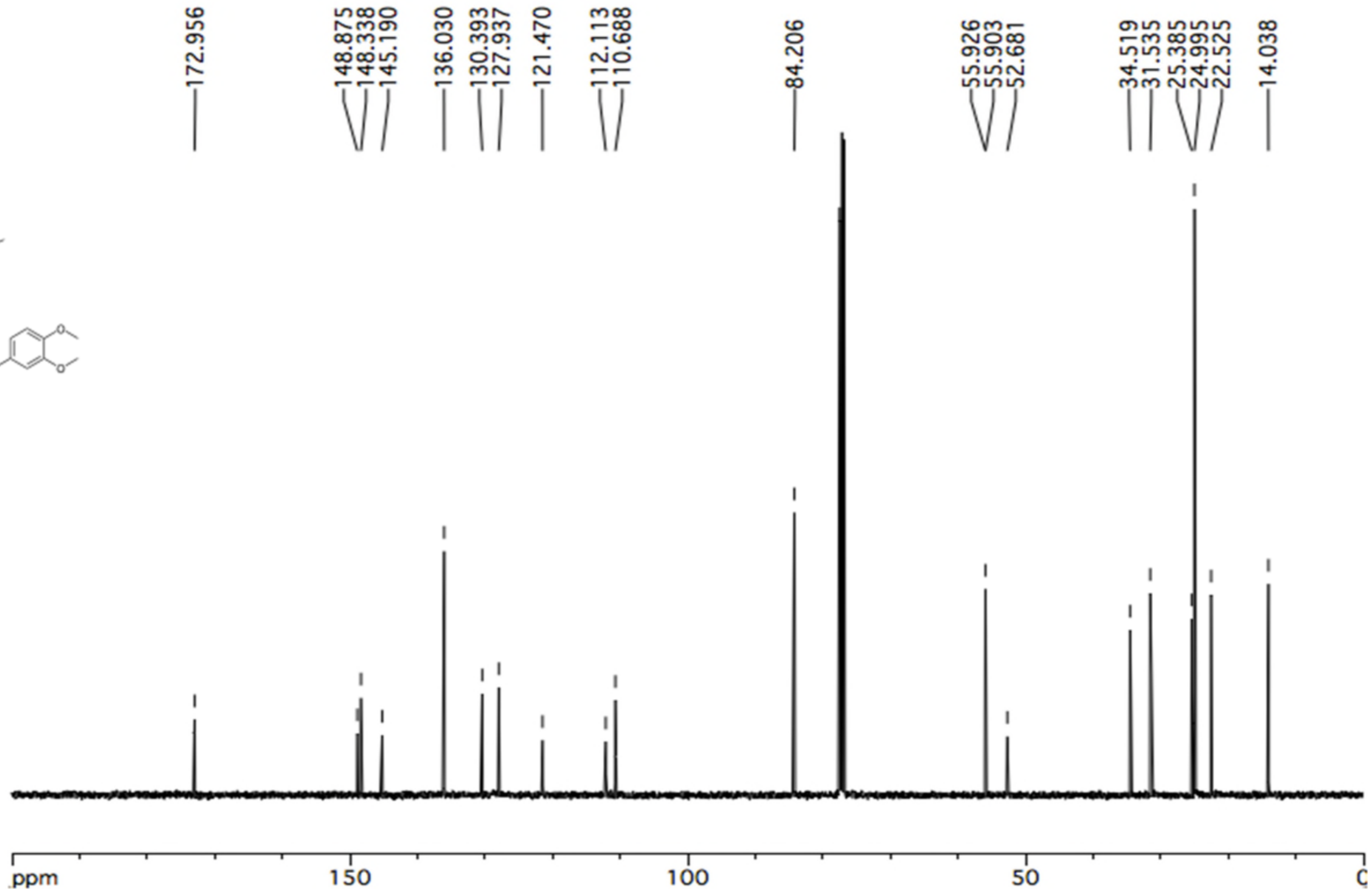

N-(3,4-dimethoxyphenylmethyl)-*N*-[4-(4,4,5,5-tetramethyl-1,3,2-dioxaborolan-2-yl)phenyl]-hexanamide (**23**) – ^{11}B NMR

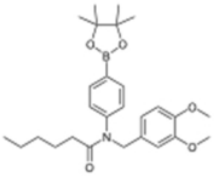

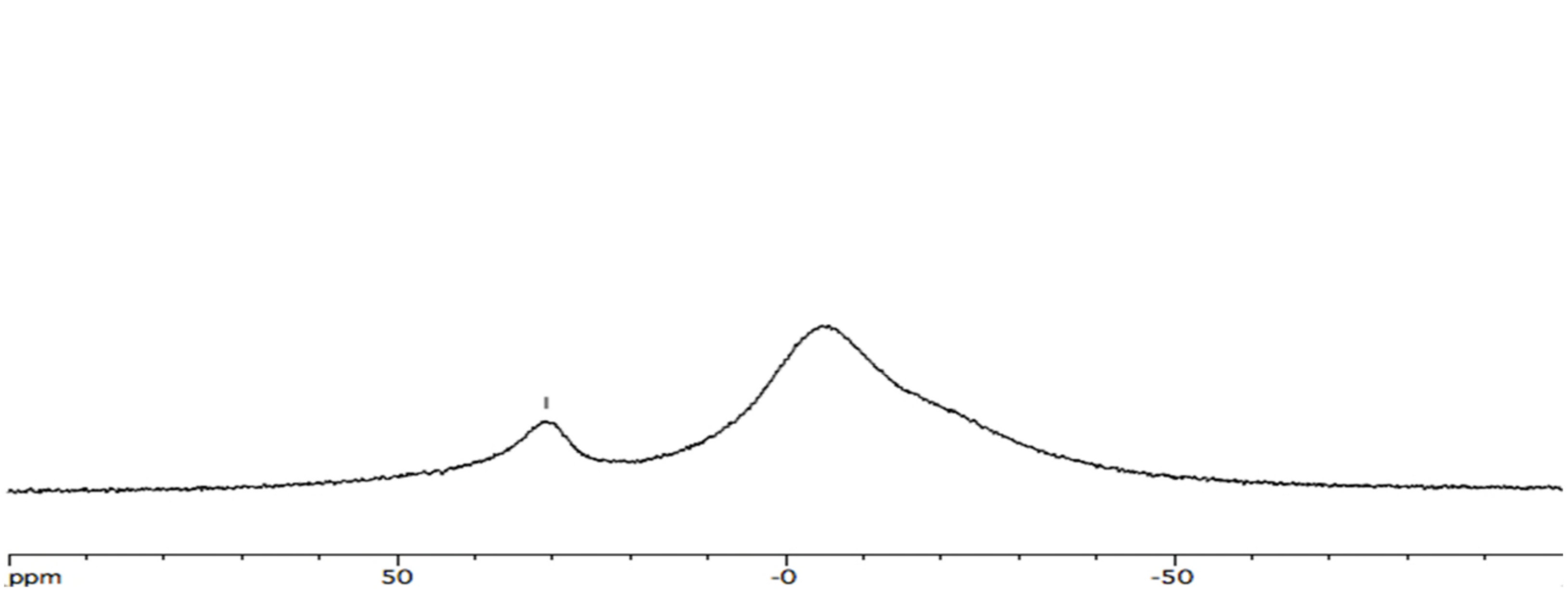

N-(3,4-dimethoxyphenylmethyl)-*N*-[4-(4,4,5,5-tetramethyl-1,3,2-dioxaborolan-2-yl)phenyl]-hexanamide (**23**) – IR

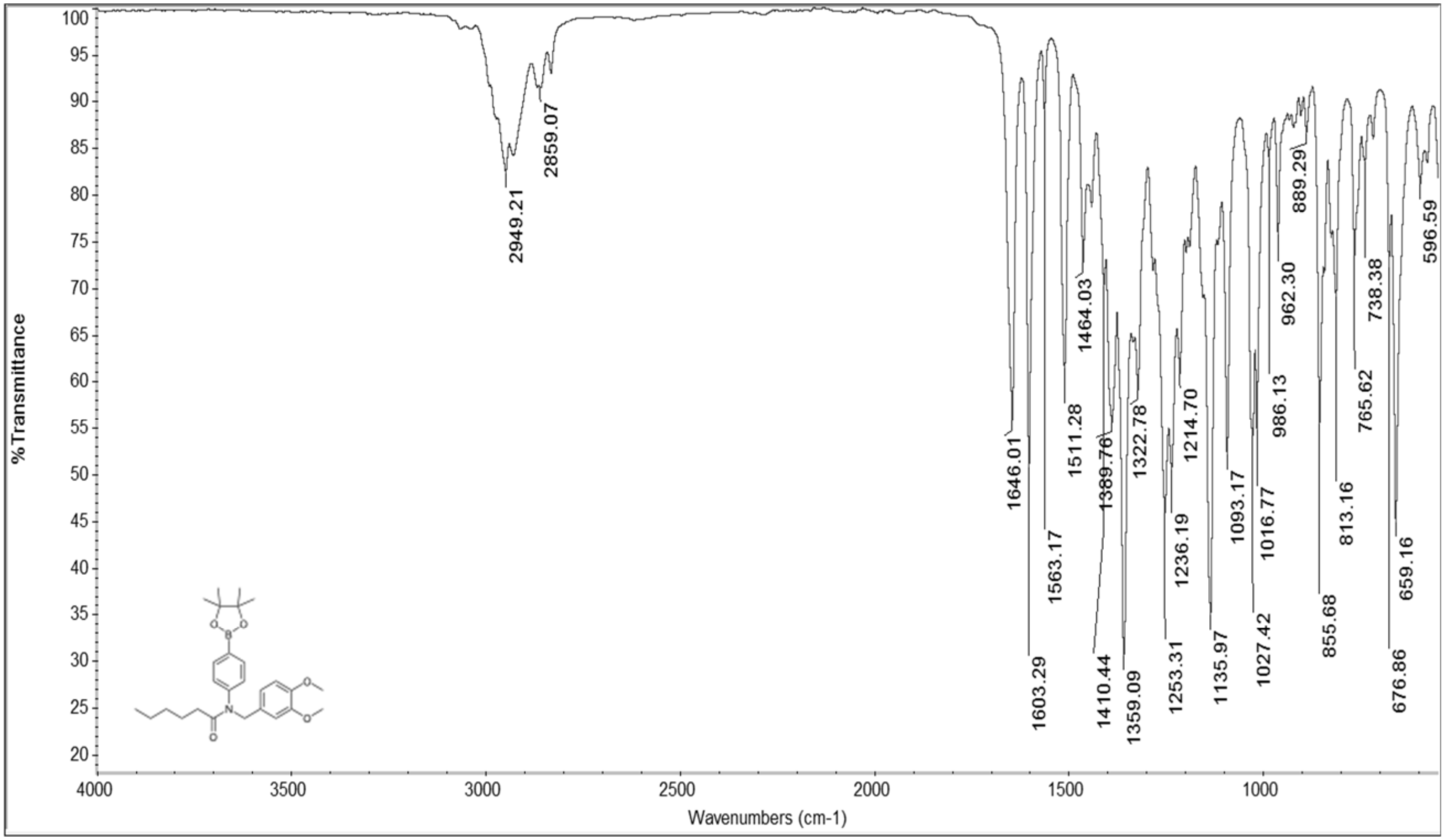

N-(3,4-dimethoxyphenylmethyl)-*N*-[4-(4,4,5,5-tetramethyl-1,3,2-dioxaborolan-2-yl)phenyl]-hexanamide (**23**) – HRMS

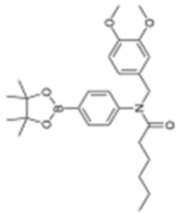

Analysis Info

Analysis Name	D:\Data\Xiao\Nov 05 2020000010.d	Acquisition Date	11/5/2020 11:21:34 AM
Method	Xiao 2.m	Operator	Administrator
Sample Name	MFL-91	Instrument	micrOTOF 57
Comment			

Acquisition Parameter

Source Type	ESI	Ion Polarity	Positive	Set Corrector Fill	45 V
Scan Range	n/a	Capillary Exit	90.0 V	Set Pulsar Pull	399 V
Scan Begin	50 m/z	Hexapole RF	125.0 V	Set Pulsar Push	399 V
Scan End	1500 m/z	Skimmer 1	40.0 V	Set Reflector	1300 V
		Hexapole 1	23.0 V	Set Flight Tube	9000 V
				Set Detector TOF	2200 V

Sum Formula	Sigma	m/z	Err [ppm]	Mean Err [ppm]	rdb	N Rule	e⁻
C 27 H 38 B 1 N 1 Na 1 O 5	0.17	490.2735	-2.31	-1.46	9.50	ok	even

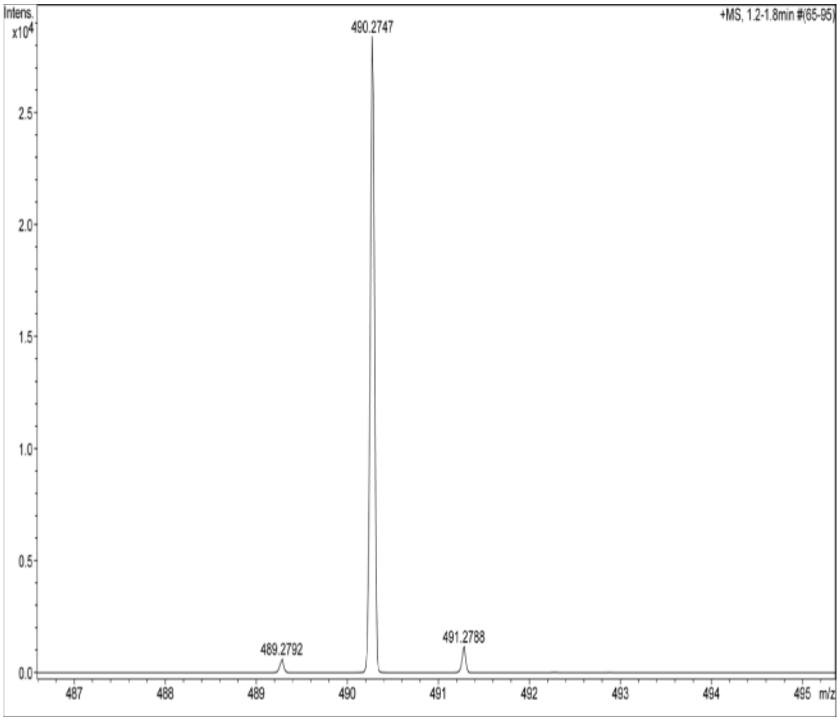

N-(3,4-dimethoxyphenylmethyl)-*N*-[4-(4,4,5,5-tetramethyl-1,3,2-dioxaborolan-2-yl)phenyl]-decanamide (**24**) – ^{1}H NMR

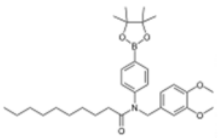

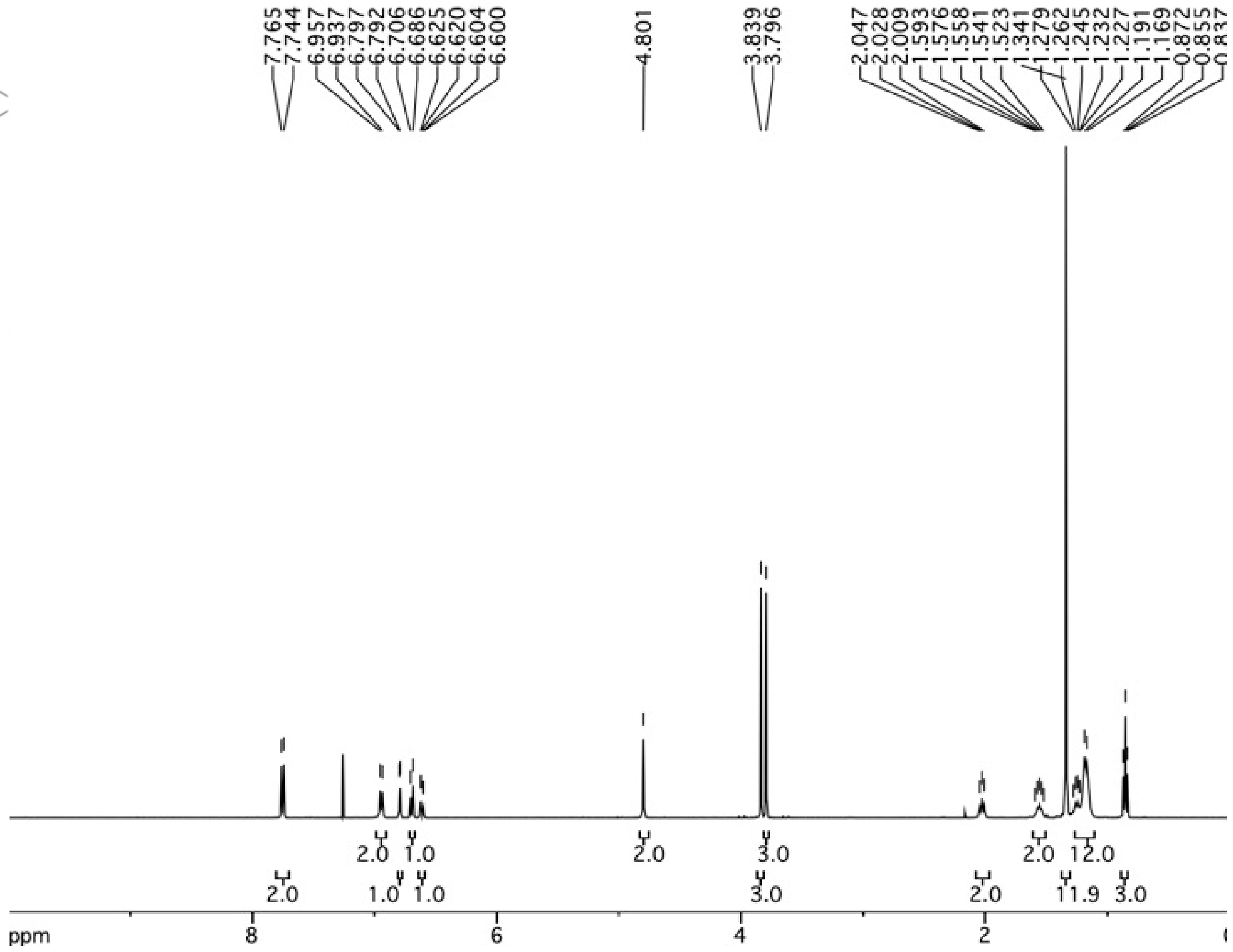

N-(3,4-dimethoxyphenylmethyl)-*N*-[4-(4,4,5,5-tetramethyl-1,3,2-dioxaborolan-2-yl)phenyl]-decanamide (**24**)– ^{13}C NMR

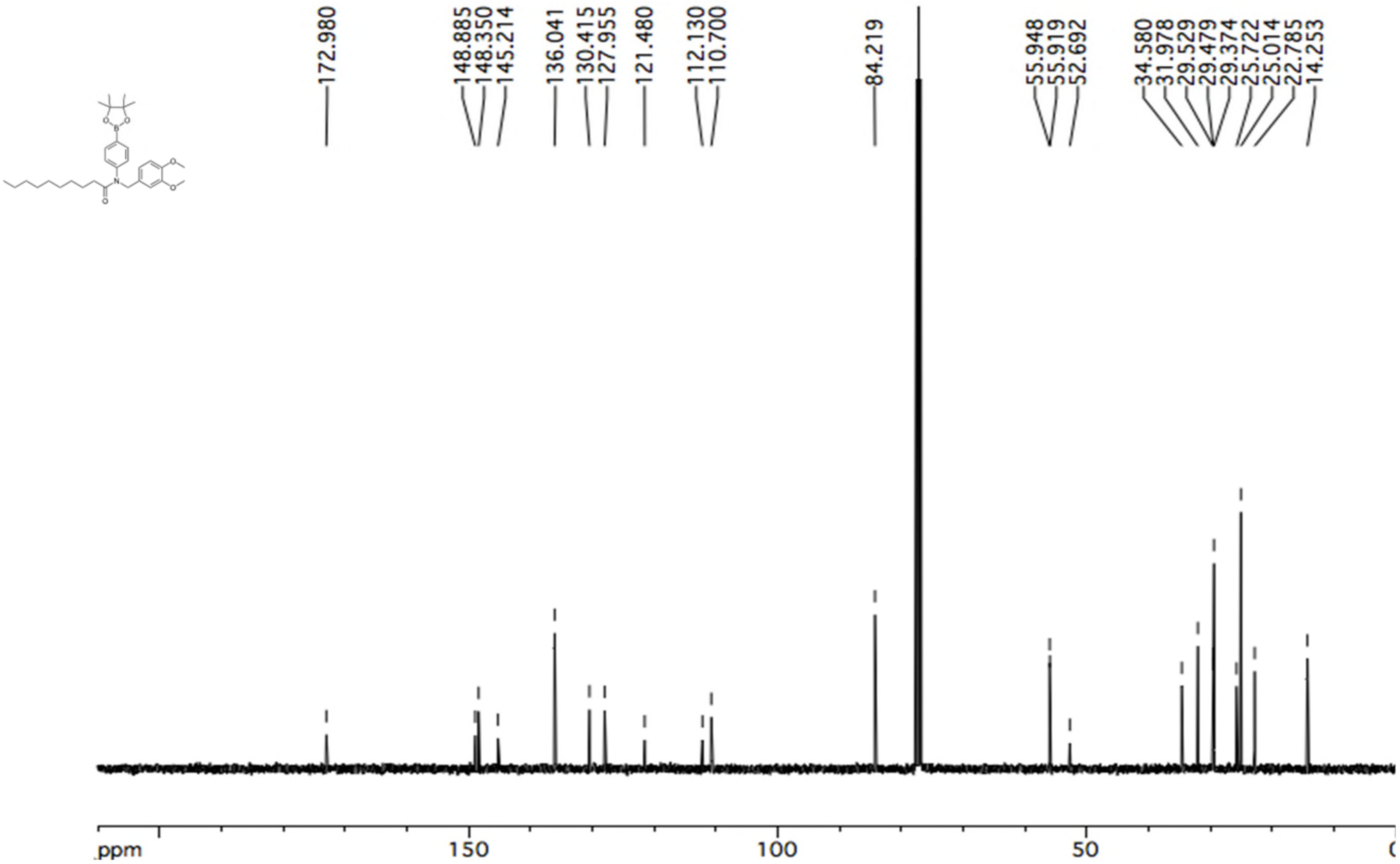

N-(3,4-dimethoxyphenylmethyl)-*N*-[4-(4,4,5,5-tetramethyl-1,3,2-dioxaborolan-2-yl)phenyl]-decanamide (**24**) – ^{11}B NMR

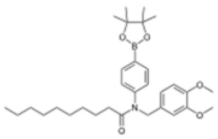

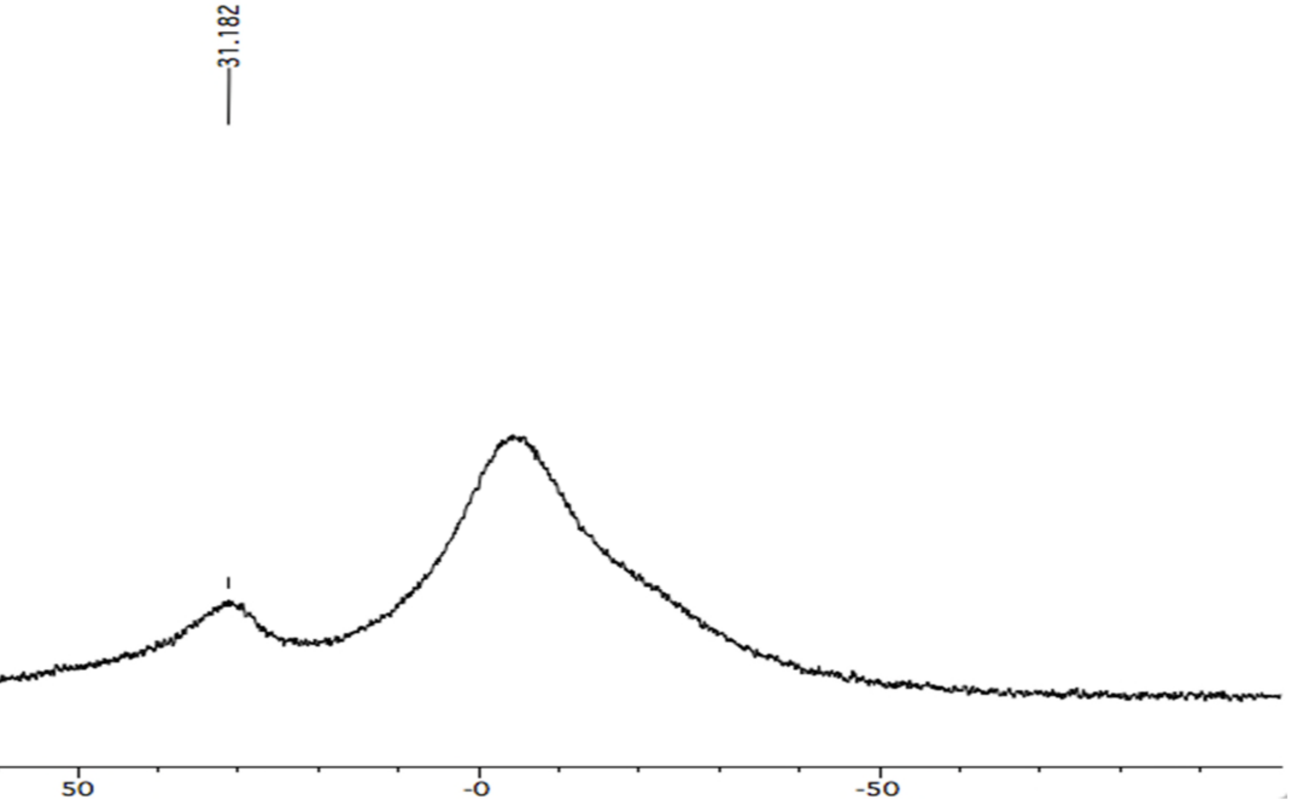

<u>*N*-(3,4-dimethoxyphenylmethyl)-*N*-[4-(4,4,5,5-tetramethyl-1,3,2-dioxaborolan-2-yl)phenyl]-decanamide (**24**) – </u>IR

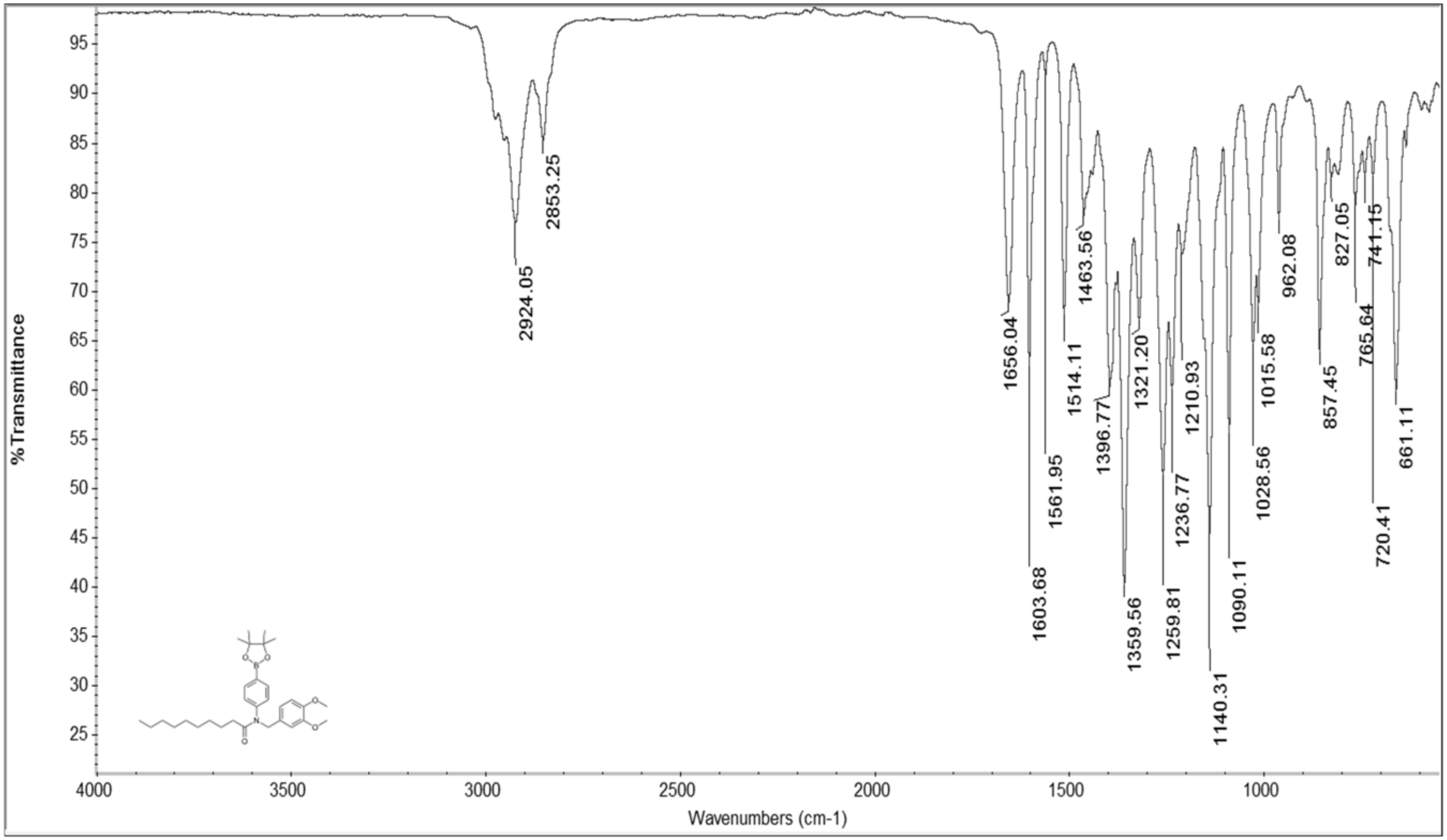

<u>*N*-(3,4-dimethoxyphenylmethyl)-*N*-[4-(4,4,5,5-tetramethyl-1,3,2-dioxaborolan-2-yl)phenyl]-decanamide (**24**) – HRMS</u>

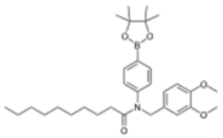

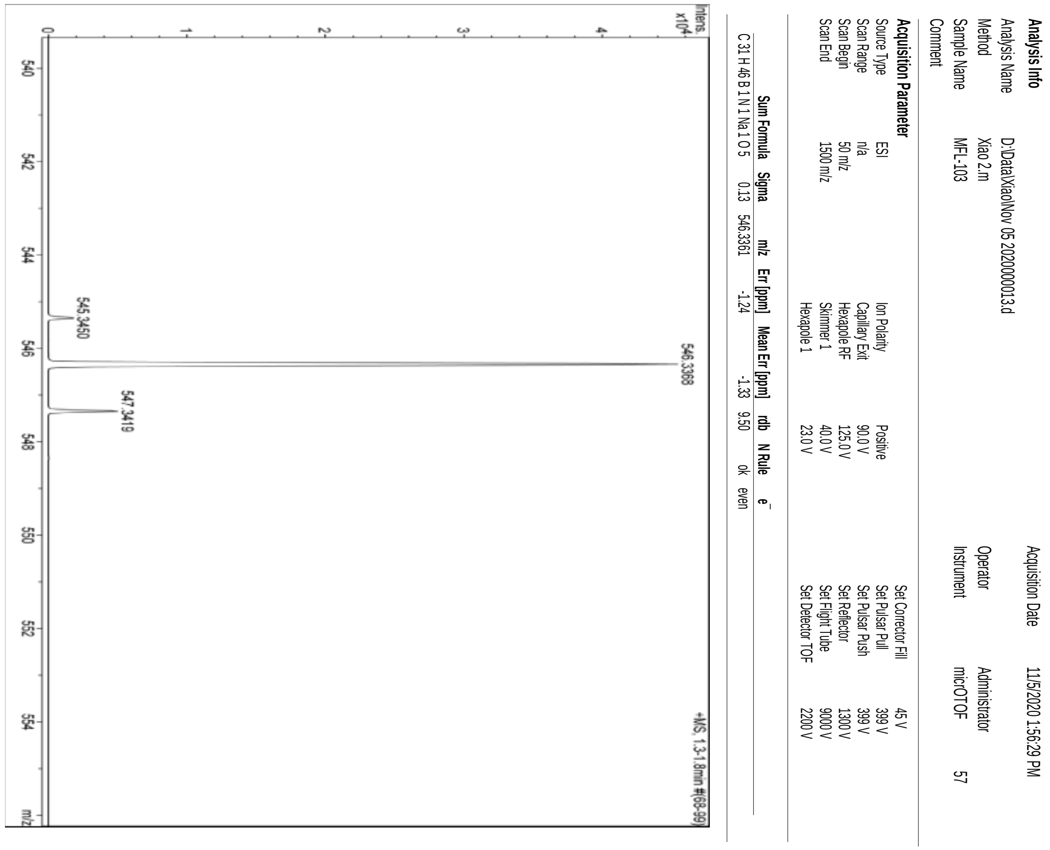

N-(3,4-dimethoxyphenylmethyl)-*N*-[3-(4,4,5,5-tetramethyl-1,3,2-dioxaborolan-2-yl)phenyl]-hexanamide (**25**) – ^{1}H NMR

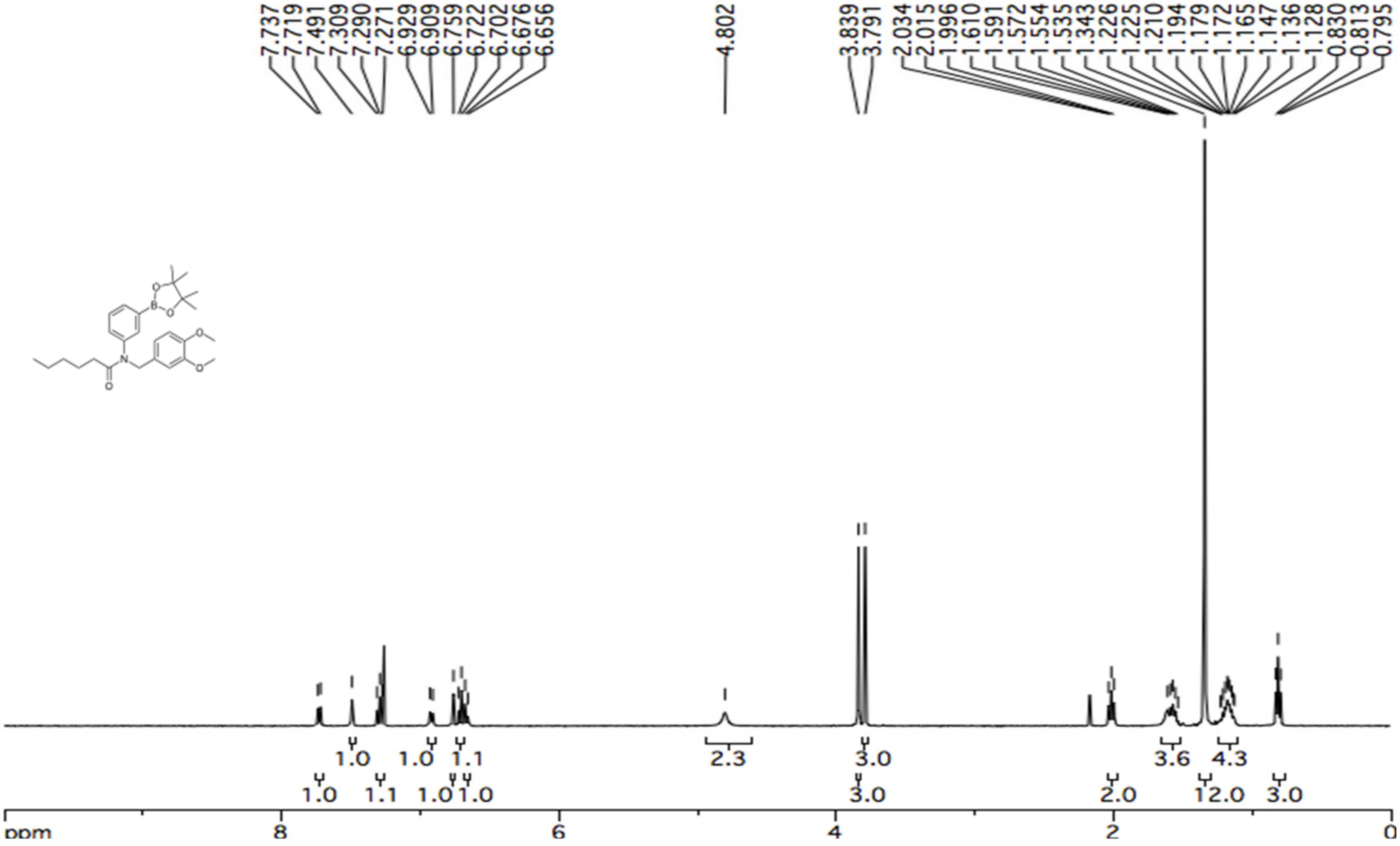

N-(3,4-dimethoxyphenylmethyl)-*N*-[3-(4,4,5,5-tetramethyl-1,3,2-dioxaborolan-2-yl)phenyl]-hexanamide (**25**) – ^{13}C NMR

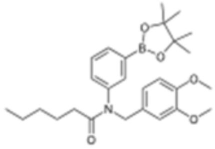

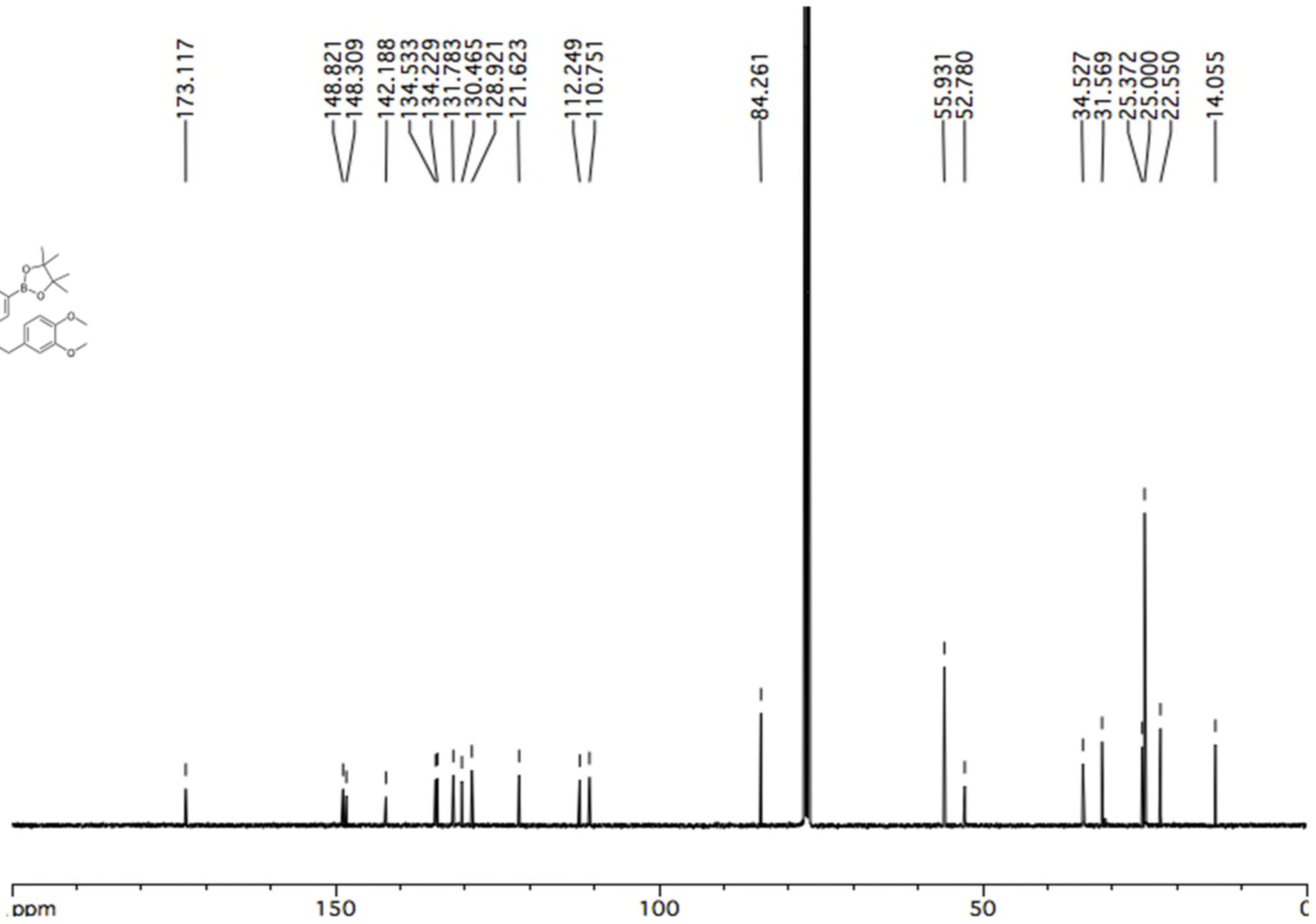

N-(3,4-dimethoxyphenylmethyl)-*N*-[3-(4,4,5,5-tetramethyl-1,3,2-dioxaborolan-2-yl)phenyl]-hexanamide (**25**) – ^{11}B NMR

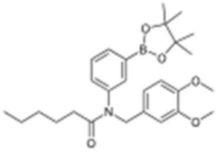

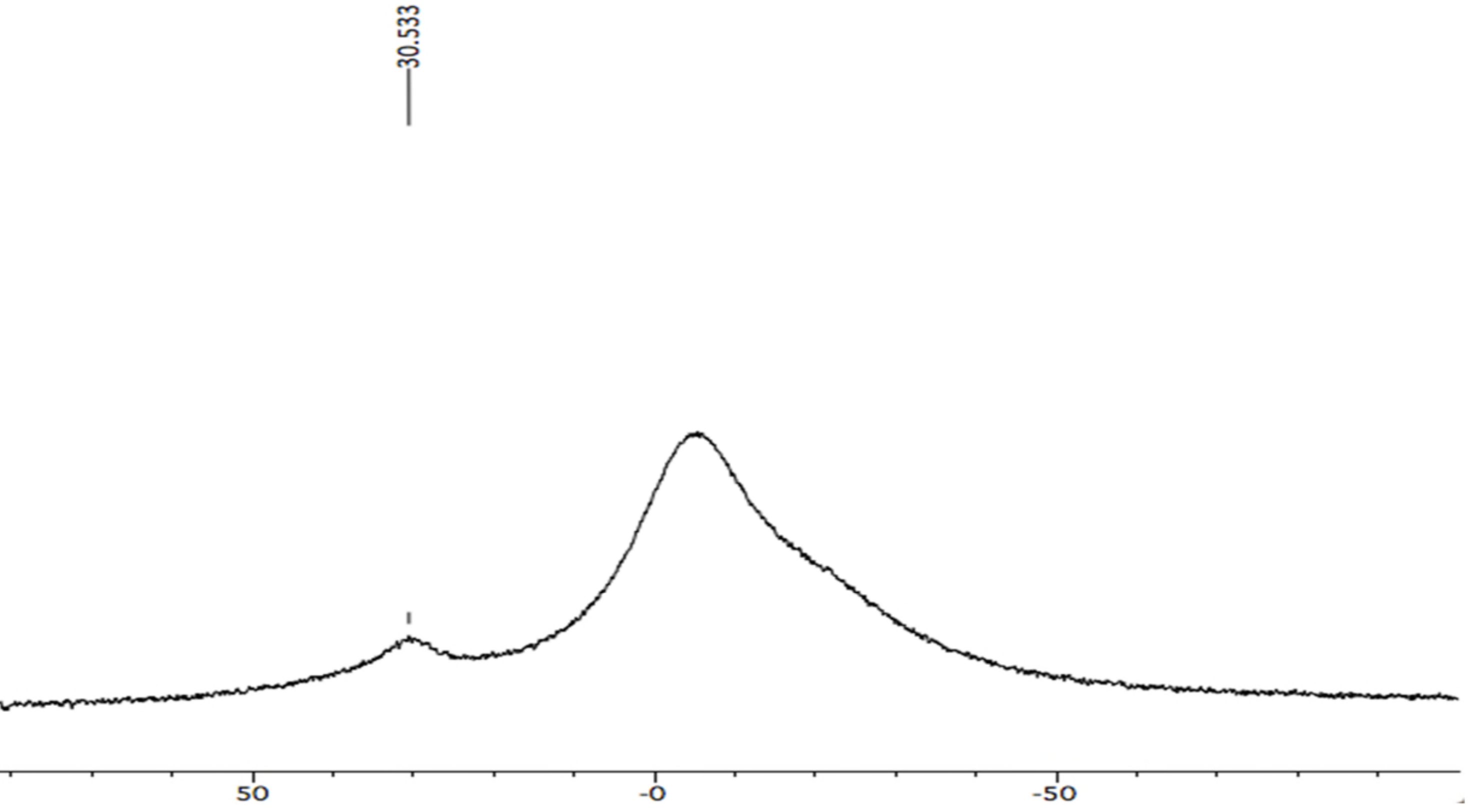

<u>*N*-(3,4-dimethoxyphenylmethyl)-*N*-[3-(4,4,5,5-tetramethyl-1,3,2-dioxaborolan-2-yl)phenyl]-hexanamide (**25**) – IR</u>

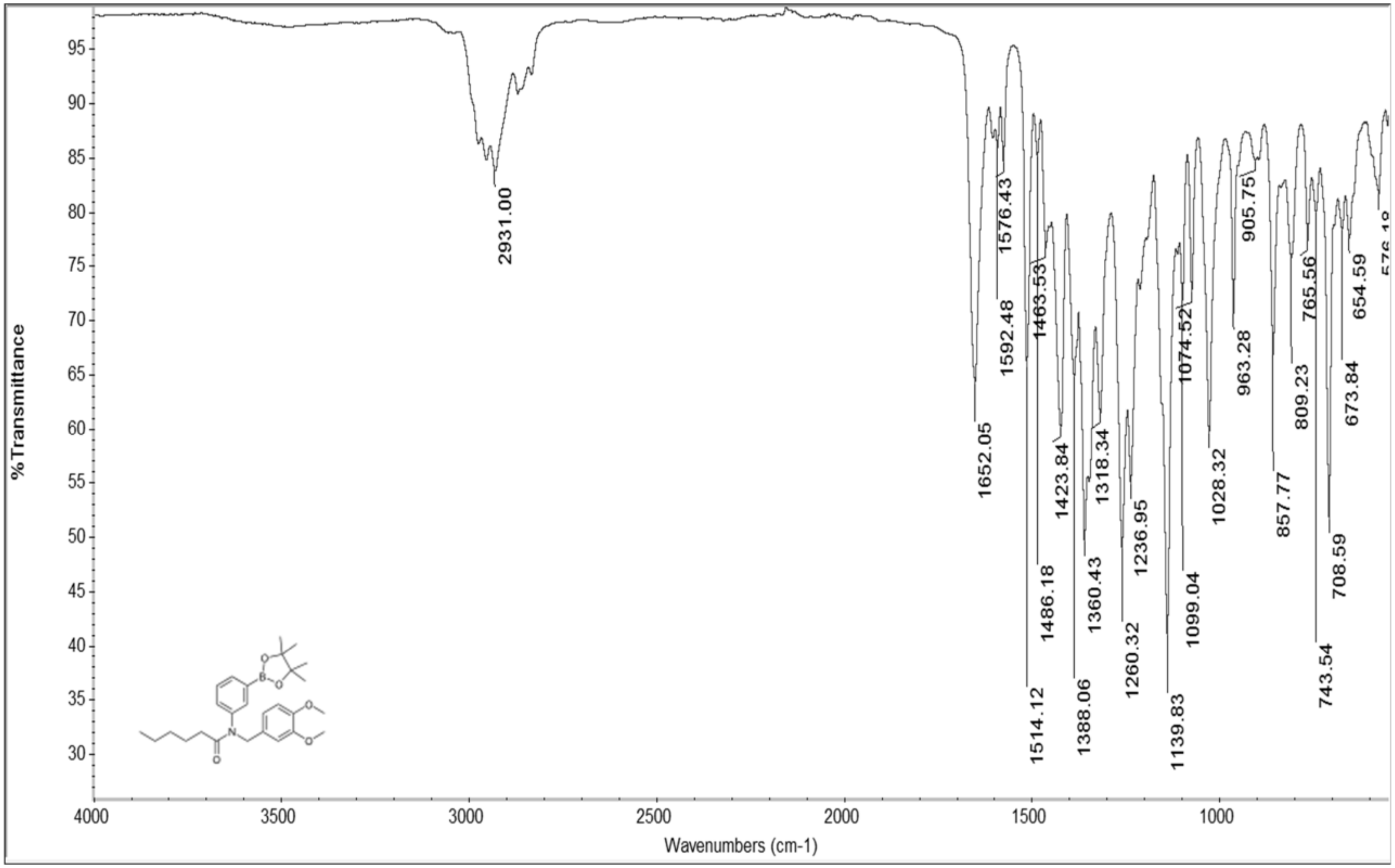

N-(3,4-dimethoxyphenylmethyl)-*N*-[3-(4,4,5,5-tetramethyl-1,3,2-dioxaborolan-2-yl)phenyl]-hexanamide (**25**) – HRMS

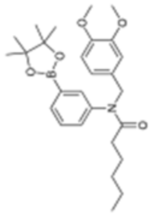

Analysis Info

Analysis Name	D:\Data\Xiao\Nov 05 2020000011.d	Acquisition Date	11/5/2020 11:31:10 AM
Method	Xiao 2.m	Operator	Administrator
Sample Name	MFL-98	Instrument	micrOTOF 57
Comment			

Acquisition Parameter

Source Type	ESI	Ion Polarity	Positive	Set Corrector Fill	45 V
Scan Range	n/a	Capillary Exit	90.0 V	Set Pulsar Pull	399 V
Scan Begin	50 m/z	Hexapole RF	125.0 V	Set Pulsar Push	399 V
Scan End	1500 m/z	Skimmer 1	40.0 V	Set Reflector	1300 V
		Hexapole 1	23.0 V	Set Flight Tube	9000 V
				Set Detector TOF	2200 V

Sum Formula	Sigma	m/z	Err [ppm]	Mean Err [ppm]	rdb	N Rule	e⁻
C 27 H 38 B 1 N 1 Na 1 O 5	0.15	490.2735	-2.93	-2.17	9.50	ok	even

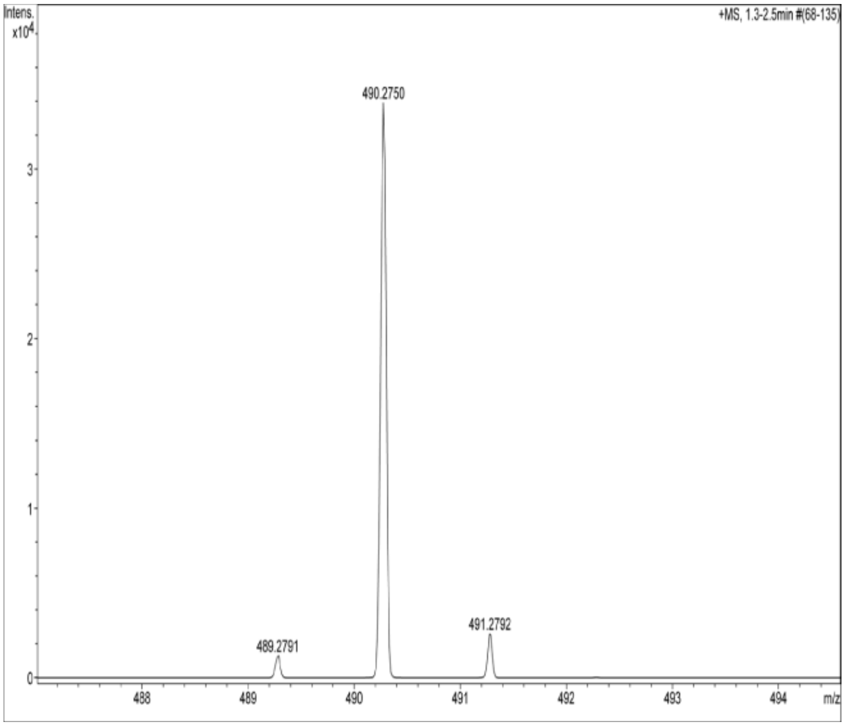

N-(3,4-dimethoxyphenylmethyl)-*N*-[3-(4,4,5,5-tetramethyl-1,3,2-dioxaborolan-2-yl)phenyl]-decanamide (**26**) – ^{1}H NMR

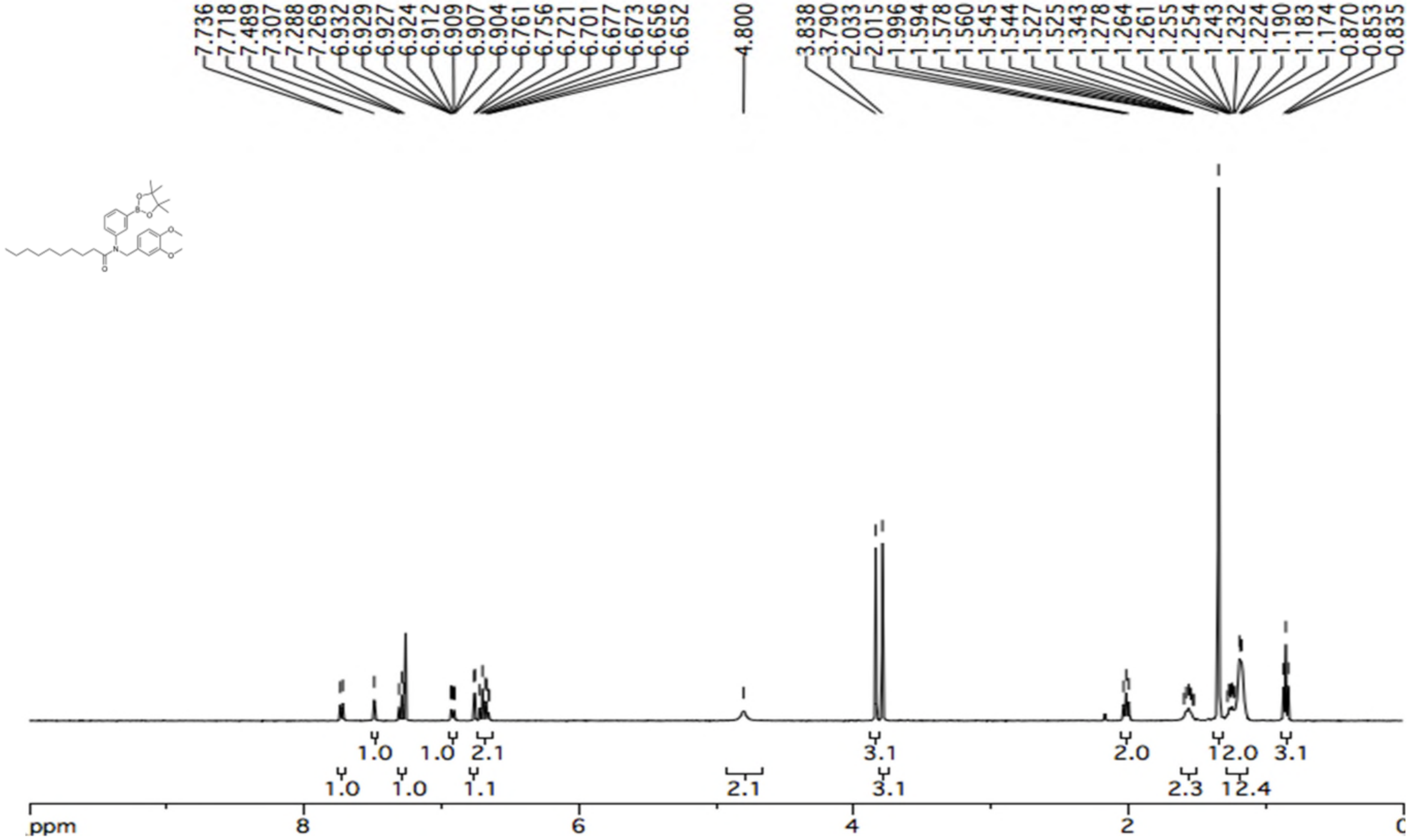

N-(3,4-dimethoxyphenylmethyl)-*N*-[3-(4,4,5,5-tetramethyl-1,3,2-dioxaborolan-2-yl)phenyl]-decanamide (**26**) – ^{13}C NMR

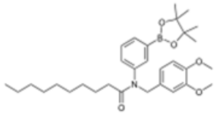

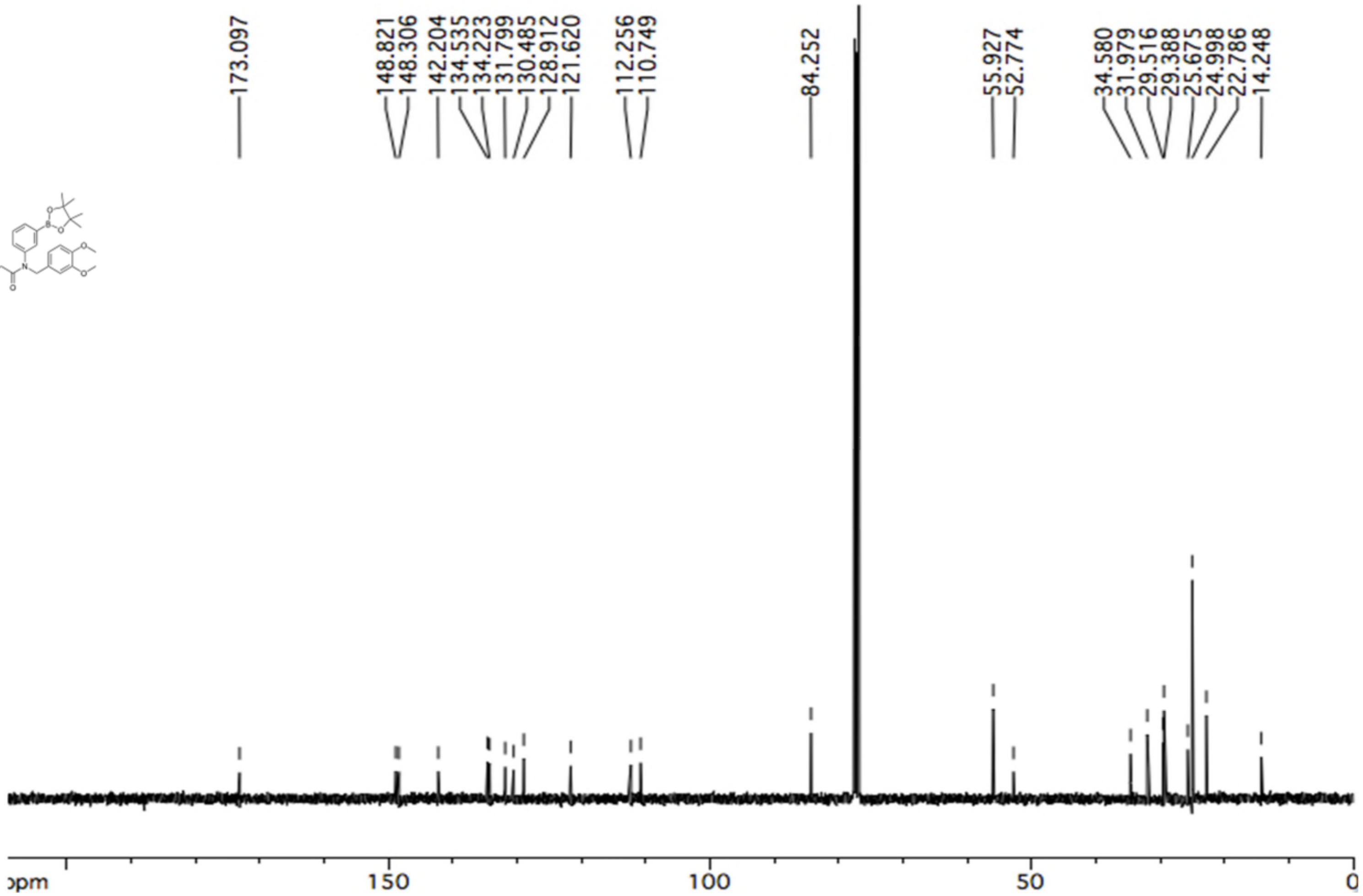

N-(3,4-dimethoxyphenylmethyl)-*N*-[3-(4,4,5,5-tetramethyl-1,3,2-dioxaborolan-2-yl)phenyl]-decanamide (**26**) – ^{11}B NMR

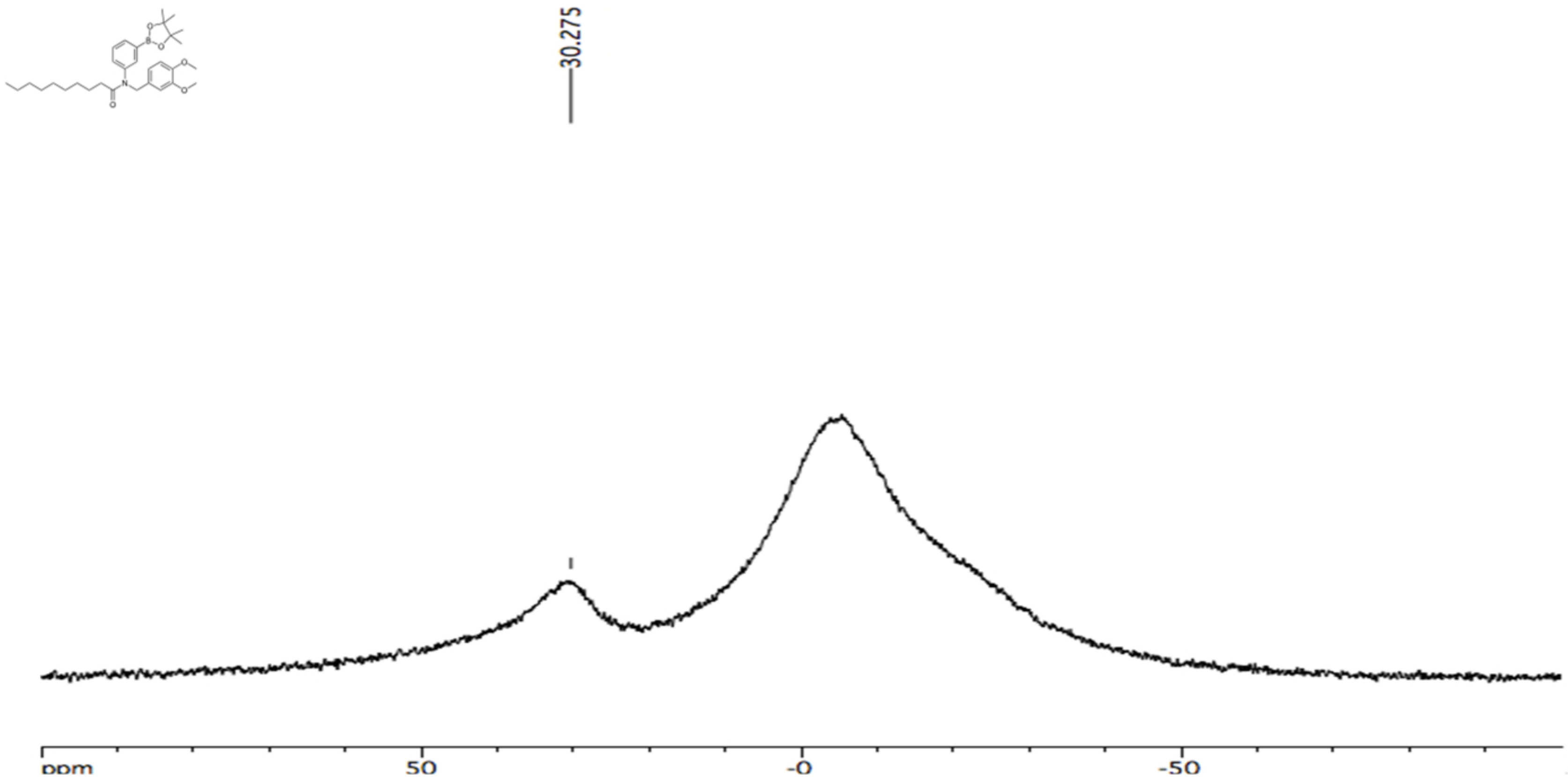

N-(3,4-dimethoxyphenylmethyl)-N-[3-(4,4,5,5-tetramethyl-1,3,2-dioxaborolan-2-yl)phenyl]-decanamide (**26**) – IR

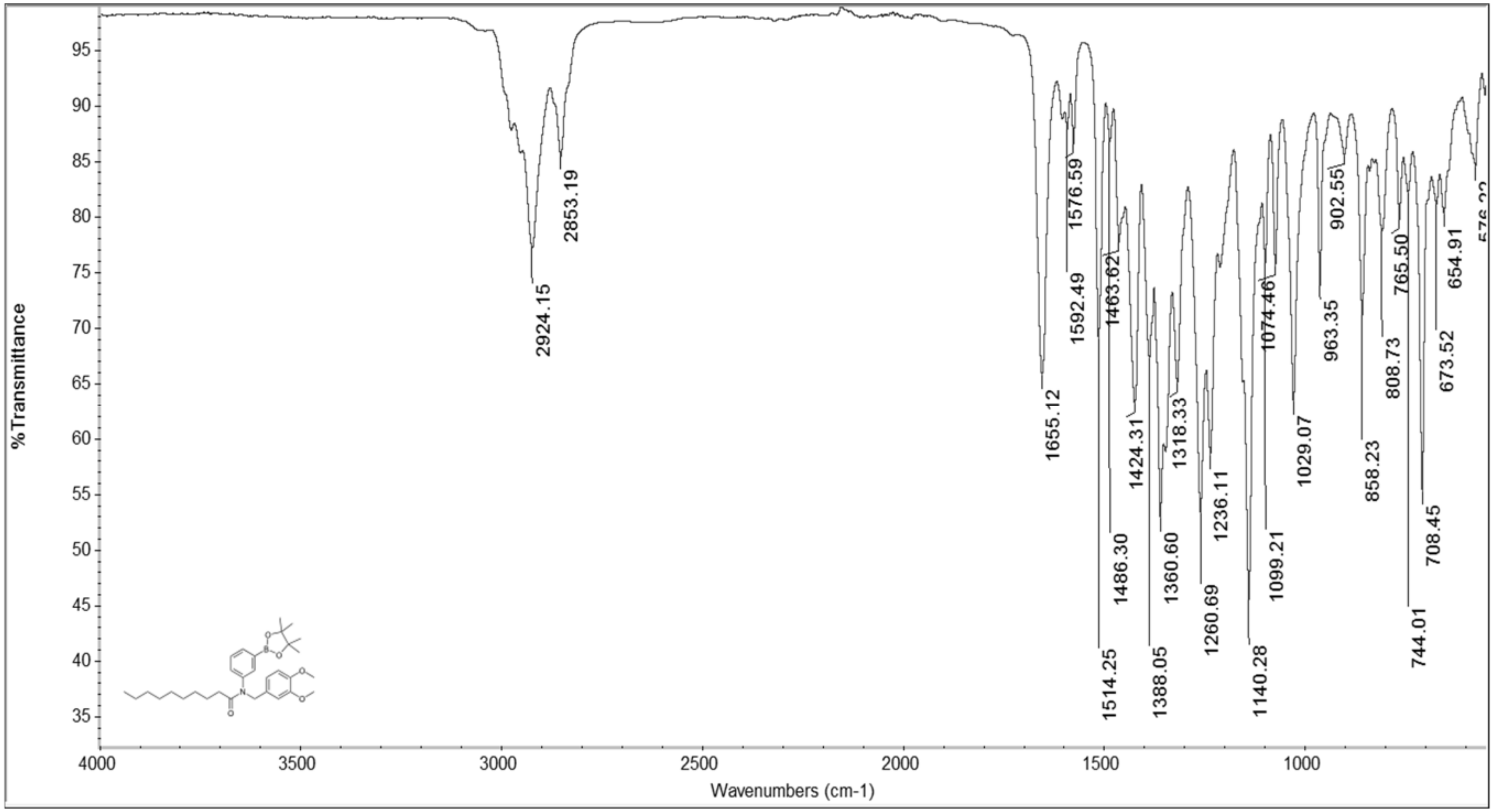

<u>*N*-(3,4-dimethoxyphenylmethyl)-*N*-[3-(4,4,5,5-tetramethyl-1,3,2-dioxaborolan-2-yl)phenyl]-decanamide (**26**) – HRMS</u>

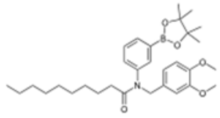

Analysis Info

Analysis Name	D:\Data\Xiao\Nov 05 2020000014.d	Acquisition Date	11/5/2020 2:06:14 PM
Method	Xiao 2.m	Operator	Administrator
Sample Name	MFL-102	Instrument	micrOTOF 57
Comment			

Acquisition Parameter

Source Type	ESI	Ion Polarity	Positive	Set Corrector Fill	45 V
Scan Range	n/a	Capillary Exit	90.0 V	Set Pulsar Pull	399 V
Scan Begin	50 m/z	Hexapole RF	125.0 V	Set Pulsar Push	399 V
Scan End	1500 m/z	Skimmer 1	40.0 V	Set Reflector	1300 V
		Hexapole 1	23.0 V	Set Flight Tube	9000 V
				Set Detector TOF	2200 V

Sum Formula	Sigma	m/z	Err [ppm]	Mean Err [ppm]	rdb	N Rule	e⁻
C 31 H 46 B 1 N 1 Na 1 O 5	0.13	546.3361	-0.85	0.99	9.50	ok	even

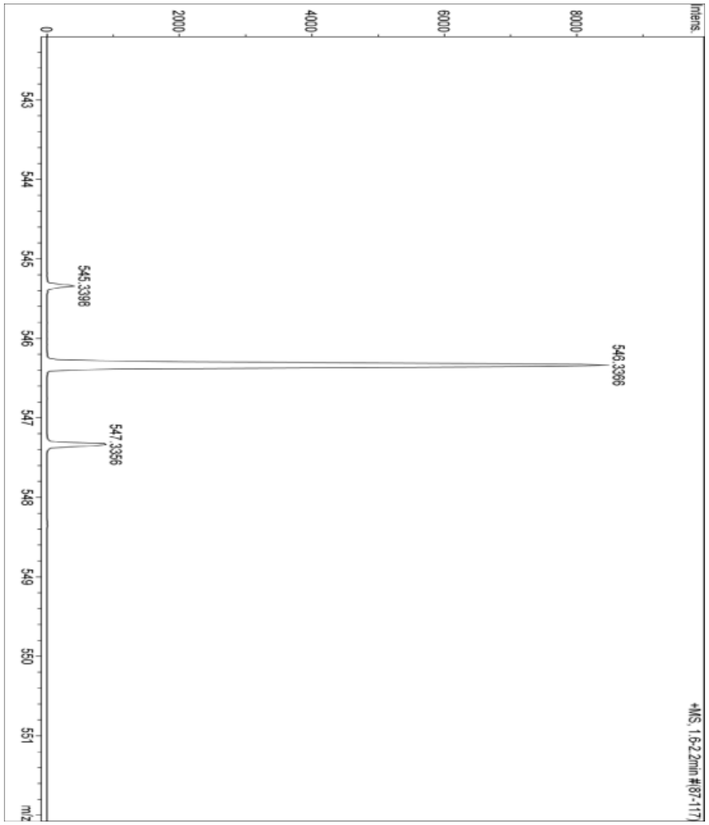

N-(3,4-dimethoxybenzyl)-*N*-phenylpent-4-enamide (**27**) – ^{1}H NMR

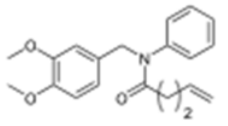

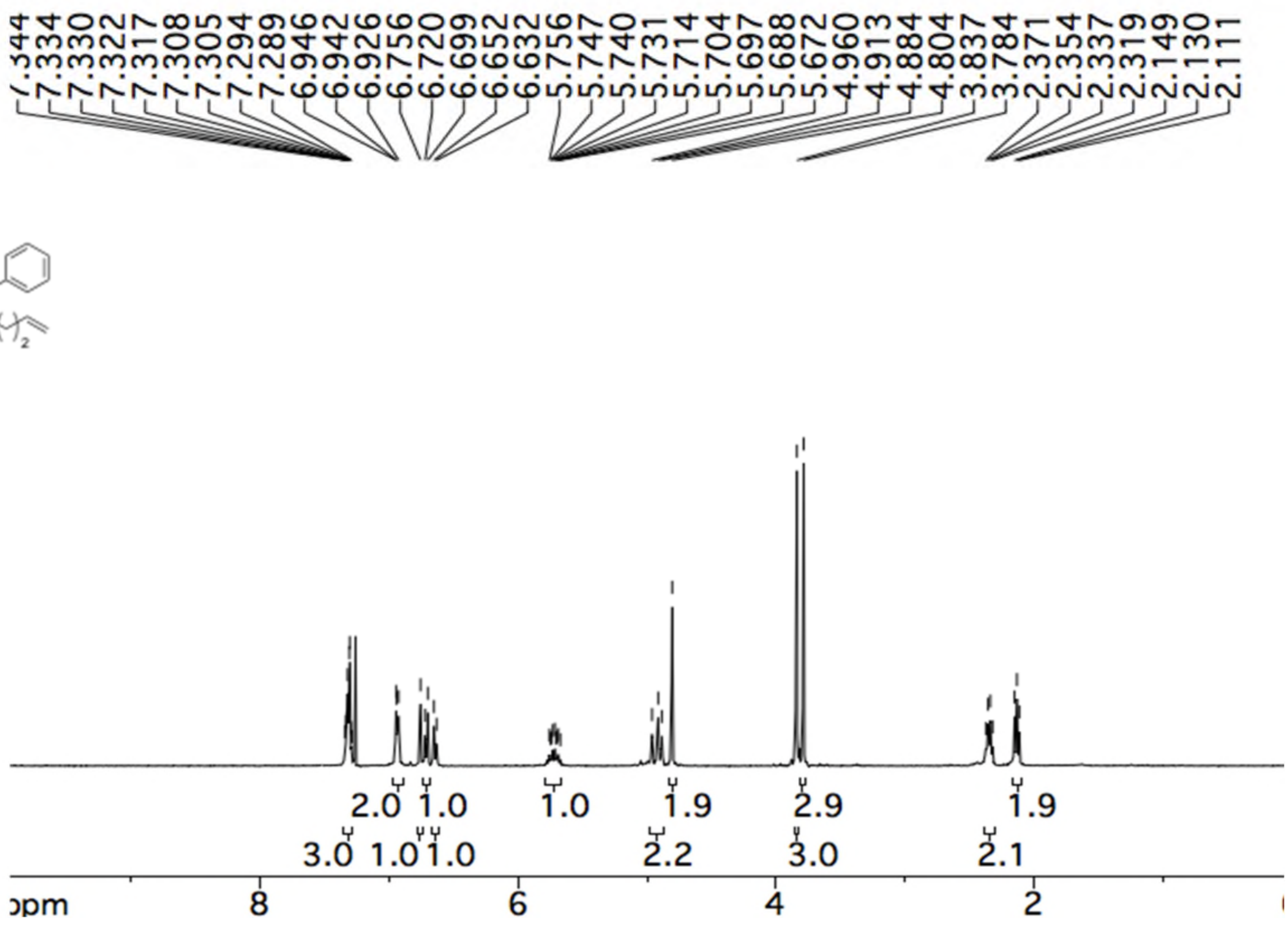

N-(3,4-dimethoxybenzyl)-N-phenylpent-4-enamide (**27**) – ^{13}C NMR

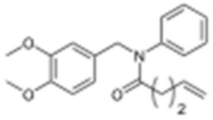

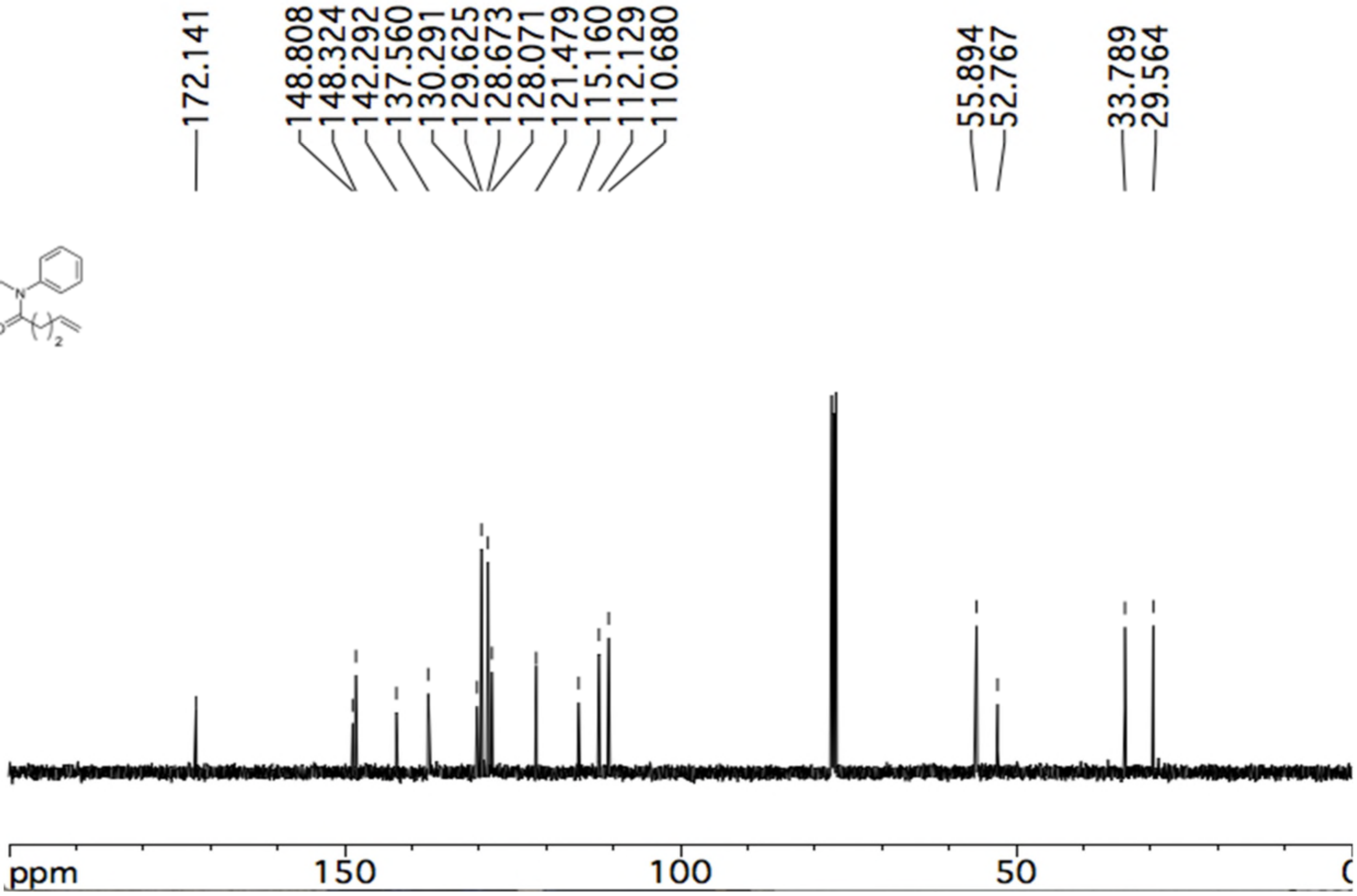

N-(3,4-dimethoxybenzyl)-*N*-phenylpent-4-enamide (**27**) – IR

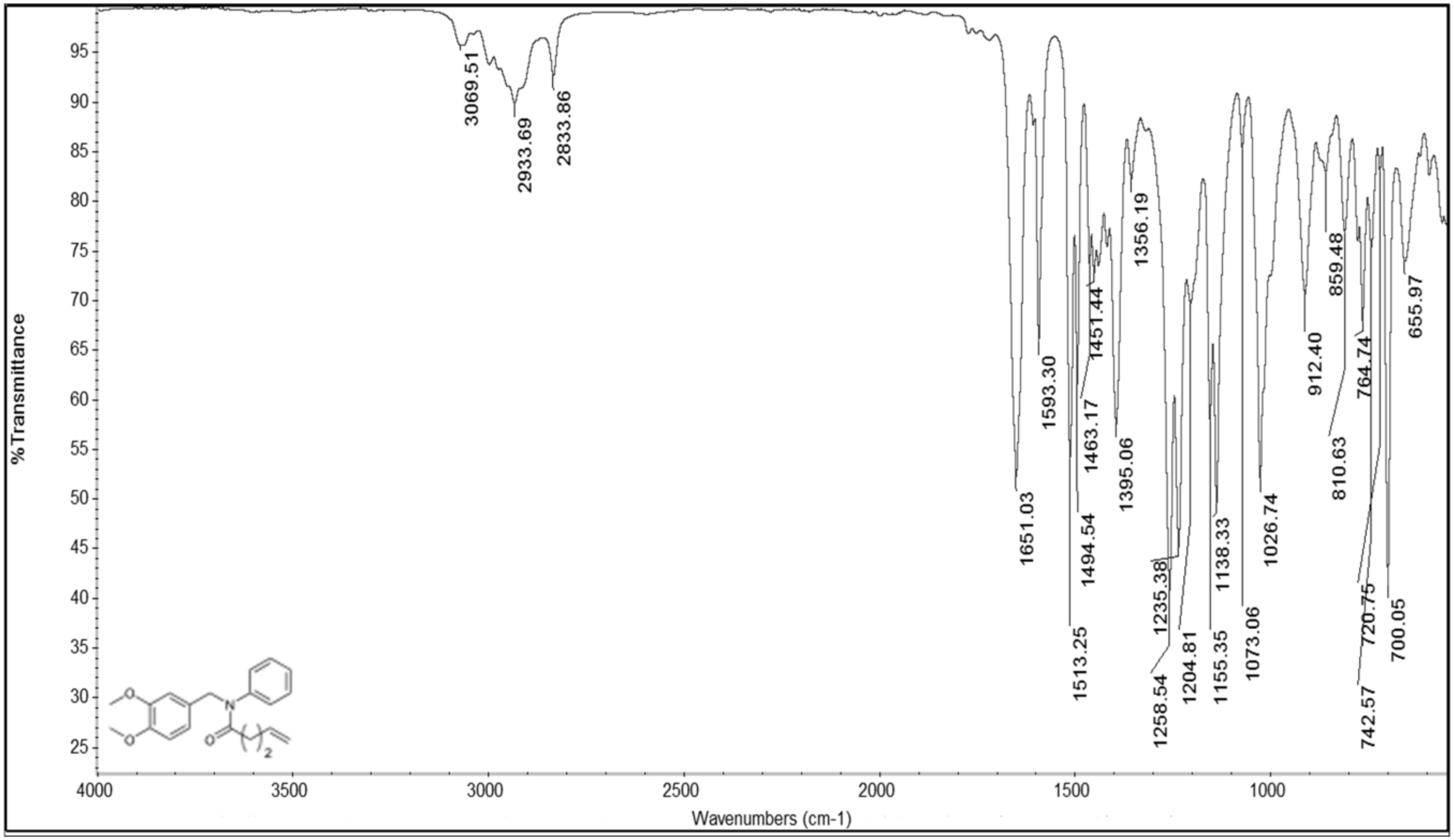

N-(3,4-dimethoxybenzyl)-*N*-phenylpent-4-enamide (**27**) – HRMS

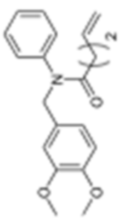

Analysis Info

Analysis Name	D:\Data\Xiao\Nov 05 2020000012.d	Acquisition Date	11/5/2020 1:44:11 PM
Method	Xiao 2.m	Operator	Administrator
Sample Name	JM-34	Instrument	micrOTOF 57
Comment			

Acquisition Parameter

Source Type	ESI	Ion Polarity	Positive	Set Corrector Fill	45 V
Scan Range	n/a	Capillary Exit	90.0 V	Set Pulsar Pull	399 V
Scan Begin	50 m/z	Hexapole RF	125.0 V	Set Pulsar Push	399 V
Scan End	1500 m/z	Skimmer 1	40.0 V	Set Reflector	1300 V
		Hexapole 1	23.0 V	Set Flight Tube	9000 V
				Set Detector TOF	2200 V

Sum Formula	Sigma	m/z	Err [ppm]	Mean Err [ppm]	rdb	N Rule	e⁻
C 20 H 23 N 1 Na 1 O 3	0.12	348.1570	-1.31	-1.35	9.50	ok	even

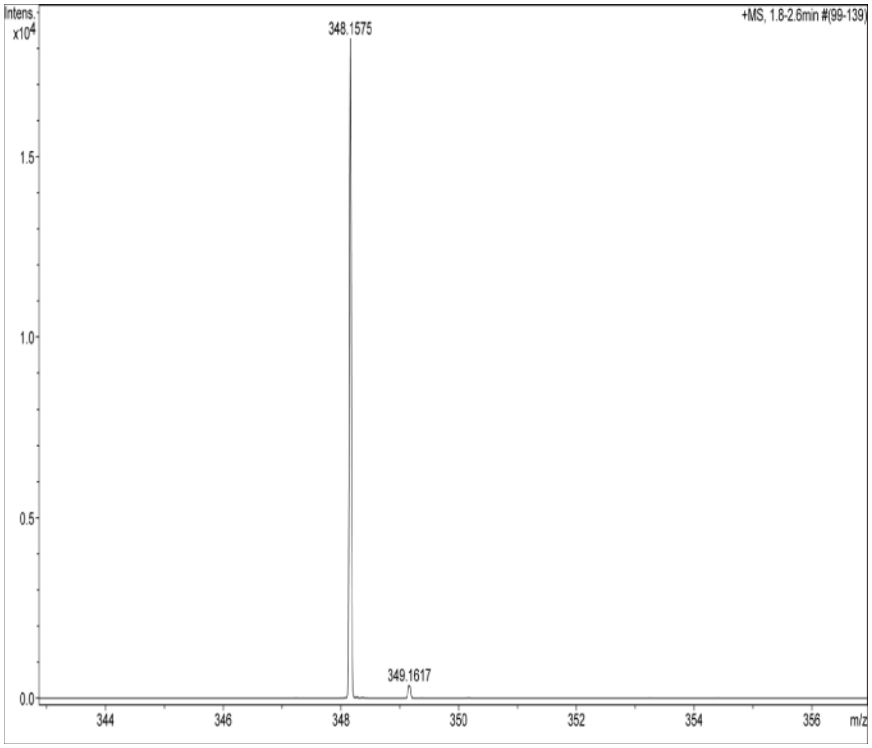

N-(3,4-dimethoxybenzyl)-*N*-phenylnon-8-enamide (**28**) – ^{1}H NMR

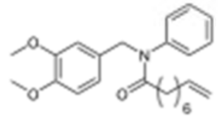

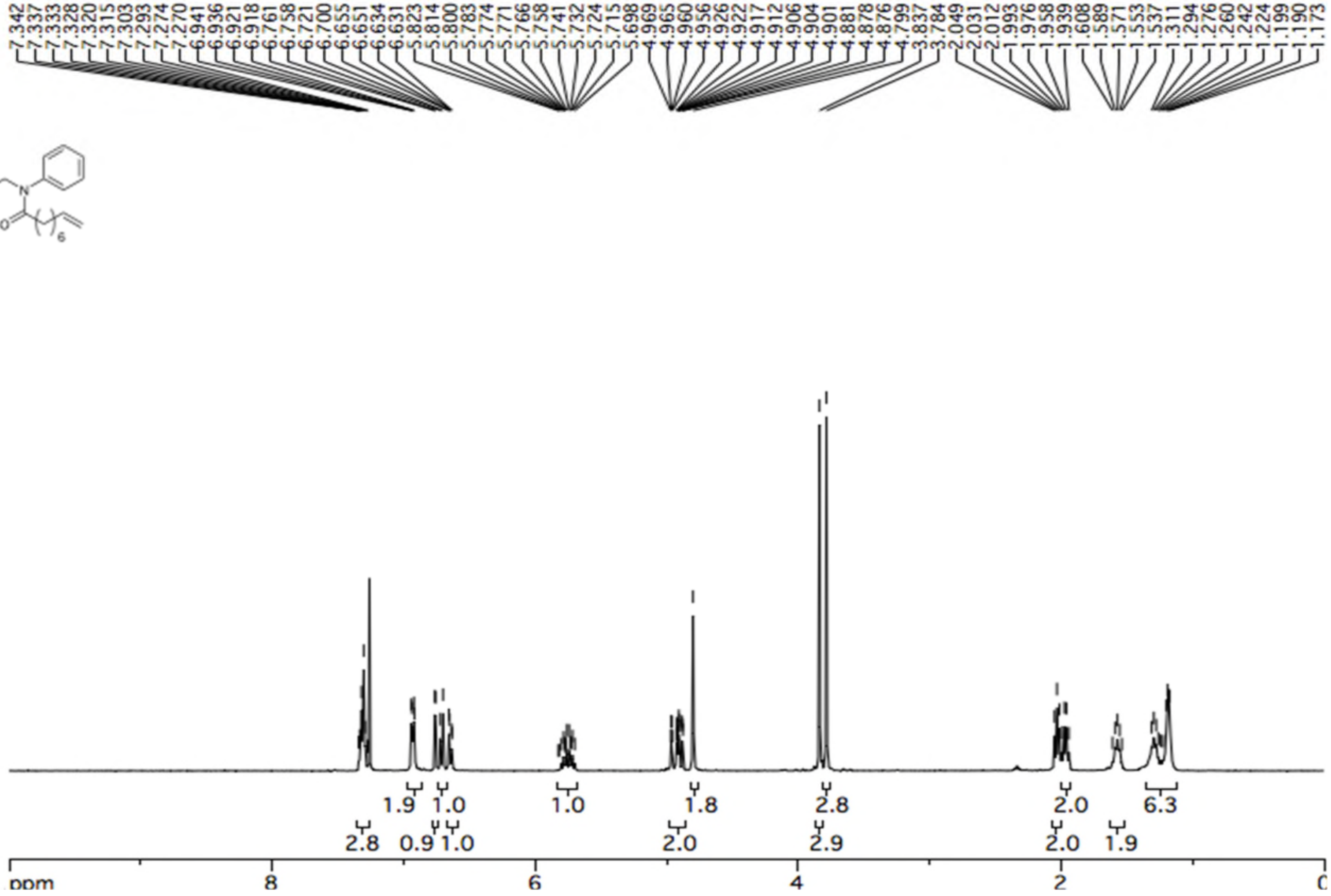

N-(3,4-dimethoxybenzyl)-*N*-phenylnon-8-enamide (**28**) – ^{13}C NMR

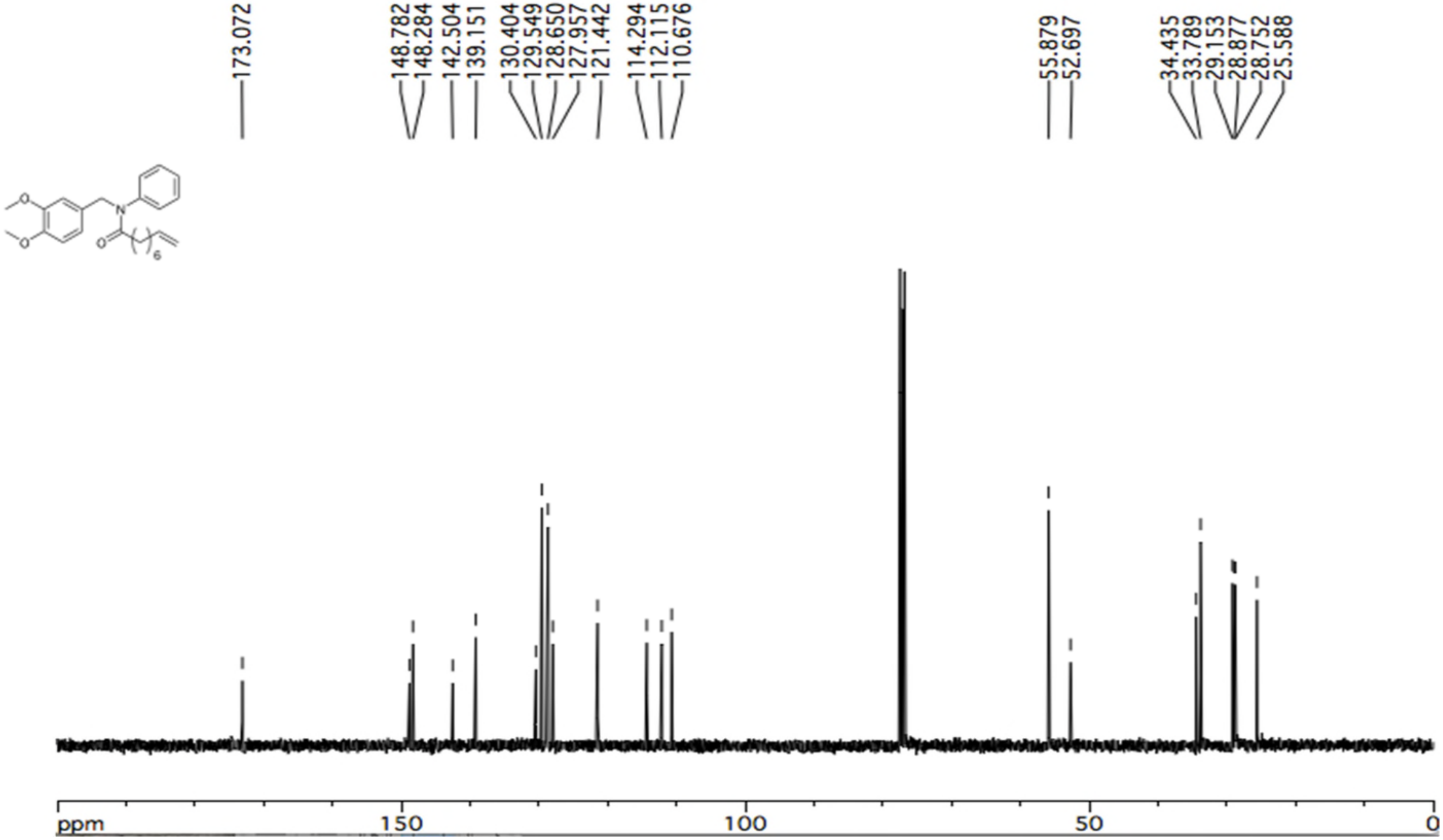

N-(3,4-dimethoxybenzyl)-N-phenylnon-8-enamide (**28**) – IR

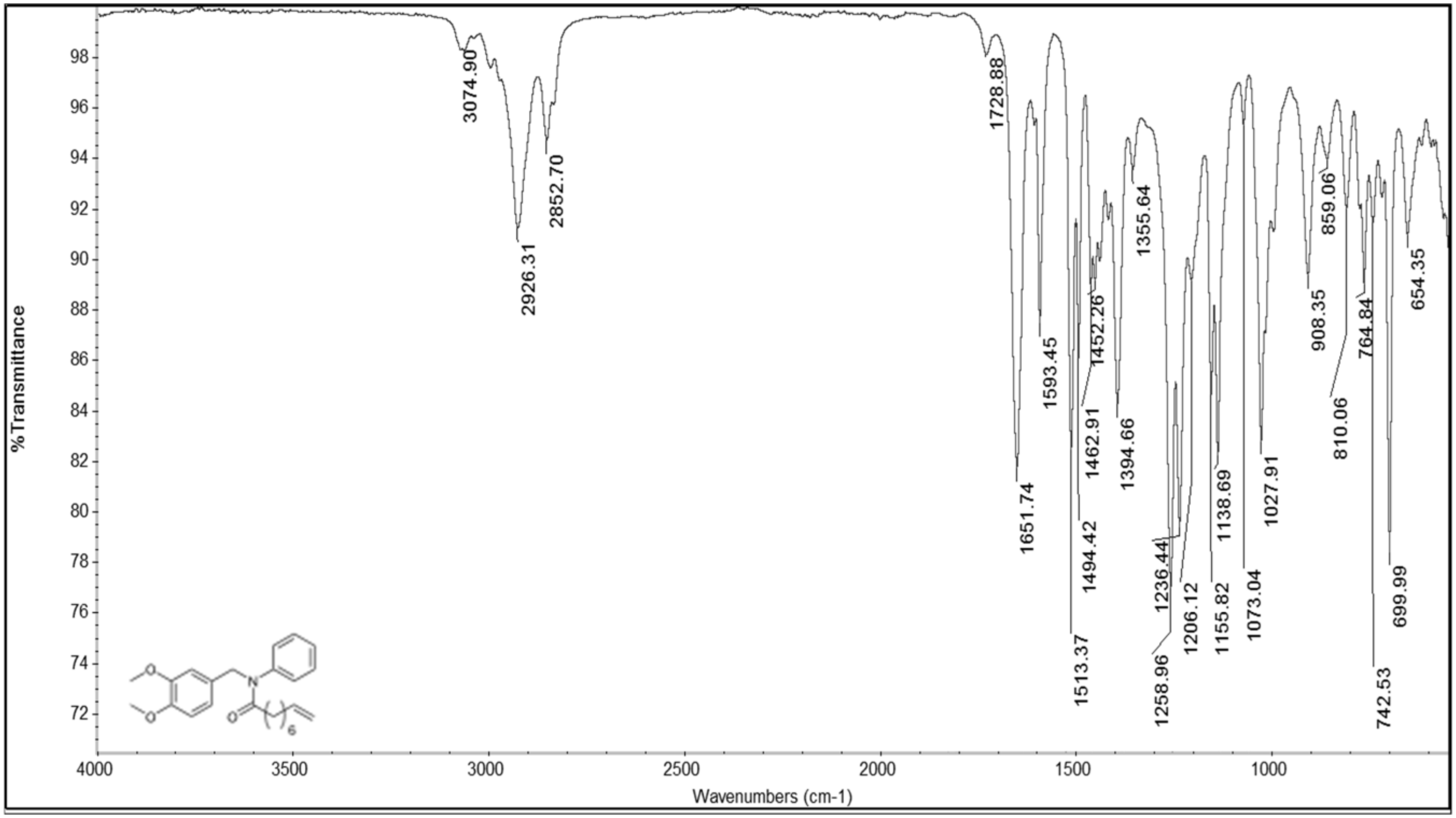

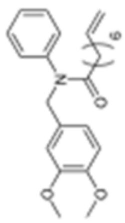

Analysis Info

Analysis Name	D:\Data\Xiao\Nov 05 2020000015.d	Acquisition Date	11/5/2020 2:15:24 PM
Method	Xiao 2.m	Operator	Administrator
Sample Name	JM-38	Instrument	micrOTOF 57
Comment			

Acquisition Parameter

				Set Corrector Fill	45 V
Source Type	ESI	Ion Polarity	Positive	Set Pulsar Pull	399 V
Scan Range	n/a	Capillary Exit	90.0 V	Set Pulsar Push	399 V
Scan Begin	50 m/z	Hexapole RF	125.0 V	Set Reflector	1300 V
Scan End	1500 m/z	Skimmer 1	40.0 V	Set Flight Tube	9000 V
		Hexapole 1	23.0 V	Set Detector TOF	2200 V

Sum Formula	Sigma	m/z	Err [ppm]	Mean Err [ppm]	rdb	N Rule	e⁻
C 24 H 31 N 1 Na 1 O 3	0.12	404.2196	1.34	0.74	9.50	ok	even

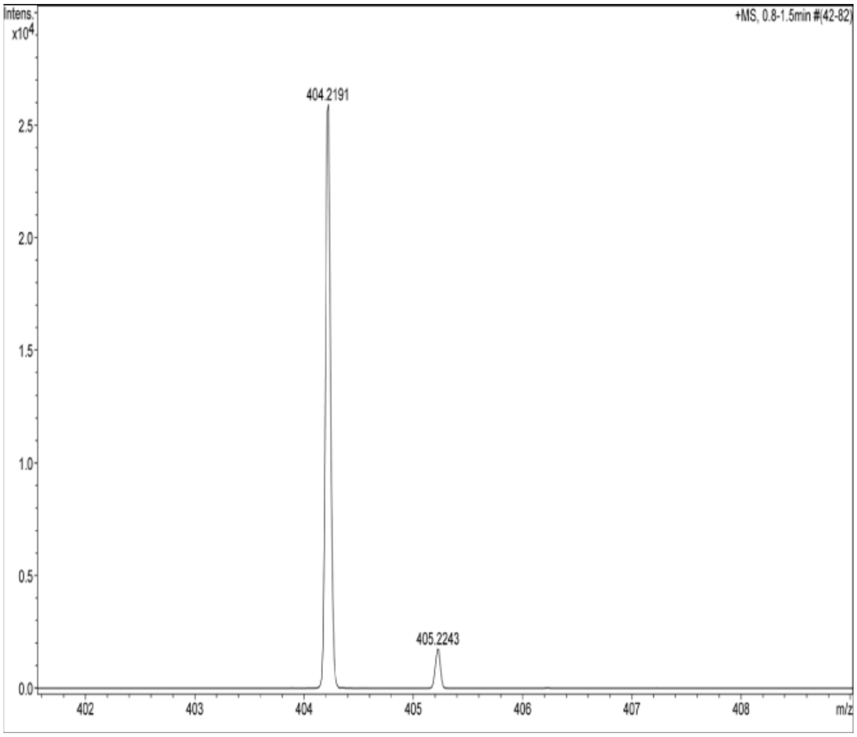

N-(3,4-dimethoxybenzyl)-*N*-phenylnon-8-enamide (**28**) – HRMS

N-(4-butylphenyl)-*N*-(3,4-dimethoxybenzyl)non-8-enamide (**29**) – ^{1}H NMR

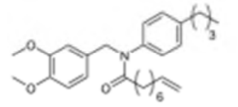

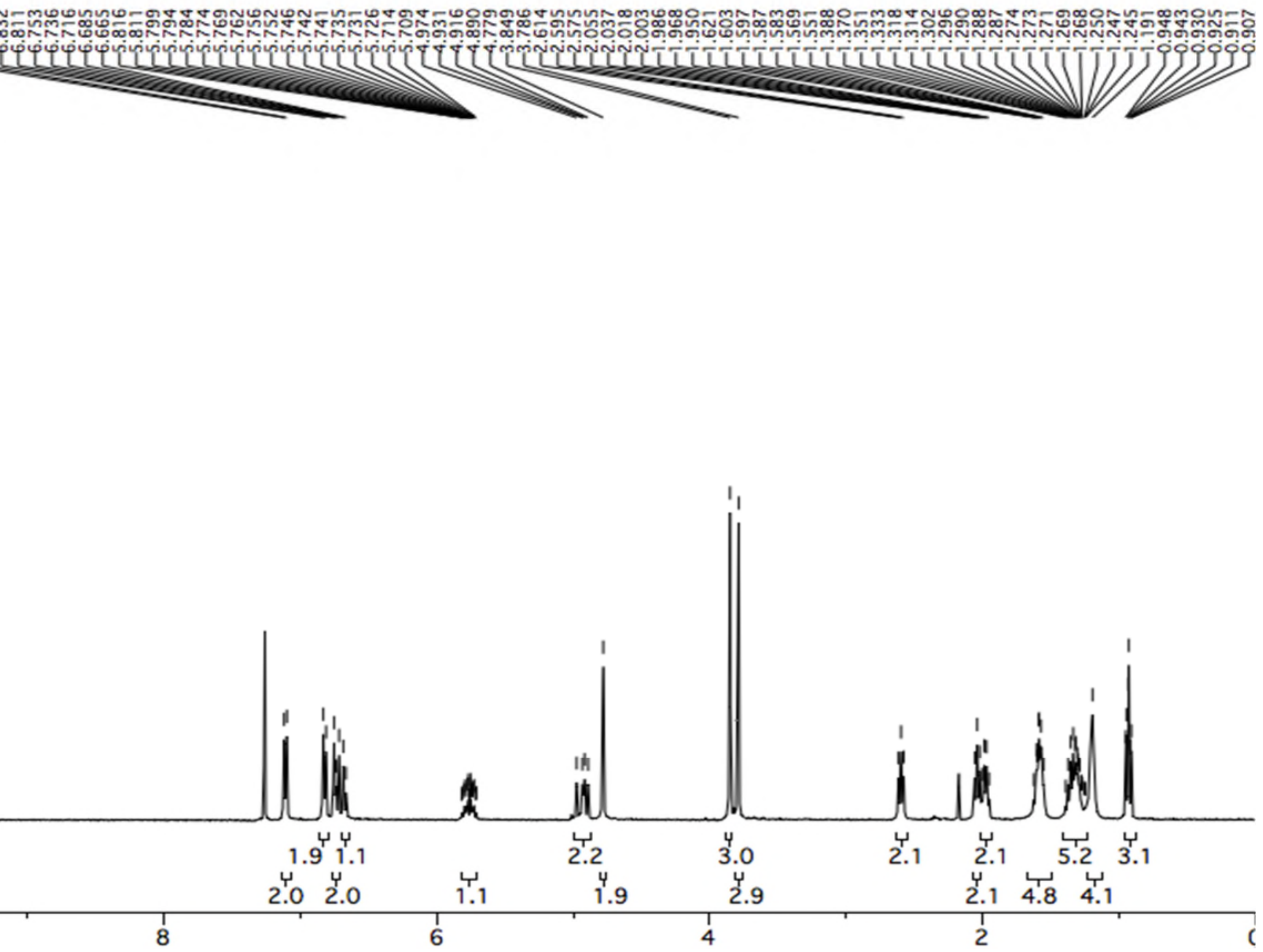

N-(4-butylphenyl)-*N*-(3,4-dimethoxybenzyl)non-8-enamide (**29**) – ^{13}C NMR

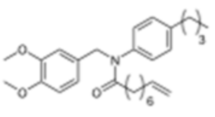

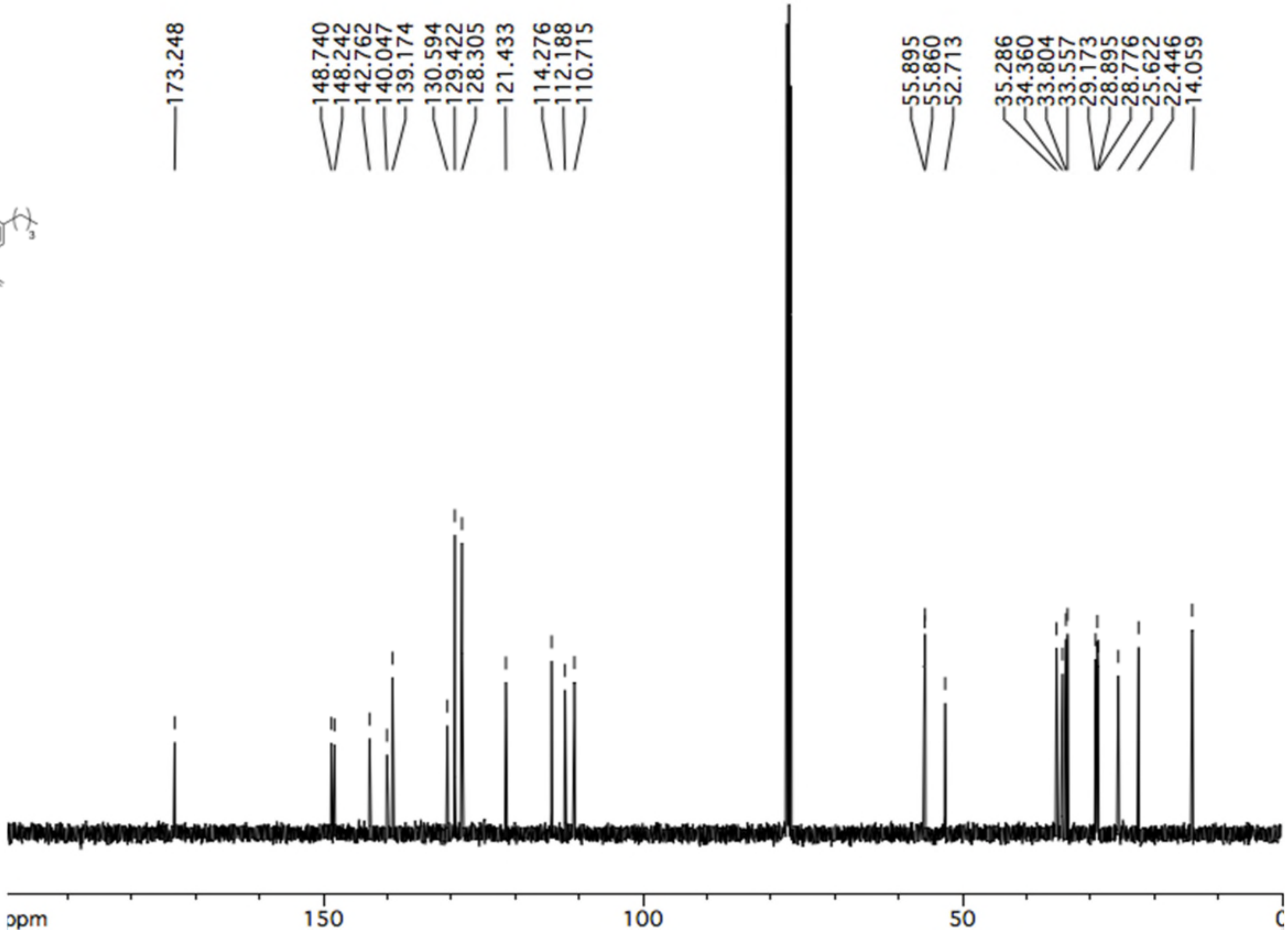

N-(4-butylphenyl)-N-(3,4-dimethoxybenzyl)non-8-enamide (**29**) – IR

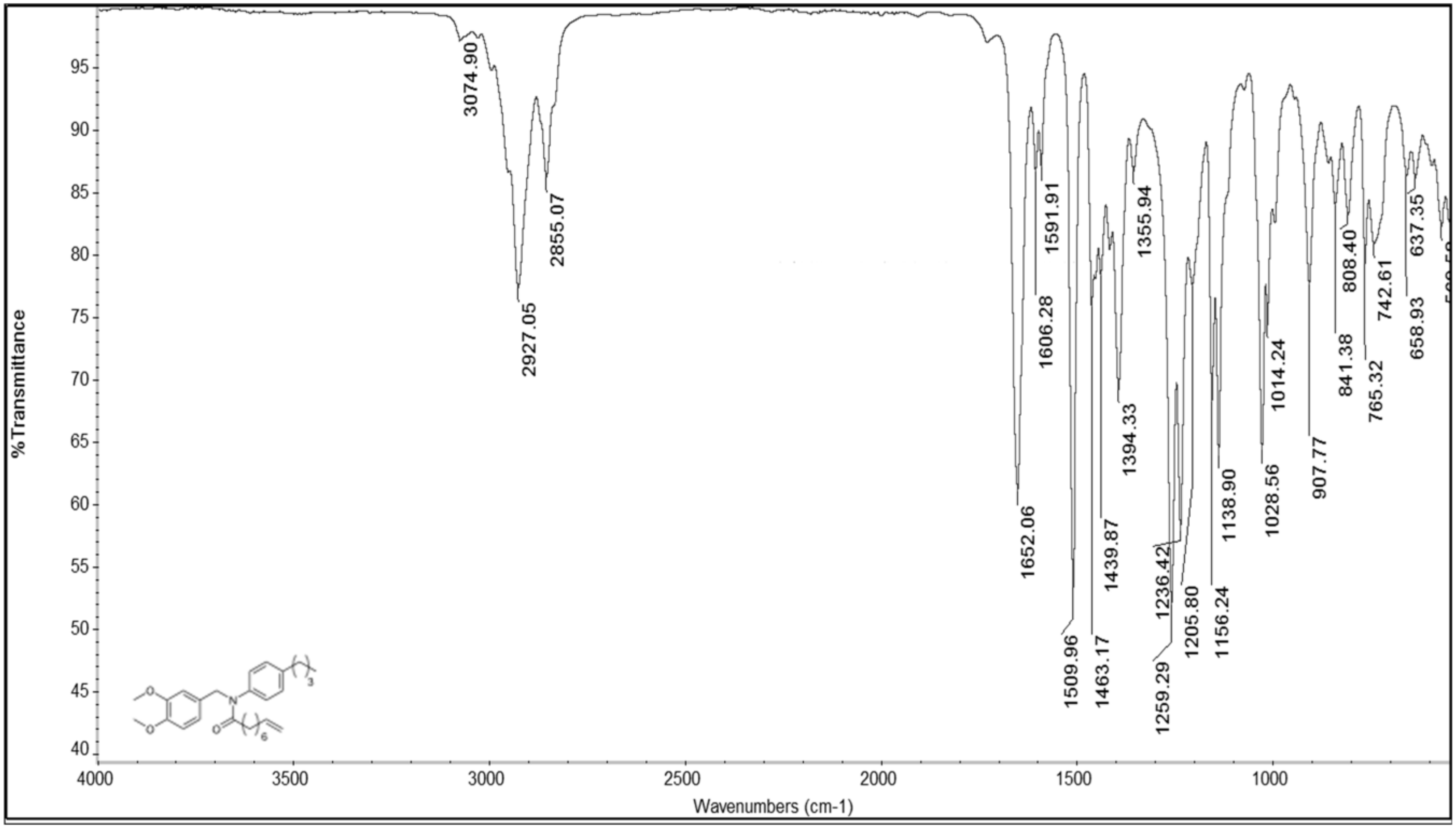

<u>*N*-(4-butylphenyl)-*N*-(3,4-dimethoxybenzyl)non-8-enamide (**29**) – HRMS</u>

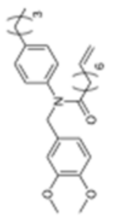

Analysis Info

Analysis Name	D:\Data\Xiao\Nov 05 2020000001.d	Acquisition Date	11/5/2020 9:50:03 AM
Method	Xiao 2.m	Operator	Administrator
Sample Name	JM-52	Instrument	micrOTOF 57
Comment			

Acquisition Parameter

Source Type	ESI	Ion Polarity	Positive	Set Corrector Fill	45 V
Scan Range	n/a	Capillary Exit	90.0 V	Set Pulsar Pull	399 V
Scan Begin	50 m/z	Hexapole RF	125.0 V	Set Pulsar Push	399 V
Scan End	1500 m/z	Skimmer 1	40.0 V	Set Reflector	1300 V
		Hexapole 1	23.0 V	Set Flight Tube	9000 V
				Set Detector TOF	2200 V

Sum Formula	Sigma	m/z	Err [ppm]	Mean Err [ppm]	rdb	N Rule	e⁻
C 28 H 39 N 1 Na 1 O 3	0.14	460.2822	-0.46	-0.22	9.50	ok	even

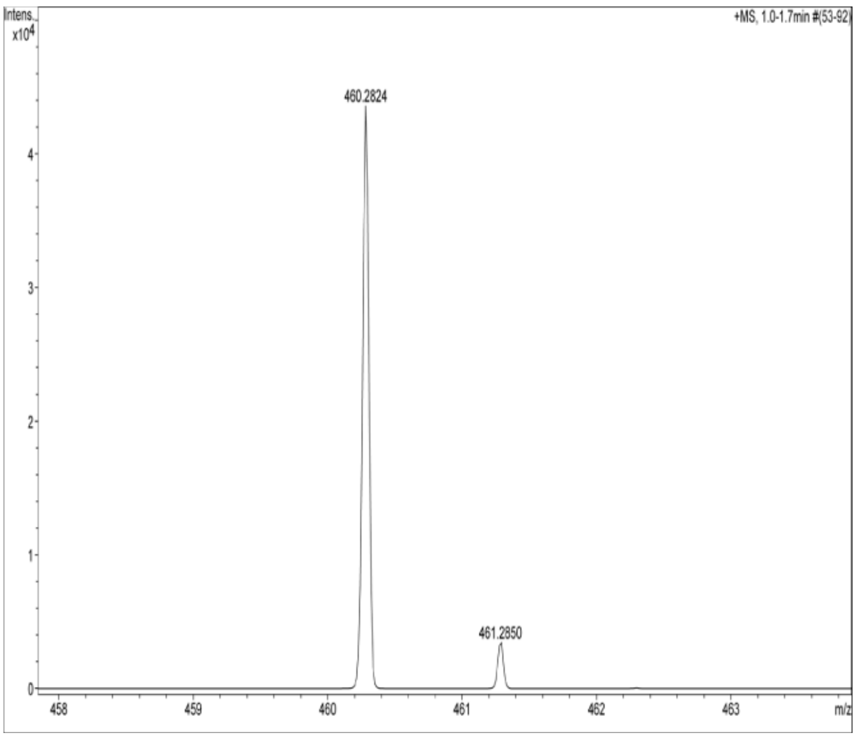

<u>*N*-(3,4-dimethoxybenzyl)-*N*-(4-tetradecylphenyl)non-8-enamide (**30**) – ^{1}H NMR</u>

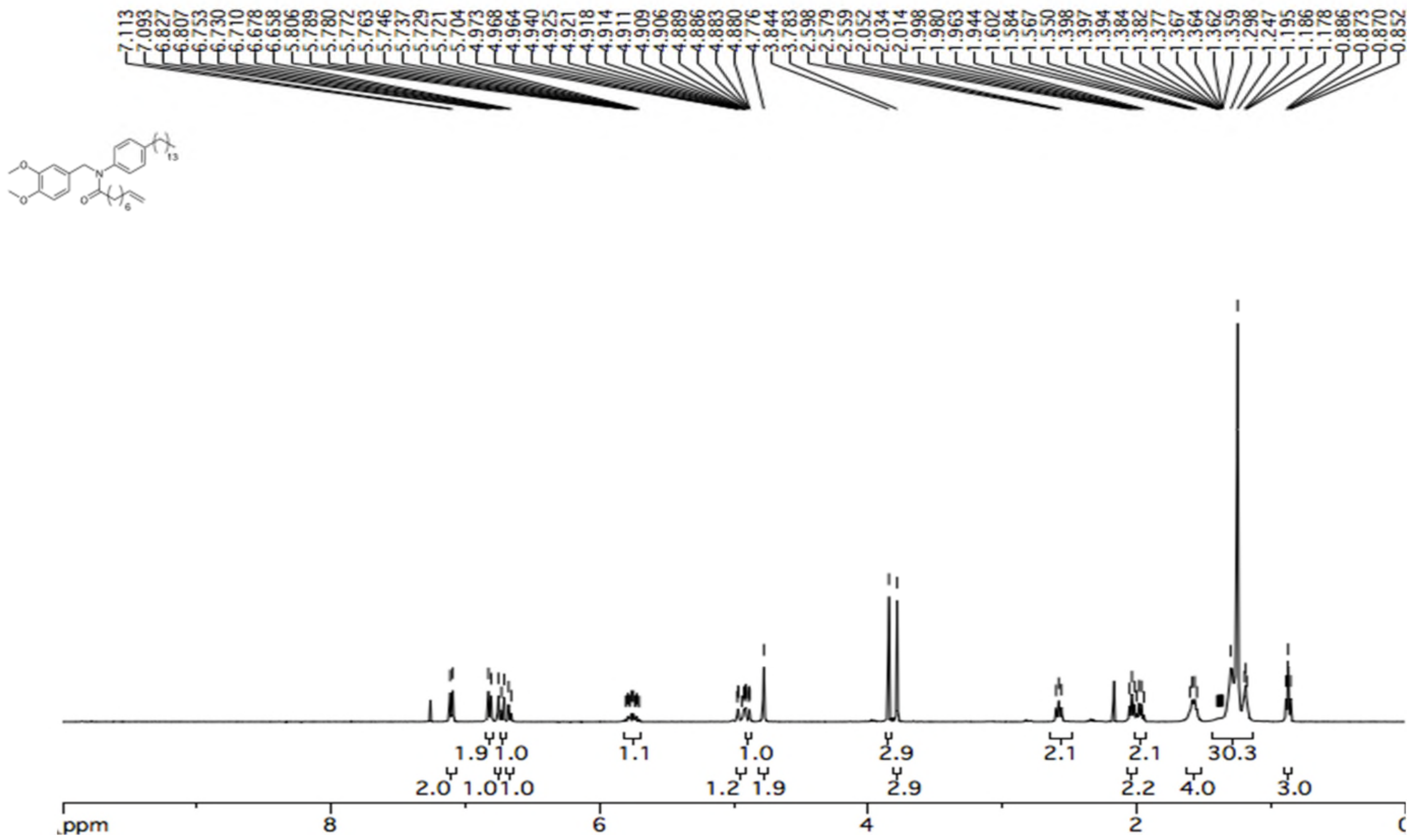

N-(3,4-dimethoxybenzyl)-N-(4-tetradecylphenyl)non-8-enamide (**30**) – ^{13}C NMR

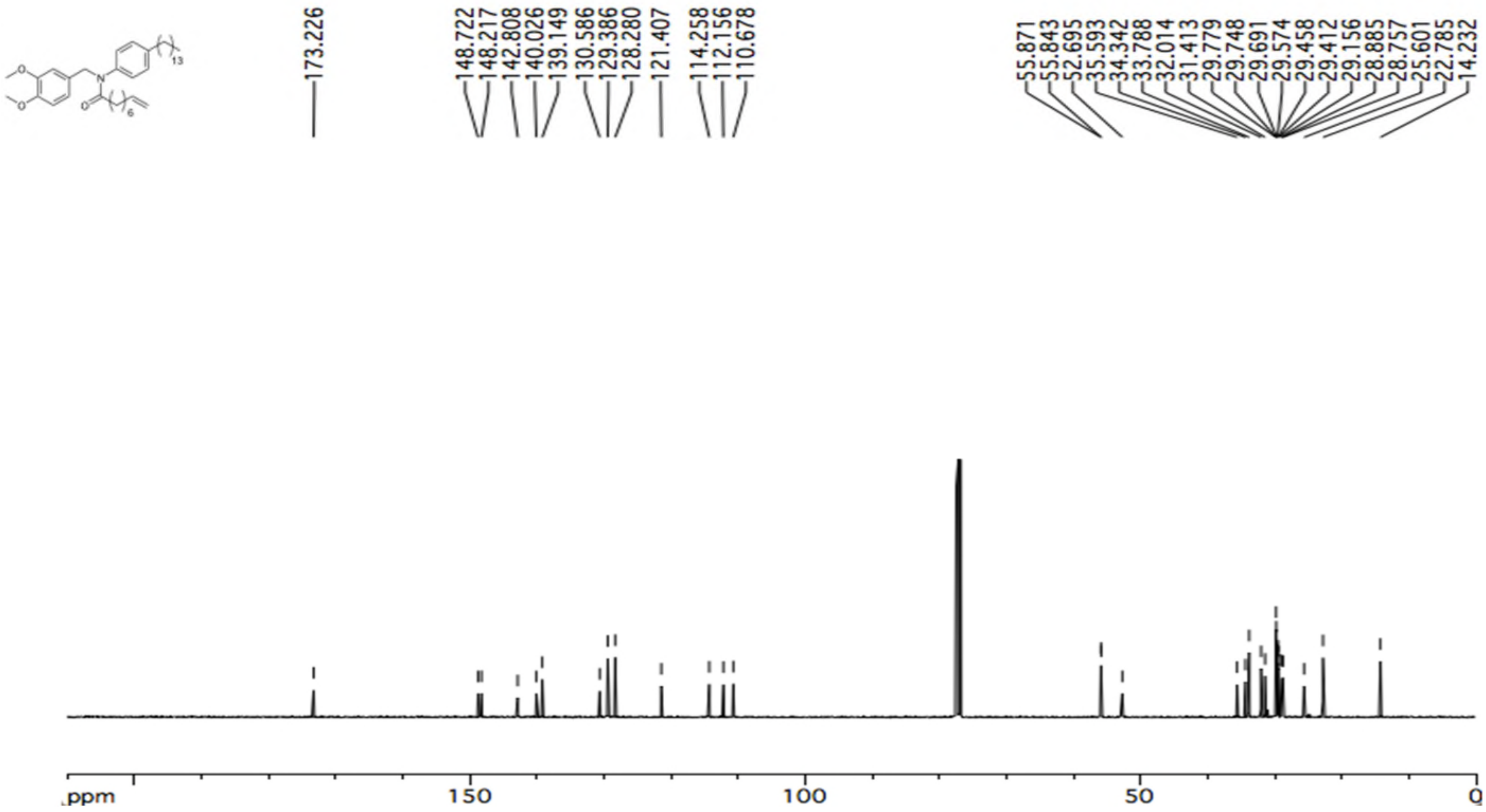

N-(3,4-dimethoxybenzyl)-N-(4-tetradecylphenyl)non-8-enamide (**30**) – IR

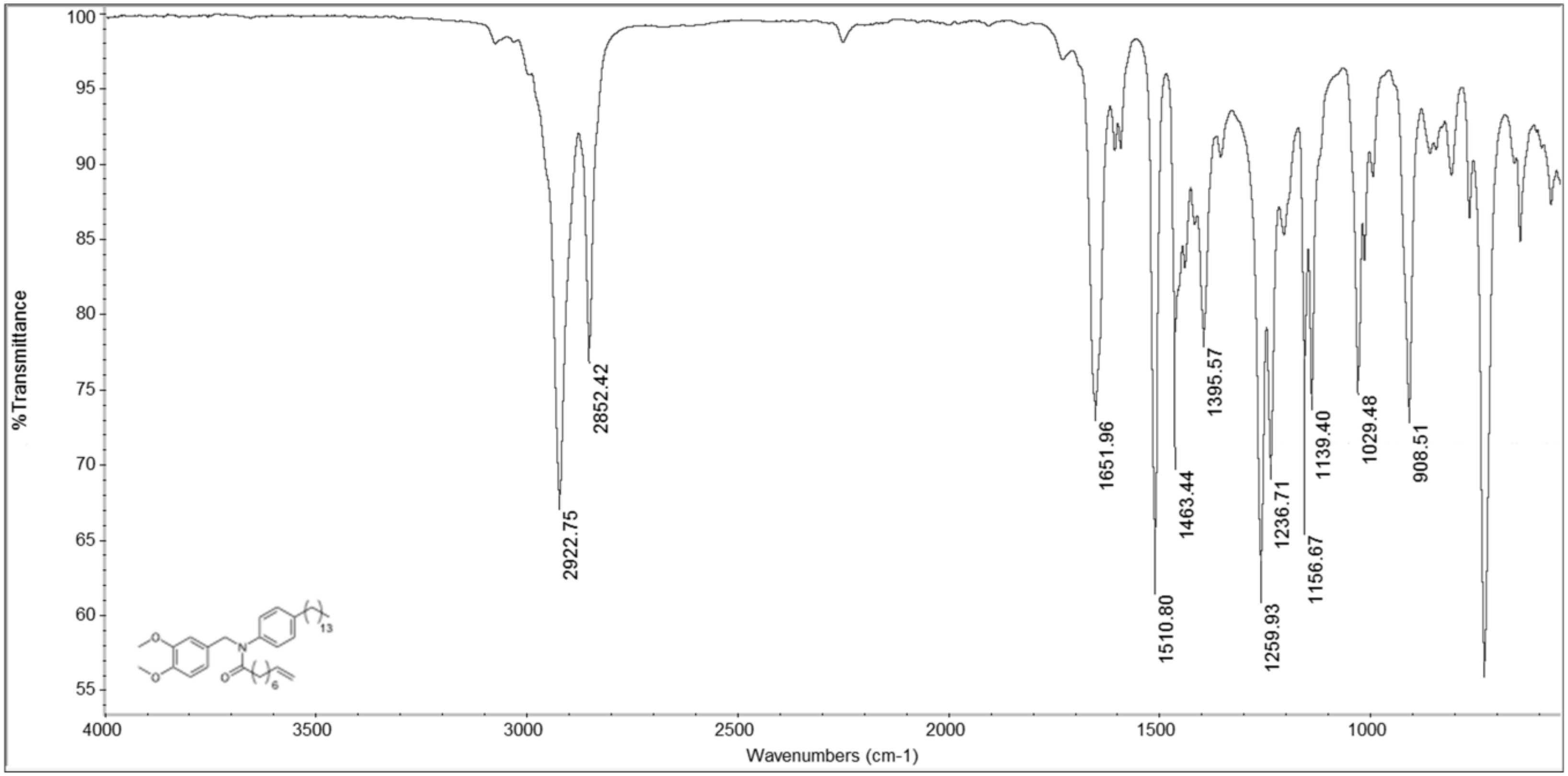

N-(3,4-dimethoxybenzyl)-*N*-(4-tetradecylphenyl)non-8-enamide (**30**) – HRMS

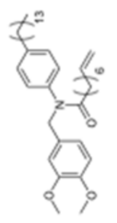

Analysis Info

Analysis Name	D:\Data\Xiao\Nov 05 2020000006.d	Acquisition Date	11/5/2020 10:38:59 AM
Method	Xiao 2.m	Operator	Administrator
Sample Name	JM-72	Instrument	micrOTOF 57
Comment			

Acquisition Parameter

Source Type	ESI	Ion Polarity	Positive	Set Corrector Fill	45 V
Scan Range	n/a	Capillary Exit	90.0 V	Set Pulsar Pull	399 V
Scan Begin	50 m/z	Hexapole RF	125.0 V	Set Pulsar Push	399 V
Scan End	1500 m/z	Skimmer 1	40.0 V	Set Reflector	1300 V
		Hexapole 1	23.0 V	Set Flight Tube	9000 V
				Set Detector TOF	2200 V

Sum Formula	Sigma	m/z	Err [ppm]	Mean Err [ppm]	rdb	N Rule	e⁻
C 38 H 59 N 1 Na 1 O 3	0.11	600.4387	1.68	2.01	9.50	ok	even

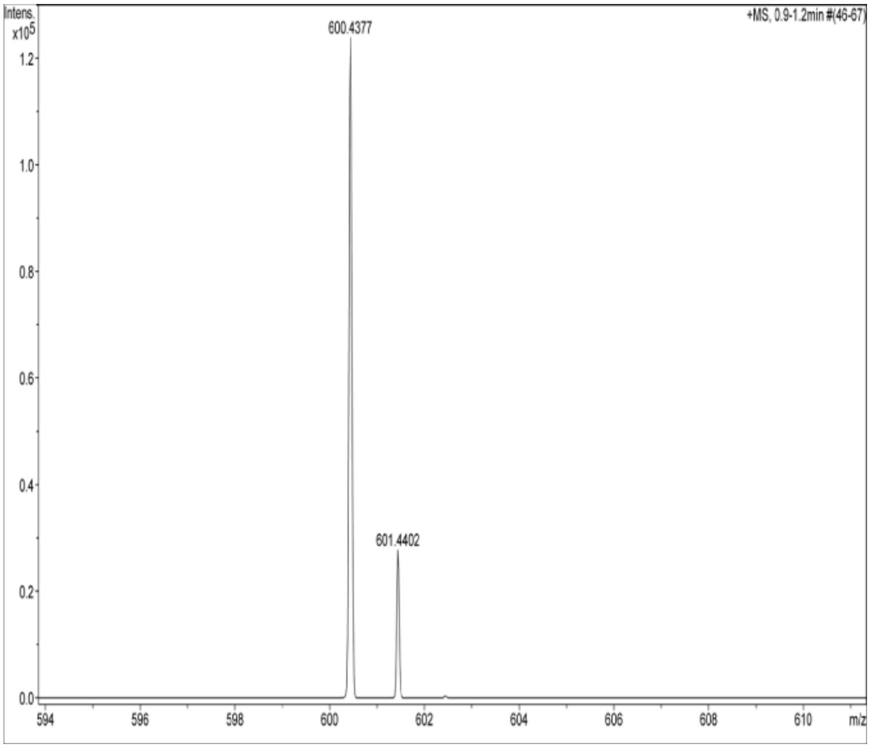

N-(3,4-dimethoxybenzyl)-*N*-phenyl-5-(4,4,5,5-tetramethyl-1,3,2-dioxaborolan-2-yl)pentanamide (**31**) – ^{1}H NMR

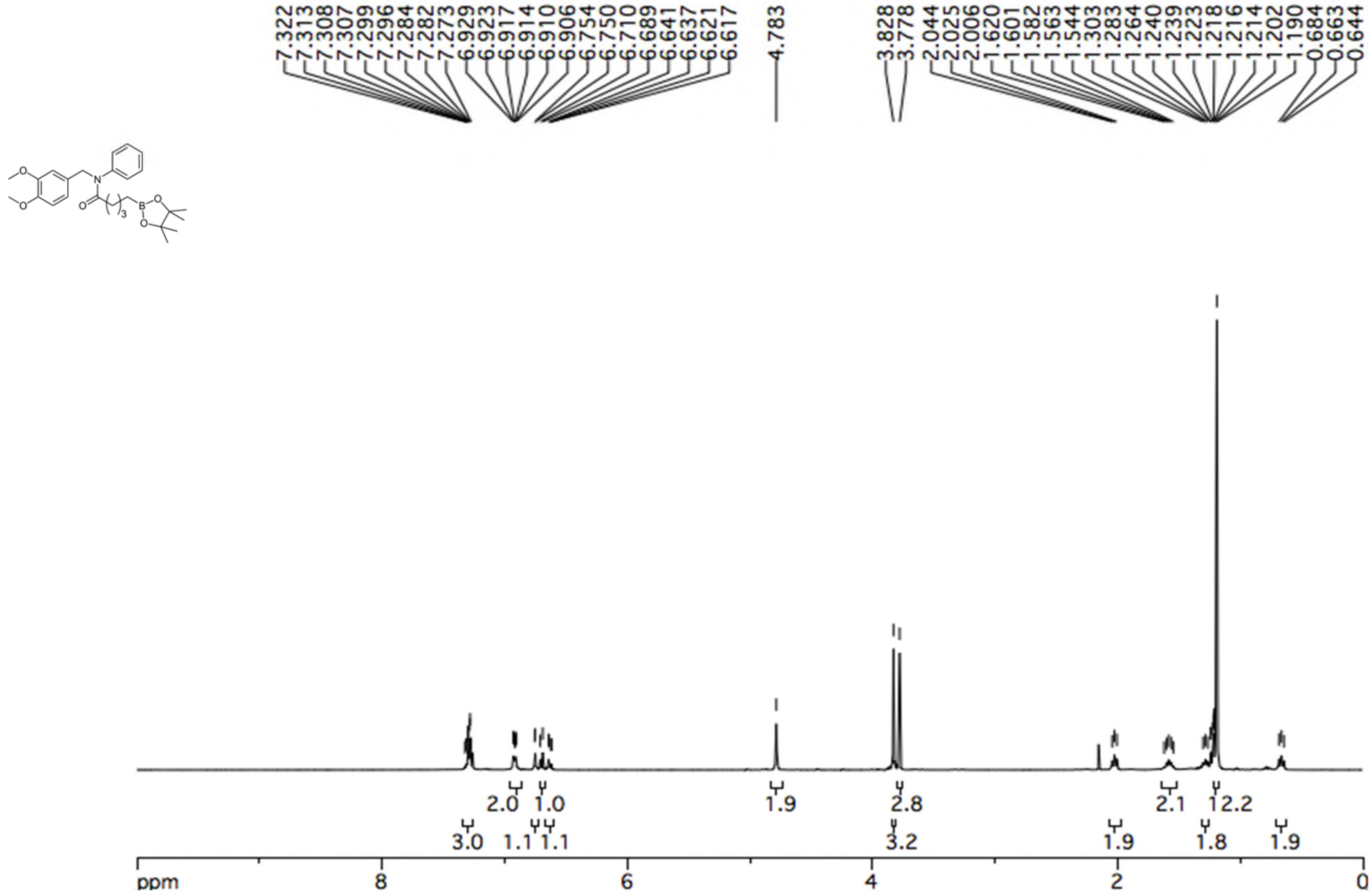

<u>*N*-(3,4-dimethoxybenzyl)-*N*-phenyl-5-(4,4,5,5-tetramethyl-1,3,2-dioxaborolan-2-yl)pentanamide (**31**) – ^{13}C NMR</u>

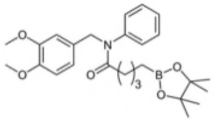

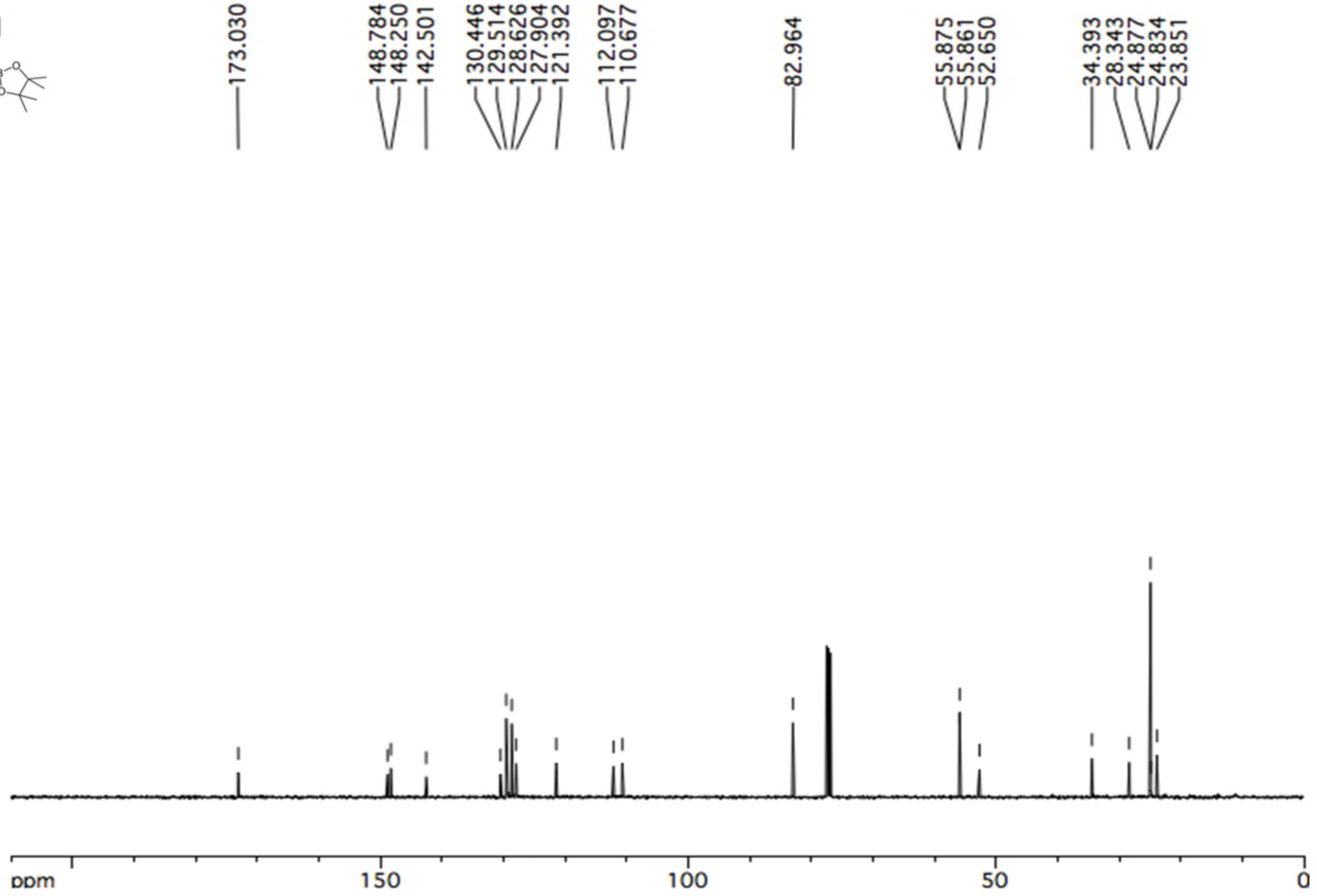

N-(3,4-dimethoxybenzyl)-N-phenyl-5-(4,4,5,5-tetramethyl-1,3,2-dioxaborolan-2-yl)pentanamide (**31**) – ^{11}B NMR

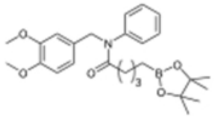

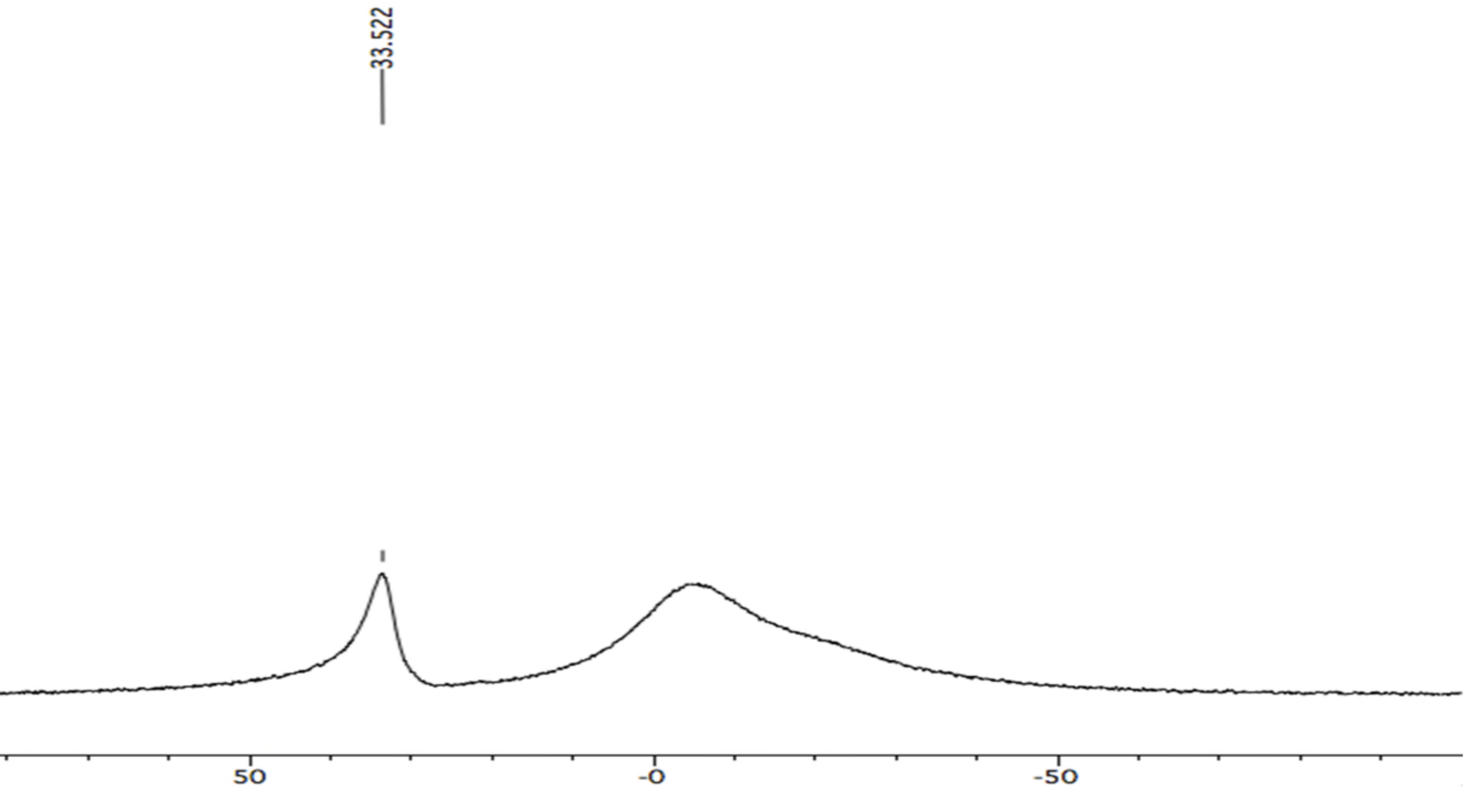

N-(3,4-dimethoxybenzyl)-*N*-phenyl-5-(4,4,5,5-tetramethyl-1,3,2-dioxaborolan-2-yl)pentanamide (**31**) – IR

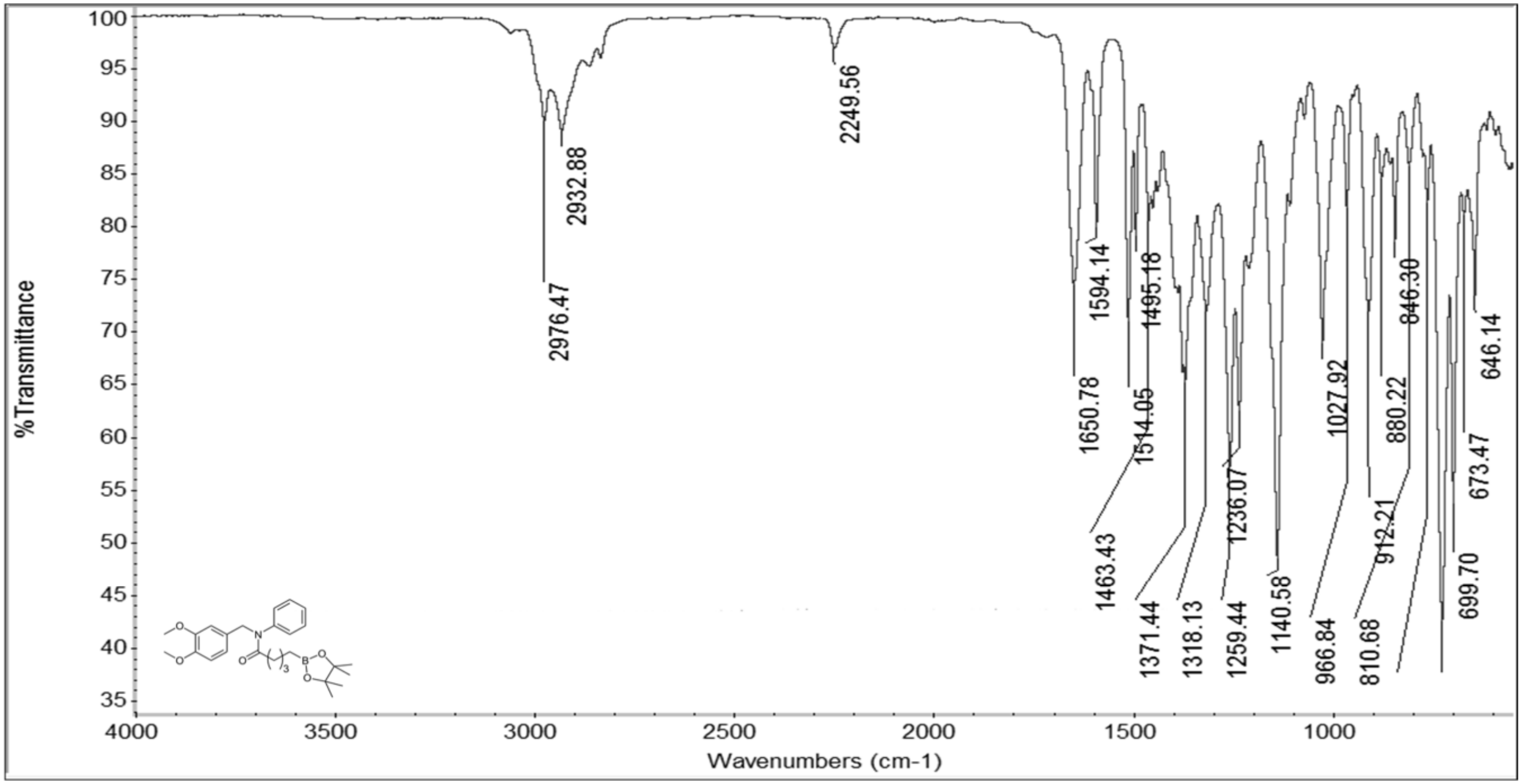

N-(3,4-dimethoxybenzyl)-*N*-phenyl-5-(4,4,5,5-tetramethyl-1,3,2-dioxaborolan-2-yl)pentanamide (**31**) – HRMS

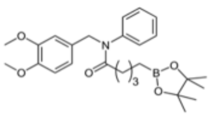

Analysis Info

Analysis Name	D:\Data\Xiao\Nov 05 2020000020.d	Acquisition Date	11/5/2020 3:06:15 PM
Method	Xiao 2.m	Operator	Administrator
Sample Name	JM-47	Instrument	micrOTOF 57
Comment			

Acquisition Parameter

Source Type	ESI	Ion Polarity	Positive	Set Corrector Fill	45 V
Scan Range	n/a	Capillary Exit	90.0 V	Set Pulsar Pull	399 V
Scan Begin	50 m/z	Hexapole RF	125.0 V	Set Pulsar Push	399 V
Scan End	1500 m/z	Skimmer 1	40.0 V	Set Reflector	1300 V
		Hexapole 1	23.0 V	Set Flight Tube	9000 V
				Set Detector TOF	2200 V

Sum Formula	Sigma	m/z	Err [ppm]	Mean Err [ppm]	rdb	N Rule	e⁻
C 26 H 36 B 1 N 1 Na 1 O 5	0.15	476.2579	0.94	1.95	9.50	ok	even

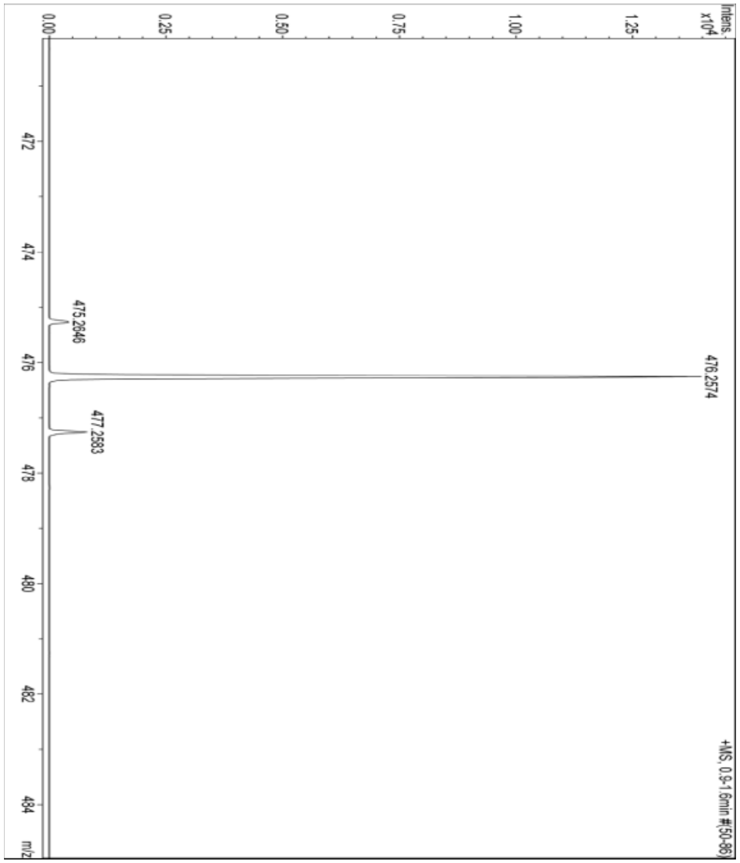

N-(3,4-dimethoxybenzyl)-*N*-phenyl-9-(4,4,5,5-tetramethyl-1,3,2-dioxaborolan-2-yl)nonamide (**32**) – ^{1}H NMR

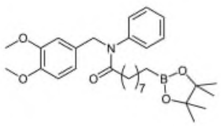

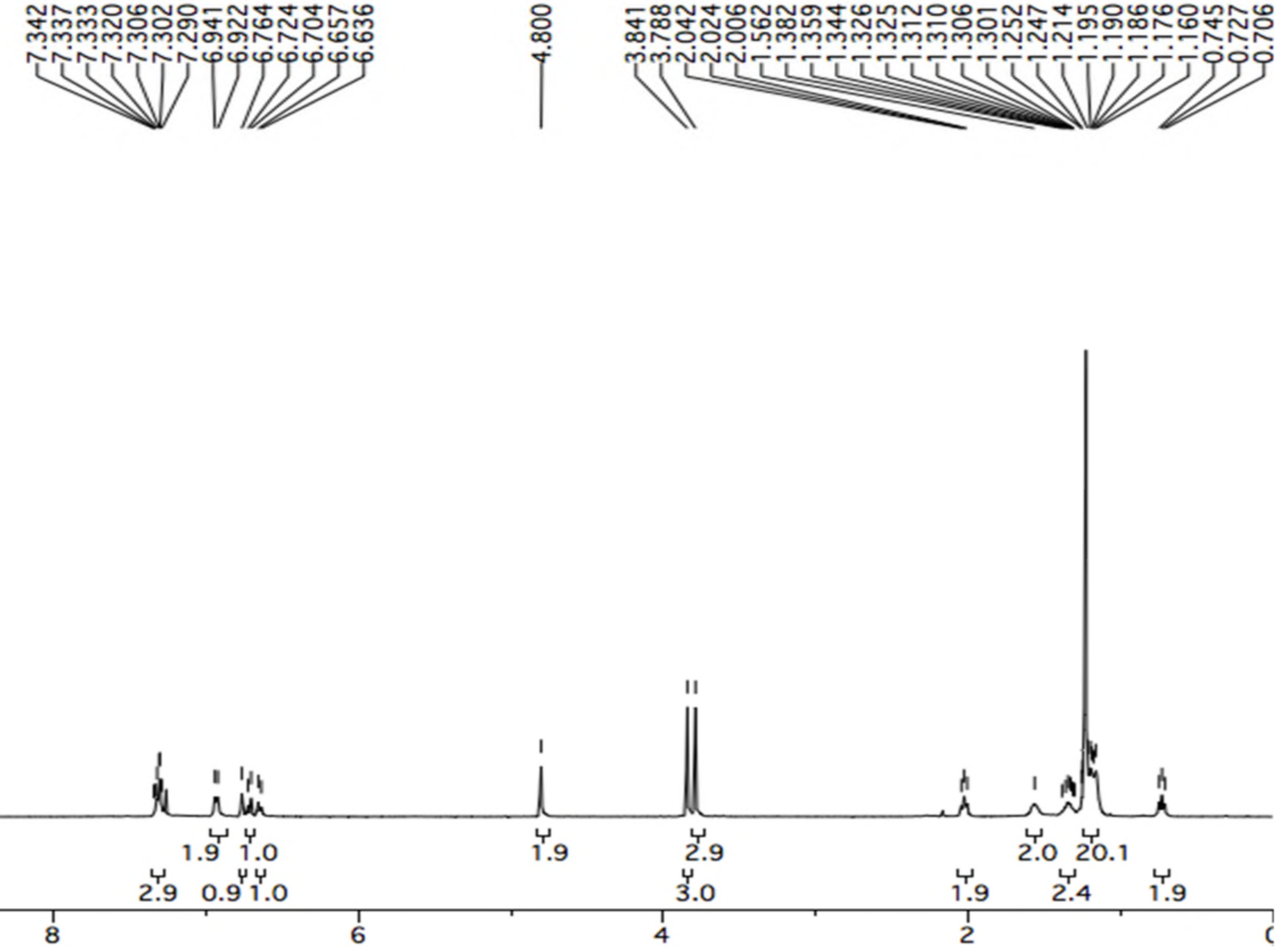

N-(3,4-dimethoxybenzyl)-N-phenyl-9-(4,4,5,5-tetramethyl-1,3,2-dioxaborolan-2-yl)nonamide (**32**) – ^{13}C NMR

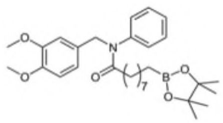

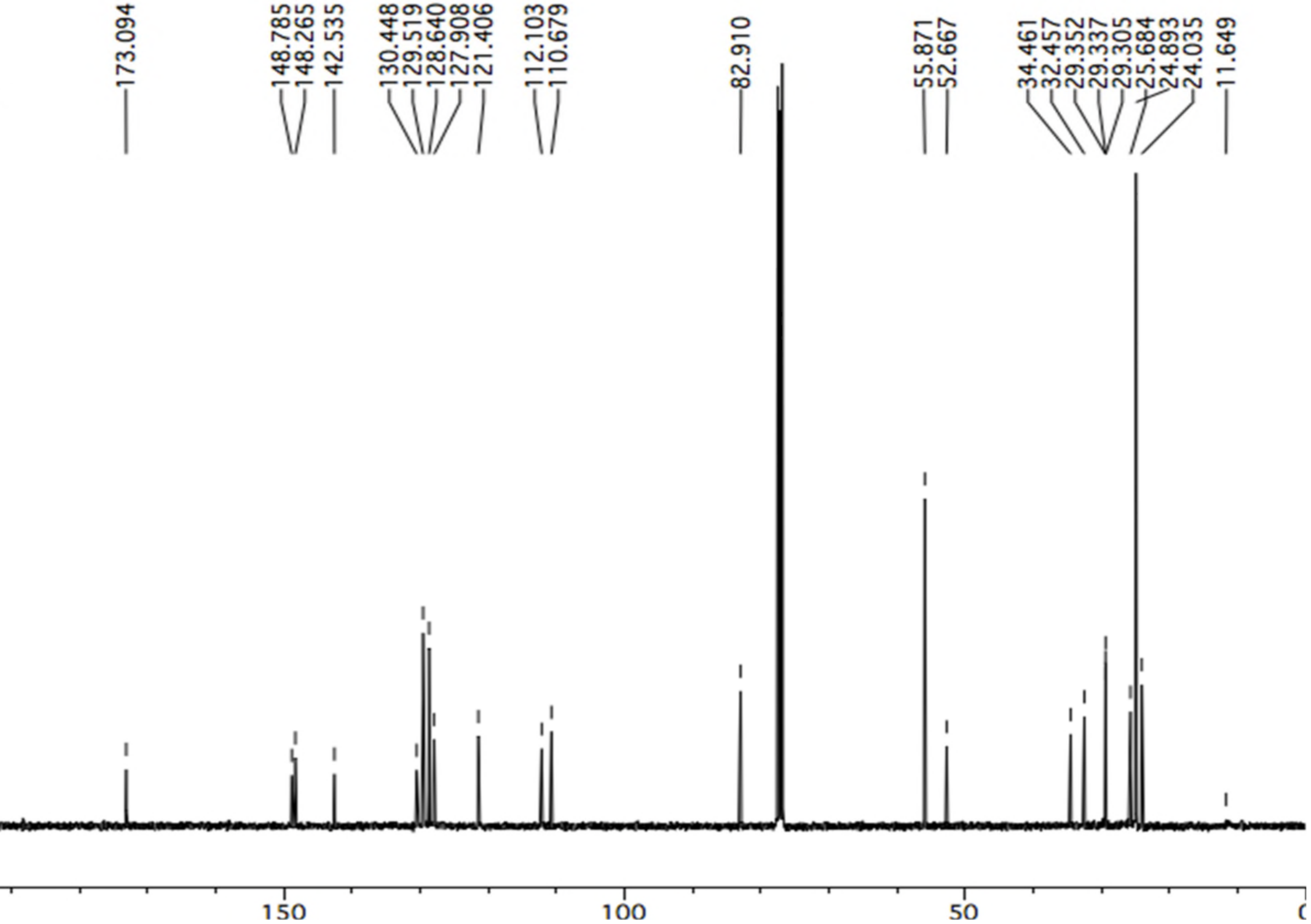

N-(3,4-dimethoxybenzyl)-N-phenyl-9-(4,4,5,5-tetramethyl-1,3,2-dioxaborolan-2-yl)nonamide (**32**) – ^{11}B NMR

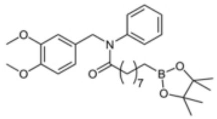

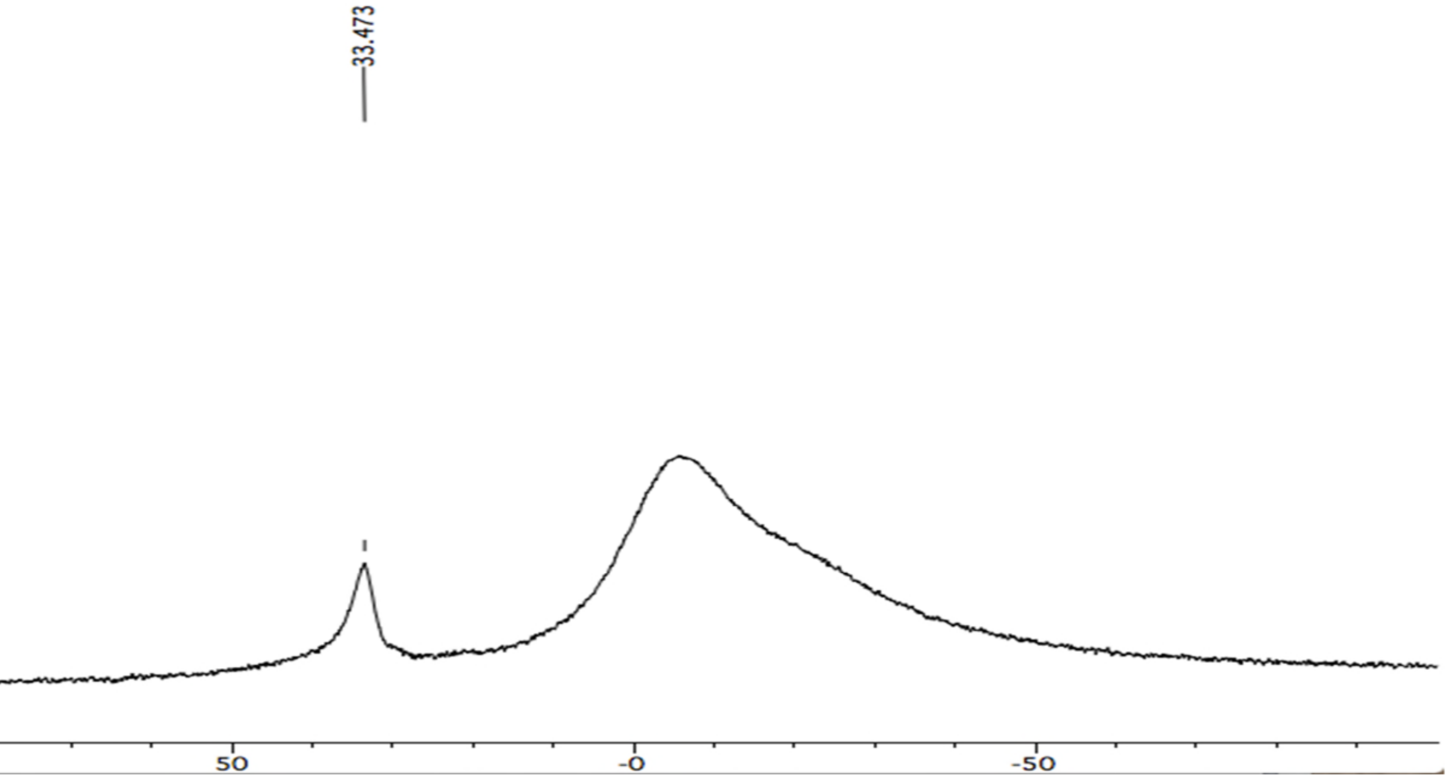

N-(3,4-dimethoxybenzyl)-*N*-phenyl-9-(4,4,5,5-tetramethyl-1,3,2-dioxaborolan-2-yl)nonamide (**32**) – IR

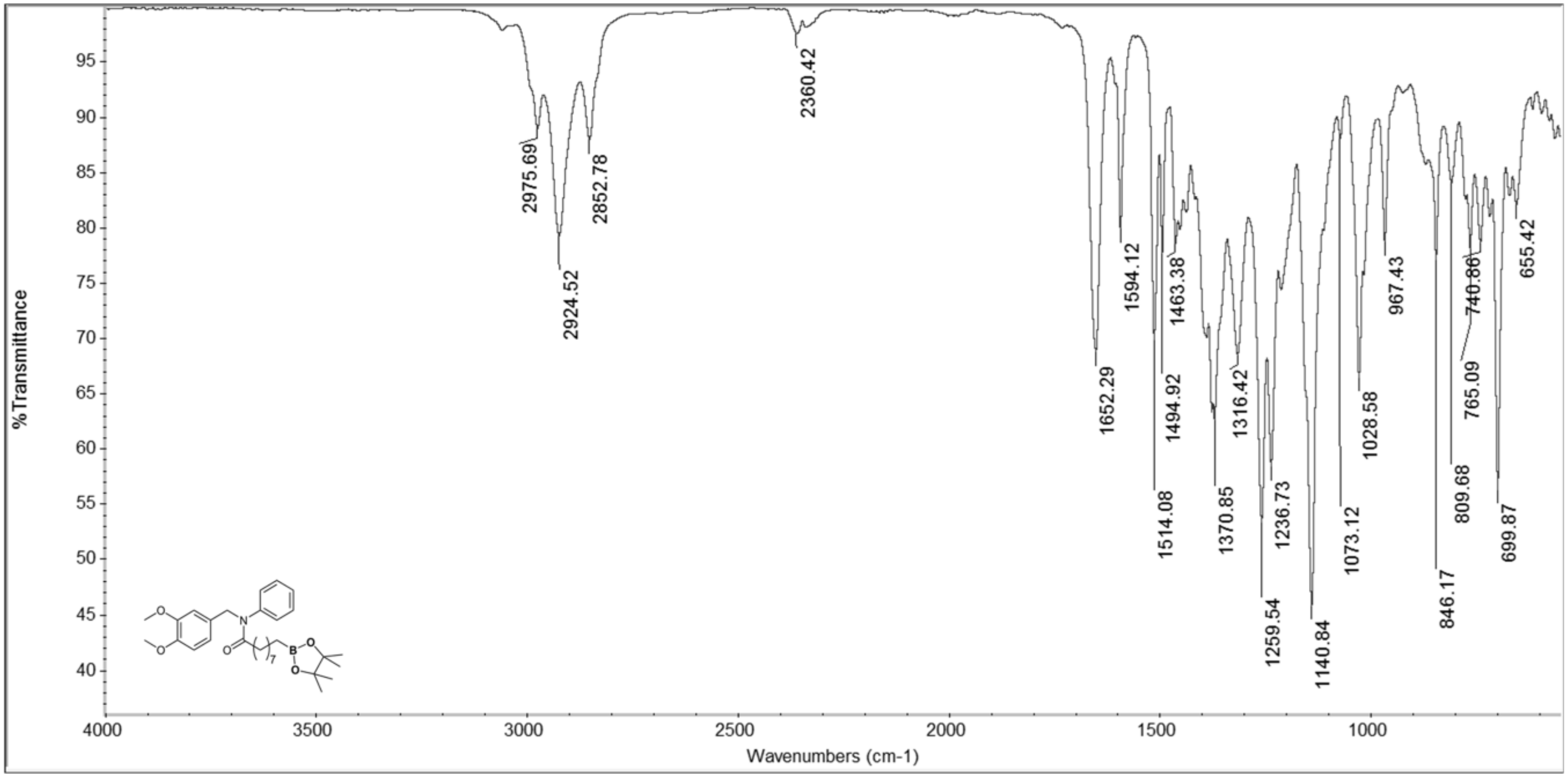

N-(3,4-dimethoxybenzyl)-*N*-phenyl-9-(4,4,5,5-tetramethyl-1,3,2-dioxaborolan-2-yl)nonamide (**32**) – HRMS

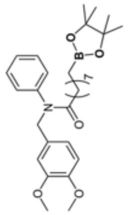

Analysis Info

Analysis Name	D:\Data\Xiao\Nov 05 2020000008.d	Acquisition Date	11/5/2020 10:57:50 AM
Method	Xiao 2.m	Operator	Administrator
Sample Name	JM-61	Instrument	micrOTOF 57
Comment			

Acquisition Parameter

				Set Corrector Fill	45 V
Source Type	ESI	Ion Polarity	Positive	Set Pulsar Pull	399 V
Scan Range	n/a	Capillary Exit	90.0 V	Set Pulsar Push	399 V
Scan Begin	50 m/z	Hexapole RF	125.0 V	Set Reflector	1300 V
Scan End	1500 m/z	Skimmer 1	40.0 V	Set Flight Tube	9000 V
		Hexapole 1	23.0 V	Set Detector TOF	2200 V

Sum Formula	Sigma	m/z	Err [ppm]	Mean Err [ppm]	rdb	N Rule	e⁻
C 30 H 44 B 1 N 1 Na 1 O 5	0.15	532.3205	2.02	2.98	9.50	ok	even

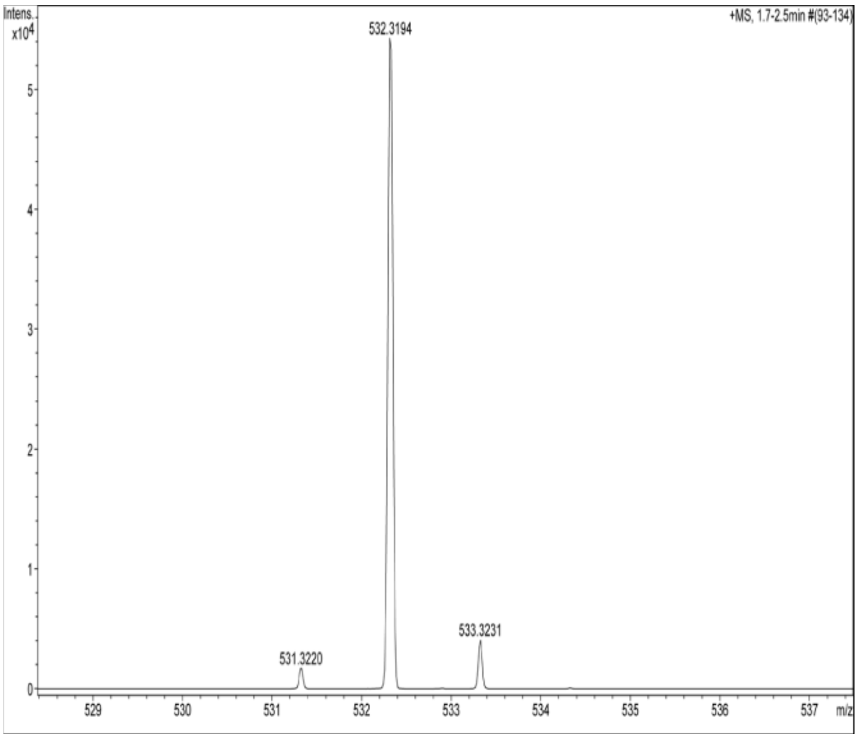

<u>*N*-(4-butylphenyl)-*N*-(3,4-dimethoxybenzyl)-9-(4,4,5,5-tetramethyl-1,3,2-dioxaborolan-2-yl)nonamide (**33**) – ^{1}H NMR</u>

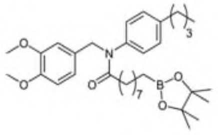

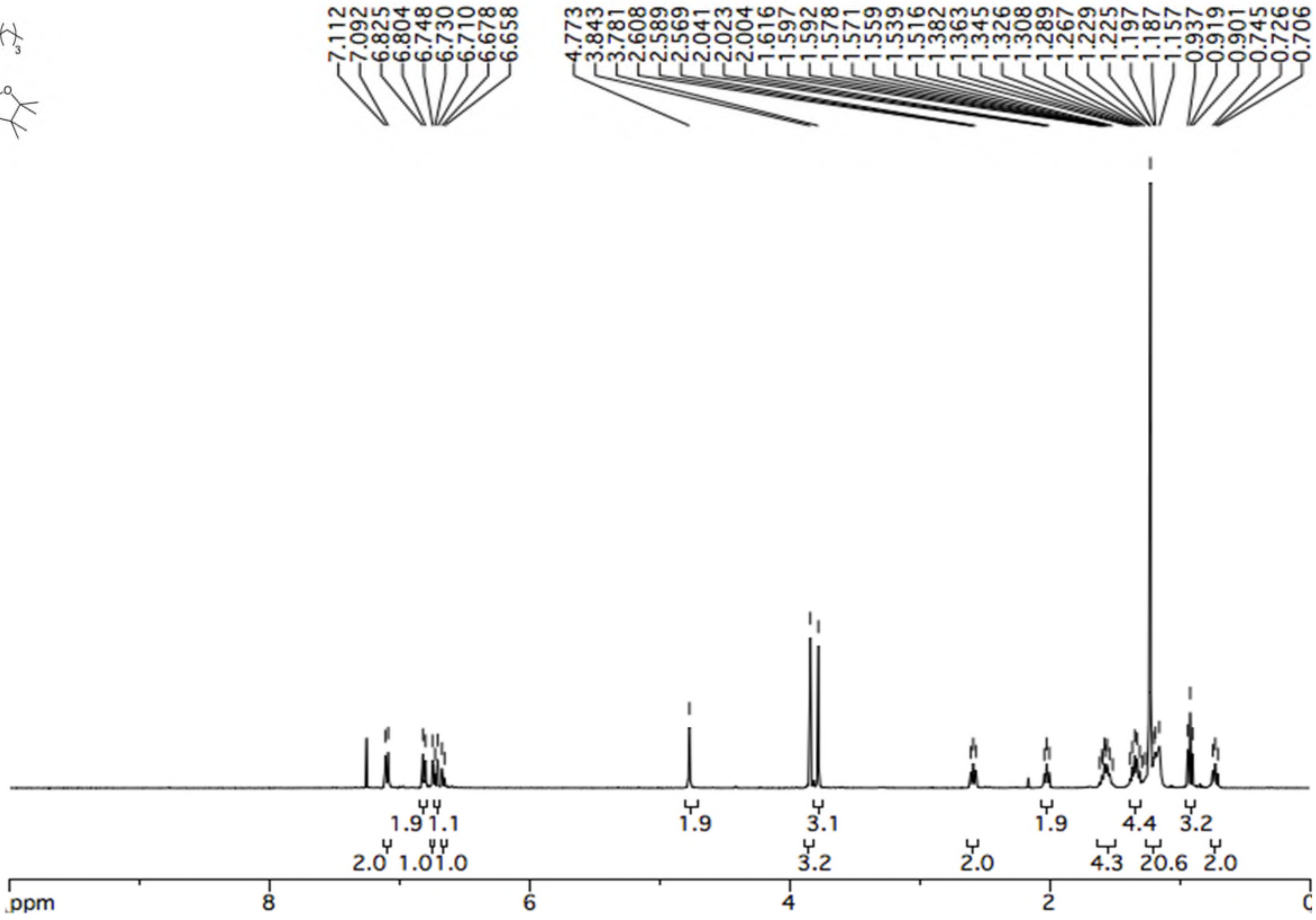

N-(4-butylphenyl)-*N*-(3,4-dimethoxybenzyl)-9-(4,4,5,5-tetramethyl-1,3,2-dioxaborolan-2-yl)nonamide (**33**) – ^{13}C NMR

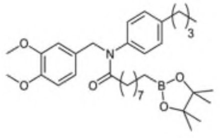

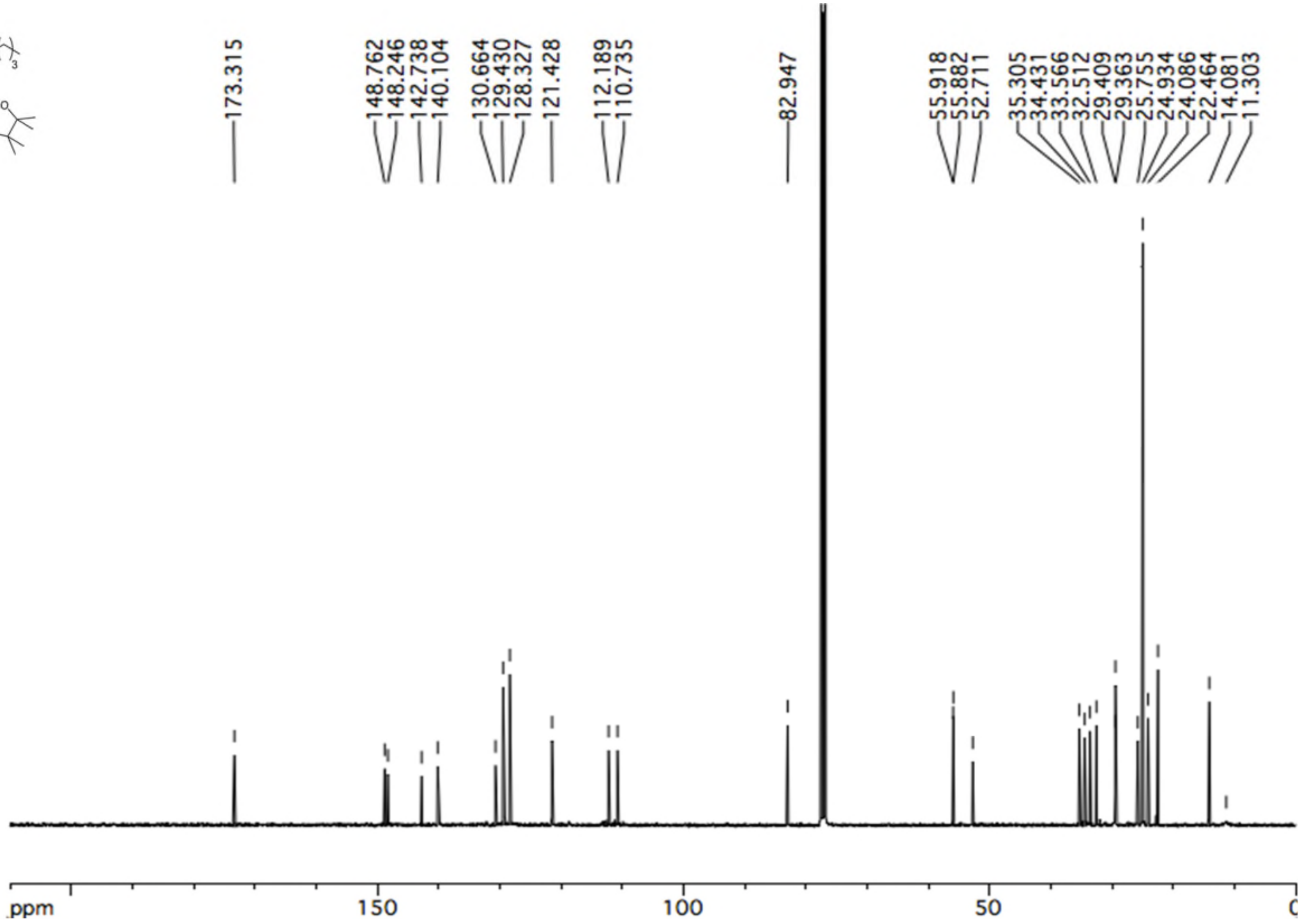

N-(4-butylphenyl)-*N*-(3,4-dimethoxybenzyl)-9-(4,4,5,5-tetramethyl-1,3,2-dioxaborolan-2-yl)nonamide (**33**) – ^{11}B NMR

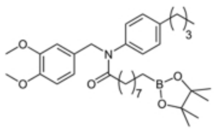

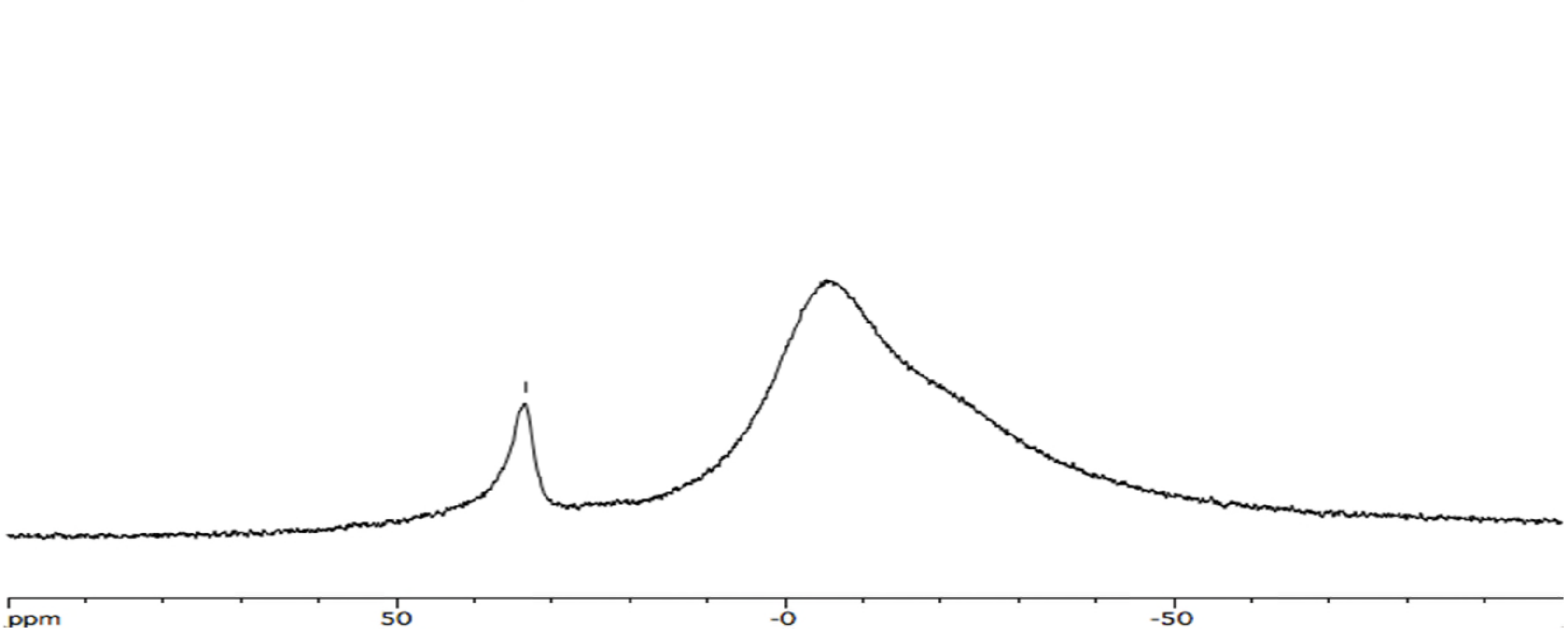

N-(4-butylphenyl)-*N*-(3,4-dimethoxybenzyl)-9-(4,4,5,5-tetramethyl-1,3,2-dioxaborolan-2-yl)nonamide (**33**) – IR

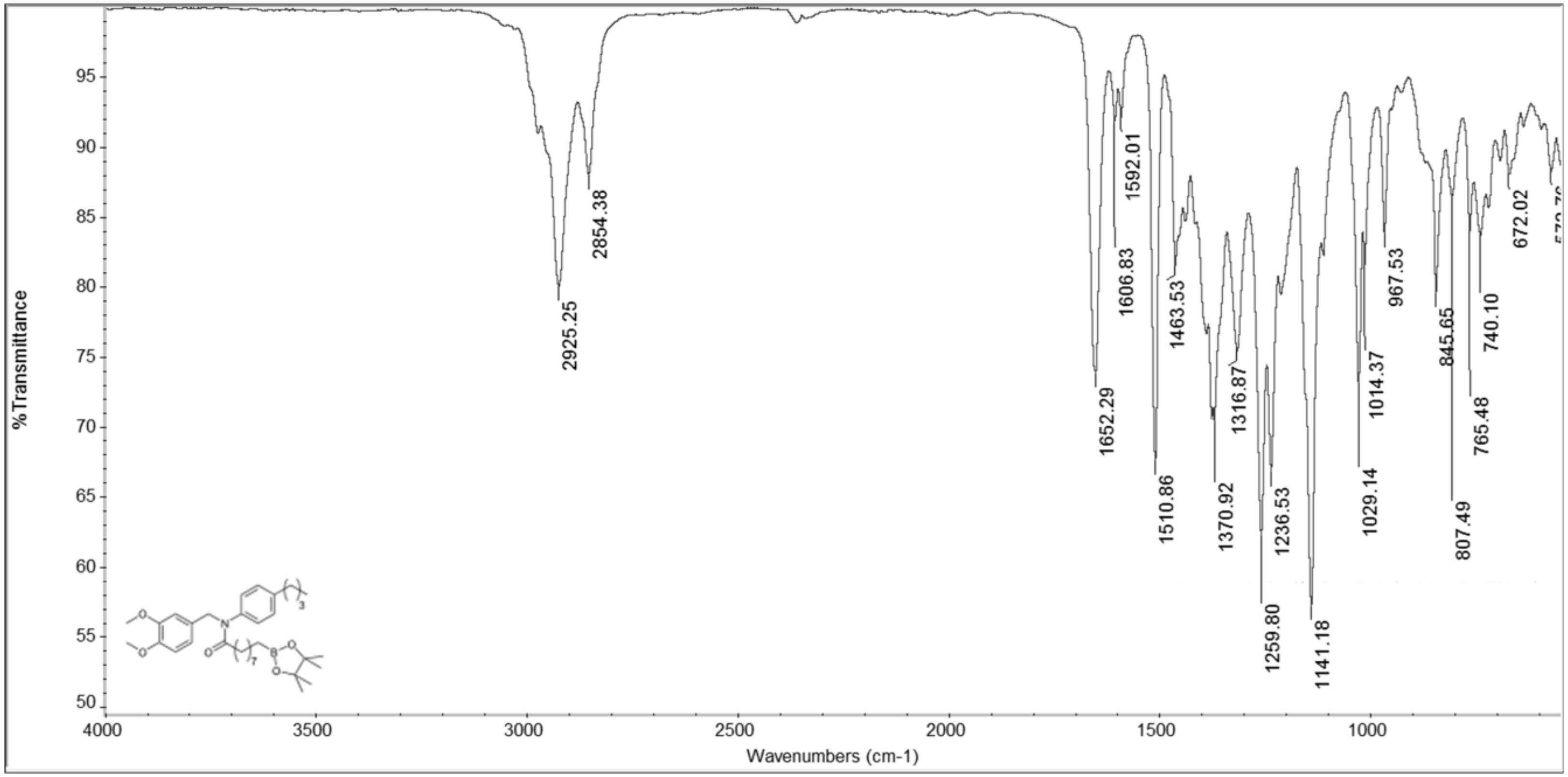

<u>*N*-(4-butylphenyl)-*N*-(3,4-dimethoxybenzyl)-9-(4,4,5,5-tetramethyl-1,3,2-dioxaborolan-2-yl)nonamide (**33**) – HRMS</u>

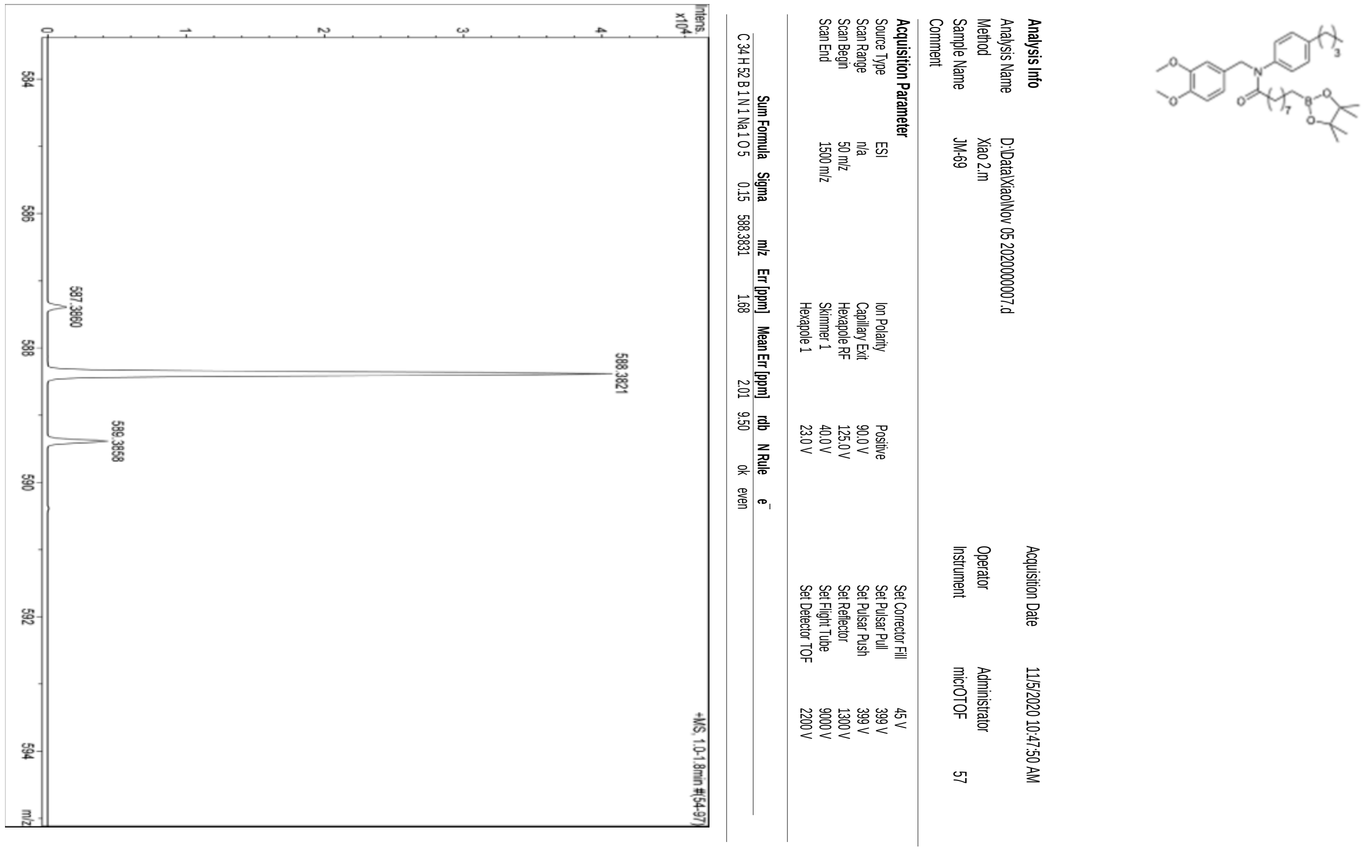

N-(3,4-dimethoxybenzyl)-*N*-(4-tetradecylphenyl)-9-(4,4,5,5-tetramethyl-1,3,2-dioxaborolan-2-yl)nonamide (**34**) – ^{1}H NMR

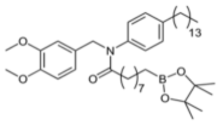

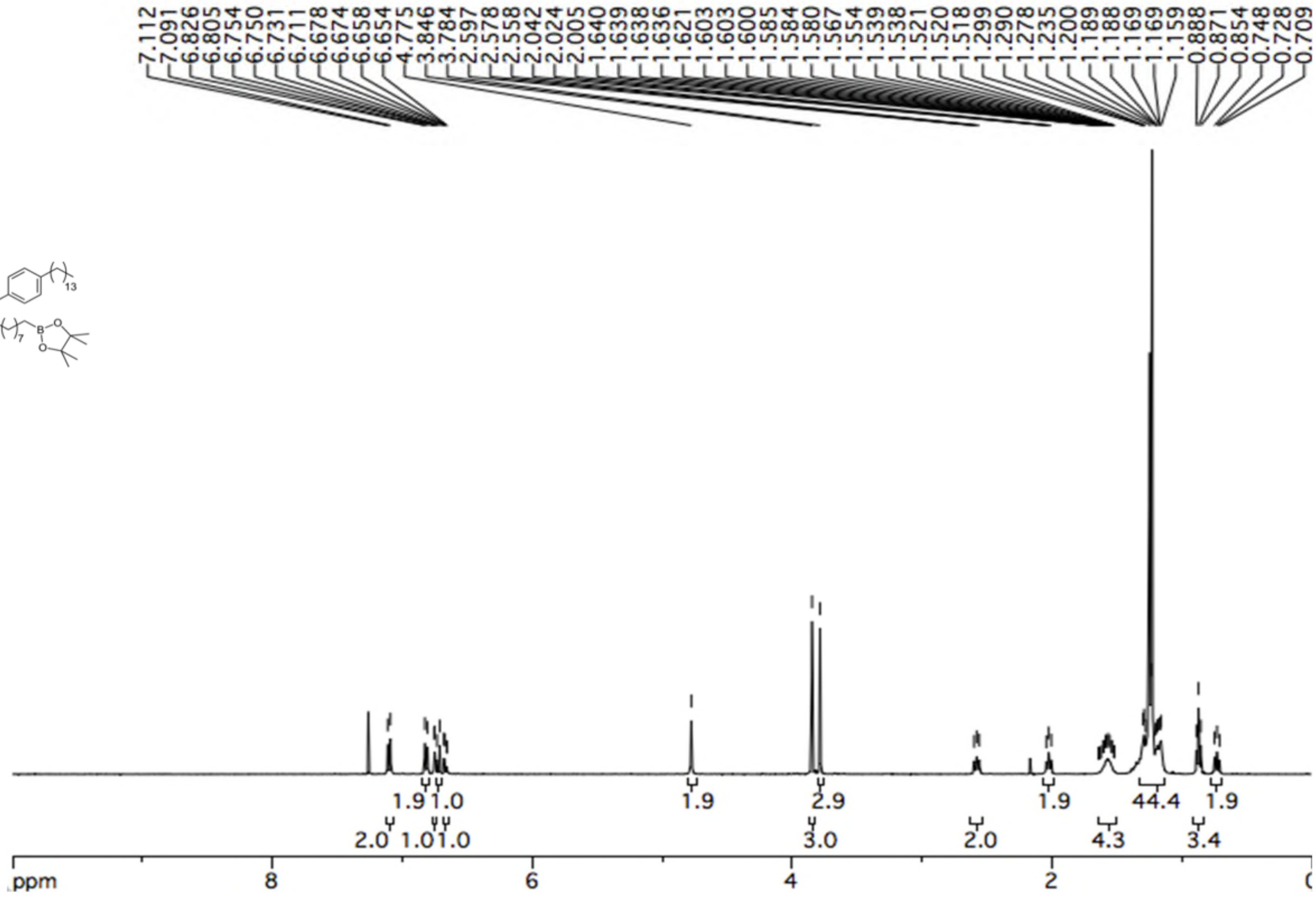

N-(3,4-dimethoxybenzyl)-*N*-(4-tetradecylphenyl)-9-(4,4,5,5-tetramethyl-1,3,2-dioxaborolan-2-yl)nonamide (**34**) – ^{13}C NMR

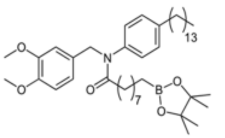

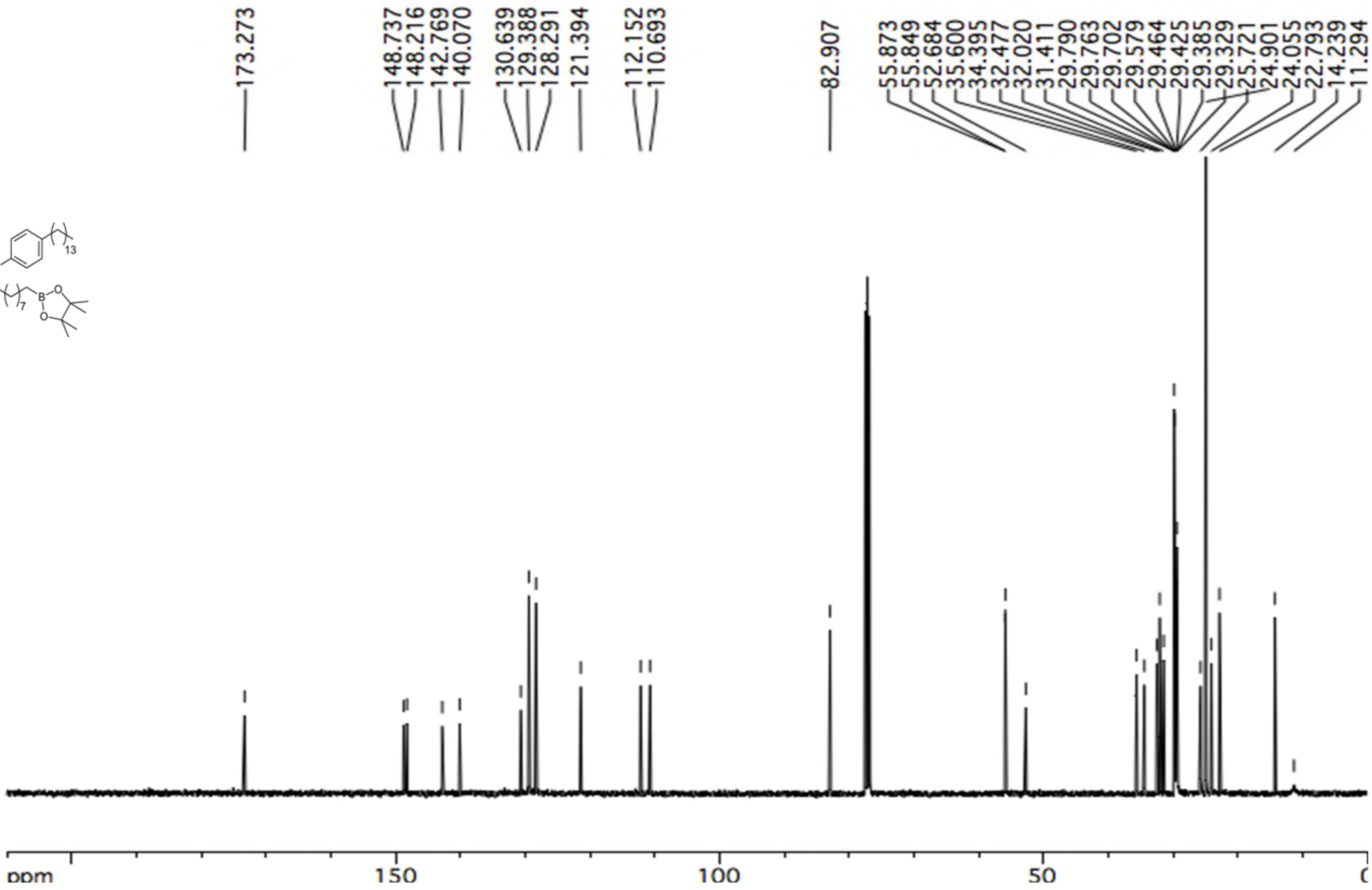

N-(3,4-dimethoxybenzyl)-N-(4-tetradecylphenyl)-9-(4,4,5,5-tetramethyl-1,3,2-dioxaborolan-2-yl)nonamide (**34**) – ^{11}B NMR

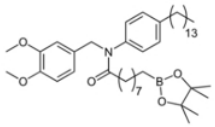

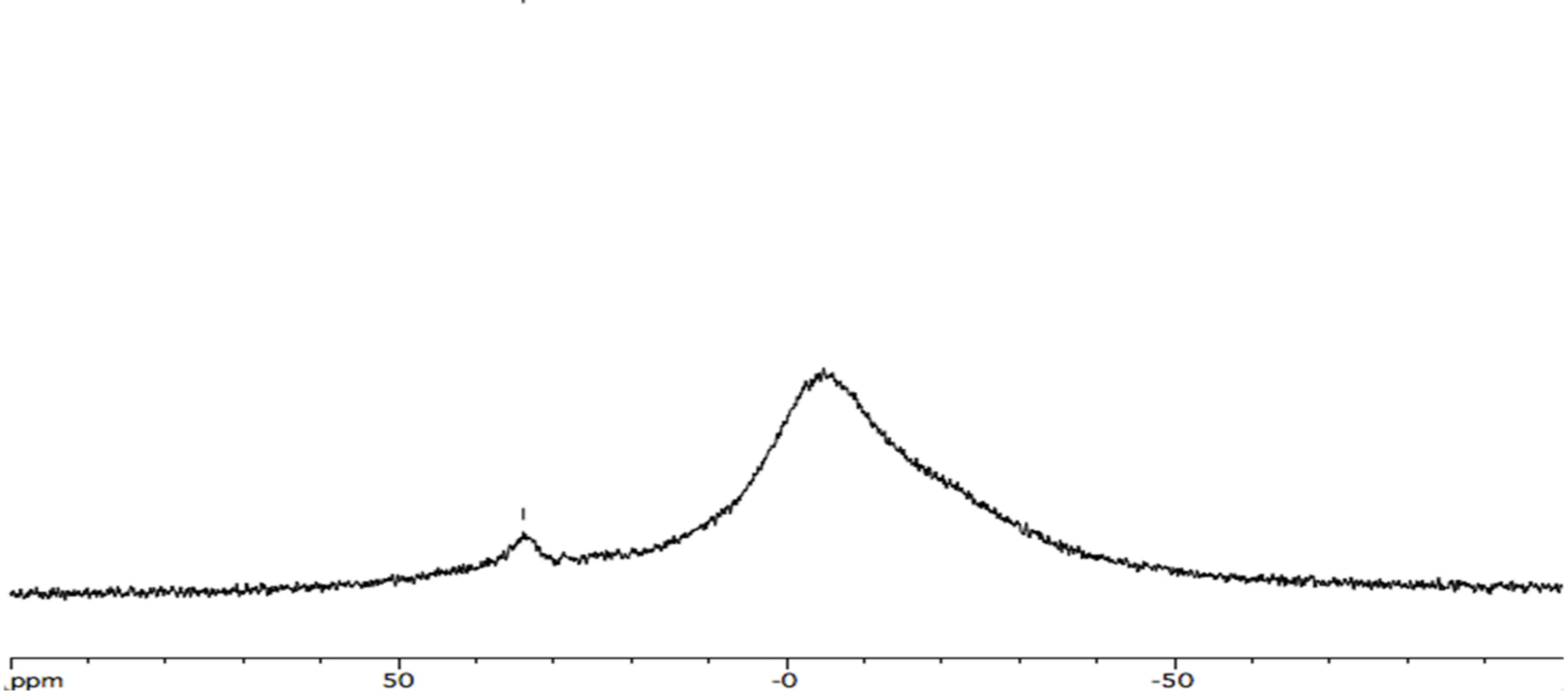

N-(3,4-dimethoxybenzyl)-*N*-(4-tetradecylphenyl)-9-(4,4,5,5-tetramethyl-1,3,2-dioxaborolan-2-yl)nonamide (**34**) – IR

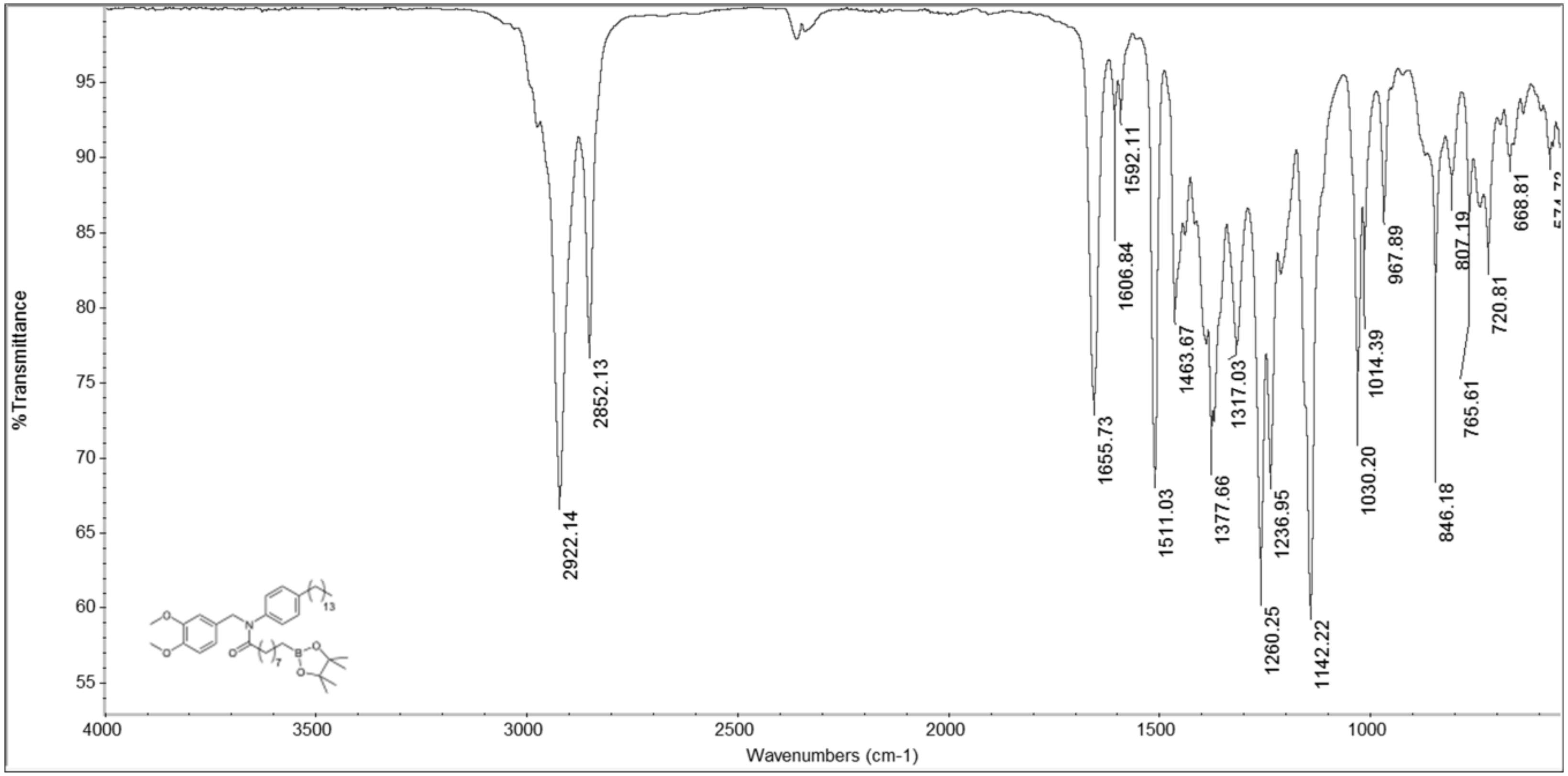

<u>*N*-(3,4-dimethoxybenzyl)-*N*-(4-tetradecylphenyl)-9-(4,4,5,5-tetramethyl-1,3,2-dioxaborolan-2-yl)nonamide (**34**) – HRMS</u>

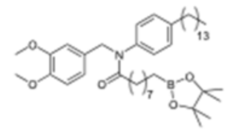

Analysis Info

Analysis Name	D:\Data\Xiao\Nov 05 2020000009.d	Acquisition Date	11/5/2020 11:08:22 AM
Method	Xiao 2.m	Operator	Administrator
Sample Name	JM-74	Instrument	micrOTOF 57
Comment			

Acquisition Parameter

Source Type	ESI	Ion Polarity	Positive	Set Corrector Fill	45 V
Scan Range	n/a	Capillary Exit	90.0 V	Set Pulsar Pull	399 V
Scan Begin	50 m/z	Hexapole RF	125.0 V	Set Pulsar Push	399 V
Scan End	1500 m/z	Skimmer 1	40.0 V	Set Reflector	1300 V
		Hexapole 1	23.0 V	Set Flight Tube	9000 V
				Set Detector TOF	2200 V

Sum Formula	Sigma	m/z	Err [ppm]	Mean Err [ppm]	rdb	N Rule	e⁻
C 44 H 72 B 1 N 1 Na 1 O 5	0.19	728.5396	3.68	3.60	9.50	ok	even

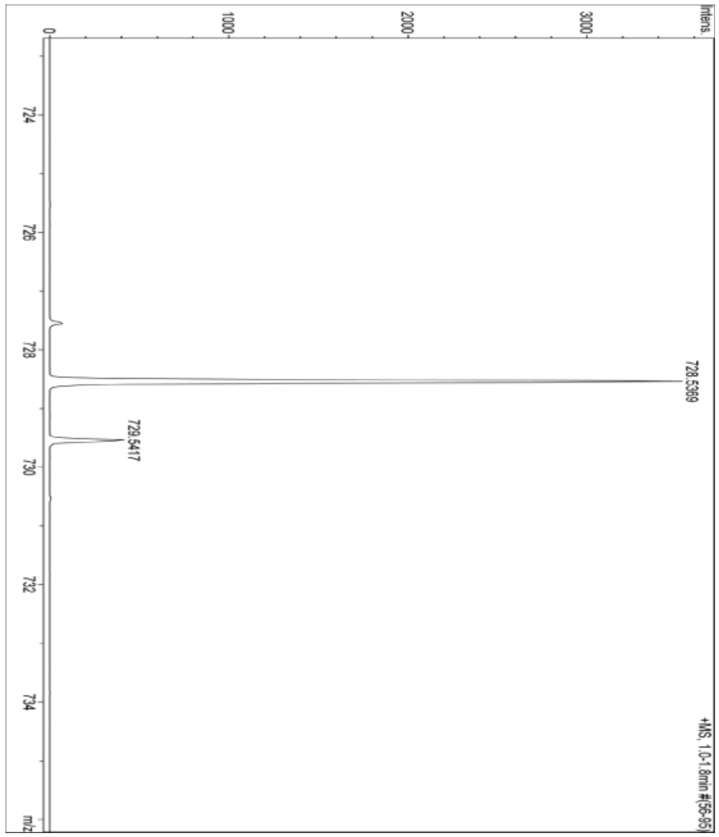

Antimicrobial and Cytotoxicity Data

Antimicrobial Data

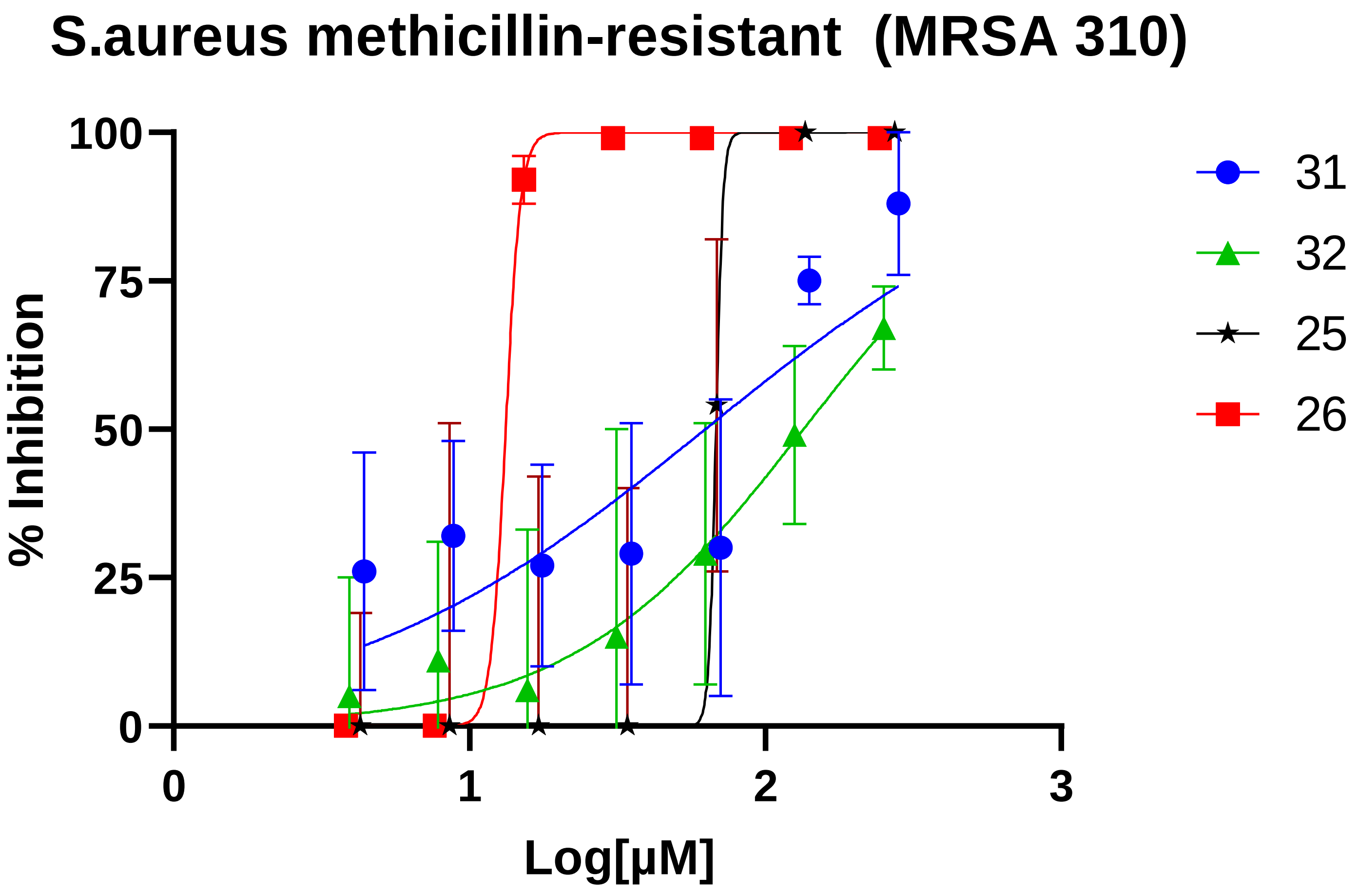

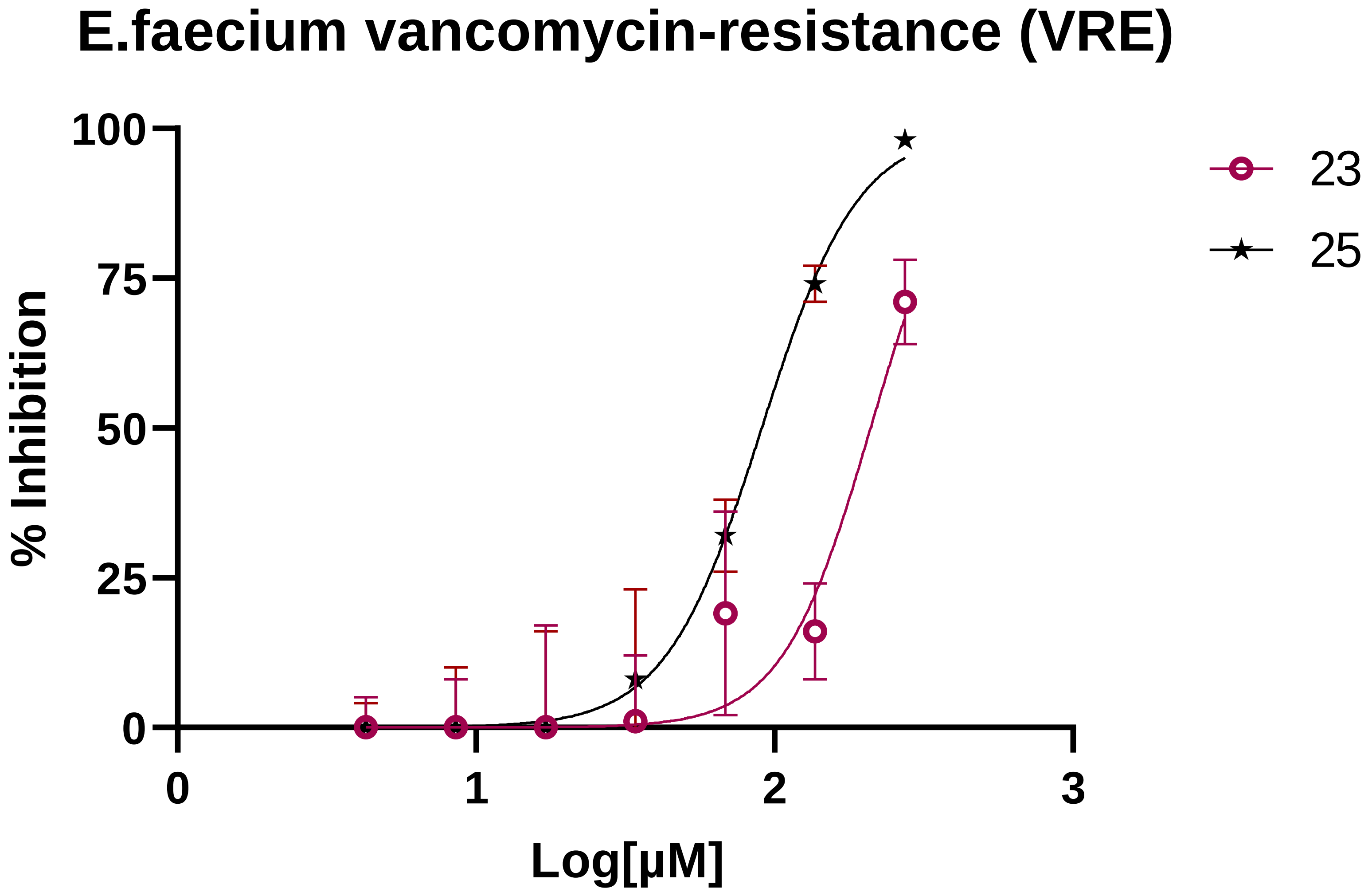
E.faecium vancomycin-resistance (VRE)
100
75
50
25
0
% Inhibition
0
1
2
3
Log[µM]
23
25

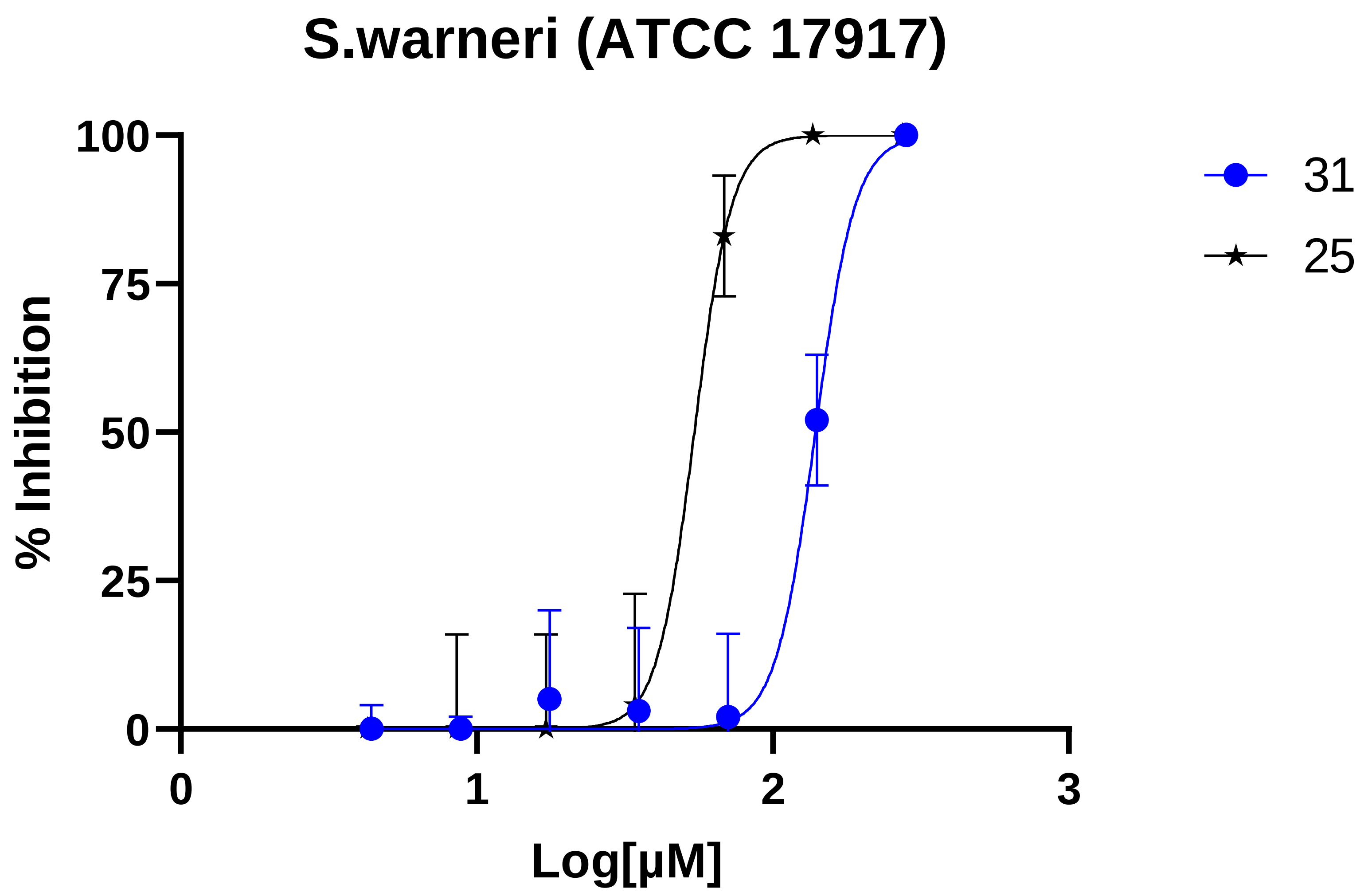
S.warneri (ATCC 17917)
100
75
50
25
0
% Inhibition
0
1
2
3
Log[μM]
31
25

Cytotoxicity Data

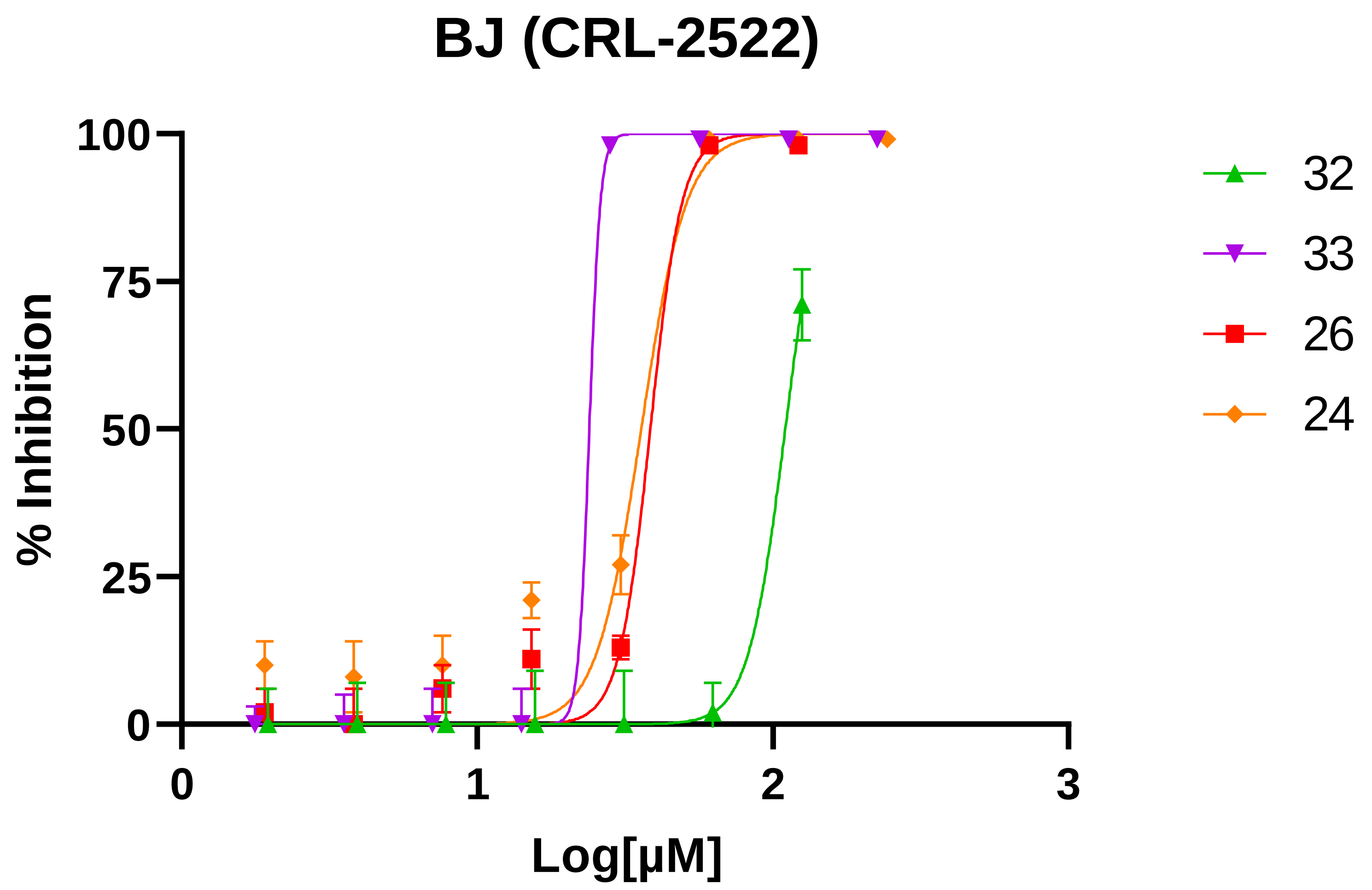

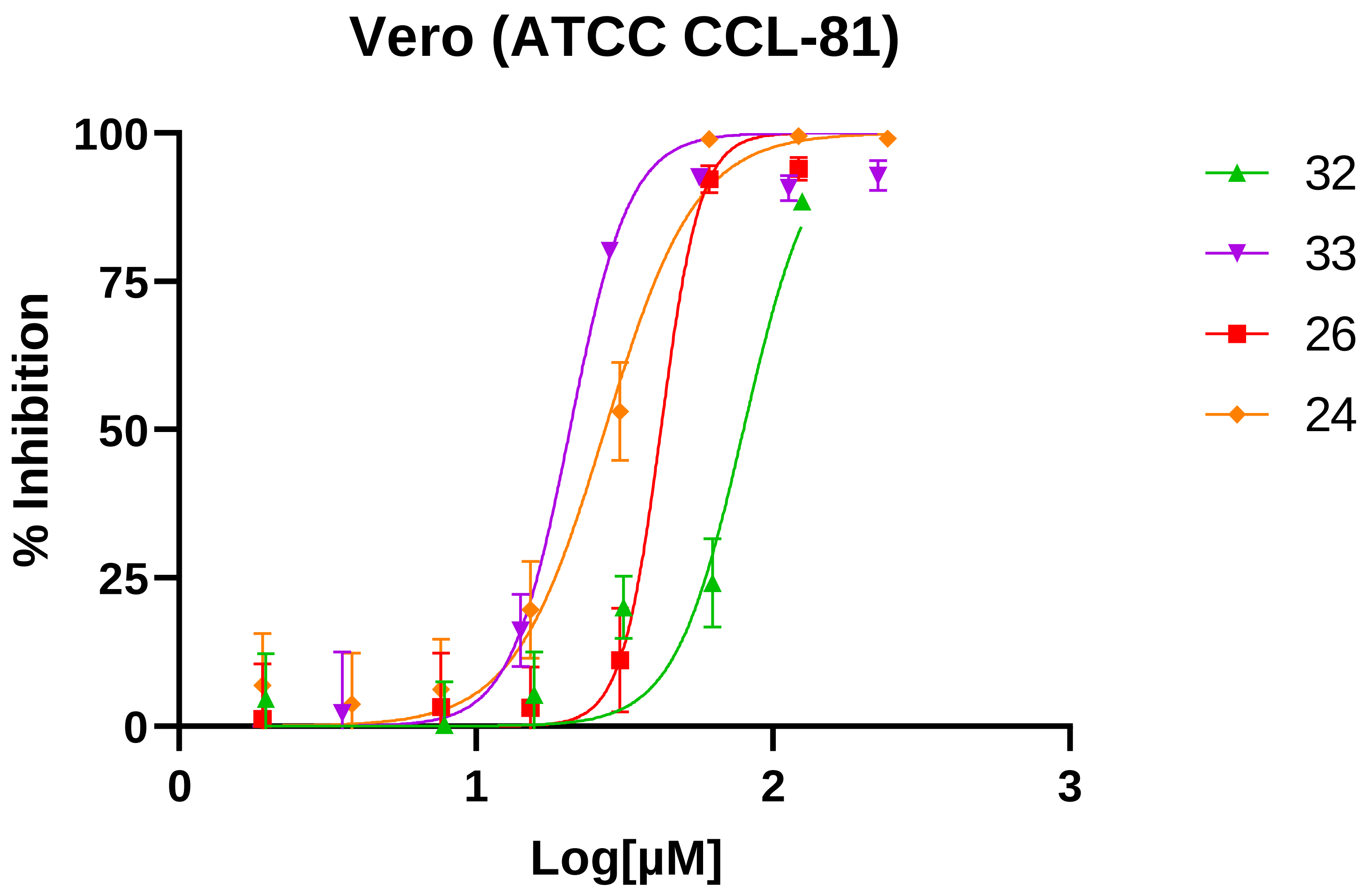
Vero (ATCC CCL-81)
% Inhibition
100
75
50
25
0
0
1
2
3
Log[μM]
32
33
26
24

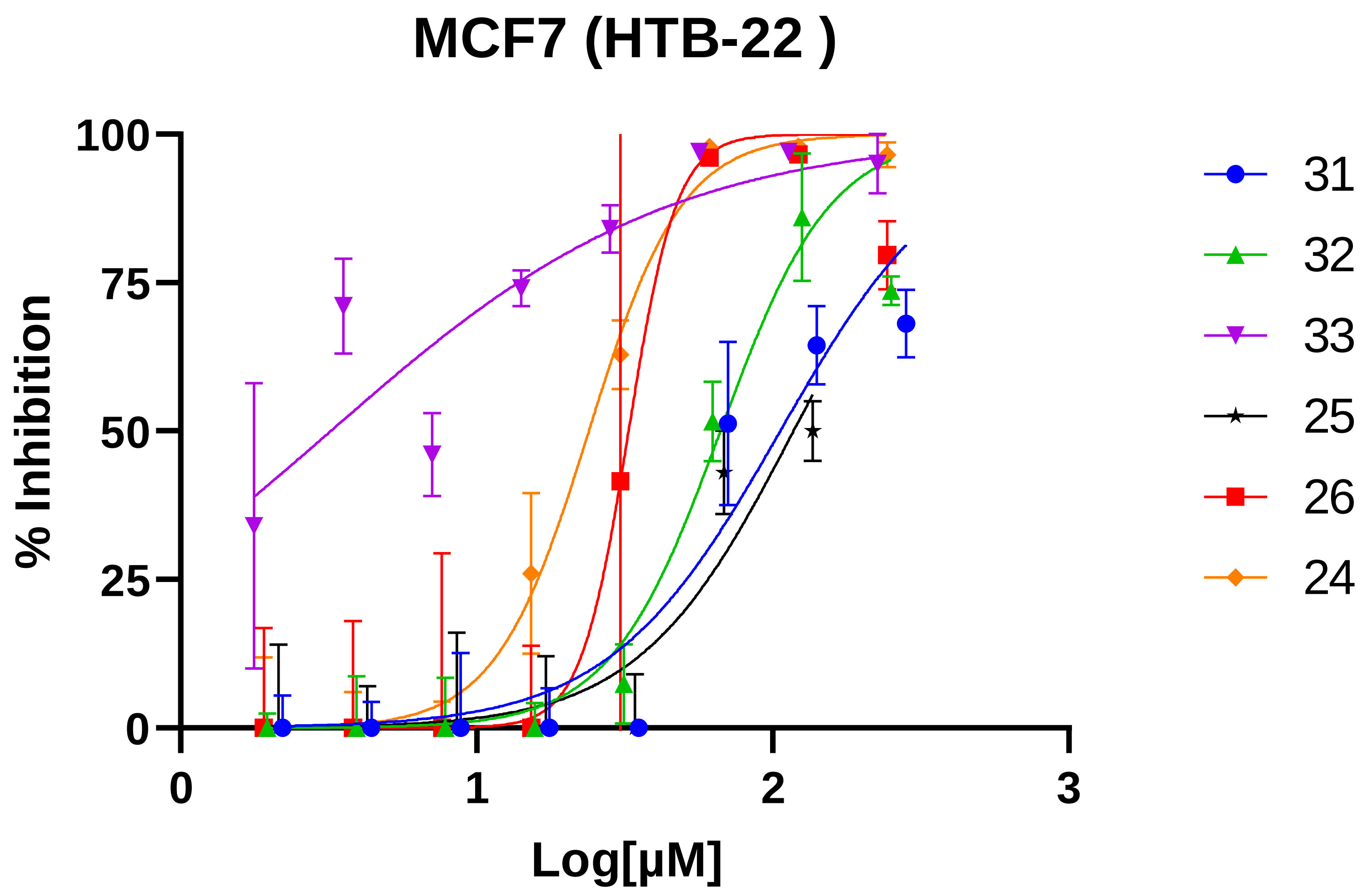
MCF7 (HTB-22)
% Inhibition
100
75
50
25
0
0
1
2
3
Log[μM]
31
32
33
25
26
24

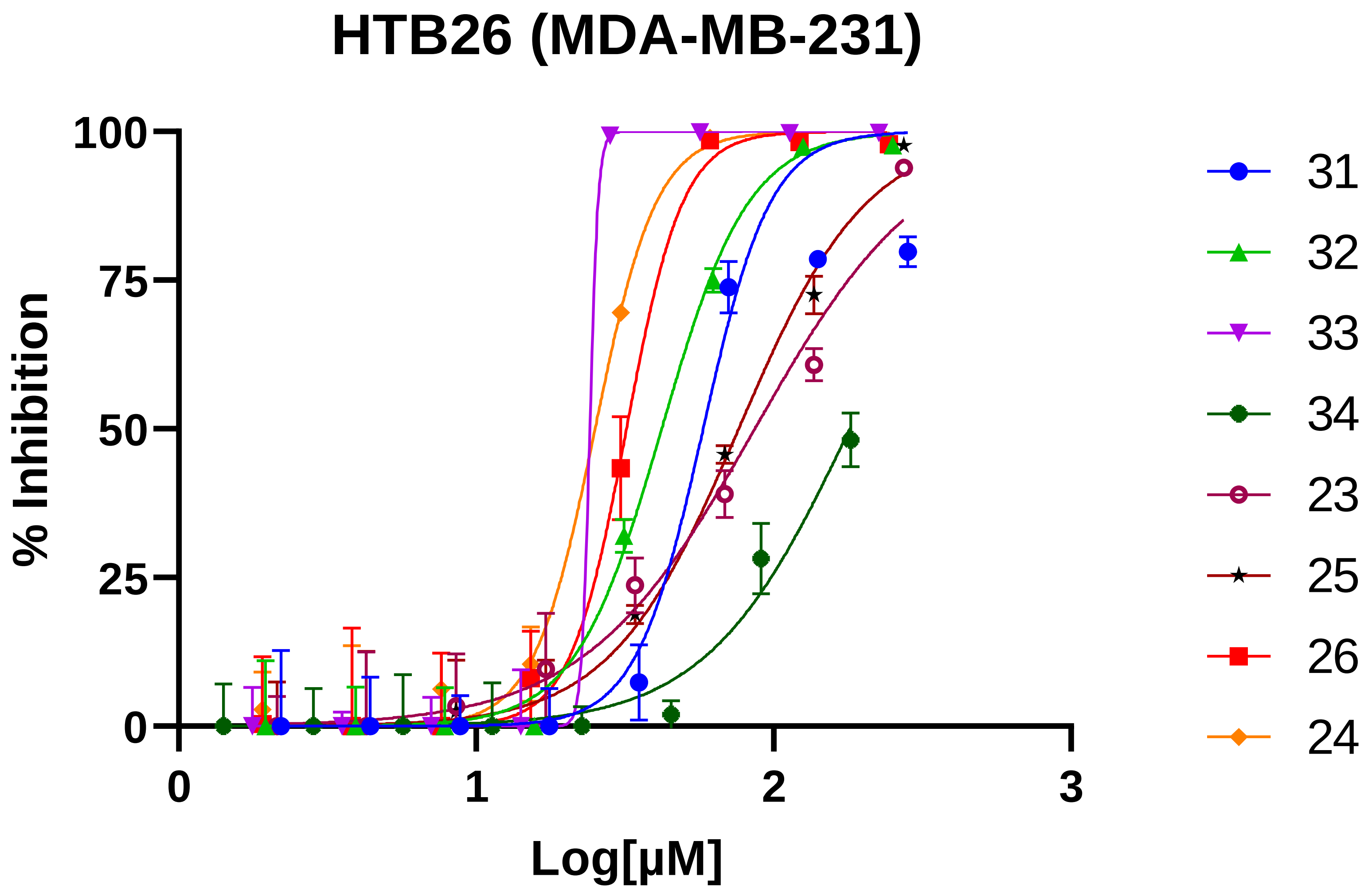

HTB26 (MDA-MB-231)
100
75
50
25
0
% Inhibition
0
1
2
3
Log[µM]
31
32
33
34
23
25
26
24

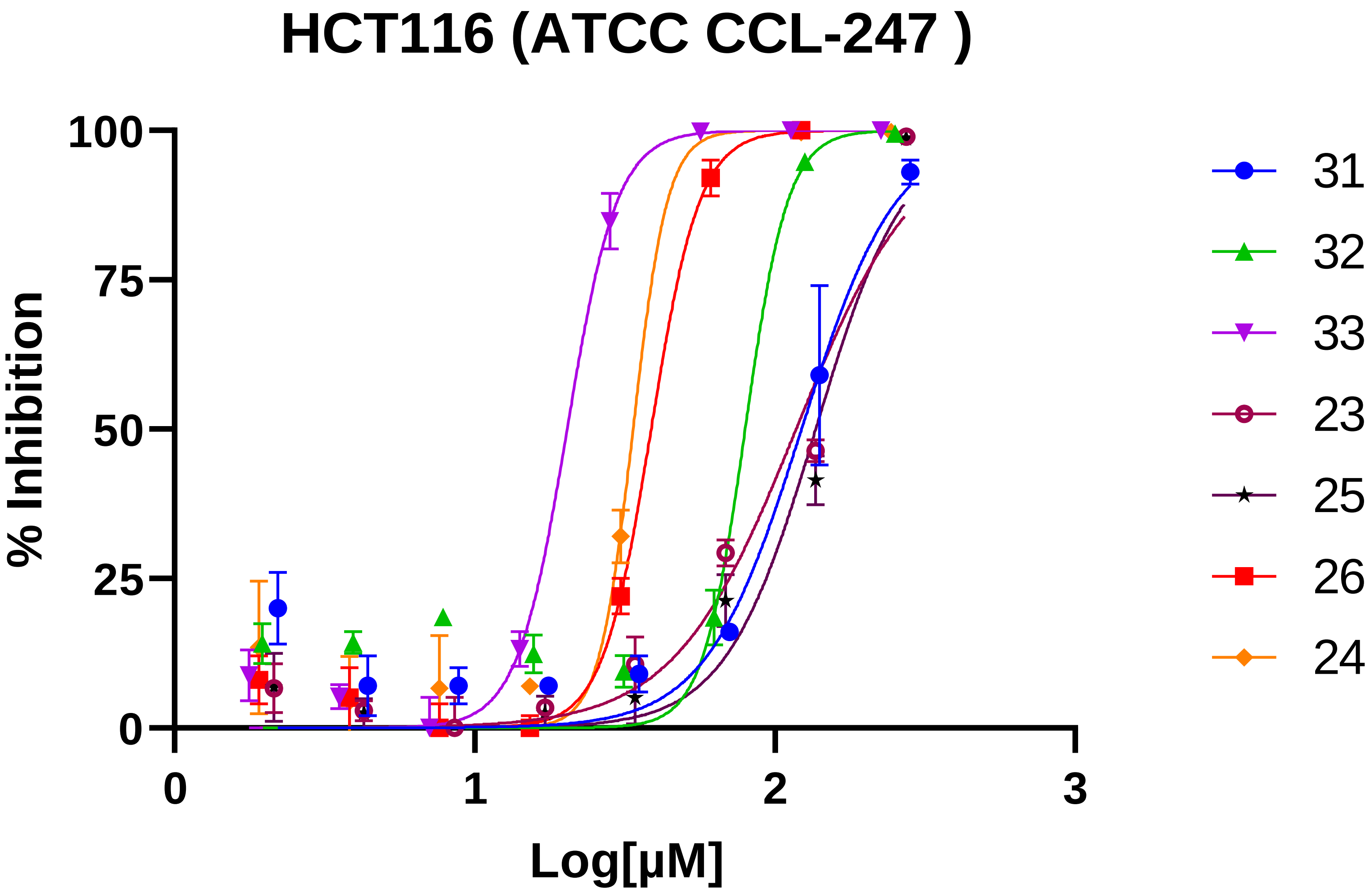

HCT116 (ATCC CCL-247)
% Inhibition
100
75
50
25
0
0
1
2
3
Log[μM]
31
32
33
23
25
26
24
